# Diretriz Brasileira de Dislipidemias e Prevenção da Aterosclerose – 2025

**DOI:** 10.36660/abc.20250640

**Published:** 2025-10-13

**Authors:** Fabiana Hanna Rached, Marcio Hiroshi Miname, Viviane Zorzanelli Rocha, André Zimerman, Fernando Henpin Yue Cesena, Andrei Carvalho Sposito, Raul Dias dos Santos, Paulo Eduardo Ballvé Behr, Henrique Tria Bianco, Renato Jorge Alves, André Arpad Faludi, Elaine dos Reis Coutinho, Francisco Antonio Helfenstein Fonseca, Luiz Sérgio Fernandes de Carvalho, Adriana Bertolami, Aloísio Marchi da Rocha, Ana Paula Marte, Antonio Carlos Palandri Chagas, Bruno Caramelli, Carisi Anne Polanczyk, Carlos Eduardo dos Santos Ferreira, Carlos Vicente Serrano, Daniel Branco de Araujo, Emilio Hideyuki Moriguchi, Fausto J. Pinto, Humberto Graner Moreira, Isabela de Carlos Back, Jose Rocha Faria, Kleisson Antônio Pontes Maia, Marcelo Chiara Bertolami, Marcelo Heitor Vieira Assad, Maria Cristina de Oliveira Izar, Mauricio Alves Barreto, Natasha Slhessarenko Fraife Barreto, Pedro Gabriel Melo de Barros e Silva, Pedro Pimentel, Raul Cavalcante Maranhão, Sergio Emanuel Kaiser, Valeria Arruda Machado, Jose Francisco Kerr Saraiva

**Affiliations:** 1 Hospital das Clínicas da Faculdade de Medicina da Universidade de São Paulo Instituto do Coração São Paulo SP Brasil Instituto do Coração (Incor) do Hospital das Clínicas da Faculdade de Medicina da Universidade de São Paulo (HCFMUSP), São Paulo, SP – Brasil; 2 Hospital Israelita Albert Einstein São Paulo SP Brasil Hospital Israelita Albert Einstein, São Paulo, SP – Brasil; 3 Hospital Moinhos de Vento Porto Alegre RS Brasil Hospital Moinhos de Vento, Porto Alegre, RS – Brasil; 4 Universidade Federal do Rio Grande do Sul Porto Alegre RS Brasil Universidade Federal do Rio Grande do Sul, Porto Alegre, RS – Brasil; 5 Instituto Dante Pazzanese de Cardiologia São Paulo SP Brasil Instituto Dante Pazzanese de Cardiologia, São Paulo, SP – Brasil; 6 Universidade Estadual de Campinas Campinas SP Brasil Universidade Estadual de Campinas (UNICAMP), Campinas, SP – Brasil; 7 Universidade de São Paulo São Paulo SP Brasil Universidade de São Paulo (USP), São Paulo, SP – Brasil; 8 Santa Casa de Porto Alegre Porto Alegre RS Brasil Santa Casa de Porto Alegre, Porto Alegre, RS – Brasil; 9 Universidade Federal de São Paulo São Paulo SP Brasil Universidade Federal de São Paulo (UNIFESP), São Paulo, SP – Brasil; 10 Irmandade da Santa Casa de Misericórdia de São Paulo São Paulo SP Brasil Irmandade da Santa Casa de Misericórdia de São Paulo (ISCMSP), São Paulo, SP – Brasil; 11 Faculdade de Ciências Médicas da Santa Casa de São Paulo São Paulo SP Brasil Faculdade de Ciências Médicas da Santa Casa de São Paulo (FCMSCSP), São Paulo, SP – Brasil; 12 Pontifícia Universidade Católica de Campinas Campinas SP Brasil Pontifícia Universidade Católica de Campinas, Campinas, SP – Brasil; 13 Clarity Healthcare Desenvolvimento de Software LTDA Jundiaí SP Brasil Clarity Healthcare Desenvolvimento de Software LTDA, Jundiaí, SP – Brasil; 14 Centro Universitário Faculdade de Medicina do ABC Santo André SP Brasil Centro Universitário Faculdade de Medicina do ABC, Santo André, SP – Brasil; 15 Hospital de Clínicas de Porto Alegre Porto Alegre RS Brasil Hospital de Clínicas de Porto Alegre, Porto Alegre, RS – Brasil; 16 Faculdade de Medicina da Universidade de Lisboa Lisboa Portugal Faculdade de Medicina da Universidade de Lisboa, Lisboa – Portugal; 17 Universidade Federal de Goiás Goiânia GO Brasil Universidade Federal de Goiás (UFG), Goiânia, GO – Brasil; 18 Universidade Federal de Santa Catarina Florianópolis SC Brasil Universidade Federal de Santa Catarina, Florianópolis, SC – Brasil; 19 Pontifícia Universidade Católica do Paraná Curitiba PR Brasil Pontifícia Universidade Católica do Paraná, Curitiba, PR – Brasil; 20 Faculdade Ciências Médicas de Minas Gerais Belo Horizonte MG Brasil Faculdade Ciências Médicas de Minas Gerais, Belo Horizonte, MG – Brasil; 21 Instituto Nacional de Cardiologia Rio de Janeiro RJ Brasil Instituto Nacional de Cardiologia (INC), Rio de Janeiro, RJ – Brasil; 22 Escola Bahiana de Medicina e Saúde Pública Salvador BA Brasil Escola Bahiana de Medicina e Saúde Pública, Salvador, BA – Brasil; 23 Universidade Federal da Bahia Hospital Ana Nery Salvador BA Brasil Hospital Ana Nery, Universidade Federal da Bahia (UFBA), Salvador, BA – Brasil; 24 Hospital Fundação Bahiana de Cardiologia e Combate ao Câncer Salvador BA Brasil Hospital Fundação Bahiana de Cardiologia e Combate ao Câncer, Salvador, BA – Brasil; 25 Diagnósticos da América Cuiabá MT Brasil Diagnósticos da América (DASA), Cuiabá, MT – Brasil; 26 Universidade Federal de Mato Grosso Cuiabá MT Brasil Universidade Federal de Mato Grosso (UFMT), Cuiabá, MT – Brasil; 27 Hospital do Coração São Paulo SP Brasil Hospital do Coração (Hcor), São Paulo, SP – Brasil; 28 Centro Universitário São Camilo São Paulo SP Brasil Centro Universitário São Camilo, São Paulo, SP – Brasil; 29 Brazilian Clinical Research Institute São Paulo SP Brasil Brazilian Clinical Research Institute, São Paulo, SP – Brasil; 30 Hospital Nossa Senhora Conceição de Porto Alegre Porto Alegre RS Brasil Hospital Nossa Senhora Conceição de Porto Alegre, Porto Alegre, RS – Brasil; 31 Universidade do Estado do Rio de Janeiro Rio de Janeiro RJ Brasil Universidade do Estado do Rio de Janeiro (UERJ), Rio de Janeiro, RJ – Brasil

**Table:** 

Diretriz Brasileira de Dislipidemias e Prevenção da Aterosclerose – 2025	
O relatório abaixo lista as declarações de interesse conforme relatadas à SBC pelos especialistas durante o período de desenvolvimento desta diretriz, 2024/2025.	
Especialista	Tipo de relacionamento com a indústria
Adriana Bertolami	Declaração financeira A - Pagamento de qualquer espécie e desde que economicamente apreciáveis, feitos a (i) você, (ii) ao seu cônjuge/ companheiro ou a qualquer outro membro que resida com você, (iii) a qualquer pessoa jurídica em que qualquer destes seja controlador, sócio, acionista ou participante, de forma direta ou indireta, recebimento por palestras, aulas, atuação como proctor de treinamentos, remunerações, honorários pagos por participações em conselhos consultivos, de investigadores, ou outros comitês, etc. Provenientes da indústria farmacêutica, de órteses, próteses, equipamentos e implantes, brasileiras ou estrangeiras: - Daiichi Sankyo.
Aloísio Marchi da Rocha	Declaração financeira A - Pagamento de qualquer espécie e desde que economicamente apreciáveis, feitos a (i) você, (ii) ao seu cônjuge/ companheiro ou a qualquer outro membro que resida com você, (iii) a qualquer pessoa jurídica em que qualquer destes seja controlador, sócio, acionista ou participante, de forma direta ou indireta, recebimento por palestras, aulas, atuação como proctor de treinamentos, remunerações, honorários pagos por participações em conselhos consultivos, de investigadores, ou outros comitês, etc. Provenientes da indústria farmacêutica, de órteses, próteses, equipamentos e implantes, brasileiras ou estrangeiras: - Amgen: Repatha; AstraZeneca: Forxiga; Bayer: Xarelto, Firialta; BMS: Camzyos; Boehringer Ingelheim: Jardiance; Daiichi Sankyo: Nustendi; GSK: Shingrix; Novartis: Entresto, Sybrava; NovoNordisk: Ozempic; Pfizer: Tafamidis, Amiloidose; Servier: Vastarel, Triplixam; Viatris: Inspra. B - Financiamento de pesquisas sob sua responsabilidade direta/pessoal (direcionado ao departamento ou instituição) provenientes da indústria farmacêutica, de órteses, próteses, equipamentos e implantes, brasileiras ou estrangeiras: - Astrazeneca: Baxduo; Bayer: Finerinona; Lilly: Tirzepatida; Novartis: Entresto; Novo Nordisk: Ziltivequimabe. Outros relacionamentos Financiamento de atividades de educação médica continuada, incluindo viagens, hospedagens e inscrições para congressos e cursos, provenientes da indústria farmacêutica, de órteses, próteses, equipamentos e implantes, brasileiras ou estrangeiras: - Lilly; AstraZeneca; NovoNordisk; Servier.
Ana Paula Marte	Declaração financeira A - Pagamento de qualquer espécie e desde que economicamente apreciáveis, feitos a (i) você, (ii) ao seu cônjuge/ companheiro ou a qualquer outro membro que resida com você, (iii) a qualquer pessoa jurídica em que qualquer destes seja controlador, sócio, acionista ou participante, de forma direta ou indireta, recebimento por palestras, aulas, atuação como proctor de treinamentos, remunerações, honorários pagos por participações em conselhos consultivos, de investigadores, ou outros comitês, etc. Provenientes da indústria farmacêutica, de órteses, próteses, equipamentos e implantes, brasileiras ou estrangeiras: - Fundação Zerbini: Ensino InCor; Ultragenyx: Evinacumabe; PTC therapeutics: Volanesorsen.
André Arpad Faludi	Nada a ser declarado
André Zimerman	Declaração financeira A - Pagamento de qualquer espécie e desde que economicamente apreciáveis, feitos a (i) você, (ii) ao seu cônjuge/ companheiro ou a qualquer outro membro que resida com você, (iii) a qualquer pessoa jurídica em que qualquer destes seja controlador, sócio, acionista ou participante, de forma direta ou indireta, recebimento por palestras, aulas, atuação como proctor de treinamentos, remunerações, honorários pagos por participações em conselhos consultivos, de investigadores, ou outros comitês, etc. Provenientes da indústria farmacêutica, de órteses, próteses, equipamentos e implantes, brasileiras ou estrangeiras: - Novartis: Sybrava.
Andrei C. Sposito	Declaração financeira A - Pagamento de qualquer espécie e desde que economicamente apreciáveis, feitos a (i) você, (ii) ao seu cônjuge/ companheiro ou a qualquer outro membro que resida com você, (iii) a qualquer pessoa jurídica em que qualquer destes seja controlador, sócio, acionista ou participante, de forma direta ou indireta, recebimento por palestras, aulas, atuação como proctor de treinamentos, remunerações, honorários pagos por participações em conselhos consultivos, de investigadores, ou outros comitês, etc. Provenientes da indústria farmacêutica, de órteses, próteses, equipamentos e implantes, brasileiras ou estrangeiras: - Lilly, Novo Nordisk, Daiichi Sankyo. B - Financiamento de pesquisas sob sua responsabilidade direta/pessoal (direcionado ao departamento ou instituição) provenientes da indústria farmacêutica, de órteses, próteses, equipamentos e implantes, brasileiras ou estrangeiras: - AstraZeneca. Outros relacionamentos Financiamento de atividades de educação médica continuada, incluindo viagens, hospedagens e inscrições para congressos e cursos, provenientes da indústria farmacêutica, de órteses, próteses, equipamentos e implantes, brasileiras ou estrangeiras: - Daiichi, Novo Nordisk.
Antonio Carlos Palandri Chagas	Declaração financeira A - Pagamento de qualquer espécie e desde que economicamente apreciáveis, feitos a (i) você, (ii) ao seu cônjuge/ companheiro ou a qualquer outro membro que resida com você, (iii) a qualquer pessoa jurídica em que qualquer destes seja controlador, sócio, acionista ou participante, de forma direta ou indireta, recebimento por palestras, aulas, atuação como proctor de treinamentos, remunerações, honorários pagos por participações em conselhos consultivos, de investigadores, ou outros comitês, etc. Provenientes da indústria farmacêutica, de órteses, próteses, equipamentos e implantes, brasileiras ou estrangeiras: - Instituto de Vita.
Bruno Caramelli	Nada a ser declarado
Carisi Anne Polanczyk	Declaração financeira A - Pagamento de qualquer espécie e desde que economicamente apreciáveis, feitos a (i) você, (ii) ao seu cônjuge/ companheiro ou a qualquer outro membro que resida com você, (iii) a qualquer pessoa jurídica em que qualquer destes seja controlador, sócio, acionista ou participante, de forma direta ou indireta, recebimento por palestras, aulas, atuação como proctor de treinamentos, remunerações, honorários pagos por participações em conselhos consultivos, de investigadores, ou outros comitês, etc. Provenientes da indústria farmacêutica, de órteses, próteses, equipamentos e implantes, brasileiras ou estrangeiras: - AstraZeneca; Amgen; Abbott: HeartMate; Bayer: Xarelto, Lipidil; Baxter; Pfizer; BMS; Roche. Novartis: Inclisiran; Organon; Sanofi. B - Financiamento de pesquisas sob sua responsabilidade direta/pessoal (direcionado ao departamento ou instituição) provenientes da indústria farmacêutica, de órteses, próteses, equipamentos e implantes, brasileiras ou estrangeiras: - AstraZeneca, Amgen, Abbott, Bayer, Novartis, Sanofi, Roche: pesquisas não relacionadas a produtos específicos. C - Financiamento de pesquisa (pessoal), cujas receitas tenham sido provenientes da indústria farmacêutica, de órteses, próteses, equipamentos e implantes, brasileiras ou estrangeiras: - Bayer: Xarelto, Lipidil; Pfizer; BMS; Roche; Organon; Sanofi. Sem área específica. Outros relacionamentos Financiamento de atividades de educação médica continuada, incluindo viagens, hospedagens e inscrições para congressos e cursos, provenientes da indústria farmacêutica, de órteses, próteses, equipamentos e implantes, brasileiras ou estrangeiras: - AstraZeneca; Amgen; Bayer: Lipidil; Pfizer; BMS; Sanofi. Cursos sem produtos específicos. Participação societária de qualquer natureza e qualquer valor economicamente apreciável de empresas na área de saúde, de ensino ou em empresas concorrentes ou fornecedoras da SBC: - Área de consultoria em saúde: PEV.
Carlos Eduardo dos Santos Ferreira	Nada a ser declarado
Carlos Vicente Serrano Junior	Nada a ser declarado
Daniel Branco de Araujo	Declaração financeira A - Pagamento de qualquer espécie e desde que economicamente apreciáveis, feitos a (i) você, (ii) ao seu cônjuge/ companheiro ou a qualquer outro membro que resida com você, (iii) a qualquer pessoa jurídica em que qualquer destes seja controlador, sócio, acionista ou participante, de forma direta ou indireta, recebimento por palestras, aulas, atuação como proctor de treinamentos, remunerações, honorários pagos por participações em conselhos consultivos, de investigadores, ou outros comitês, etc. Provenientes da indústria farmacêutica, de órteses, próteses, equipamentos e implantes, brasileiras ou estrangeiras: - Novo Nordisk: Wegovy; Sanofi: Zinpass Eze; Daiichi Sankyo: Nustendi; AstraZeneca: Forxiga; EMS: Linadib; Boehringer: Jardiance. B - Financiamento de pesquisas sob sua responsabilidade direta/pessoal (direcionado ao departamento ou instituição) provenientes da indústria farmacêutica, de órteses, próteses, equipamentos e implantes, brasileiras ou estrangeiras: - MSD: MK0616. Outros relacionamentos Financiamento de atividades de educação médica continuada, incluindo viagens, hospedagens e inscrições para congressos e cursos, provenientes da indústria farmacêutica, de órteses, próteses, equipamentos e implantes, brasileiras ou estrangeiras: - Sanofi: Zinpass Eze.
Elaine dos Reis Coutinho	Declaração financeira A - Pagamento de qualquer espécie e desde que economicamente apreciáveis, feitos a (i) você, (ii) ao seu cônjuge/ companheiro ou a qualquer outro membro que resida com você, (iii) a qualquer pessoa jurídica em que qualquer destes seja controlador, sócio, acionista ou participante, de forma direta ou indireta, recebimento por palestras, aulas, atuação como proctor de treinamentos, remunerações, honorários pagos por participações em conselhos consultivos, de investigadores, ou outros comitês, etc. Provenientes da indústria farmacêutica, de órteses, próteses, equipamentos e implantes, brasileiras ou estrangeiras: - Novartis: Sybrava; Biolab: Repatha; Daiichi Sankyo: Nustendi. Outros relacionamentos Financiamento de atividades de educação médica continuada, incluindo viagens, hospedagens e inscrições para congressos e cursos, provenientes da indústria farmacêutica, de órteses, próteses, equipamentos e implantes, brasileiras ou estrangeiras: - Novartis: Sybrava; Biolab: Repatha; Daiichi Sankyo: Nustendi.. Vínculo empregatício com a indústria farmacêutica, de órteses, próteses, equipamentos e implantes, brasileiras ou estrangeiras, assim como se tem relação vínculo empregatício com operadoras de planos de saúde ou em auditorias médicas (incluindo meio período) durante o ano para o qual você está declarando: - Unimed Campinas.
Emilio Hideyuki Moriguchi	Declaração financeira A - Pagamento de qualquer espécie e desde que economicamente apreciáveis, feitos a (i) você, (ii) ao seu cônjuge/ companheiro ou a qualquer outro membro que resida com você, (iii) a qualquer pessoa jurídica em que qualquer destes seja controlador, sócio, acionista ou participante, de forma direta ou indireta, recebimento por palestras, aulas, atuação como proctor de treinamentos, remunerações, honorários pagos por participações em conselhos consultivos, de investigadores, ou outros comitês, etc. Provenientes da indústria farmacêutica, de órteses, próteses, equipamentos e implantes, brasileiras ou estrangeiras: - Biolab: Repatha; Biolab: Livalo; Novartis: Sybrava. B - Financiamento de pesquisas sob sua responsabilidade direta/pessoal (direcionado ao departamento ou instituição) provenientes da indústria farmacêutica, de órteses, próteses, equipamentos e implantes, brasileiras ou estrangeiras: - Human Life CORD Japan inc.: Sarcopenia. Outros relacionamentos Financiamento de atividades de educação médica continuada, incluindo viagens, hospedagens e inscrições para congressos e cursos, provenientes da indústria farmacêutica, de órteses, próteses, equipamentos e implantes, brasileiras ou estrangeiras: - Biolab: Repatha; Biolab: Livalo; Novartis Sybrava.
Fabiana Hanna Rached	Declaração financeira A - Pagamento de qualquer espécie e desde que economicamente apreciáveis, feitos a (i) você, (ii) ao seu cônjuge/ companheiro ou a qualquer outro membro que resida com você, (iii) a qualquer pessoa jurídica em que qualquer destes seja controlador, sócio, acionista ou participante, de forma direta ou indireta, recebimento por palestras, aulas, atuação como proctor de treinamentos, remunerações, honorários pagos por participações em conselhos consultivos, de investigadores, ou outros comitês, etc. Provenientes da indústria farmacêutica, de órteses, próteses, equipamentos e implantes, brasileiras ou estrangeiras: - Novo Nordisk: semaglutida; Novartis: inclisirana; Daiichi Sankyo: ácido bempedoico. Outros relacionamentos Financiamento de atividades de educação médica continuada, incluindo viagens, hospedagens e inscrições para congressos e cursos, provenientes da indústria farmacêutica, de órteses, próteses, equipamentos e implantes, brasileiras ou estrangeiras: - Novo Nordisk: semaglutida; Novartis: inclisirana; Daiichi Sankyo: ácido bempedoico.
Fausto J. Pinto	Declaração financeira A - Pagamento de qualquer espécie e desde que economicamente apreciáveis, feitos a (i) você, (ii) ao seu cônjuge/ companheiro ou a qualquer outro membro que resida com você, (iii) a qualquer pessoa jurídica em que qualquer destes seja controlador, sócio, acionista ou participante, de forma direta ou indireta, recebimento por palestras, aulas, atuação como proctor de treinamentos, remunerações, honorários pagos por participações em conselhos consultivos, de investigadores, ou outros comitês, etc. Provenientes da indústria farmacêutica, de órteses, próteses, equipamentos e implantes, brasileiras ou estrangeiras: - Boehringer Ingelheim; Daiichi Sankyo; Novartis; Servier; CSL Vifor; Zydus. B - Financiamento de pesquisas sob sua responsabilidade direta/pessoal (direcionado ao departamento ou instituição) provenientes da indústria farmacêutica, de órteses, próteses, equipamentos e implantes, brasileiras ou estrangeiras: - Abbott; Biosensors; Medtronic; Novartis; Pfizer. C - Financiamento de pesquisa (pessoal), cujas receitas tenham sido provenientes da indústria farmacêutica, de órteses, próteses, equipamentos e implantes, brasileiras ou estrangeiras: - Boehringer Ingelheim; Daiichi Sankyo; Medtronic; Novartis; Novo Nordisk; Servier; CSL Vifor.
Fernando Henpin Yue Cesena	Nada a ser declarado
Francisco Antonio Helfenstein Fonseca	Declaração financeira A - Pagamento de qualquer espécie e desde que economicamente apreciáveis, feitos a (i) você, (ii) ao seu cônjuge/ companheiro ou a qualquer outro membro que resida com você, (iii) a qualquer pessoa jurídica em que qualquer destes seja controlador, sócio, acionista ou participante, de forma direta ou indireta, recebimento por palestras, aulas, atuação como proctor de treinamentos, remunerações, honorários pagos por participações em conselhos consultivos, de investigadores, ou outros comitês, etc. Provenientes da indústria farmacêutica, de órteses, próteses, equipamentos e implantes, brasileiras ou estrangeiras: - Libbs: Artag, antiplaquetário. B - Financiamento de pesquisas sob sua responsabilidade direta/pessoal (direcionado ao departamento ou instituição) provenientes da indústria farmacêutica, de órteses, próteses, equipamentos e implantes, brasileiras ou estrangeiras: - Libbs: Plenance, dislipidemia. C - Financiamento de pesquisa (pessoal), cujas receitas tenham sido provenientes da indústria farmacêutica, de órteses, próteses, equipamentos e implantes, brasileiras ou estrangeiras: - Hypera: Addera, vitamina D. Outros relacionamentos Financiamento de atividades de educação médica continuada, incluindo viagens, hospedagens e inscrições para congressos e cursos, provenientes da indústria farmacêutica, de órteses, próteses, equipamentos e implantes, brasileiras ou estrangeiras: - NovoNordisk: Wegovy, Analogo GLP-1.
Henrique Tria Bianco	Outros relacionamentos Financiamento de atividades de educação médica continuada, incluindo viagens, hospedagens e inscrições para congressos e cursos, provenientes da indústria farmacêutica, de órteses, próteses, equipamentos e implantes, brasileiras ou estrangeiras: - Novo Nordisk: semaglutida, diabetes.
Humberto Graner Moreira	Declaração financeira A - Pagamento de qualquer espécie e desde que economicamente apreciáveis, feitos a (i) você, (ii) ao seu cônjuge/ companheiro ou a qualquer outro membro que resida com você, (iii) a qualquer pessoa jurídica em que qualquer destes seja controlador, sócio, acionista ou participante, de forma direta ou indireta, recebimento por palestras, aulas, atuação como proctor de treinamentos, remunerações, honorários pagos por participações em conselhos consultivos, de investigadores, ou outros comitês, etc. Provenientes da indústria farmacêutica, de órteses, próteses, equipamentos e implantes, brasileiras ou estrangeiras: - Pfizer: amiloidose e imunizações; Novo Nordisk: obesidade e inflamação; Novartis: dislipidemia; Daichii-Sankyo: dislipidemia; Bayer: cardio-oncologia. Outros relacionamentos Financiamento de atividades de educação médica continuada, incluindo viagens, hospedagens e inscrições para congressos e cursos, provenientes da indústria farmacêutica, de órteses, próteses, equipamentos e implantes, brasileiras ou estrangeiras: - Novo Nordisk: obesidade.
Isabela de Carlos Back	Outros relacionamentos Financiamento de atividades de educação médica continuada, incluindo viagens, hospedagens e inscrições para congressos e cursos, provenientes da indústria farmacêutica, de órteses, próteses, equipamentos e implantes, brasileiras ou estrangeiras: - Ultragenyx: Evckeeza.
José Francisco Kerr Saraiva	Declaração financeira C - Financiamento de pesquisa (pessoal), cujas receitas tenham sido provenientes da indústria farmacêutica, de órteses, próteses, equipamentos e implantes, brasileiras ou estrangeiras: - Bayer: finerinona; Novo Nordisk: semaglutida; AstraZeneca: ciclosilicato de Zirconio, dapagliflozina; Amgen: evolocumabe; Boehringer Ingelheimer: empagliflozina; Lilly: tirzepatida, viatris atorvastatina; Daichii Sankyo: ácido bempedoico/Edoxabana; Mantecorp: rosuvastatina. Outros relacionamentos Financiamento de atividades de educação médica continuada, incluindo viagens, hospedagens e inscrições para congressos e cursos, provenientes da indústria farmacêutica, de órteses, próteses, equipamentos e implantes, brasileiras ou estrangeiras: - Bayer: finerinona; Novo Nordisk: Semaglutida; AstraZeneca: ciclosilicato de Zirconio, dapagliflozina; Amgen: evolocumabe; Boehringer Ingelheimer: empagliflozina; Lilly: tirzepatida, viatris atorvastatina; Daichii Sankyo: ácido bempedoico/edoxabana.
Jose Rocha Faria Neto	Declaração financeira A - Pagamento de qualquer espécie e desde que economicamente apreciáveis, feitos a (i) você, (ii) ao seu cônjuge/ companheiro ou a qualquer outro membro que resida com você, (iii) a qualquer pessoa jurídica em que qualquer destes seja controlador, sócio, acionista ou participante, de forma direta ou indireta, recebimento por palestras, aulas, atuação como proctor de treinamentos, remunerações, honorários pagos por participações em conselhos consultivos, de investigadores, ou outros comitês, etc. Provenientes da indústria farmacêutica, de órteses, próteses, equipamentos e implantes, brasileiras ou estrangeiras: - Aché: DAC e dislipidemia; Daiichi Sankyo: DAC e dislipidemia; Libbs: DAC e dislipidemia; Novartis: dislipidemia; AstraZeneca: diabetes; Lilly: diabetes e obesidade; Novo Nordisk: diabetes e obesidade; Sanofi e Medley: dislipidemia; Bayer: risco cardiovascular. Outros relacionamentos Financiamento de atividades de educação médica continuada, incluindo viagens, hospedagens e inscrições para congressos e cursos, provenientes da indústria farmacêutica, de órteses, próteses, equipamentos e implantes, brasileiras ou estrangeiras: - Novo Nordisk; Bayer; Daiichi Sankyo; AstraZeneca.
Kleisson Antônio Pontes Maia	Declaração financeira A - Pagamento de qualquer espécie e desde que economicamente apreciáveis, feitos a (i) você, (ii) ao seu cônjuge/ companheiro ou a qualquer outro membro que resida com você, (iii) a qualquer pessoa jurídica em que qualquer destes seja controlador, sócio, acionista ou participante, de forma direta ou indireta, recebimento por palestras, aulas, atuação como proctor de treinamentos, remunerações, honorários pagos por participações em conselhos consultivos, de investigadores, ou outros comitês, etc. Provenientes da indústria farmacêutica, de órteses, próteses, equipamentos e implantes, brasileiras ou estrangeiras: - Novartis: hipercolesterolemia; GSK: vacinas; Biolab: hipertensão, Lilly: diabetes, obesidade; Novo Nordisk: diabetes, obesidade; Servier: doença coronária. B - Financiamento de pesquisas sob sua responsabilidade direta/pessoal (direcionado ao departamento ou instituição) provenientes da indústria farmacêutica, de órteses, próteses, equipamentos e implantes, brasileiras ou estrangeiras: - Lilly: Lp(a). Outros relacionamentos Financiamento de atividades de educação médica continuada, incluindo viagens, hospedagens e inscrições para congressos e cursos, provenientes da indústria farmacêutica, de órteses, próteses, equipamentos e implantes, brasileiras ou estrangeiras: - Servier: doença coronária; Lilly: diabetes; Novo Nordisk: diabetes, obesidade; Viatris: hipercolesterolemia.
Luiz Sérgio Fernandes de Carvalho	Nada a ser declarado
Marcelo Chiara Bertolami	Declaração financeira A - Pagamento de qualquer espécie e desde que economicamente apreciáveis, feitos a (i) você, (ii) ao seu cônjuge/ companheiro ou a qualquer outro membro que resida com você, (iii) a qualquer pessoa jurídica em que qualquer destes seja controlador, sócio, acionista ou participante, de forma direta ou indireta, recebimento por palestras, aulas, atuação como proctor de treinamentos, remunerações, honorários pagos por participações em conselhos consultivos, de investigadores, ou outros comitês, etc. Provenientes da indústria farmacêutica, de órteses, próteses, equipamentos e implantes, brasileiras ou estrangeiras: - Abbott: Lipidil.
Marcelo Heitor Vieira Assad	Declaração financeira A - Pagamento de qualquer espécie e desde que economicamente apreciáveis, feitos a (i) você, (ii) ao seu cônjuge/ companheiro ou a qualquer outro membro que resida com você, (iii) a qualquer pessoa jurídica em que qualquer destes seja controlador, sócio, acionista ou participante, de forma direta ou indireta, recebimento por palestras, aulas, atuação como proctor de treinamentos, remunerações, honorários pagos por participações em conselhos consultivos, de investigadores, ou outros comitês, etc. Provenientes da indústria farmacêutica, de órteses, próteses, equipamentos e implantes, brasileiras ou estrangeiras: - AstraZeneca: Forxiga; BAYER: Firialta; Biolab: Repath; Boerhringer Ingelheim: Glyxambi; Daiichy Sankyo: Benicar, Nustendi; EMS: Bramicar; GSK: Shingrix; Libbs: Stanglit; Lilly: Mounjaro; Novo Nordisk: Wegovy; Novartis: Sybrava; Pfizer: Prevenar 20; Viatris: Lipitor, Inspra. B - Financiamento de pesquisas sob sua responsabilidade direta/pessoal (direcionado ao departamento ou instituição) provenientes da indústria farmacêutica, de órteses, próteses, equipamentos e implantes, brasileiras ou estrangeiras: - AMGEN: Olpasirana. Outros relacionamentos Financiamento de atividades de educação médica continuada, incluindo viagens, hospedagens e inscrições para congressos e cursos, provenientes da indústria farmacêutica, de órteses, próteses, equipamentos e implantes, brasileiras ou estrangeiras: - Bayer: Firialta; Daiichi Sankyo: Benicar; Novo Nordisk: Wegovy.
Marcio Hiroshi Miname	Declaração financeira A - Pagamento de qualquer espécie e desde que economicamente apreciáveis, feitos a (i) você, (ii) ao seu cônjuge/ companheiro ou a qualquer outro membro que resida com você, (iii) a qualquer pessoa jurídica em que qualquer destes seja controlador, sócio, acionista ou participante, de forma direta ou indireta, recebimento por palestras, aulas, atuação como proctor de treinamentos, remunerações, honorários pagos por participações em conselhos consultivos, de investigadores, ou outros comitês, etc. Provenientes da indústria farmacêutica, de órteses, próteses, equipamentos e implantes, brasileiras ou estrangeiras: - Novartis: dislipidemia; Ache: dislipidemia; Libbs: dislipidemia B - Financiamento de pesquisas sob sua responsabilidade direta/pessoal (direcionado ao departamento ou instituição) provenientes da indústria farmacêutica, de órteses, próteses, equipamentos e implantes, brasileiras ou estrangeiras: - Kowa: Pemafibrato.
Maria Cristina de Oliveira Izar	Declaração financeira A - Pagamento de qualquer espécie e desde que economicamente apreciáveis, feitos a (i) você, (ii) ao seu cônjuge/ companheiro ou a qualquer outro membro que resida com você, (iii) a qualquer pessoa jurídica em que qualquer destes seja controlador, sócio, acionista ou participante, de forma direta ou indireta, recebimento por palestras, aulas, atuação como proctor de treinamentos, remunerações, honorários pagos por participações em conselhos consultivos, de investigadores, ou outros comitês, etc. Provenientes da indústria farmacêutica, de órteses, próteses, equipamentos e implantes, brasileiras ou estrangeiras: - Amgen: Repatha; Amryt Pharma: Lojuxta; AstraZeneca: Dapagliflozina; Aché: Trezor, Trezete; Biolab: Livalo, Posicor, Repatha; Abbott: Lipidil; EMS: Rosuvastatina; Eurofarma: Rosuvastatina; Sanofi: Praluent, Zympass, Zympass Eze, Efluelda; Libbs: Plenance, Plenance Eze; NovoNordisk: Ozempic; Servier: Acertamlo, Acertalix; PTCBio: Waylivra; Ultragenyx: Evkeeza; Alnylam: AMVUTTRA; GSK: Shingrix, Arexvy. B - Financiamento de pesquisas sob sua responsabilidade direta/pessoal (direcionado ao departamento ou instituição) provenientes da indústria farmacêutica, de órteses, próteses, equipamentos e implantes, brasileiras ou estrangeiras: - PTCBio: Waylivra; Amgen: Repatha; Novartis: Inclisiran, Pelacarsen; NovoNordisk: Ziltivekimab. Outros relacionamentos Financiamento de atividades de educação médica continuada, incluindo viagens, hospedagens e inscrições para congressos e cursos, provenientes da indústria farmacêutica, de órteses, próteses, equipamentos e implantes, brasileiras ou estrangeiras: - Novo Nordisk: Diabetes, Ziltivekimabe; GSK: vacinas. Possui qualquer outro interesse (financeiro ou a qualquer outro título) que deva ser declarado tendo em vista o cargo ocupado na SBC, ainda que não expressamente elencado anteriormente: - Membro do Comitê Gestor da Rede Hipertri Brasil.
Maurício Alves Barreto	Declaração financeira A - Pagamento de qualquer espécie e desde que economicamente apreciáveis, feitos a (i) você, (ii) ao seu cônjuge/ companheiro ou a qualquer outro membro que resida com você, (iii) a qualquer pessoa jurídica em que qualquer destes seja controlador, sócio, acionista ou participante, de forma direta ou indireta, recebimento por palestras, aulas, atuação como proctor de treinamentos, remunerações, honorários pagos por participações em conselhos consultivos, de investigadores, ou outros comitês, etc. Provenientes da indústria farmacêutica, de órteses, próteses, equipamentos e implantes, brasileiras ou estrangeiras: - Aulas para a indústria: Novo Nordisk: Rybelsus; DM2 e Wegovy: obesidade; Novartis: Sybrava, dislipidemia; Biolab: Repatha, dislipidemia; Libbs: Plenance Eze, dislipidemia; Merck: Contrave, obesidade; escrita de material científico: Aché, dislipidemia; pesquisa clínica: investigador principal em estudos patrocinados pela Amgen.(OCEAN Outcomes). Arrowhead pharmaceuticals (SHASTA-3). Outros relacionamentos Financiamento de atividades de educação médica continuada, incluindo viagens, hospedagens e inscrições para congressos e cursos, provenientes da indústria farmacêutica, de órteses, próteses, equipamentos e implantes, brasileiras ou estrangeiras: - Novo Nordisk: Rybelsus, DM2.
Natasha Slhessarenko Fraife Barreto	Nada a ser declarado
Paulo Eduardo Ballvé Behr	Declaração financeira A - Pagamento de qualquer espécie e desde que economicamente apreciáveis, feitos a (i) você, (ii) ao seu cônjuge/ companheiro ou a qualquer outro membro que resida com você, (iii) a qualquer pessoa jurídica em que qualquer destes seja controlador, sócio, acionista ou participante, de forma direta ou indireta, recebimento por palestras, aulas, atuação como proctor de treinamentos, remunerações, honorários pagos por participações em conselhos consultivos, de investigadores, ou outros comitês, etc. Provenientes da indústria farmacêutica, de órteses, próteses, equipamentos e implantes, brasileiras ou estrangeiras: - Novartis: Sybrava; Novartis: Cosentyx; Biolab: Livalo; Aché: Trezete; PTC: Volanesorsena; Libbs: Zinpass; Novo Nordisk: Ozempic; Daiichi Sankyo: Nustendi; Amgen: Olpasiran. Outros relacionamentos Financiamento de atividades de educação médica continuada, incluindo viagens, hospedagens e inscrições para congressos e cursos, provenientes da indústria farmacêutica, de órteses, próteses, equipamentos e implantes, brasileiras ou estrangeiras: - Novartis: Sybrava; Daiichi: Nustendi; Novo Nordisk: Ozempic.
Pedro Gabriel Melo de Barros e Silva	Nada a ser declarado
Pedro Pimentel Filho	Declaração financeira A - Pagamento de qualquer espécie e desde que economicamente apreciáveis, feitos a (i) você, (ii) ao seu cônjuge/ companheiro ou a qualquer outro membro que resida com você, (iii) a qualquer pessoa jurídica em que qualquer destes seja controlador, sócio, acionista ou participante, de forma direta ou indireta, recebimento por palestras, aulas, atuação como proctor de treinamentos, remunerações, honorários pagos por participações em conselhos consultivos, de investigadores, ou outros comitês, etc. Provenientes da indústria farmacêutica, de órteses, próteses, equipamentos e implantes, brasileiras ou estrangeiras: - Pesquisa Clínica em Cardiologia com participação em estudos das empresas como Amgen, Bayer, AstraZeneca, Janssen, Lilly. B - Financiamento de pesquisas sob sua responsabilidade direta/pessoal (direcionado ao departamento ou instituição) provenientes da indústria farmacêutica, de órteses, próteses, equipamentos e implantes, brasileiras ou estrangeiras: - Amge : Evolocumabe; AstraZeneca: Forxiga; MSD: MK 606 Bayer: Finerinona; Janssen: Milvexiana; todos da área cardiovascular. Outros relacionamentos Financiamento de atividades de educação médica continuada, incluindo viagens, hospedagens e inscrições para congressos e cursos, provenientes da indústria farmacêutica, de órteses, próteses, equipamentos e implantes, brasileiras ou estrangeiras: - Daichii Sankyo : ácido bempedoico.
Raul Cavalcante Maranhão	Nada a ser declarado
Raul Dias dos Santos Filho	Declaração financeira A - Pagamento de qualquer espécie e desde que economicamente apreciáveis, feitos a (i) você, (ii) ao seu cônjuge/ companheiro ou a qualquer outro membro que resida com você, (iii) a qualquer pessoa jurídica em que qualquer destes seja controlador, sócio, acionista ou participante, de forma direta ou indireta, recebimento por palestras, aulas, atuação como proctor de treinamentos, remunerações, honorários pagos por participações em conselhos consultivos, de investigadores, ou outros comitês, etc. Provenientes da indústria farmacêutica, de órteses, próteses, equipamentos e implantes, brasileiras ou estrangeiras: - Amgen; Novartis;, Arrowhead; Ionis; Torrent, Sanofi; Daiichi Sankyo;Aché: hipolipemiantes; Novo Nordisk, Eli-Lilly: hipoglicemiantes. B - Financiamento de pesquisas sob sua responsabilidade direta/pessoal (direcionado ao departamento ou instituição) provenientes da indústria farmacêutica, de órteses, próteses, equipamentos e implantes, brasileiras ou estrangeiras: - Amgen; Arrowhead, Ionis; Eli-Lilly: hipolipemiantes.
Renato Jorge Alves	Declaração financeira A - Pagamento de qualquer espécie e desde que economicamente apreciáveis, feitos a (i) você, (ii) ao seu cônjuge/ companheiro ou a qualquer outro membro que resida com você, (iii) a qualquer pessoa jurídica em que qualquer destes seja controlador, sócio, acionista ou participante, de forma direta ou indireta, recebimento por palestras, aulas, atuação como proctor de treinamentos, remunerações, honorários pagos por participações em conselhos consultivos, de investigadores, ou outros comitês, etc. Provenientes da indústria farmacêutica, de órteses, próteses, equipamentos e implantes, brasileiras ou estrangeiras: - Novartis: Inclisirana; Viatris: Sertralina; Aché: Trezet; Mantecorp: Coledue R; Servier: Acertil; Libbs: Plenance; GSK: Vacinas; EMS: Valsartana. Outros relacionamentos Financiamento de atividades de educação médica continuada, incluindo viagens, hospedagens e inscrições para congressos e cursos, provenientes da indústria farmacêutica, de órteses, próteses, equipamentos e implantes, brasileiras ou estrangeiras: - Novartis: Inclisirana; Mantecorp: Coledue R.
Sergio Emanuel Kaiser	Declaração financeira A - Pagamento de qualquer espécie e desde que economicamente apreciáveis, feitos a (i) você, (ii) ao seu cônjuge/ companheiro ou a qualquer outro membro que resida com você, (iii) a qualquer pessoa jurídica em que qualquer destes seja controlador, sócio, acionista ou participante, de forma direta ou indireta, recebimento por palestras, aulas, atuação como proctor de treinamentos, remunerações, honorários pagos por participações em conselhos consultivos, de investigadores, ou outros comitês, etc. Provenientes da indústria farmacêutica, de órteses, próteses, equipamentos e implantes, brasileiras ou estrangeiras: - Bayer: vericiguat e Firialta; Daiichi Sankyo: Nustendi, Lixiana e Benicar; Novo Nordisk: Wegovy; Biolab: Repatha; Libbs:, Naprix; Astrazeneca: Forxiga e Selozok - Novartis, Sybrava, Boehringer Ingelheim: Jardiance; Farmoquimica: Rosuvastatina. Outros relacionamentos Financiamento de atividades de educação médica continuada, incluindo viagens, hospedagens e inscrições para congressos e cursos, provenientes da indústria farmacêutica, de órteses, próteses, equipamentos e implantes, brasileiras ou estrangeiras: - Novo Nordisk: Wegovy; Daiichi Sanlyo: Nustendi; Astrazeneca: insuficiência cardíaca; Bayer: Firialta; Novartis: Sybrava.
Valéria Arruda Machado	Declaração financeira A - Pagamento de qualquer espécie e desde que economicamente apreciáveis, feitos a (i) você, (ii) ao seu cônjuge/ companheiro ou a qualquer outro membro que resida com você, (iii) a qualquer pessoa jurídica em que qualquer destes seja controlador, sócio, acionista ou participante, de forma direta ou indireta, recebimento por palestras, aulas, atuação como proctor de treinamentos, remunerações, honorários pagos por participações em conselhos consultivos, de investigadores, ou outros comitês, etc. Provenientes da indústria farmacêutica, de órteses, próteses, equipamentos e implantes, brasileiras ou estrangeiras: - Abbott: nutrição.
Viviane Zorzanelli Rocha	Declaração financeira A - Pagamento de qualquer espécie e desde que economicamente apreciáveis, feitos a (i) você, (ii) ao seu cônjuge/ companheiro ou a qualquer outro membro que resida com você, (iii) a qualquer pessoa jurídica em que qualquer destes seja controlador, sócio, acionista ou participante, de forma direta ou indireta, recebimento por palestras, aulas, atuação como proctor de treinamentos, remunerações, honorários pagos por participações em conselhos consultivos, de investigadores, ou outros comitês, etc. Provenientes da indústria farmacêutica, de órteses, próteses, equipamentos e implantes, brasileiras ou estrangeiras: - Abbott: Lipidil; Aché: Trezete; Biolab: Repatha; Daichii-Sankyo: Nustendi; Lilly: Mounjaro; Novartis: Sybrava; Novo-Nordisk: Wegovy; Ultragenyx: Evkeeza. Outros relacionamentos Financiamento de atividades de educação médica continuada, incluindo viagens, hospedagens e inscrições para congressos e cursos, provenientes da indústria farmacêutica, de órteses, próteses, equipamentos e implantes, brasileiras ou estrangeiras: - Novartis: Sybrava; Novo Nordisk: Wegovy.

## Sumário

**Preâmbulo** 13
**1. Introdução** 15
**1.1. A perspectiva populacional brasileira** 15
**1.2. Foco ampliado: da dislipidemia à prevenção da aterosclerose** 15
**1.2.1. Fase precoce – infância e adolescência** 15
**1.2.2. Fase intermediária – adultos jovens à meia-idade** 15
**1.2.3. Fase tardia – idosos e pacientes com doença clínica estabelecida** 15
**1.3. Um modelo estratificado de prevenção da doença cardiovascular aterosclerótica** 15
**1.4. Pontos-chave da Diretriz de Dislipidemias e Prevenção da Aterosclerose de 2025** 16
**1.5. Força da Recomendação e Certeza da Evidência** 16
**1.6. Sumário de recomendações** 17
**2. Epidemiologia** 27
**2.1. Níveis Médios de Lípides Plasmáticos e Prevalências das Dislipidemias** 27
**2.2. Mortalidade Cardiovascular Atribuível ao LDL-colesterol Elevado** 29
**2.3. Dados sobre Tratamento e Metas Atingidas** 29
**3. Diagnóstico** 31
**3.1. Avaliação Laboratorial dos Parâmetros Lipídicos e das Apolipoproteínas** 31
**3.1.1. Fases Pré-Analítica e Analítica** 31
**3.1.1.1. Fase Pré-Analítica** 31
**3.1.1.2. Fase Analítica** 32
**3.1.1.2.1. Métodos Restritos à Pesquisa** 32
**3.1.1.2.2. Métodos Convencionais – Rotina Laboratorial** 32
**3.1.1.2.2.1. Métodos Enzimáticos Colorimétricos** 32
**3.1.1.2.2.2. Point of Care Testing** 33
**3.1.1.2.2.3. Cálculo do Colesterol da Lipoproteína de Baixa Densidade** 33
**3.1.1.2.2.4. Medida do Colesterol Não HDL (não-HDL-c)** 33
**3.1.1.2.2.5. Medida da Apolipoproteína B (ApoB)** 34
**3.1.1.2.2.6. Lipoproteína(a)** 34
**3.1.1.2.2.7. Valores Referenciais do Perfil Lipídico** 36
**3.2. Diagnóstico Genético das Dislipidemias** 36
**3.2.1. Hipercolesterolemias de Base Genética** 36
**3.2.1.1. Considerações para Solicitar um Teste Genético** 36
**3.3. Diagnóstico das Hipertrigliceridemias** 38
**3.3.1. Síndrome da Quilomicronemia Familiar** 38
**3.3.1.1. Definição** 38
**3.3.1.2. Diagnóstico Clínico e Laboratorial da Quilomicronemia Familiar** 39
**3.3.1.3. Escores Diagnósticos** 39
**3.3.1.4. Diagnóstico Diferencial** 39
**3.3.1.5. Diagnóstico Genético** 39
**3.3.1.6. Atividade da Lipoproteína Lipase** 39
**3.3.1.7. Outros Exames Diagnósticos** 39
**4. Estratificação de Risco** 40
**4.1. Estratificação do Risco Cardiovascular** 40
**4.2. Escores de Risco Cardiovascular** 42
**4.3. Fatores Agravantes do Risco Cardiovascular** 42
**4.3.1. História Familiar de Doença Cardiovascular Prematura** 42
**4.3.2. Adiposidade e suas Manifestações** 42
**4.3.3. Condições Inflamatórias Crônicas** 43
**4.3.4. Transplante de Órgãos** 43
**4.3.5. Fatores Agravantes de Risco Específicos das Mulheres** 44
**4.3.5.1. Idade da Menarca** 44
**4.3.5.2. Distúrbios Durante a Gestação e Parto Prematuro** 44
**4.3.5.3. Abortos de Repetição** 45
**4.3.5.4. Menopausa Precoce** 45
**4.4. Exames Complementares** 45
**4.4.1. Lipoproteína(a)** 45
**4.4.2. Proteína C-Reativa Ultrassensível** 45
**4.4.3. Troponinas Cardíacas de Alta Sensibilidade** 45
**4.4.4. Peptídeo Natriurético Tipo B e Peptídeo Natriurético Tipo B N-Terminal NT-proBNP** 46
**4.5. Marcadores de Doença Aterosclerótica Subclínica** 46
**4.5.1. Escore de Cálcio Coronário** 46
**4.5.2. Ultrassom das Artérias Carótidas** 48
**4.6. Estratificação do Risco Cardiovascular no Diabetes *Mellitus*** 48
**4.7. As Categorias do Risco Cardiovascular Aterosclerótico** 49
**4.8. Particularidades da Estratificação do Risco Cardiovascular em Idosos** 50
**4.9. Particularidades da Estratificação do Risco Cardiovascular em Adultos Jovens** 50
**4.10. Estratificação do Risco Cardiovascular na Infância e Adolescência** 50
**5. Metas de Tratamento** 52
**5.1. Meta Primária e Coprimária: Colesterol de Lipoproteína de Baixa Densidade e Colesterol Não Associado à Lipoproteína de Alta Densidade** 52
**5.2. Recomendações de Metas de acordo com a Estratificação de Risco Cardiovascular** 52
**5.2.1. Indivíduos de Risco Baixo** 52
**5.2.2. Indivíduos de Risco Intermediário** 53
**5.2.3. Indivíduos de Risco Alto** 53
**5.2.4. Indivíduos de Risco Muito Alto** 53
**5.2.5. Indivíduos de Risco Cardiovascular Extremo** 54
**5.3. Apolipoproteína B** 54
**5.4. Colesterol da Lipoproteína de Alta Densidade (HDL-c)** 54
**5.5. Triglicérides** 55
**5.6. Lipoproteína(a)** 55
**6. Tratamento Não Farmacológico** 56
**6.1. Recomendações de Estilo de Vida para Melhorar o Perfil Lipídico** 56
**6.1.1. Aspectos Nutricionais** 56
**6.1.2. Carboidratos** 56
**6.1.3. Gorduras** 56
**6.1.4. Fibras Solúveis** 56
**6.2. Cessação do Tabagismo** 57
**6.3. Controle de Peso** 57
**6.4. Espiritualidade** 57
**6.5. Prática de Atividades Físicas** 58
**6.6. Ingesta Alcoólica** 58
**6.7. Suplementos Dietéticos e Alimentos Funcionais em Dislipidemias** 58
**7. Tratamento Farmacológico** 59
**7.1. Estatinas** 59
**7.2. Ezetimiba** 61
**7.3. Novas Abordagens que Têm como Alvo o RNA Mensageiro** 61
**7.4. Terapia anti-PCSK9** 61
**7.5. Ácido Bempedoico** 62
**7.6. Inibidores da Proteína de Transferência de Ésteres de Colesterol e Terapias para Aumentar o Colesterol HDL** 62
**7.7. Fibratos** 62
**7.8. Ômega-3** 63
**7.9. Inibidores da Apolipoproteína C-III** 63
**7.10. Inibidores da Proteína Angiopoietina-like 3** 64
**7.11. Inibidores da Lipoproteína(a)** 64
**7.12. CRISPR e Terapias Gênicas** 64
**7.13. Terapia Combinada** 65
**7.13.1. Benefícios da Terapêutica Combinada** 65
**7.13.2. Combinação de Estatina e Ezetimiba** 66
**7.13.3. Combinação de Estatina e Terapia Direcionada para PCSK9** 66
**7.13.4. Combinação de Ezetimiba e Ácido Bempedoico** 66
**7.13.5. Combinação de Estatina, Ezetimiba e Terapia Direcionada para PCSK9** 67
**7.13.6. Combinação de Estatina, Ezetimiba e Ácido Bempedoico** 67
**8. Manejo da Intolerância à Estatina** 67
**8.1. Definição** 67
**8.2. Prevalência** 67
**8.3. Diagnóstico** 68
**8.4. Efeito Nocebo** 68
**8.5. Sintomas Musculares** 68
**8.5.1. Características Clínicas, Classificação e Manejo dos Sintomas Musculares Relacionados às Estatinas** 68
**8.5.2. Sintomas Musculares Toleráveis e Intoleráveis** 68
**8.5.3. Elevação da CK** 68
**8.5.4. Rabdomiólise** 69
**8.5.5. Miopatia Necrotizante Autoimune por Estatinas** 70
**8.6. Causas Associadas à Intolerância às Estatinas** 70
**8.7. Manejo do Paciente com Intolerância às Estatinas** 70
**8.7.1. Interrupção e Reinício do Uso da Estatina** 74
**8.7.2. Utilização de Produtos que não têm Comprovação de Benefícios** 74
**8.7.3. Interações medicamentosas das estatinas** 75
**8.7.3.1. Anticoagulantes** 75
**8.7.3.2. Antifúngicos Azólicos** 75
**8.7.3.3. Agentes Antirretrovirais** 75
**8.7.3.4. Bloqueadores dos Canais de Cálcio** 76
**8.7.3.5. Agentes Antiarrítmicos** 76
**8.7.3.6. Imunossupressores** 76
**8.7.3.7. Macrolídeos** 76
**8.7.3.8. Interações entre Agentes Hipolipemiantes** 76
**9. Dislipidemia em Populações Específicas: Considerações para o Manejo Clínico** 77
**9.1. Insuficiência Cardíaca** 77
**9.2. Pessoas vivendo com o HIV** 78
**9.3. Diabetes** 78
**9.3.1. Caraterísticas Específicas da Dislipidemia na Resistência à Insulina e no Diabetes Mellitus de Tipo 2** 79
**9.3.2. Tratamento para Dislipidemia em Pacientes Diabéticos** 79
**9.3.3. Tratamento Medicamentoso** 79
**9.4. Hipotireoidismo** 81
**9.5. Doença Renal Crônica** 81
**9.6. Obesidade** 82
**9.7. Idosos** 83
**9.8. Tratamento Não Farmacológico** 83
**9.9. Tratamento Farmacológico** 84
**9.10. Crianças** 84
**9.10.1. Perfil Lipídico na Infância** 84
**9.10.2. Rastreamento** 84
**9.10.3. Dislipidemias Primárias** 84
**9.10.4. Hipercolesterolemia Familiar Homozigótica** 85
**9.10.5. Hipertrigliceridemias** 85
**9.10.6. Hipertrigliceridemia Monogênica (Hipertrigliceridemias Graves)** 85
**9.10.7. Dislipidemias Secundárias** 85
**9.10.8. Indicação do Uso de Estatinas de acordo com o Risco nas Dislipidemias Secundárias, Condições de Alto Risco ou Presença de Fatores de Risco (Valores de Corte para Iniciar Tratamento)** 85
**9.11. Transplantados** 86
**9.12. Doenças Hepáticas Crônicas** 88
**9.12.1. Esteatose Hepática Associada à Disfunção Metabólica** 88
**9.12.1.1. Definição** 88
**9.12.1.2. Prevalência e Risco Cardiovascular** 88
**9.12.1.3. Redução do Risco Cardiovascular** 88
**9.12.1.4. Desfechos Hepáticos** 88
**9.12.1.5. Segurança** 88
**9.12.2. Colestases Intra-Hepáticas** 88
**9.12.3. Cirrose Hepática** 88
**9.12.4. Carcinoma Hepatocelular** 88
**9.13. Síndrome Coronária Aguda** 89
**9.14. Doenças Imunomediadas** 90
**9.15. Gestação** 91
**9.15.1. Dislipidemia da Gestação em Mulheres Normolipidêmicas** 91
**9.15.2. Dislipidemia da Gestação em Mulheres Dislipidêmicas** 91
**9.15.3. Lipoproteína(a)** 91
**9.15.4. Tratamento Medicamentoso** 91
**9.15.4.1. Estatinas** 91
**9.15.4.2. Novas Evidências** 91
**9.15.4.3. Resinas ou Sequestradoras de Sais Biliares** 92
**9.15.4.4. Aférese de Lipoproteínas** 92
**9.15.4.5. Ezetimiba** 92
**9.15.4.6. Ômega-3** 92
**9.16. Mulher** 92
**10. Conclusão** 93
**Referências** 94

## Preâmbulo

A doença cardiovascular aterosclerótica (DCVA) representa a principal causa de morte em todo o mundo, apesar dos grandes avanços na compreensão da fisiopatologia da aterosclerose, suas consequências e o desenvolvimento de novas terapias preventivas.^1 , 2^ O aumento da obesidade e do diabetes em todo o mundo resulta frequentemente em dislipidemia, caracterizada por uma queda nos níveis de colesterol da lipoproteína de alta densidade (HDL-c) e um aumento nos níveis de colesterol não HDL (não-HDL-c) e altos níveis de triglicérides.^3 , 4^ É preocupante o fato de a DCVA estar aumentando em prevalência tanto em países de baixa e média renda, onde reside a maioria da população global, quanto se manifestando nesses países cerca de uma década ou mais antes do que em países de alta renda.^5^

A relevância das diretrizes clínicas na prática médica tem sido amplamente demonstrada, evidenciando seu papel fundamental na orientação dos profissionais de saúde para o manejo adequado das diversas condições clínicas. Ao fornecer recomendações baseadas em evidências, as diretrizes contribuem para a padronização das condutas, promovem melhores desfechos clínicos e auxiliam na tomada de decisões terapêuticas mais eficazes. Na realidade, seguir as diretrizes melhora o prognóstico dos nossos pacientes, reforçando sua importância como ferramenta essencial na prática assistencial. Além disso, elas fornecem uma matriz que pode contribuir para o desenho de políticas de saúde pública, baseadas em evidências científicas convincentes, identificam os obstáculos à implementação das melhores práticas e, em última análise, oferecem soluções potenciais a serem adaptadas em diferentes regiões. A presente Diretriz da Sociedade Brasileira de Cardiologia sobre o manejo do colesterol e prevenção de aterosclerose é um bom exemplo disso, constituindo uma ferramenta que deverá ser de grande interesse para a classe médica que lida com essas situações, bem como para os decisores políticos, a fim de que sejam tomadas as medidas necessárias para reduzir o impacto das alterações do metabolismo lipídico nas populações.

Hoje em dia, está bem demonstrado o papel dos níveis elevados de colesterol no sangue para a carga global da doença aterosclerótica. A redução dos níveis de colesterol da lipoproteína de baixa densidade (LDL-c) tem se mostrado amplamente benéfica ao longo do contínuo do risco cardiovascular. Indivíduos com maior probabilidade de eventos aterotrombóticos — como infarto do miocárdio, acidente vascular cerebral (AVC), revascularização ou morte cardiovascular — são aqueles que mais se beneficiam, alcançando reduções absolutas de risco mais expressivas com a diminuição do LDL-c. A importância do momento do início do tratamento e da sua duração no curso da doença aterosclerótica deve ser devidamente ressaltada, orientando de forma clara as políticas de saúde e, sobretudo, sua implementação. Além disso, a maioria dos pacientes, globalmente, não alcança uma redução adequada do LDL-c necessária para minimizar seu risco individual de DCVA. Isso se deve, em grande parte, ao uso limitado de estatinas potentes, em doses eficazes, como terapia de primeira linha, à baixa utilização de estratégias combinadas e à adesão insuficiente a regimes hipolipemiantes – fatores que contribuem para uma exposição cumulativa ao colesterol ainda elevada. Ressalta-se, portanto, cada vez mais o papel das combinações terapêuticas, inclusive desde fases muito precoces, visando a obtenção de melhores resultados e maior impacto na redução da carga aterosclerótica.^6^

Está também demonstrado que a redução do LDL-c é benéfica não apenas para aqueles com níveis elevados de colesterol, mas também para aqueles com maior risco das principais manifestações clínicas da aterosclerose.^7^ A redução do LDL-c deve, assim, ser considerada em todos os indivíduos com maior risco.^8^ identificados como tal pela presença de: i) manifestação anterior de DCVA; ii) presença de condição de alto risco, como diabetes, doença renal crônica, uso de tabaco ou hipertensão, que aumentam o risco de DCVA mesmo na ausência de alterações lipídicas concomitantes; iii) elevações extremas do LDL-c que têm uma base genética, como hipercolesterolemia familiar (HF); iv) com alto risco cardiovascular devido aos efeitos combinados de múltiplos fatores de risco; v) elevações isoladas em lipoproteínas aterogênicas, incluindo lipoproteínas ricas em triglicérides (comumente chamadas de dislipidemia aterogênica) ou elevações em lipoproteína(a) (Lp(a)); vi) carga elevada de aterosclerose coronariana subclínica.^9^

O tratamento e controle das dislipidemias representam um dos grandes desafios médicos da atualidade. Diretrizes atuais e bem desenhadas, como esta, constituem ferramenta fundamental para atingir o objetivo de reduzir o impacto das doenças cardiovasculares (DCV) e, neste caso em particular, da doença aterosclerótica.

**Fausto J Pinto**

## 1. Introdução

A DCVA continua sendo a principal causa de morte no mundo, apesar dos avanços significativos na compreensão de sua fisiopatologia e no desenvolvimento de terapias preventivas.^10^ No Brasil, essa realidade se repete: a DCVA representa a condição de maior impacto em morbimortalidade, afetando milhões de pessoas e sobrecarregando o sistema de saúde.^11^

Preocupa especialmente o aumento da prevalência da DCVA em países de baixa e média renda, como o Brasil, onde a maioria da população global reside.^3^ Nesses contextos, a doença frequentemente se manifesta ao menos uma década mais cedo do que em países de alta renda, comprometendo não apenas a saúde individual, mas também o potencial produtivo e econômico durante os anos mais ativos da vida. O impacto se estende ainda aos familiares e cuidadores, ampliando o peso social da doença.

A aterosclerose é uma doença crônica, silenciosa e progressiva, que se inicia ainda na infância. Estudos histopatológicos demonstram que alterações iniciais na parede arterial, como as estrias gordurosas, podem surgir já na primeira década de vida. Por isso, é fundamental compreender a DCVA não como uma doença exclusiva do adulto ou do idoso, mas como um processo contínuo, com múltiplas oportunidades de intervenção e prevenção ao longo da vida.^12 , 13^

Diante desse cenário, diretrizes atualizadas e adaptadas à realidade brasileira são essenciais para orientar estratégias de prevenção, diagnóstico precoce e tratamento eficaz, com foco na redução do risco cardiovascular em todas as fases da vida ( Figura Central ).

**Figure:**
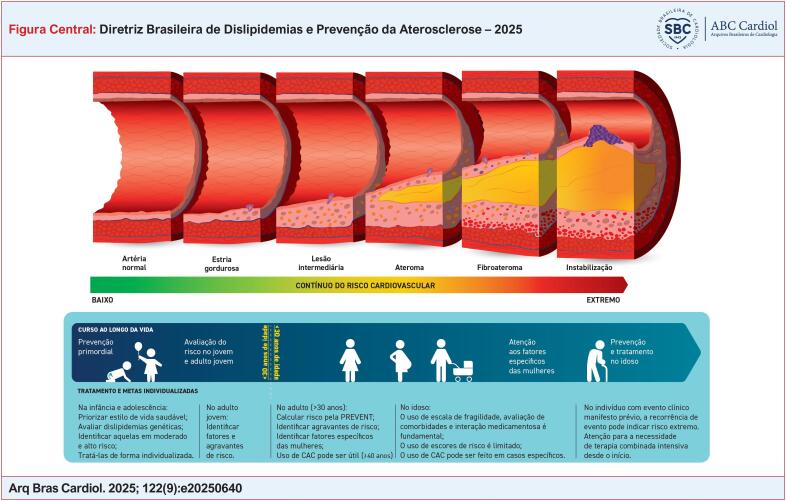


### 1.1. A perspectiva populacional brasileira

Com uma população estimada em aproximadamente 218,56 milhões de habitantes em 2025, o Brasil apresenta uma distribuição etária que impõe desafios específicos à prevenção cardiovascular. Estima-se que:

- cerca de 25% da população (≈ 54 milhões) tenham menos de 20 anos;
- aproximadamente 54% (≈ 118 milhões) estejam entre 20 e 60 anos;
- e 21% (≈ 45 milhões) tenham mais de 60 anos.

Essa distribuição reforça a necessidade de estratégias preventivas adaptadas ao estágio de vida e ao grau de progressão da aterosclerose, com foco em intervenções precoces, contínuas e direcionadas.

### 1.2. Foco ampliado: da dislipidemia à prevenção da aterosclerose

Embora a dislipidemia seja um fator central no desenvolvimento da aterosclerose, esta diretriz propõe uma abordagem ampliada, cujo objetivo principal é a prevenção da própria DCVA. Segundo dados do estudo *Global Burden of Disease* (GBD), o LDL-c elevado e a hipertensão são os dois principais fatores de risco, responsáveis por mais mortes cardiovasculares no Brasil. Diante dessa relevância,^14^ adota-se um modelo de ação baseado em três fases do curso de vida, com intervenções específicas e graduadas.

#### 1.2.1. Fase precoce – infância e adolescência

- Prevenção primordial, com foco na promoção de hábitos saudáveis e na prevenção do surgimento de fatores de risco;
- Rastreamento de dislipidemias genéticas, como a HF, com início precoce de intervenções em crianças e adolescentes;
- Ausência de manifestações clínicas, mas possibilidade de alterações histopatológicas iniciais na parede arterial.

#### 1.2.2. Fase intermediária – adultos jovens à meia-idade

- Presença frequente de aterosclerose subclínica, detectável por métodos de imagem (por exemplo, ultrassonografia de carótidas, escore de cálcio coronariano [CAC]);
- Indicação de intervenção precoce e intensiva, com mudanças no estilo de vida e, quando necessário, terapia farmacológica;
- Objetivo: interromper a progressão da doença e reduzir o risco de eventos cardiovasculares maiores ao longo da vida.

#### 1.2.3. Fase tardia – idosos e pacientes com doença clínica estabelecida

- Presença de doença aterosclerótica manifesta, como infarto agudo do miocárdio, AVC ou doença arterial periférica;
- Necessidade de tratamento intensivo e metas terapêuticas agressivas, especialmente no controle da dislipidemia;
- Ênfase nos indivíduos com maior risco cardiovascular dentro do contínuo e na redução da recorrência de eventos cardiovasculares.

### 1.3. Um modelo estratificado de prevenção da doença cardiovascular aterosclerótica

A aterosclerose deve ser compreendida como uma condição crônica, progressiva e de início precoce, que exige uma abordagem preventiva ao longo de todo o curso de vida. Esta diretriz propõe um modelo de cuidado que transcende o controle isolado da dislipidemia, incorporando a estratificação de risco individualizada, o uso de biomarcadores emergentes, como Lp(a), apolipoproteína B (ApoB) e proteína C reativa ultrassensível (PCR-us), bem como ferramentas de imagem para detecção de aterosclerose subclínica, como o CAC, e a adoção de metas terapêuticas baseadas em evidências.

Ao reconhecer a heterogeneidade da população brasileira e a complexidade da DCVA, reforça-se a importância de políticas públicas integradas, educação em saúde e acesso equitativo a estratégias diagnósticas e terapêuticas eficazes.

### 1.4. Pontos-chave da Diretriz de Dislipidemias e Prevenção da Aterosclerose de 2025

A Diretriz de Dislipidemias e Prevenção da Aterosclerose de 2025 atualiza e expande os conceitos da versão de 2017, mantendo o foco na estratificação de risco, mas com uma abordagem mais refinada e personalizada. O enfoque está centrado no contínuo de risco cardiovascular, iniciando pela identificação precoce e precisa de fatores agravantes — como histórico familiar de doença cardiovascular precoce e obesidade, entre outros — e avançando para a estratificação progressiva até os níveis de risco muito alto e risco extremo, nos quais a abordagem terapêutica deve ser mais intensiva. Essa avaliação inclui não apenas os fatores clássicos, mas também biomarcadores, como Lp(a), ApoB e PCR-us, além de ferramentas de imagem para detecção de aterosclerose subclínica, como o CAC ( Quadro 1.1 ).

**Quadro 1.1:** 10 Principais mensagens da Diretriz Brasileira de Dislipidemias e Prevenção da Aterosclerose

Nº	Mensagem-chave	Descrição
**1**	Promoção do estilo de vida saudável ao longo da vida	A base da prevenção CV é a adoção precoce e sustentada de hábitos saudáveis, como uma dieta equilibrada e atividade física regular, facilitando o controle dos fatores de risco.
**2**	Dosagem única de Lp(a)	Recomenda-se a dosagem de Lp(a) uma vez na vida em todos os adultos, para identificação de risco residual elevado.
**3**	Não-HDL-c como meta coprimária	O não-HDL-c é reconhecido como meta coprimária junto ao LDL-c, especialmente em pacientes com hipertrigliceridemia.
**4**	ApoB como meta secundária	ApoB é recomendada como meta complementar, sobretudo quando LDL-c e não-HDL-c estão controlados.
**5**	Uso do escore PREVENT para estratificação de risco	O escore PREVENT é a ferramenta preferencial para estratificação de risco em adultos sem DCVA conhecida.
**6**	Consideração de agravantes para reclassificação de risco	Fatores como Lp(a) elevada, doenças inflamatórias crônicas e história familiar devem ser considerados para reclassificação e intensificação terapêutica.
**7**	Uso do escore de cálcio coronariano e imagem em casos selecionados	Em pacientes de risco intermediário, exames como o CAC podem refinar a decisão terapêutica.
**8**	Meta de LDL-c < 115 mg/dL para baixo risco	Considerar tratamento farmacológico se LDL-c persistentemente ≥ 145 mg/dL, mesmo em indivíduos de baixo risco.
**9**	Reconhecimento da categoria de risco cardiovascular extremo	Nova categoria com alvo de LDL-c < 40 mg/dL para pacientes com risco extremamente elevado.
**10**	Terapia combinada precoce como estratégia inicial	Combinação de estatina + ezetimiba ± anti-PCSK9 pode ser iniciada precocemente em pacientes de alto, muito alto ou extremo risco.

CV: cardiovascular; Não-HDL-c: colesterol não HDL; Lp(a): lipoproteína(a); ApoB: apolipoproteína B, PREVENT: Predicting Risk of Cardiovascular Disease Events; DCVA: doença cardiovascular aterosclerótica; CAC: escore de cálcio coronariano; PCSK9: proproteína convertase subtilisina/quexina tipo 9.

Com base nessa estratificação ampliada, as metas terapêuticas tornam-se mais intensivas: níveis de LDL-c < 50 mg/d para pacientes de muito alto risco e < 40 mg/dL para aqueles em risco extremo. A terapia combinada passa a ser recomendada como primeira linha de tratamento nesses grupos, com o uso de estatinas associadas à ezetimiba, terapias anti-PCSK9 e ácido bempedoico, especialmente em casos de intolerância às estatinas ou necessidade de redução adicional do risco cardiovascular (CV).

Outro avanço importante é o manejo estruturado da intolerância às estatinas, frequentemente subestimada na prática clínica. A diretriz propõe algoritmos claros para diagnóstico e alternativas terapêuticas eficazes.

Por fim, reconhecendo os desafios específicos do Brasil – como baixa adesão ao tratamento e dificuldade em atingir metas terapêuticas – esta diretriz busca não apenas atualizar o conhecimento técnico, mas transformar a prática clínica, promovendo melhores desfechos e redução da carga da doença cardiovascular no país.

### 1.5. Força da Recomendação e Certeza da Evidência

As recomendações foram elaboradas de acordo com a metodologia Grading of Recommendations Assessment, Development and Evaluation (GRADE), que classifica a força da recomendação (forte ou fraca/condicional) e a certeza da evidência (alta, moderada, baixa ou muito baixa) ( Quadros 1.2 e 1.3 ).

**Quadro 1.2:** Força da recomendação

Categoria	Definição
FORTE	Deve ser feito, para a maioria dos pacientes, em praticamente todas as circunstâncias.
CONDICIONAL	Pode ser feito, dependendo de circunstâncias individuais, preferências e contexto.

**Quadro 1.3:** Certeza da evidência

Categoria	Definição
ALTA	Há forte confiança de que o verdadeiro efeito esteja próximo daquele estimado.
MODERADA	Há confiança moderada no efeito estimado.
BAIXA	A confiança no efeito é limitada.
MUITO BAIXA	A confiança na estimativa de efeito é muito limitada. Há importante grau de incerteza nos achados.

Metodologia:

- A elaboração da diretriz foi estruturada em capítulos temáticos, conduzidos por coordenadores com expertise reconhecida nas respectivas áreas. Cada coordenador foi responsável por compor um grupo de autores, com os quais realizou revisão crítica da literatura científica disponível, visando embasar as recomendações com as melhores evidências.
- Os grupos de trabalho realizaram reuniões periódicas para discussão dos temas e construção das recomendações, sob supervisão da coordenação geral da diretriz. As recomendações foram posteriormente analisadas e ajustadas pela comissão editorial, com base em critérios técnicos e científicos previamente definidos.
- O texto final de cada capítulo foi submetido à revisão e aprovação por todos os membros da comissão, assegurando alinhamento metodológico e consistência entre os conteúdos apresentados.

### 1.6. Sumário de recomendações

**Table:** Recomendações para coleta e interpretação do perfil lipídico no diagnóstico da dislipidemia

Recomendação	Força da recomendação	Certeza da evidência
Recomenda-se coletar a amostra para a realização do perfil lipídico idealmente em condição metabólica estável.	FORTE	MODERADA
Para a avaliação inicial, é aceitável obter a amostra sem jejum, em particular em populações selecionadas, como crianças e idosos.	FORTE	MODERADA
Se os triglicérides estiverem elevados (> 440 mg/dL) em amostra sem jejum, recomenda-se nova coleta em jejum de 12 horas, de acordo com critério do médico solicitante.	FORTE	MODERADA

**Table:** Recomendações para o cálculo do colesterol da lipoproteína de baixa densidade (LDL-c)

Recomendação	Força da recomendação	Certeza da evidência
Recomenda-se a favor do uso da equação de Martin/Hopkins para cálculo de LDL-c para todos os indivíduos.	FORTE	MODERADA
Quando os valores de triglicérides estão acima de 800 mg/dL, os resultados de LDL-c pela fórmula de Martin podem estar subestimados. Recomenda-se a avaliação do não-HDL-c.	FORTE	MODERADA

LDL-c: colesterol da lipoproteína de baixa densidade; não-HDL-c: colesterol não HDL.

**Table:** Recomendações sobre a utilização do colesterol não-HDL (não-HDL-c) na avaliação do risco cardiovascular

Recomendação	Força da recomendação	Certeza da evidência
Tanto o LDL-c quanto o não-HDL-c apresentam grande utilidade na avaliação do risco cardiovascular e como metas terapêuticas. O colesterol não-HDL é particularmente útil na estimativa da quantidade de lipoproteínas aterogênicas circulantes em indivíduos com triglicérides > 150 mg/dL.	FORTE	ALTA

LDL-c: colesterol da lipoproteína de baixa densidade; não-HDL-c: colesterol não-HDL.

**Table:** Recomendações sobre a utilização da Apolipoproteína B (ApoB) na avaliação do risco cardiovascular

Recomendação	Força da recomendação	Certeza da evidência
A medida da ApoB pode auxiliar na avaliação de risco cardiovascular e guiar a terapia em indivíduos com triglicérides > 150 mg/dL.	FORTE	MODERADA
O não-HDL-c é atualmente uma escolha mais prática porque pode ser facilmente calculado e não acarreta despesas adicionais para o paciente ou para o sistema de saúde.	FORTE	ALTA

ApoB: Apolipoproteína B; não-HDL-c: colesterol não HDL.

**Table:** Recomendações sobre a utilização da lipoproteína(a) (Lp(a)) na estratificação de risco cardiovascular

Recomendação	Força da recomendação	Certeza da evidência
Na população geral, é recomendada a dosagem de Lp(a) uma vez na vida, quando disponível, para auxiliar na estratificação de risco e/ou manejo terapêutico.	FORTE	MODERADA
Em populações específicas, como aquelas com DAC precoce, estenose aórtica, HF, história familiar de DCVA precoce ou de Lp(a) aumentada, é recomendada a dosagem de Lp(a) uma vez na vida, quando disponível, para auxiliar na estratificação de risco e/ou manejo terapêutico.	FORTE	ALTA
Recomenda-se como método preferencial para medir a Lp(a) um ensaio que seja independente da isoforma, ou seja, que meça o número de partículas por litro (nmol/L). A dosagem por unidade de massa (mg/dL) deve ser evitada. As fórmulas de conversão não corrigem a diferença entre os métodos e não são recomendadas.	FORTE	ALTA
A medida da Lp(a) por ensaio não independente da isoforma, ou seja, que mede unidades de massa (mg/dL), pode ser usada quando for a única disponível.	FORTE	ALTA
Em indivíduos com níveis aumentados de lipoproteína(a) ≥ 50 mg/dL (ou ≥ 125 nmol/L), cuja concentração é predominantemente determinada geneticamente, é recomendada a investigação em cascata nos familiares para auxiliar na identificação de outros possíveis portadores e na avaliação precoce do risco cardiovascular.	FORTE	ALTA

DAC: doença arterial coronariana; HF: hipercolesterolemia familiar; DCVA: Doença Cardiovascular Aterosclerótica; Lp(a): Lipoproteína(a).

**Table:** Recomendações para o teste genético na hipercolesterolemia familiar (HF)

Recomendação	Força da recomendação	Certeza da evidência
**Probando (caso-índice)** – O teste genético para HF deve ser oferecido a indivíduos de qualquer idade nos quais exista um forte indício clínico de suspeita de HF, com base no exame da história clínica e/ou familiar do paciente. Observação – esse índice de suspeita inclui: crianças com níveis persistentes * de LDL-c ≥ 160 mg/dL ou adultos com níveis persistentes * de LDL-c ≥ 190 mg/dL sem uma causa secundária aparente de hipercolesterolemia † e com pelo menos um familiar de primeiro grau igualmente afetado, ou com doença arterial coronária prematura ‡ , ou quando o histórico familiar não está disponível (por exemplo, adoção); crianças com níveis persistentes * de LDL-c ≥ 190 mg/dL ou adultos com níveis persistentes * de LDL-c ≥ 250 mg/dL sem uma causa secundária aparente de hipercolesterolemia † , mesmo na ausência de histórico familiar positivo.	FORTE	MODERADA
**Familiares sob risco –** O teste genético em cascata para a(s) variante(s) específica(s) identificada(s) no probando com hipercolesterolemia familiar (teste da variante familiar conhecida) deve ser oferecido a todos os parentes de primeiro grau. Se os parentes de primeiro grau não estiverem disponíveis ou não desejarem realizar o teste, o teste da variante familiar conhecida deve ser oferecido aos parentes de segundo grau. O teste genético em cascata deve continuar por toda a família extensa até que todos os indivíduos em risco tenham sido testados e todos os parentes com HF tenham sido identificados.	FORTE	MODERADA

* Persistente: níveis elevados de LDL-c confirmados em pelo menos duas dosagens separadas.

† Causas secundárias de hipercolesterolemia incluem hipotireoidismo, síndrome nefrótica, doença hepática colestática e certos medicamentos.

‡ Doença arterial coronária prematura: < 55 anos em homens e < 65 anos em mulheres. HF: hipercolesterolemia familiar; LDL-c: colesterol da lipoproteína de baixa densidade.

**Table:** Recomendações para o teste genético para hipercolesterolemia familiar em diferentes cenários clínicos

Recomendação	Força da recomendação	Certeza da evidência
Pode ser considerado teste genético para HF em adultos sem níveis de LDL-c disponíveis antes do tratamento, mas com histórico pessoal de doença arterial coronária prematura ‡ e histórico familiar de hipercolesterolemia e doença arterial coronária prematura ‡ .	CONDICIONAL	MODERADA
Pode ser considerado teste genético em adultos com níveis persistentes * de LDL-c ≥ 160 mg/dL (sem uma causa secundária aparente de hipercolesterolemia † ) no contexto de histórico familiar de hipercolesterolemia e histórico pessoal ou familiar de doença arterial coronária prematura ‡ .	CONDICIONAL	MODERADA

* Persistente: níveis elevados de LDL-c confirmados em pelo menos duas dosagens separadas;

† Causas secundárias de hipercolesterolemia incluem hipotireoidismo, síndrome nefrótica, doença hepática colestática e certos medicamentos;

‡ Doença arterial coronária prematura: < 55 anos em homens e < 65 anos em mulheres. LDL-c: colesterol da lipoproteína de baixa densidade.

**Table:** Recomendações para exames diagnósticos complementares na suspeita da Síndrome da Quilomicronemia Familiar

Recomendação	Força da recomendação	Certeza da evidência
Recomenda-se a utilização de escores clínicos para diagnóstico de SQF.	FORTE	ALTA
Em circunstâncias ideais, o teste genético é o método recomendado para confirmação diagnóstica de SQF.	FORTE	ALTA
O painel genético para SQF deve incluir o sequenciamento dos genes LPL, GPIHBP1, LMF1, APOA5 e APOC2.	FORTE	ALTA

SQF: Síndrome da Quilomicronemia Familiar.

**Table:** Recomendações para estratificação do risco cardiovascular em adultos

Recomendação	Força da recomendação	Certeza da evidência
Para indivíduos entre 30-79 anos, sem doença cardiovascular prévia estabelecida, recomenda-se a favor do uso de equação de risco para estimar o risco de evento cardiovascular aterosclerótico em 10 anos.	FORTE	ALTA
Para indivíduos entre 30-79 anos, sem doença cardiovascular prévia estabelecida, recomenda-se a favor do uso do escore PREVENT para a estimar o risco de evento cardiovascular aterosclerótico.	FORTE	ALTA
Para a população de risco calculado intermediário, recomenda-se a favor do uso de fatores agravantes para reclassificar o risco, independentemente da faixa etária.	FORTE	ALTA
Para a população de risco calculado baixo ou com idade entre 18-30 anos, o uso de fatores agravantes pode ser empregado para reclassificar o risco.	FORTE	BAIXA
Para indivíduos classificados inicialmente como de risco intermediário, com idade > 40 anos e LDL-c entre 70-159 mg/dL, o escore de cálcio coronário pode ser útil para decidir sobre a necessidade e a intensidade da terapia hipolipemiante.	FORTE	MODERADA
Para indivíduos classificados inicialmente como de risco baixo, com idade > 40 anos e LDL-c entre 70-159 mg/dL, o escore de cálcio coronário é razoável para aqueles com histórico familiar de DCVA prematura para definir a necessidade e a intensidade da terapia hipolipemiante.	CONDICIONAL	MODERADA

PREVENT: Predicting Risk of Cardiovascular Disease Events; LDL-c: colesterol da lipoproteína de baixa densidade; DCVA: doença cardiovascular aterosclerótica.

**Table:** Recomendações para metas terapêuticas lipídicas conforme risco cardiovascular

Recomendação	Força da recomendação	Certeza da evidência
Em indivíduos de risco cardiovascular extremo, recomenda-se a favor das metas de LDL-c < 40 mg/dL e de não-HDL-c < 70 mg/dL.	FORTE	MODERADA
Em indivíduos de risco cardiovascular muito alto, recomenda-se a favor das metas de LDL-c < 50 mg/dL e de não-HDL-c < 80 mg/dL.	FORTE	ALTA
Em indivíduos de risco cardiovascular alto, recomenda-se a favor das metas de LDL-c < 70 mg/dL e de não-HDL-c < 100 mg/dL.	FORTE	ALTA
Em indivíduos de risco cardiovascular intermediário, recomenda-se a favor das metas de LDL-c < 100 mg/dL e de não-HDL-c < 130 mg/dL.	FORTE	ALTA
Em indivíduos de risco cardiovascular baixo, recomenda-se a favor das metas de LDL-c < 115 mg/dL e de não-HDL-c < 145 mg/dL.	FORTE	MODERADA
Em indivíduos de risco cardiovascular alto, muito alto ou extremo, recomenda-se a favor de uma redução percentual do LDL-c de pelo menos 50%.	FORTE	ALTA
Em indivíduos de risco cardiovascular baixo ou intermediário, recomenda-se a favor de uma redução percentual do LDL-c de pelo menos 30%.	FORTE	ALTA
Em todos os indivíduos, especialmente naqueles com níveis de LDL-c ou não-HDL-c acima da meta, recomenda-se a favor de intervenções em medidas de estilo de vida.	FORTE	ALTA
Em indivíduos de risco cardiovascular alto, muito alto ou extremo, recomenda-se a favor de terapia farmacológica associada a medidas de estilo de vida.	FORTE	ALTA
Em indivíduos de risco cardiovascular alto, muito alto ou extremo, com LDL-c ou não-HDL-c persistentemente acima da meta, recomenda-se a favor da intensificação da terapia farmacológica associada a medidas de estilo de vida.	FORTE	ALTA
Em indivíduos de risco cardiovascular baixo ou intermediário, com LDL-c ou não-HDL-c persistentemente ≥ 30 mg/dL acima da meta, recomenda-se a favor do início ou intensificação da terapia farmacológica associada a medidas de estilo de vida.	FORTE	ALTA
Em indivíduos com níveis de LDL-c e não-HDL-c dentro da meta estabelecida, recomenda-se a favor de considerar meta de ApoB para optar sobre intensificação terapêutica.	FORTE	MODERADA

LDL-c: colesterol da lipoproteína de baixa densidade; ApoB: Apolipoproteína B.

**Table:** Recomendações dietéticas para o tratamento das dislipidemias

Recomendação	% do valor calórico total	Força da recomendação	Certeza da evidência
Gorduras totais	20-35%	FORTE	ALTA
Gorduras saturadas	< 7%	FORTE	ALTA
Gorduras trans	Não ingerir	FORTE	ALTA
Ácidos graxos monoinsaturados	15%	FORTE	ALTA
Ácidos graxos poli-insaturados	5-10%	FORTE	ALTA
Fibras	25 g/dia	CONDICIONAL	MODERADA
Carboidratos totais	50-55%	FORTE	ALTA

**Table:** Recomendações sobre suplementos dietéticos, alimentos funcionais e medidas de estilo de vida no manejo das dislipidemias

Recomendação	Força da recomendação	Certeza da evidência
Em indivíduos fumantes, recomenda-se a favor da cessação do tabagismo para reduzir o risco cardiovascular.	FORTE	ALTA
Recomenda-se a favor da abordagem de espiritualidade e religiosidade na consulta médica pelo seu impacto positivo em saúde cardiovascular.	CONDICIONAL	MODERADA
Em indivíduos com sobrepeso ou obesidade, recomenda-se a favor da redução do peso por meio de medidas não farmacológicas para aumento dos níveis de HDL-c, redução de triglicérides e redução menos pronunciada de LDL-c.	FORTE	ALTA
Recomenda-se a favor de atividade física para reduzir o risco cardiovascular e promover melhorias no perfil lipídico, como o aumento de HDL-c e redução dos triglicérides.	FORTE	ALTA
Em todos os adultos, recomenda-se a favor de pelo menos 150 minutos por semana de atividade física aeróbica de intensidade moderada ou 75 minutos de atividade vigorosa, podendo-se combinar ambas para maior benefício.	FORTE	ALTA
Recomenda-se contra a ingestão de álcool com o objetivo de prevenir ou tratar a aterosclerose.	FORTE	MODERADA
Recomenda-se a favor de considerar suplementação * dietética no manejo das dislipidemias.	CONDICIONAL	MODERADA

* São consideradas suplementações com impacto nos níveis de LDL-c o arroz de levedura vermelho, probióticos, fitoesteróis e para redução de TG, óleo de peixe (EPA/DHA).

**Table:** Recomendações farmacológicas para o tratamento da dislipidemia

Recomendação	Força da recomendação	Certeza da evidência
Em indivíduos com indicação de terapia hipolipemiante, recomenda-se a favor da estatina como primeira opção de tratamento.	FORTE	ALTA
Em indivíduos sem tratamento com meta de redução de LDL-c de 50% ou mais, recomenda-se a favor da associação de estatina com ezetimiba como alternativa à estatina de alta intensidade.	FORTE	ALTA
Em indivíduos que não atingem o alvo a despeito de estatina em dose máxima tolerada, recomenda-se a favor de intensificação terapêutica com ezetimiba ou terapia anti-PCSK9.	FORTE	ALTA
Recomenda-se a favor do uso de inclisirana como alternativa aos anticorpos monoclonais inibidores da PCSK9, como evolocumabe ou alirocumabe.	FORTE	MODERADA
Em indivíduos intolerantes a estatinas e que não atingem o alvo a despeito de ezetimiba, recomenda-se a favor de intensificação terapêutica com ácido bempedoico.	FORTE	ALTA
Em indivíduos com diagnóstico de Hipercolesterolemia Familiar homozigótica que não atingem a meta de LDL-c apesar do uso das doses máximas de terapias hipolipemiantes, recomenda-se a favor do uso de evinacumabe a partir dos 5 anos de idade.	FORTE	MODERADA
Recomenda-se contra o tratamento farmacológico com o objetivo de aumentar o HDL-c.	FORTE	ALTA
Em indivíduos com hipertrigliceridemia (≥ 150 mg/dL) em que a terapia hipolipemiante é indicada para redução de risco cardiovascular, recomenda-se a favor das estatinas como terapia de escolha.	FORTE	ALTA
Em indivíduos com triglicérides entre 150 e 499 mg/dL que possuem DCVA ou alto risco cardiovascular, recomenda-se a favor de icosapenta etila (4 g/dia) para reduzir a incidência de eventos cardiovasculares maiores, embora não esteja disponível no Brasil.	CONDICIONAL	MODERADA
Em indivíduos com triglicérides entre 150 e 499 mg/dL que possuem DCVA ou alto risco cardiovascular, recomenda-se contra formulações de EPA com DHA para prevenir eventos cardiovasculares.	FORTE	ALTA
Em indivíduos com triglicérides ≥ 500 mg/dL de forma persistente a despeito de medidas de estilo de vida, recomenda-se a favor do uso de fibrato para reduzir o risco de pancreatite.	FORTE	MODERADA
Em adultos portadores da Síndrome da Quilomicronemia Familiar (SQF) com triglicérides ≥ 500 mg/dL, recomenda-se a favor de uso da volanesorsena para reduzir risco de pancreatite	FORTE	MODERADA

DCVA: doença cardiovascular aterosclerótica; LDL-c: colesterol da lipoproteína de baixa densidade; PCSK9: proproteína convertase subtilisina/quexina tipo 9; SQF: Síndrome da Quilomicronemia Familiar.

**Table:** Recomendações para o uso combinado de estatina, ezetimiba, ácido bempedoico e terapias anti-PCSK9

Recomendação	Força da recomendação	Certeza da evidência
Em indivíduos de alto risco cardiovascular, recomenda-se a favor da terapia inicial com estatina de alta intensidade e ezetimiba para atingir a meta terapêutica.	FORTE	ALTA
Em indivíduos de muito alto risco cardiovascular, recomenda-se a favor da terapia inicial com estatina de alta intensidade e ezetimiba, e potencialmente terapia anti-PCSK9, para atingir a meta terapêutica.	FORTE	ALTA
Em indivíduos de extremo risco cardiovascular, recomenda-se a favor do uso de terapia inicial com estatina de alta intensidade, ezetimiba e terapia anti-PCSK9 para atingir a meta terapêutica.	FORTE	ALTA
Em indivíduos que não atingirem a meta terapêutica com estatina de alta intensidade e ezetimiba, recomenda-se a favor de adicionar terapia anti-PCSK9 ou ácido bempedoico, conforme a meta terapêutica.	FORTE	ALTA
Em indivíduos com HF ou LDL-c ≥ 190 mg/dL, recomenda-se a favor do uso de estatina de alta intensidade + ezetimiba como terapia inicial para atingir a meta terapêutica.	FORTE	ALTA
Em indivíduos com intolerância à estatina, recomenda-se a favor da terapia combinada adaptada (por exemplo, ácido bempedoico com ezetimiba ou terapia anti-PCSK9 com ou sem ezetimiba), conforme meta terapêutica.	FORTE	ALTA

HF: hipercolesterolemia familiar; LDL-c: colesterol da lipoproteína de baixa densidade; PCSK9: proproteína convertase subtilisina/quexina tipo 9.

**Table:** Recomendações para o manejo diante de sintomas musculares relacionados a estatinas

Recomendação	Força da recomendação	Certeza da evidência
Em pacientes nos quais se pretende iniciar estatina, recomenda-se a favor da dosagem basal de CK e de enzimas hepáticas (ALT e AST), especialmente em indivíduos de alto risco para eventos musculares ou hepatotoxicidade.	CONDICIONAL	BAIXA
Em pacientes recebendo estatinas, recomenda-se contra dosagens rotineiras de CK e de enzimas hepáticas na ausência de sintomas musculares, de sinais de hepatotoxicidade ou de alterações na terapia.	FORTE	MODERADA
Em pacientes recebendo estatinas, recomenda-se a favor da dosagem de CK na presença de sintomas musculares graves, e da dosagem de enzimas hepáticas na presença de sinais de hepatotoxicidade.	FORTE	MODERADA
Em pacientes que não toleram a dose sugerida de estatina, recomenda-se a favor de estratégias alternativas para atingir a meta de redução do LDL-c, incluindo a redução da frequência de administração, a troca para outra estatina ou a combinação de outros agentes hipolipemiantes.	FORTE	ALTA
Em pacientes nos quais se opta pela suspensão da estatina, recomenda-se a favor do início imediato da terapia hipolipemiante não estatínica (por exemplo, ezetimiba, ácido bempedoico ou terapia anti-PCSK9) durante o período de suspensão, com o objetivo de mitigar o risco cardiovascular decorrente da elevação do LDL-c.	FORTE	ALTA
Em pacientes recebendo estatinas, recomenda-se contra a reposição de vitamina D com o objetivo de mitigar sintomas musculares associados à estatina.	FORTE	ALTA
Em pacientes recebendo estatinas, recomenda-se contra a suplementação rotineira de coenzima Q10 com o objetivo de mitigar sintomas musculares associados à estatina.	FORTE	MODERADA

CK: Creatina Quinase; ALT: Alanina aminotransferase; AST: Aspartato aminotransferase; LDL-c: colesterol da lipoproteína de baixa densidade; PCSK9: proproteína convertase subtilisina/quexina tipo 9.

**Table:** Recomendações para o uso de terapias hipolipemiantes em pacientes com insuficiência cardíaca

Recomendação	Força da recomendação	Certeza da evidência
Pacientes com IC e doença aterosclerótica estabelecida devem manter o uso de estatinas para reduzir o risco de eventos cardiovasculares ateroscleróticos.	FORTE	MODERADA
Para população com IC em uso prévio de estatina, recomenda-se contra a suspensão da mesma caso o paciente apresente sobrevida clinicamente aceitável.	FORTE	MODERADA
Pacientes com ICFER sem doença aterosclerótica podem utilizar estatinas, desde não haja contraindicação e considerando individualização do tratamento.	CONDICIONAL	MODERADA
Terapia anti-PCSK9 devem ser mantidos para pacientes com IC de alto risco cardiovascular quando as metas de LDL-c não forem alcançadas com estatinas e ezetimiba.	FORTE	MODERADA

IC: insuficiência cardíaca; ICFER: insuficiência cardíaca com fração de ejeção reduzida; LDL-c: colesterol da lipoproteína de baixa densidade; PCSK9: proproteína convertase subtilisina/quexina tipo 9.

**Table:** Recomendações para o manejo da dislipidemia em pessoas vivendo com HIV

Recomendação	Força da recomendação	Certeza da evidência
Para PVHIV, as estatinas devem ser consideradas como terapia de primeira linha para a redução do LDL-c e do risco cardiovascular, de acordo com a meta pertinente. A escolha da estatina deve levar em consideração risco de interação medicamentosa.	FORTE	ALTA
Para PVHIV com intolerância às estatinas ou redução insuficiente do LDL-c, o ezetimiba deve ser adicionado.	FORTE	MODERADA
Para PVHIV, a terapia anti-PCSK9, como o evolocumabe, devem ser considerados em PVHIV com alto risco cardiovascular e controle inadequado do LDL-c, apesar da terapia máxima.	FORTE	MODERADA

PVHIV: Pessoas Vivendo com HIV; LDL-c: colesterol da lipoproteína de baixa densidade; PCSK9: proproteína convertase subtilisina/quexina tipo 9.

**Table:** Recomendações para o manejo da dislipidemia em pessoas com diabetes *mellitus*

Recomendação	Força da recomendação	Certeza da evidência
Para população com diabetes *mellitus* , a estatina é o hipolipemiante de escolha inicial para pacientes com LDL-c acima da meta estabelecida.	FORTE	ALTA
Para população com diabetes *mellitus* , ezetimiba pode ser utilizado para os indivíduos que permanecem com LDL-c acima da meta, a despeito de terapia com estatina em dose máxima tolerada.	FORTE	MODERADA
Para população com diabetes *mellitus* , a terapia anti-PCSK9 pode ser utilizado para os indivíduos que permanecem com LDL-c acima da meta, a despeito de terapia com estatina em dose máxima tolerada e ezetimiba.	FORTE	MODERADA
Para população com diabetes *mellitus* e retinopatia leve, o fenofibrato pode ser utilizado para redução da progressão da retinopatia diabética.	FORTE	MODERADA

LDL-c: colesterol da lipoproteína de baixa densidade; PCSK9: proproteína convertase subtilisina/quexina tipo 9.

**Table:** Recomendações para o manejo da dislipidemia em pacientes com hipotireoidismo

Recomendação	Força da recomendação	Certeza da evidência
Em pacientes com hipotireoidismo clínico e dislipidemia, recomenda-se a favor da reposição hormonal com levotiroxina, visando a normalizar o TSH e melhorar o perfil lipídico.	FORTE	ALTA
Em pacientes com hipotireoidismo clínico e dislipidemia, recomenda-se a favor do uso de estatinas em pacientes cuja dislipidemia persiste após normalização da função tireoidiana, principalmente em presença de risco cardiovascular elevado.	FORTE	MODERADA
Em pacientes com hipotireoidismo subclínico e dislipidemia, recomenda-se a favor do uso de levotiroxina se o TSH estiver entre 4,5 e 9,9 mUI/L e houver sintomas de hipotireoidismo ou alto risco cardiovascular.	FORTE	MODERADA
Em pacientes com hipotireoidismo subclínico e dislipidemia, recomenda-se a favor do uso de levotiroxina se o TSH estiver acima de 10 mUI/L.	FORTE	ALTA

TSH: Hormônio Estimulante da Tireoide.

**Table:** Recomendações para o manejo da dislipidemia em pacientes com doença renal crônica

Recomendação	Força da recomendação	Certeza da evidência
Em indivíduos com DRC estágio 1-3 e risco CV aumentado, recomenda-se a favor do uso de estatinas de alta potência para reduzir risco CV.	FORTE	ALTA
Em indivíduos com DRC estágio 1-3 e risco CV aumentado que não atingiram as metas, recomenda-se a favor da associação de ezetimiba às estatinas de alta potência.	FORTE	MODERADA
Em indivíduos com DRC estágio 4-5 não dialítico, recomenda-se a favor de iniciar estatina de alta potência, associada ou não ao uso de ezetimiba.	FORTE	ALTA
Em indivíduos com DRC em programa de diálise, sem doença cardiovascular estabelecida, recomenda-se contra o início de estatinas.	FORTE	ALTA

DRC: Doença Renal Crônica; CV: Cardiovascular.

**Table:** Recomendações para o manejo da dislipidemia em pacientes com obesidade

Recomendação	Força da recomendação	Certeza da evidência
Em indivíduos com dislipidemia secundária à obesidade, recomenda-se a favor de intervenções não farmacológicas, que constituem a linha no tratamento da dislipidemia nessa condição.	FORTE	ALTA
Em indivíduos com dislipidemia secundária à obesidade, recomenda-se a favor do uso de estatinas como base do tratamento farmacológico.	FORTE	ALTA
Em indivíduos com dislipidemia secundária à obesidade, recomenda-se a favor do uso de fibratos quando os triglicérides estiverem ≥ 500 mg/dL, visando à redução do risco de pancreatite.	FORTE	MODERADA
Em indivíduos com dislipidemia secundária à obesidade, recomenda-se contra o uso de fibratos para redução do risco cardiovascular ou para reduzir o risco de pancreatite quando os triglicérides estiverem < 500 mg/dL.	FORTE	ALTA
Em indivíduos com dislipidemia secundária à obesidade, recomenda-se a favor do uso de análogos do GLP-1 por seu efeito duplo (perda de peso e redução de eventos cardiovasculares).	FORTE	ALTA
Em indivíduos com dislipidemia secundária à obesidade, recomenda-se a favor da cirurgia bariátrica para melhorar o perfil lipídico e reduzir eventos cardiovasculares.	FORTE	MODERADA

**Table:** Recomendações para o tratamento farmacológico da dislipidemia em idosos

Recomendação	Força da recomendação	Certeza da evidência
Após os 75 anos de idade, recomenda-se a favor da individualização das doses dos hipolipemiantes, de acordo com a fragilidade, presença de comorbidades, expectativa de vida e o uso de polifarmácia.	FORTE	MODERADA

**Table:** Recomendações para o manejo da dislipidemia em crianças

Recomendação	Força da recomendação	Certeza da evidência
Para população pediátrica, recomenda-se a favor da dosagem do perfil lipídico completo de forma universal entre 9 e 11 anos de idade.	FORTE	MODERADA
Para população pediátrica com fatores de risco (citados no texto), recomenda-se a favor de dosagem do perfil lipídico completo a partir dos 2 anos de idade.	FORTE	MODERADA
Para população pediátrica, recomenda-se a favor de estimular modificação de estilo de vida com orientação nutricional, controle de peso e atividade física compatível com a idade e quadro clínico.	FORTE	ALTA
Para população pediátrica que está acima da meta de LDL-c, a despeito de modificação do estilo de vida, recomenda-se a favor da terapia hipolipemiante com estatina a partir dos 8 anos de idade.	FORTE	MODERADA
Para população pediátrica que está acima da meta de LDL-c, a despeito de modificação do estilo de vida, recomenda-se a favor da terapia por ezetimiba a partir dos 6 anos de idade e terapia combinada com estatina a partir dos 8 anos de idade.	CONDICIONAL	BAIXA
Para população pediátrica, de acordo com avaliação médica e risco do paciente e conforme o nível do LDL-c, que já esteja em tratamento com estatina e ezetimiba, recomenda-se a favor de considerar uso de evolocumabe a partir dos 10 anos de idade ou alirocumabe a partir dos 8 anos de idade.	FORTE	MODERADA
Para população pediátrica com diagnóstico de Hipercolesterolemia Familiar homozigótica recomenda-se a favor do uso de evinacumabe a partir dos 5 anos de idade, que não atingem a meta de LDL-c apesar do uso das doses máximas de terapias hipolipemiantes.	FORTE	MODERADA

LDL-c: colesterol da lipoproteína de baixa densidade.

**Table:** Recomendações para o manejo da dislipidemia em pacientes transplantados

Recomendação	Força da recomendação	Certeza da evidência
Recomenda-se a favor de considerar todos os pacientes transplantados como tendo um agravante de risco cardiovascular.	FORTE	MODERADA
Em todos os pacientes transplantados, recomenda-se a favor de medir o perfil lipídico cerca de 2 a 3 meses após o transplante.	FORTE	MODERADA
Em pacientes transplantados, recomenda-se a favor de utilizar estatinas como primeira linha de tratamento da dislipidemia para reduzir eventos cardiovasculares.	FORTE	ALTA
Em pacientes transplantados, recomenda-se contra o uso de sinvastatina ou lovastatina associado a ciclosporina, tacrolimo, sirolimo ou everolimo.	FORTE	ALTA
Em pacientes transplantados utilizando ciclosporina recomenda-se a favor de utilizar dose máxima de 5 mg de rosuvastatina ou 10 mg de atorvastatina para evitar interação medicamentosa.	FORTE	MODERADA
Em pacientes transplantados em uso de imunossupressores, recomenda-se a favor do uso de estatinas com menor risco de rabdomiólise, como pravastatina, fluvastatina ou rosuvastatina.	FORTE	MODERADA

**Table:** Recomendações para o manejo da dislipidemia em pacientes com doenças hepáticas crônicas

Recomendação	Força da recomendação	Certeza da evidência
O uso de estatinas pode ser indicado para pacientes com esteatose hepática, na presença de enzimas hepáticas alteradas (até três vezes os valores de referência da normalidade), para redução do risco cardiovascular.	FORTE	ALTA
O uso de estatinas pode ser indicado para pacientes com esteatose hepática, na presença de enzimas hepáticas alteradas (até três vezes os valores de referência da normalidade), para melhora da evolução hepática.	FORTE	MODERADA
A doença hepática crônica compensada (Child-Pugh A e B) não é considerada contraindicação absoluta para a início ou manutenção da terapia com estatina e terapia anti-PCSK9.	FORTE	MODERADA
Para pacientes portadores de insuficiência hepática avançada (Child B e C) recomenda-se contra o uso da ezetimiba.	CONDICIONAL	MODERADA

PCSK9: proproteína convertase subtilisina/quexina tipo 9.

**Table:** Recomendações para diagnóstico laboratorial e tratamento da dislipidemia em pacientes com síndrome coronariana aguda (SCA)

Recomendação	Força da recomendação	Certeza da evidência
Em pacientes com SCA, recomenda-se a favor da dosagem do perfil lipídico precocemente (preferencialmente em até 24 horas do evento agudo) como base para decisões terapêuticas.	FORTE	MODERADA
Em pacientes com SCA, recomenda-se a favor de dosar o perfil lipídico dentro de 4 a 6 semanas após a alta hospitalar.	FORTE	MODERADA
Em pacientes com SCA, recomenda-se a favor de iniciar estatinas potentes nas primeiras 24h após internação por SCA.	FORTE	ALTA
Em pacientes com SCA, recomenda-se a favor de iniciar estatinas potentes associadas à ezetimiba ainda na fase aguda, além de considerar precocemente terapia anti-PCSK9 em indivíduos com risco muito alto ou extremo, como estratégia intensiva voltada à rápida redução dos níveis de LDL-c, minimização da inércia terapêutica e maior probabilidade de alcançar as metas lipídicas recomendadas.	FORTE	MODERADA

SCA: síndrome coronariana aguda; PCSK9: proproteína convertase subtilisina/quexina tipo 9; LDL-c: colesterol da lipoproteína de baixa densidade.

**Table:** Recomendações para o manejo da dislipidemia em pacientes com doenças imunomediadas

Recomendação	Força da recomendação	Certeza da evidência
Em indivíduos com artrite reumatoide, com critérios de alto risco para a doença imunomediada, recomenda-se a favor de considerar agravante de risco cardiovascular.	FORTE	MODERADA
Em indivíduos com doenças imunomediadas, recomenda-se a favor do controle adequado da atividade inflamatória como estratégia essencial para reduzir o risco cardiovascular.	FORTE	MODERADA
Em indivíduos com doenças imunomediadas, recomenda-se a favor do uso de CAC para estratificação do risco em pacientes com risco intermediário.	CONDICIONAL	ALTA
Em indivíduos com doenças imunomediadas recomenda-se a favor do uso de estatinas como primeira linha no tratamento da dislipidemia.	FORTE	MODERADA
Em indivíduos com doenças imunomediadas: Quando houver intolerância ou resposta inadequada às estatinas, recomenda-se a favor de adicionar ezetimiba ou, se risco muito alto, terapia anti-PCSK9.	FORTE	MODERADA

CAC: escore de cálcio coronariano.

**Table:** Recomendações para o manejo da dislipidemia em gestantes

Recomendação	Força da recomendação	Certeza da evidência
Para gestantes com dislipidemia relacionadas à gestação ou outras formas de dislipidemia primária ou secundária, recomenda-se a favor de seguir dieta com baixo teor de gordura, alto teor de fibras solúveis e carboidratos de baixo índice glicêmico.	FORTE	MODERADA
Para mulheres em planejamento de engravidar, em uso prévio de estatina, recomenda-se a favor de interromper a estatina 60 dias antes da concepção.	FORTE	MODERADA
Para gestantes em uso de estatina, recomenda-se a favor de suspensão imediata do medicamento, devendo o mesmo ser reiniciado após o período de amamentação.	FORTE	MODERADA
Para gestantes de muito alto risco, recomenda-se a favor de individualização terapêutica e decisão compartilhada sobre o reinício da estatina no 3º trimestre da gestação.	CONDICIONAL	MODERADA
Para população gestante com hipercolesterolemia, recomenda-se a favor do uso de resinas de troca.	CONDICIONAL	BAIXA
Para gestantes e lactantes, recomenda-se contra uso da ezetimiba, terapias anti-PCSK9, inibidores da ANGPTL3 (como evinacumabe), ácido bempedoico e lomitapida.	FORTE	MODERADA
Para gestante com triglicérides acima de 880 mg/dL a despeito de modificação de estilo de vida, recomenda-se a favor do uso de fenofibrato no 2º trimestre da gestação.	CONDICIONAL	BAIXA
Para gestante com triglicérides acima de 880 mg/dL a despeito de modificação de estilo de vida, recomenda-se a favor do uso de ácidos graxos ômega-3.	CONDICIONAL	BAIXA

PCSK9: proproteína convertase subtilisina/quexina tipo 9.

**Table:** Recomendações para o manejo da dislipidemia em mulheres segundo o risco cardiovascular

Recomendação	Força da recomendação	Certeza da evidência
Para mulheres classificadas como de risco cardiovascular baixo ou intermediário, recomenda-se a favor do uso clínico de agravantes de risco, com o objetivo de refinar a estratificação de risco e orientar decisões terapêuticas mais intensivas.	FORTE	MODERADA
Para mulheres classificadas como de alto risco, muito alto risco ou risco extremo, recomenda-se a favor da adoção de terapia intensiva e combinada.	FORTE	ALTA

## 2. Epidemiologia

### 2.1. Níveis Médios de Lípides Plasmáticos e Prevalências das Dislipidemias

**Figura 2.1:**
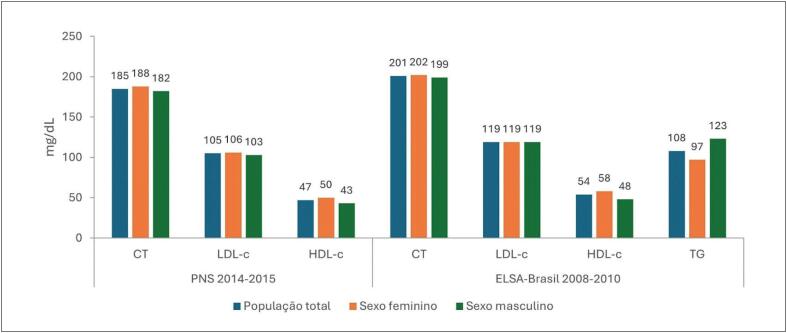
Níveis de lípides sanguíneos na população brasileira, de acordo com sexo. Níveis médios de colesterol total (CT), colesterol da lipoproteína de baixa densidade (LDL-c) e colesterol da lipoproteína de alta densidade (HDL-c) e níveis medianos de triglicérides (TG) na Pesquisa Nacional de Saúde (PNS) de 2014-2015 e na linha de base do *Estudo Longitudinal de Saúde do Adulto* (ELSA-Brasil).

**Figura 2.2:**
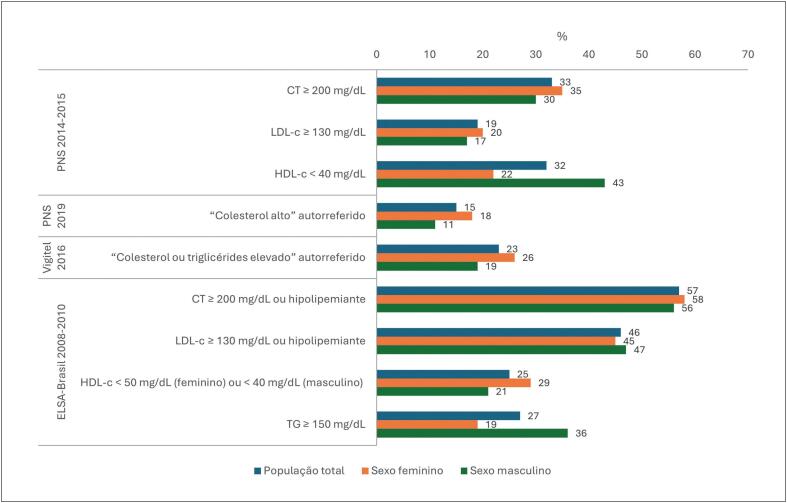
Alterações lipídicas na população brasileira, de acordo com sexo. Prevalências de alterações lipídicas na Pesquisa Nacional de Saúde (PNS) de 2014-2015, na PNS de 2019, na Vigilância de Fatores de Risco e Proteção para Doenças Crônicas por Inquérito Telefônico (Vigitel) de 2016 e na linha de base do *Estudo Longitudinal de Saúde do Adulto* (ELSA-Brasil). CT: colesterol total; HDL-c: colesterol da lipoproteína de alta densidade; LDL-c: colesterol da lipoproteína de baixa densidade; TG: triglicérides.

Informações epidemiológicas sobre as dislipidemias no Brasil podem ser obtidas de inquéritos populacionais e estudos observacionais. No contexto da Pesquisa Nacional de Saúde (PNS) de 2014-2015, amostras sanguíneas foram coletadas de uma subpopulação de 8.534 indivíduos adultos para se estimar valores representativos do perfil lipídico da população brasileira. Os níveis médios de colesterol total (CT) e LDL-c foram 185 mg/dL e 105 mg/dL, respectivamente, com valores discretamente mais elevados em mulheres em comparação aos homens ( Figura 2.1 ). Estimou-se que uma em cada três pessoas apresentava CT > 200 mg/dL e um em cada cinco indivíduos tinha LDL-c > 130 mg/dL, com prevalências maiores no sexo feminino ( Figura 2.2 ). Níveis mais baixos de CT e LDL-c foram encontrados em jovens de 18 a 29 anos, valores intermediários entre 30 e 44 anos e concentrações mais elevadas em indivíduos com 45 anos ou mais. As prevalências de CT e LDL-c elevados foram maiores entre pessoas com menor escolaridade.^11 , 15^

Fontes: Pesquisa Nacional de Saúde de 2014-2015^15^ e base de dados do ELSA-Brasil.

Fontes: PNS de 2014-2015,^15^ PNS de 2019,^16^ Vigitel de 2016^17^ e base de dados do ELSA-Brasil.

Em relação à PNS de 2014-2015, menores prevalências de dislipidemia autorreferida foram encontradas na PNS de 2019^16^ (estudo de abrangência nacional envolvendo 88.531 indivíduos adultos) e na *Vigilância de Fatores de Risco e Proteção para Doenças Crônicas por Inquérito Telefônico* de 2016^17^ (53.210 entrevistas com pessoas adultas das capitais dos 26 estados brasileiros e do Distrito Federal) ( Figura 2.2 ).

Outra fonte de dados lipídicos da população brasileira é o *Estudo Longitudinal de Saúde do Adulto* (ELSA-Brasil), uma coorte prospectiva com 15.105 servidores públicos que inicialmente tinham de 35 a 74 anos (média de 52 ± 9 anos) e que eram oriundos de seis capitais (Salvador, Belo Horizonte, Vitória, Rio de Janeiro, São Paulo e Porto Alegre).^18^ Em relação à PNS de 2014-2015, a linha de base do ELSA-Brasil (2008-2010) revelou níveis mais elevados de CT, LDL-c e HDL-c, bem como prevalências substancialmente maiores de CT ou LDL-c elevados ( Figura 2.1 e Figura 2.2 ). Diferenças nas características populacionais podem justificar essas discrepâncias.

O ELSA-Brasil também foi utilizado para estimar a prevalência de HF com base nos critérios da Dutch Lipid Clinic Network, estratificada por sexo e cor da pele. A prevalência geral foi de 3,8 por 1.000 indivíduos, equivalente a 1 em cada 263. Essa taxa foi mais alta em mulheres (1:244) do que em homens (1:333) e mais elevada entre pessoas pardas (1:204) e pretas (1:156), em comparação com pessoas brancas (1:417).^19^

**Figura 2.3:**
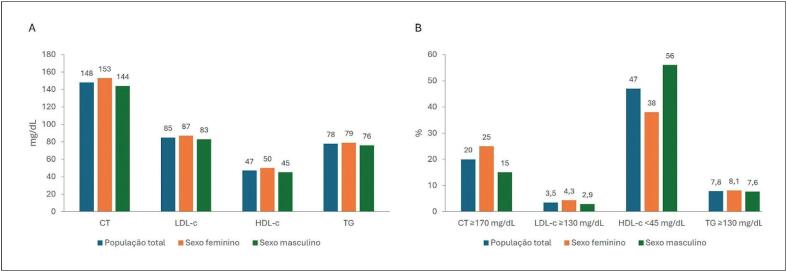
Lípides sanguíneos em crianças e adolescentes no Brasil, estratificados por sexo, de acordo com dados do *Estudo de Riscos Cardiovasculares em Adolescentes* (ERICA). A. Níveis médios de colesterol total (CT), colesterol da lipoproteína de baixa densidade (LDL-c), colesterol da lipoproteína de alta densidade (HDL-c) e triglicérides (TG). B. Prevalências de alterações lipídicas. Fonte: ERICA.^20^

Informação epidemiológica sobre dislipidemias em crianças e adolescentes foi fornecida pelo *Estudo de Riscos Cardiovasculares em Adolescentes* (ERICA), projeto de abrangência nacional, de base escolar, com 38.069 adolescentes de 12 a 17 anos residentes nas 27 capitais brasileiras ou seus entornos, realizado entre 2013 e 2014. Os resultados principais são mostrados na Figura 2.3 . De cada cinco adolescentes, um apresentava CT ≥ 170 mg/dL, sendo a prevalência maior no sexo feminino do que no masculino.^20^

### 2.2. Mortalidade Cardiovascular Atribuível ao LDL-colesterol Elevado

Segundo dados do GBD, o LDL-c elevado é o segundo fator de risco responsável por mortes cardiovasculares no Brasil, após a hipertensão. Estima-se que a taxa de mortalidade cardiovascular atribuível à elevação do LDL-c, padronizada por idade, tenha caído de 49,6 para 32,1 por 100.000 habitantes entre 2001 e 2021 no Brasil.^1^ Essa redução reflete avanços no controle de fatores de risco, maior acesso aos serviços de saúde e melhorias na qualidade do cuidado. No entanto, considerando o crescimento populacional no mesmo período, o número absoluto anual estimado de mortes por DCV atribuíveis à elevação do LDL-c aumentou de 60.716 para 79.604,^1^ destacando a relevância epidemiológica da hipercolesterolemia.

### 2.3. Dados sobre Tratamento e Metas Atingidas

Dados sobre o tratamento e metas lipídicas atingidas no Brasil são escassos e, portanto, é desafiador traçar o perfil do tratamento da dislipidemia no Brasil. O estudo ELSA-Brasil revela que estávamos distantes do panorama ideal em 2008-2010: entre os participantes com LDL-c elevado, 42,3% estavam em uso de terapia hipolipemiante e apenas 58,3% atingiam as metas recomendadas pelo *National Cholesterol Education Program Adult Treatment Panel III* (NCEP-ATPIII).^21^ O estudo *Lipid Treatment Assessment Project 2* (L-TAP 2, 2006-2007) levantou dados de tratamento lipídico em nove países, incluindo o Brasil (n = 391), e constatou percentual semelhante (62,1%) de brasileiros dentro da meta de LDL-c.^22^ Um cenário preocupante surge quando focamos a atenção no tratamento dos portadores de dislipidemia grave (LDL-c > 190 mg/dL): em um estudo de uma instituição privada brasileira com dados de 2004 a 2019, o uso de medicação hipolipemiante inicial foi de 5,9%, subindo para 45,4% no final do seguimento, sendo que apenas 19,1% alcançaram uma redução do LDL-c acima de 50%.^23^

**Figura 2.4:**
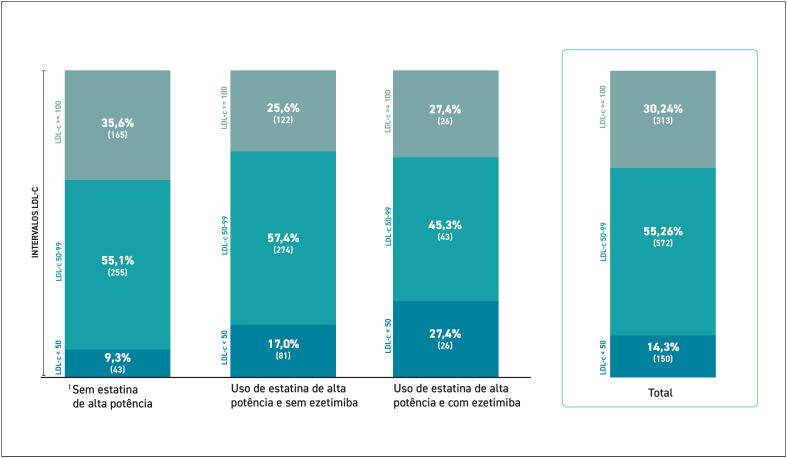
Distribuição do LDL-colesterol (LDL-c) em pacientes sob tratamento de acordo com o grupo terapêutico do Estudo NEAT na população com LDL-c Disponível (n =1035): faixa de LDL-colesterol n (%). Foram excluídos da análise pacientes sem tratamento (n = 36) e aqueles em terapia com anti-PCSK9 (n = 2).

**Figura 2.5:**
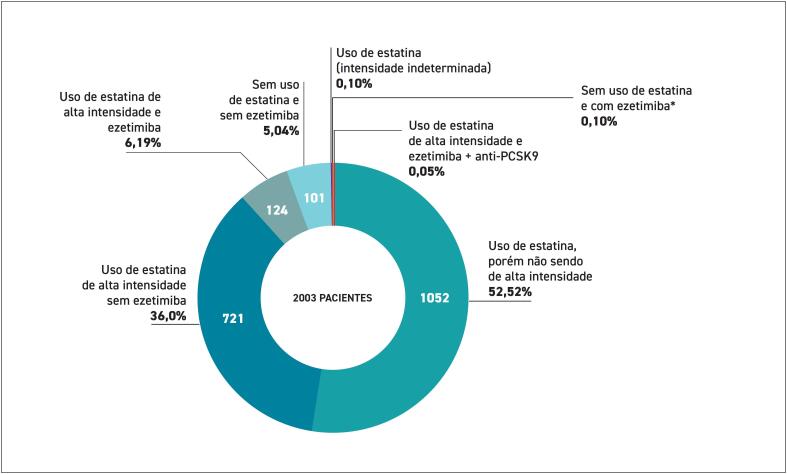
Distribuição dos grupos terapêuticos entre os pacientes com LDL disponível (N=2003): grupo terapêutico n(%). *1 paciente do grupo sem uso de estatina e com ezetimiba estava em uso de anti-PCSK9.

Evidências mais recentes sobre o manejo da dislipidemia no Brasil foram reportadas peloo registro *NEtwork to control AtheroThrombosis* (NEAT), que envolveu 25 centros (56% públicos) distribuídos pelas cinco regiões do país. O estudo avaliou 2.003 pacientes com doença arterial coronária e/ou periférica entre 2020 e 2022. Cerca de 5,1% dos pacientes não utilizavam estatinas e, entre aqueles em uso, 55,4% não estavam sob terapia de alta intensidade. Dentre os pacientes com dosagem de LDL-c, apenas 14,4% apresentavam valores de LDL-c inferiores a 50 mg/dL enquanto que aproximadamente 30% dos indivíduos estavam com níveis ≥ 100 mg/dL ( Figura 2.4 ). Destaca-se, na população total do estudo, igualmente a baixa utilização de terapias combinadas: apenas 6,19% dos pacientes faziam uso concomitante de estatina de alta intensidade e ezetimiba, e somente 1 paciente (0,05%) estava em terapia tripla, utilizando estatina de alta intensidade, ezetimiba e anti-PCSK9, conforme ilustrado na Figura 2.5 .^24^ A principal barreira à adoção de condutas baseadas em evidências não foi de ordem financeira, mas sim decisão médica pela não indicação dessas estratégias terapêuticas.^25^

Resultados alarmantes foram reportados por um estudo transversal de mundo real no contexto do programa Estratégia de Saúde da Família, com dados de 2016 a 2021. Entre mais de 35.000 adultos com histórico de infarto do miocárdio ou AVC, somente 6,7% e 0,6% estavam em uso de estatina e estatina em alta dose, respectivamente.^26^

Fora do Brasil, o alcance de metas terapêuticas de LDL-c também está longe do cenário ideal. O estudo observacional *EU-Wide Cross-Sectional Observational Study of Lipid-Modifying Therapy Use in Secondary and Primary Care* (DA VINCI) analisou o alcance de metas em indivíduos de prevenção primária e secundária que receberam prescrição de hipolipemiante (94% recebiam estatina). Os dados foram coletados entre 2017 e 2018, em 18 países da Europa. Apenas 33% atingiram as metas estabelecidas pela diretriz europeia de 2019 (54% para a diretriz de 2016), em um contexto de baixa taxa de uso de estatina de alta intensidade (22% na prevenção primária e 42% na secundária). Além disso, a prevalência de terapia combinada foi muito baixa: 9% com ezetimiba e 1% com inibidor de PCSK9.^27^

No registro multicêntrico *Getting to an imprOved Understanding of Low-Density Lipoprotein Cholesterol and Dyslipidemia Management* (GOULD), nos EUA, realizado entre 2016 e 2020, entre indivíduos em prevenção secundária que não estavam em uso de inibidor de PCSK9, o tratamento hipolipemiante foi intensificado em apenas 22% dos que estavam com LDL-c ≥ 100 mg/dL e em 14% daqueles com LDL-c entre 70 e 99 mg/dL, ao longo de 2 anos. Níveis de LDL-c < 70 mg/dL foram alcançados em 2 anos por 21% e 34% nas coortes com níveis iniciais de LDL-c ≥ 100 mg/dL e 70-99 mg/dL, respectivamente. Ao final do estudo, ezetimiba foi adicionada a apenas 5,3% dos pacientes.^28^

Já no estudo *Prospective Urban Rural Epidemiology* (PURE), fica claro o impacto de condições socioeconômicas na taxa de uso de estatinas. Entre participantes de prevenção secundária, estatinas foram utilizadas em apenas 3,3% dos indivíduos em países de baixa renda, 4,3% nos de baixa-média renda, 17,6% nos de média-alta renda e em 66,5% nos países de alta renda.^29^ Em outro estudo, realizado entre 2013 e 2019, com mais de 116.000 indivíduos (mais de 9.000 com histórico de doença cardiovascular) de 41 países de baixa e média renda, o uso de estatina foi de apenas 8% entre indivíduos elegíveis em prevenção primária e de 22% entre os que estavam em prevenção secundária.^30^

Nos serviços brasileiros de saúde pública, geralmente há disponibilização apenas de sinvastatina, ficando a atorvastatina reservada ao alto custo e a ezetimiba disponibilizada em poucos centros de atendimento de cardiologia. Inibidores de PCSK9 ficam restritos ao custeio particular pelos pacientes. Esses fatores dificultam a associação medicamentosa hipolipemiante e o atingimento das metas de LDL-c na população geral.^31^ Além disso, frequentemente exames de controle, seja de segurança ou perfil lipídico, não são realizados rotineiramente,^32^ o que dificulta a prevenção eficaz dos raros eventos adversos que podem ocorrer e não valoriza as recomendações de diretrizes de se buscar metas lipídicas, resultando em prejuízo à qualidade do cuidado.^33^

Portanto, apesar do uso de estatinas ter demonstrado eficácia e segurança há mais de 30 anos, inclusive propiciando redução de morte cardiovascular em estudos de prevenção primária e secundária, sua utilização ainda é abaixo da esperada. Além disso, o controle da colesterolemia, com atingimento de metas preconizadas pelas diretrizes, é insatisfatório em diferentes regiões do mundo. Sendo o Brasil um país de tamanho continental, com distribuição de renda altamente desigual entre as regiões, torna-se um grande desafio de saúde pública elevar as taxas de uso de hipolipemiantes para pessoas elegíveis e, consequentemente, aumentar a proporção de indivíduos que atingem as metas lipídicas. Ainda assim, o enfrentamento eficaz dessa situação deve ser perseguido para reduzir o impacto da doença cardiovascular na sociedade.

## 3. Diagnóstico

### 3.1. Avaliação Laboratorial dos Parâmetros Lipídicos e das Apolipoproteínas

#### 3.1.1. Fases Pré-Analítica e Analítica

Os lípides circulam na corrente sanguínea ligados a proteínas específicas, formando complexos denominados lipoproteínas. A acurácia na determinação das lipoproteínas depende principalmente de duas fases do processo laboratorial: a fase pré-analítica, relacionada a procedimentos de coleta, orientações aos pacientes, transporte da amostra, preparo da amostra ou a fatores intrínsecos do indivíduo, como estilo de vida, uso de medicações e doenças associadas; e a analítica, relacionada aos métodos e procedimentos utilizados pelos laboratórios.^34 , 35^

##### 3.1.1.1 Fase Pré-Analítica

Nesta fase, encontram-se todos os procedimentos realizados antes da amostra do paciente ser processada pelos equipamentos nos laboratórios.

- **Variação biológica:** as lipoproteínas podem sofrer alterações ao longo do tempo, caracterizadas como variação biológica intraindividual.^36^
- **Uso do torniquete na punção venosa:** após 1 minuto de torniquete, pode haver hemoconcentração com consequente alteração no perfil lipídico. Visando minimizar o "efeito torniquete", este deve ser desfeito tão logo a agulha penetre na veia.^37^
- **Condições de preparo do paciente para a coleta:** no preparo do paciente para a realização das dosagens do perfil lipídico, recomenda-se manter o estado metabólico estável e a dieta habitual. Condições de transgressão da dieta habitual (ingestão de álcool e dieta rica em gordura) assim como atividade física extenuante, na véspera do exame, podem alterar temporariamente o perfil lipídico. Diferentes diretrizes sinalizam que o jejum não é necessário para a testagem lipídica, pelo menos em uma avaliação inicial. A medida de triglicérides (TG) é a que mais pode sofrer alteração se realizada sem jejum. A elevação de TG por sua vez pode interferir na precisão do cálculo do LDL-c pela equação de Friedewald, mas esse problema pode ser mitigado pelo uso da fórmula de Martin/Hopkins.^38^

Os laboratórios devem adequar seus procedimentos, incluindo a flexibilização do tempo de jejum, respeitando sempre a orientação do médico solicitante. O laboratório deve informar no laudo se o exame foi realizado sem jejum ou com jejum de 12 horas, de acordo com o critério do médico solicitante. Em algumas situações clínicas específicas (exemplo: hiperlipidemia genética familiar), nas quais a concentração de TG encontra-se muito elevada (> 440 mg/dL), uma nova coleta de amostra para o perfil lipídico deve ser solicitada pelo médico ao paciente, com jejum de 12 horas. O médico deve avaliar o resultado do perfil lipídico do paciente, de acordo com a indicação do exame, o estado metabólico e a estratificação de risco.^39^

**Table:** 

Recomendação	Força da recomendação	Certeza da evidência
Recomenda-se coletar a amostra para a realização do perfil lipídico idealmente em condição metabólica estável.	FORTE	MODERADA
Para a avaliação inicial, é aceitável obter a amostra sem jejum, em particular em populações selecionadas, como crianças e idosos.	FORTE	MODERADA
Se os triglicérides estiverem elevados (> 440 mg/dL) em amostra sem jejum, recomenda-se nova coleta em jejum de 12 horas, de acordo com critério do médico solicitante.	FORTE	MODERADA

##### 3.1.1.2. Fase Analítica

Existem vários métodos disponíveis e utilizados na rotina dos laboratórios clínicos e outros restritos à pesquisa, sendo pouco utilizados na prática clínica, seja por baixa capacidade operacional em grandes rotinas e/ou por custo elevado.

###### 3.1.1.2.1. Métodos Restritos à Pesquisa

- **Ultracentrifugação:** ^40^ método de referência para a separação das diferentes lipoproteínas, baseando-se na propriedade de flutuação das partículas em relação às suas densidades de equilíbrio em campo gravitacional intenso. Por ultracentrifugação, é possível separar grande parte das lipoproteínas: LDL, lipoproteína de densidade intermediária (IDL), Lp(a), HDL e VLDL, além dos quilomícrons. O método, apesar de todas essas virtudes, não é adequado à rotina laboratorial, por ser muito caro e moroso, sendo restrito a protocolos de pesquisa.
- **Espectroscopia por ressonância magnética:** a expressão "contagem de partículas aterogênicas por espectroscopia de ressonância magnética" refere-se à quantificação do número de partículas lipoproteicas potencialmente aterogênicas (principalmente LDL, mas também VLDL, IDL e Lp(a)) utilizando-se espectroscopia de ressonância magnética nuclear (RMN ou NMR, do inglês nuclear magnetic resonance).^41 - 43^ Este método é reconhecido e validado na literatura médica para avaliação do risco cardiovascular, pois a concentração de partículas aterogênicas (LDL pequena e densa) apresenta associação mais forte com desfechos cardiovasculares do que a simples determinação do LDL-c. A técnica de RMN permite a quantificação direta do número de partículas lipoproteicas no plasma, baseando-se nas propriedades físicas e químicas dos lipídios e apolipoproteínas presentes nas partículas. O método mais amplamente utilizado clinicamente é a espectroscopia de RMN de próton (1H), que analisa sinais específicos dos grupos metila e metileno dos lipídios, permitindo a determinação do número e do tamanho das subclasses de lipoproteínas, incluindo LDL, VLDL e HDL.
- **Espectrometria de massas (MS):** a MS tem um papel crescente de destaque na avaliação da dislipidemia, indo além da quantificação tradicional de CT, LDL, HDL e TG. As diferentes abordagens baseadas em MS, especialmente as técnicas de lipidômica, permitem a caracterização detalhada do perfil lipídico plasmático e das subclasses de lipoproteínas, fornecendo informações sobre a composição da estrutura molecular de lipídios e apolipoproteínas em diferentes frações lipoproteicas.^44 - 46^ No contexto do diagnóstico, a MS tem sido utilizada para identificar biomarcadores lipídicos associados à hipercolesterolemia, como diferentes espécies de esfingolipídios (ceramidas) e sulfato de colesterol, que apresentam valor discriminatório elevado tanto para o diagnóstico quanto para a estratificação de risco cardiovascular.^44^ Além disso, a MS permite a análise simultânea de centenas a milhares de espécies lipídicas, com alta especificidade, reprodutibilidade e robustez, mesmo em estudos de larga escala, o que é fundamental para aplicações em medicina de precisão e epidemiologia.^45^ A capacidade de quantificar proteínas reguladoras, como PCSK9, em níveis muito baixos, também amplia o potencial da MS em estudos de metabolismo lipídico e resposta a terapias específicas.46 Em resumo, a MS, por meio de abordagens de lipidômica e proteômica, oferece uma avaliação aprofundada e precisa da dislipidemia, com potencial para identificar novos biomarcadores, elucidar mecanismos patogênicos e aprimorar a estratificação de risco cardiovascular, complementando e expandindo as metodologias laboratoriais convencionais.^44 - 46^ Entre os limitadores para que a técnica ganhe espaço na rotina dos laboratórios e na prática clínica, destacam-se: custo muito elevado dos equipamentos, validação e padronização dos métodos (estes desenvolvidos in house), restrição no número de profissionais qualificados para operacionalizar essa técnica nos laboratórios e, por fim, baixo grau de automação e integração laboratorial.^47^

###### 3.1.1.2.2. Métodos Convencionais – Rotina Laboratorial

####### 3.1.1.2.2.1. Métodos Enzimáticos Colorimétricos

São os métodos mais utilizados nos dias de hoje em laboratórios clínicos para a determinação do CT, do HDL-c e dos TG. Para o CT e os TG, os diversos conjuntos diagnósticos comerciais apresentam boa correlação e baixo coeficiente de variação (CV) entre eles, podendo-se comparar os resultados de diferentes laboratórios em uma mesma amostra. Já para o HDL-c, podem-se encontrar variações de até 15% entre os métodos disponíveis. Esses métodos constituem a melhor opção por apresentarem boa sensibilidade e especificidade, simplicidade operacional, baixo custo e possibilidade de automação em laboratórios clínicos. Para mensuração do LDL-c, existem métodos diretos colorimétricos que apresentam grande variação entre os diferentes conjuntos diagnósticos e não são utilizados na prática clínica. Para mensuração deste parâmetro o uso das fórmulas é o mais utilizado na prática clínico/laboratorial.

####### 3.1.1.2.2.2. Point of Care Testing

O chamado *point of care testing* (POCT) diz respeito a termo consagrado na literatura mundial para os pequenos dispositivos que realizam exames à beira do leito. Para avaliação do perfil lipídico com esses dispositivos, utilizamos amostras de sangue total, provenientes de coleta da punção capilar. Atualmente, existem muitos equipamentos portáteis disponíveis e aprovados para serem utilizados na avaliação das dislipidemias. Dependendo do aparelho, são avaliados os seguintes exames: CT, HDL-c, TG e até mesmo a mensuração do LDL-c de forma direta.^48 , 49^

O grande benefício dessa tecnologia é permitir que o paciente fique próximo do local de execução dos testes, com o resultado sendo disponibilizado de imediato, principalmente para os tratamentos intensivos e de Atenção Primária à Saúde. Um ponto de atenção é que existem diferentes metodologias abarcadas nesses pequenos equipamentos, fabricados por diferentes empresas. A validação de um dispositivo destes é fundamental para colocá-lo em uso e realizar exames para os pacientes. Essa validação deve ser feita comparando os resultados com amostras encaminhadas a um laboratório clínico, que tenha seus processos monitorados (laboratório acreditado).

Depois da validação para a utilização na rotina diária, é necessária a execução dos controles de qualidade que garantem uma maior segurança para a execução dos testes. A validação e esses controles estão previstos na Resolução da Diretoria Colegiada (RDC, nº 978, de 06/06/2025) da Agência Nacional de Vigilância Sanitária^50^ que permite a execução de testes que utilizam amostras de punção capilar em consultórios isolados e em serviços itinerantes (por exemplo, campanhas populacionais).

Apesar do avanço tecnológico, essas tecnologias ainda apresentam maior variação analítica quando comparadas com os testes realizados em amostras de soro em laboratórios clínicos. Outro ponto de atenção é que a execução desses testes requer treinamento do operador e uma avaliação criteriosa de todos os processos envolvidos na sua execução. Alguns fatores podem contribuir diretamente na precisão dos resultados, entre eles: temperatura ambiente, umidade relativa do ar e o volume da amostra de sangue total inserida no equipamento.^51^

Avaliando as alternativas no mercado para implantação do perfil lipídico na plataforma POCT, é vantajoso utilizar o equipamento que possua a análise completa do perfil lipídico, incluindo o HDL-c, viabilizando o cálculo do não-HDL-c. Este parâmetro é importante porque permite avaliar as lipoproteínas aterogênicas e o risco de DCV.

Apesar do custo do teste POCT ainda ser relevante, as vantagens para o perfil lipídico nesse sistema são várias, como nas situações de triagem da HF, em programas de saúde para avaliação de funcionários nas empresas, quando é necessário obter amostras de comunidades isoladas, em crianças e idosos com dificuldade para a punção venosa, entre outras ocorrências de risco iminente.^52^

####### 3.1.1.2.2.3. Cálculo do Colesterol da Lipoproteína de Baixa Densidade

O cálculo pela fórmula de Friedewald foi amplamente usado por anos, contudo, apresenta limitações na estimativa do valor de LDL-c. Martin *et al* .^53^ sugeriram outro método para estimar os valores de LDL-c, utilizando como referência a ultracentrifugação, e, por meio de cálculos estatísticos, definiram diferentes divisores para o valor de TG, que permitem estimar com maior fidedignidade os valores de VLDL-c. Para obter esses divisores, são necessárias as concentrações do não-HDL-c e do TG da amostra do paciente. Com esse novo divisor (x) aplica-se a fórmula: LDL-c = CT – HDL-c – TG/x, onde x varia de 3,1 a 11,9.

Outras fórmulas foram e continuam sendo descritas na literatura para calcular o valor do LDL-c. Em publicação recente, Samuel *et al* . avaliaram 23 fórmulas descritas na literatura, e a fórmula de Martin/Hopkins apresentou um melhor desempenho comparativo.^38^

Vale ressaltar que todas as fórmulas apresentam perda de acurácia com valores elevados de TG, principalmente com valores superiores a 800 mg/dL.

**Table:** 

Recomendação	Força da recomendação	Certeza da evidência
Recomenda-se a favor do uso da equação de Martin/Hopkins para cálculo de LDL-c para todos os indivíduos.	FORTE	MODERADA
Quando os valores de triglicérides estão acima de 800 mg/dL, os resultados de LDL-c pela fórmula de Martin podem estar subestimados. Recomenda-se a avaliação do não-HDL-c.	FORTE	MODERADA

LDL-c: colesterol da lipoproteína de baixa densidade; não-HDL-c: colesterol não HDL.

####### 3.1.1.2.2.4. Medida do Colesterol Não HDL (não-HDL-c)

O não-HDL-c representa a fração do colesterol nas lipoproteínas plasmáticas, exceto a HDL, e é estimado subtraindo-se o valor de HDL-c do valor de CT: não-HDL-c = CT - HDL-c. A utilização do não-HDL-c tem a finalidade de estimar a quantidade de lipoproteínas aterogênicas circulantes no plasma, especialmente em indivíduos com TG elevados.^54^

**Table:** 

Recomendação	Força da recomendação	Certeza da evidência
Tanto o LDL-c quanto o não-HDL-c apresentam grande utilidade na avaliação do risco cardiovascular e como metas terapêuticas. O colesterol não-HDL é particularmente útil na estimativa da quantidade de lipoproteínas aterogênicas circulantes em indivíduos com triglicérides > 150 mg/dL.	FORTE	ALTA

LDL-c: colesterol da lipoproteína de baixa densidade; não-HDL-c: colesterol não HDL.

####### 3.1.1.2.2.5. Medida da Apolipoproteína B (ApoB)

A ApoB é a principal proteína estrutural presente nas lipoproteínas aterogênicas, atuando como ligante para o receptor do LDL. Sua dosagem fornece uma estimativa direta da concentração total de partículas lipídicas aterogênicas plasmáticas, já que existe uma única molécula de ApoB em cada lipoproteína aterogênica: LDL, VLDL, IDL e Lp(a). Na população geral, as concentrações de LDL-c e ApoB estão altamente correlacionadas, e, portanto, em geral fornecem informações semelhantes sobre o risco de DCVA. Por outro lado, em indivíduos com diabetes, obesidade ou síndrome metabólica, frequentemente associados a níveis elevados de TG, a mensuração isolada do LDL-c pode subestimar a concentração total de lipoproteínas contendo ApoB.^55^ A explicação para essa situação é que na presença de níveis elevados de TG, parte do colesterol nas partículas de LDL é substituída por TG, promovendo a produção de partículas pequenas e densas de LDL. Estas partículas, mais aterogênicas, tornam a quantidade de colesterol no LDL um reflexo não confiável do número de partículas de LDL.

Neste contexto, estima-se que pode haver discordância entre os níveis medidos de LDL-c e de ApoB em cerca de 20% desses pacientes.^56^ Portanto, na presença de TG elevados, a estimativa da concentração de partículas aterogênicas pode trazer melhor acurácia na avaliação do risco CV. Nesse cenário, o colesterol não-HDL (indiretamente) e ApoB (diretamente) fornecem uma avaliação mais precisa. Como vimos acima, o não-HDL-c representa todo o colesterol carreado pelas lipoproteínas que contêm ApoB. Já a ApoB estima diretamente o número de partículas aterogênicas no plasma. Na comparação entre o não-HDL-c e a ApoB, estudos populacionais têm considerado que ambos são marcadores equivalentes de risco CV, e isso se aplica à maioria das pessoas. Análise de eventos CV no Biobank do Reino Unido,^57^ e metanálise de estudos de coorte prospectivos de pacientes sob risco de DCV ou com DCV, mostraram uma capacidade similar de avaliação de risco com ambos os marcadores.^58^ Por outro lado, publicações mais recentes apontam que um subgrupo de indivíduos, estimado entre 8 e 23%, apresenta discordância entre os níveis de ApoB e de não-HDL-c, sendo a ApoB um melhor preditor de risco de calcificação coronariana e eventos CV.^59^

A ApoB também fornece avaliação mais precisa das lipoproteínas aterogênicas mediante LDL-c muito baixo. Em relação à dosagem da ApoB, é importante ressaltar que seus níveis não são significativamente alterados no estado pós-prandial em indivíduos com TG < 400 mg/dL.^60^

Uma limitação para o uso amplo da ApoB é que os seus limiares para iniciar ou intensificar terapia farmacológica não estão bem estabelecidos, quando comparados com níveis de LDL-c e não-HDL-c.

**Table:** 

Recomendação	Força da recomendação	Certeza da evidência
A medida da ApoB pode auxiliar na avaliação de risco cardiovascular e guiar a terapia em indivíduos com triglicérides > 150 mg/dL.	FORTE	MODERADA
O não-HDL-c é atualmente uma escolha mais prática porque pode ser facilmente calculado e não acarreta despesas adicionais para o paciente ou para o sistema de saúde.	FORTE	ALTA

ApoB: Apolipoproteína B; não-HDL-c: colesterol não HDL.

####### 3.1.1.2.2.6. Lipoproteína(a)

A Lp(a) é uma partícula semelhante à LDL, na qual a ApoB é covalentemente ligada a uma molécula denominada apolipoproteína(a). Além dos efeitos pró-aterogênicos, a Lp(a) tem efeitos pró-inflamatórios, provavelmente relacionados a sua carga de fosfolipídios oxidados, e a apolipoproteína(a) se assemelha ao plasminogênio, o que levanta a possibilidade de efeitos pró-trombóticos.

As concentrações plasmáticas de Lp(a) não são influenciadas por dieta, idade, sexo, estado de jejum ou estilo de vida, sendo amplamente (> 90%) determinadas geneticamente. Os valores individuais são geralmente estáveis ao longo da vida e, portanto, medidas repetidas não são necessárias para avaliação de risco. A determinação da Lp(a) é desafiadora, pois existe variação entre os métodos analíticos, em parte relacionada à estrutura da apolipoproteína(a), que pode ser amplamente variável em tamanho.

As concentrações séricas da Lp(a) devem ser medidas preferencialmente utilizando um método em que o efeito do tamanho da isoforma seja minimizado. A recomendação, portanto, é que seja feita a medição da concentração de partículas circulantes (em nmol/L). Se não disponível, é aceitável medir a concentração da massa de Lp(a) (em mg/dL).^61^ Esta diretriz não recomenda a conversão de unidades de massa em unidades molares, pela baixa acurácia.

Uma concentração de Lp(a) > 50 mg/dL (ou > 125 nmol/L) é encontrada em aproximadamente 20% dos indivíduos de ascendência europeia e sul-asiática, em 40% dos indivíduos afro-americanos e em menos de 10% dos indivíduos do leste asiático.^62^ No entanto, estudos em amostras maiores de diferentes etnias são necessários. As concentrações de Lp(a) geralmente são 5 a 10% maiores em mulheres do que em homens. Em homens, Lp(a) permanece relativamente constante, enquanto em mulheres os níveis tendem a apresentar um discreto aumento no climatério.^63^

Estudos populacionais têm demonstrado, de maneira linear, que quanto mais elevados os níveis de Lp(a), maior é o risco de IAM e calcificação da valva aórtica. Níveis elevados de Lp(a) também aumentam o risco de DCVA recorrente, de maneira dose-dependente. Nesse contexto, é importante ressaltar que Lp(a) elevada é um fator de risco mesmo em indivíduos com níveis baixos de LDL-c.^64^

Indivíduos com elevações extremas da Lp(a) (≥ 180 mg/dL ou 390 nmol/L) demonstraram estar em risco CV marcadamente elevado, com uma taxa de eventos semelhante à de outras dislipidemias genéticas para as quais a triagem familiar é recomendada.^65^

Estudos de randomização mendeliana mostraram claramente que variantes genéticas no *locus* LPA, que regulam exclusivamente os níveis de Lp(a), estão fortemente associadas ao risco de doença coronária, sugerindo uma associação causal entre Lp(a) e DCVA. Estudos genéticos também sugerem que grandes reduções dos níveis de Lp(a) (> 60%) sejam necessárias para redução de eventos CV.^66^ Nesse contexto, agentes em investigação mais recentes, como oligonucleotídeos antisenso e pequenos RNAs interferentes, que reduzem os níveis séricos em até 90%, estão atualmente sendo avaliados em ensaios clínicos.^67^

Deve-se ressaltar também que os medicamentos hipolipemiantes comumente usados, isto é, estatinas e ezetimiba, não reduzem os níveis da Lp(a). As terapias medicamentosas disponíveis que levam à redução leve a moderada (20 a 30%) da Lp(a) incluem as terapias anti-PCSK9. Evidências preliminares sugerem que o tratamento com terapias anti-PCSK9 após SCA em pacientes com Lp(a) muito elevada pode reduzir eventos CV, independentemente da redução de LDL-c; no entanto, tais achados não são suficientes para rotinizar o seu uso em pacientes com níveis elevados de Lp(a).

Aférese é um método que não é utilizado no nosso meio, pois é oneroso, invasivo e demanda tempo para o paciente e a equipe médica.

Em resumo, a Lp(a) é um marcador de risco prevalente para a DCVA que ainda não é rotineiramente avaliado. A associação com eventos CV incidentes e recorrentes e o potencial para melhorar a estratificação de risco CV justificam a triagem universal para identificar indivíduos com níveis muito elevados. Embora esses dados apoiem o papel potencial da Lp(a) como alvo de tratamento no futuro, ainda não temos resultados de ensaios clínicos randomizados com terapias que reduzem especificamente a Lp(a).

Nesse contexto, no cenário da prevenção primária, recomendamos para os pacientes com Lp(a) ≥ 50 mg/dL (ou ≥ 125 nmol/L) aconselhamento mais precoce e intensivo sobre modificação do estilo de vida e gerenciamento de outros fatores de risco. Também podem ser considerados estudos de imagem vascular para detectar precocemente aterosclerose subclínica em indivíduos selecionados, assim como a introdução mais precoce de estatina ou outra terapia hipolipemiante, especialmente em indivíduos com risco intermediário e/ou indivíduos de baixo risco com elevações moderadas de LDL-c. No cenário da prevenção secundária, a presença de um nível elevado de Lp(a) é fortemente preditiva de eventos recorrentes e sugere a necessidade de intensificação da terapia de redução de LDL-c e controle mais estreito dos demais fatores de risco. Diante dos níveis aumentados de Lp(a) no paciente índice — cuja concentração é majoritariamente determinada geneticamente — recomendamos a realização de uma investigação em cascata nos familiares, com o objetivo de identificar outros possíveis portadores e avaliar o risco cardiovascular associado.

**Table:** 

Recomendação	Força da recomendação	Certeza da evidência
Na população geral, é recomendada a dosagem de Lp(a) uma vez na vida, quando disponível, para auxiliar na estratificação de risco e/ou manejo terapêutico.	FORTE	MODERADA
Em populações específicas, como aquelas com DAC precoce, estenose aórtica, HF, história familiar de DCVA precoce ou de Lp(a) aumentada, é recomendada a dosagem de Lp(a) uma vez na vida, quando disponível, para auxiliar na estratificação de risco e/ou manejo terapêutico.	FORTE	ALTA
Recomenda-se como método preferencial para medir a Lp(a) um ensaio que seja independente da isoforma, ou seja, que meça o número de partículas por litro (nmol/L). A dosagem por unidade de massa (mg/dL) deve ser evitada. As fórmulas de conversão não corrigem a diferença entre os métodos e não são recomendadas.	FORTE	ALTA
A medida da Lp(a) por ensaio não independente da isoforma, ou seja, que mede unidades de massa (mg/dL), pode ser usada quando for a única disponível.	FORTE	ALTA
Em indivíduos com níveis aumentados de Lp(a) (≥ 50 mg/dL ou ≥ 125 nmol/L), cuja concentração é predominantemente determinada geneticamente, é recomendada a investigação em cascata nos familiares para auxiliar na identificação de outros possíveis portadores e na avaliação precoce do risco cardiovascular.	FORTE	ALTA

DAC: doença arterial coronariana; HF: hipercolesterolemia familiar; DCVA: Doença Cardiovascular Aterosclerótica; Lp(a): Lipoproteína(a).

####### 3.1.1.2.2.7. Valores Referenciais do Perfil Lipídico

Esta atualização sugere que os valores referenciais e de alvo terapêutico do perfil lipídico (adultos > 20 anos) sejam apresentados de acordo com o estado metabólico que antecede a coleta da amostra, sem jejum e com jejum de 12 horas. Assim, os valores referenciais e de alvo terapêutico, obtidos de acordo com a avaliação de risco cardiovascular estimado pelo médico solicitante, são apresentados na Tabela 3.1 e devem constar dos laudos laboratoriais, em todo o território nacional, para que se obtenha uniformidade no tratamento das dislipidemias. Como para o LDL-c e o não-HDL-c os valores referenciais variam de acordo com o risco cardiovascular estimado, nesta atualização são sugeridos valores de alvo terapêutico para estas variáveis de acordo com a categoria de risco. Os parâmetros CT, HDL-c, LDL-c e não-HDL-c não sofrem influência do estado alimentar. Para os TG sem jejum, o valor desejável é < 175 mg/dL. O laboratório deve informar no laudo o tempo de jejum para as seguintes situações: sem jejum ou com jejum de 12 horas, de acordo com o critério do médico solicitante. Devem ser informados no laudo os valores referenciais para TG com e sem jejum.

**Tabela 3.1:** Valores referenciais ou alvos terapêuticos do perfil lipídico, de apolipoproteína B e de lipoproteína(a)

Parâmetro	Em jejum (12 h)	Sem jejum	Categoria de risco
Colesterol Total	< 190 mg/dL	< 190 mg/dL	
Triglicérides	< 150 mg/dL	< 175 mg/dL	
HDL-c	> 40 mg/dL	> 40 mg/dL	
LDL-c	< 115 mg/dL	< 115 mg/dL	Baixo
	< 100 mg/dL	< 100 mg/dL	Intermediário
	< 70 mg/dL	< 70 mg/dL	Alto
	< 50 mg/dL	< 50 mg/dL	Muito alto
	< 40 mg/dL	< 40 mg/dL	Extremo
Não-HDL-c	< 145 mg/dL	< 145 mg/dL	Baixo
	< 130 mg/dL	< 130 mg/dL	Intermediário
	< 100 mg/dL	< 100 mg/dL	Alto
	< 80 mg/dL	< 80 mg/dL	Muito alto
	< 70 mg/dL	< 70 mg/dL	Extremo
Apolipoproteína B	< 100 mg/dL	< 100 mg/dL	Baixo
	< 90 mg/dL	< 90 mg/dL	Intermediário
	< 70 mg/dL	< 70 mg/dL	Alto
	< 55 mg/dL	< 55 mg/dL	Muito alto
	< 45 mg/dL	< 45 mg/dL	Extremo
Lipoproteína(a)	< 75 nmol/L (< 30 mg/dL)	< 75 nmol/L (< 30 mg/dL)	Valor usado para **estratificação de risco cardiovascular** . Sem meta terapêutica definida.

O laboratório deve informar no laudo o tempo de jejum (sem jejum ou com jejum de 12 horas), conforme critério do médico solicitante. Os valores referenciais e de alvo terapêutico devem ser apresentados nos laudos laboratoriais para uniformizar o tratamento das dislipidemias. Para lipoproteína(a), recomenda-se o uso de ensaio independente da isoforma, expressando o resultado em nmol/L. A unidade mg/dL pode ser utilizada apenas quando não houver alternativa, mas não é o método preferencial. HDL-c: colesterol da lipoproteína de alta densidade; LDL-c: colesterol da lipoproteína de baixa densidade; Não-HDL-c: colesterol não HDL.

### 3.2. Diagnóstico Genético das Dislipidemias

#### 3.2.1. Hipercolesterolemias de Base Genética

##### 3.2.1.1. Considerações para Solicitar um Teste Genético

O teste genético permite identificar o tipo de variante e sua gravidade (LDLR-defeituoso *versus* LDLR-nulo), o que se associa com o grau de hipercolesterolemia e o risco de desenvolvimento de doença arterial coronária (DAC), incluindo DAC prematura; portadores de variantes nulas no gene *LDLR* apresentam um fenótipo mais grave,^68 , 69^ enquanto as variantes não-nulas no *LDLR* , bem como as variantes patogênicas nos genes *APOB* e *PCSK9* , geralmente têm um fenótipo mais brando.^69^ As definições genótipo-positivo e fenótipo-positivo para a HF devem ser empregadas para identificar e tratar todo o espectro de pacientes com HF com variante patogênica identificada (genótipo-positivo), aqueles sem (fenótipo-positivo, genótipo-negativo) e aqueles que não fizeram teste genético.

A confirmação genética tem um efeito positivo no início do tratamento hipolipemiante, na aderência e na redução do LDL-c;^70 - 72^ facilita o rastreamento em cascata de familiares para HF, pois, como condição genética autossômica semidominante, o rastreamento de familiares de um indivíduo afetado (caso-índice ou probando) "sob risco" pode ser altamente efetivo na identificação de indivíduos adicionais com HF que irão requerer tratamento;^71 , 73^ identifica novos pacientes com HF;^74 - 77^ e contribui para a prevenção de DAC, infarto do miocárdio e morte.^78 - 80^ A cascata genética pode reduzir a idade com que os parentes com HF são diagnosticados, comparados aos casos-índice,^81^ e reduzir o CT e o LDL-c dos parentes afetados.^71^ Diretrizes de HF recomendam que, quando uma variante patogênica for encontrada em um caso-índice, essa variante deve ser utilizada para rastreio dos familiares afetados, reduzindo o custo do rastreamento.^82 , 83^

O teste genético tem papel importante no aconselhamento genético pré- e pós-teste. O teste do probando permite informação precisa e acurada do risco durante o aconselhamento e informa a abordagem correta da cascata genética familiar. O teste genético permite a discriminação em nível molecular entre indivíduos com hipercolesterolemia unialélica semidominante, bialélica monogênica semidominante (antigo homozigoto simples), bialélica monogênica semidominante com duas variantes distintas (antigo heterozigoto composto), bialélica digênica semidominante (antigo heterozigoto duplo) e bialélica recessiva com duas cópias idênticas ou uma cópia de duas variantes distintas (antiga autossômica recessiva).^84^

Especificamente para aqueles com variantes bialélicas digênicas, os pais do probando deveriam ser testados para as variantes encontradas, para determinar qual variante foi herdada da mãe e qual foi herdada do pai, e/ou se uma das variantes foi uma variante *de novo* , que, embora rara, pode ocorrer. Todos os parentes maternos e paternos com HF deveriam ser testados para detecção da variante em cada lado da família. Sem essa forma de rastreio, os probandos com HF com duas variantes distintas podem ser classificados erroneamente como heterozigotos graves, com implicações negativas para os familiares sob risco, por desconhecerem que ambos os lados da família estão sob risco pela presença de duas variantes patogênicas no probando.^85^

A *American Society of Human Genetics* recomenda o teste genético em crianças e adolescentes, quando existe uma intervenção clínica a ser feita.^86^ Na HF heterozigótica, o tratamento com estatinas deve ser iniciado entre 8 e 10 anos, e as intervenções para um estilo de vida saudável, ainda mais precocemente. Nas crianças com HF bialélica, tratamento de alta intensidade deve ser implementado ao diagnóstico.^87^ Se não tratadas, crianças com HF estarão sob risco aumentado de eventos coronários quando adultos devido à exposição cumulativa aos níveis elevados de LDL-c, sendo que muitos terão um evento cardiovascular quando jovens. Crianças com HF que iniciam o tratamento com estatinas precocemente têm menores taxas de eventos que seus pais.^88^

Recomenda-se incluir no painel genético para HF genes nos quais variantes patogênicas causadoras de HF foram identificadas ( *LDLR,* *APOB* e *PCSK9* ) e as variantes bialélicas na *LDLRAP1* , responsáveis pela forma autossômica recessiva, além dos genes *ABCG5/ABCG8* causadores da sitosterolemia e o gene *LIPA* , causador de deficiência da lipase ácida lisossomal (LALD), fenocópias da HF,^84^ que possuem abordagens terapêuticas distintas. O teste genético permite inferir sobre formas poligênicas, quando uma variante patogênica não é encontrada (genótipo-negativo) em um indivíduo com fenótipo-positivo,^84^ onde a cascata genética não é custo-efetiva.

**Table:** 

Recomendação	Força da recomendação	Certeza da evidência
**Probando (caso-índice) –** O teste genético para HF deve ser oferecido a indivíduos de qualquer idade nos quais exista um forte indício clínico de HF, com base na história clínica e/ou familiar do paciente. Observação – esse índice de suspeita inclui: crianças com níveis persistentes * de LDL-c ≥ 160 mg/dL ou adultos com níveis persistentes * de LDL-c ≥ 190 mg/dL sem uma causa secundária aparente de hipercolesterolemia † e com pelo menos um familiar de primeiro grau igualmente afetado, ou com doença arterial coronária prematura ‡ , ou quando o histórico familiar não está disponível (por exemplo, adoção); crianças com níveis persistentes * de LDL-c ≥ 190 mg/dL ou adultos com níveis persistentes * de LDL-c ≥ 250 mg/dL sem uma causa secundária aparente de hipercolesterolemia † , mesmo na ausência de histórico familiar positivo.	FORTE	MODERADA
**Familiares sob risco –** O teste genético em cascata para a(s) variante(s) específica(s) identificada(s) no probando com HF (teste da variante familiar conhecida) deve ser oferecido a todos os parentes de primeiro grau. Se os parentes de primeiro grau não estiverem disponíveis ou não desejarem realizar o teste, o teste da variante familiar conhecida deve ser oferecido aos parentes de segundo grau. O teste genético em cascata deve continuar por toda a família extensa até que todos os indivíduos em risco tenham sido testados e todos os parentes com HF tenham sido identificados.	FORTE	MODERADA

HF: hipercolesterolemia familiar.

* Persistente: níveis elevados de LDL-c confirmados em pelo menos duas dosagens separadas;

† Causas secundárias de hipercolesterolemia incluem hipotireoidismo, síndrome nefrótica, doença hepática colestática e certos medicamentos;

‡ Doença arterial coronária prematura: < 55 anos em homens e < 65 anos em mulheres. HF: hipercolesterolemia familiar; LDL-c: colesterol da lipoproteína de baixa densidade. Adaptado de Sturm et al.^89^

**Table:** 

Recomendação	Força da recomendação	Certeza da evidência
Pode ser considerado teste genético para HF em adultos sem níveis de LDL-c disponíveis antes do tratamento, mas com histórico pessoal de doença arterial coronária prematura ‡ e histórico familiar de hipercolesterolemia e doença arterial coronária prematura ‡	CONDICIONAL	MODERADA
Pode ser considerado teste genético em adultos com níveis persistentes * de LDL-c ≥ 160 mg/dL (sem uma causa secundária aparente de hipercolesterolemia † ) no contexto de histórico familiar de hipercolesterolemia e histórico pessoal ou familiar de doença arterial coronária prematura ‡ .	CONDICIONAL	MODERADA

* Persistente: níveis elevados de LDL-c confirmados em pelo menos duas dosagens separadas;

† Causas secundárias de hipercolesterolemia incluem hipotireoidismo, síndrome nefrótica, doença hepática colestática e certos medicamentos;

‡ Doença arterial coronária prematura: < 55 anos em homens e < 65 anos em mulheres. LDL-c: colesterol da lipoproteína de baixa densidade.

### 3.3. Diagnóstico das Hipertrigliceridemias

#### 3.3.1. Síndrome da Quilomicronemia Familiar

##### 3.3.1.1. Definição

Os níveis de TG plasmáticos em jejum são normalmente inferiores a 150 mg/dL e, no estado pós-alimentar, são considerados normais valores inferiores a 175 mg/dL.^37^ Os TG podem estar aumentados devido a múltiplos fatores, incluindo, mas não se limitando à dieta, obesidade e estados de resistência à insulina, como o diabetes ou uso de certos medicamentos, que são conhecidos por interagir com determinantes poligênicos.^90 , 91^ Níveis extremamente elevados de TG podem ocorrer em condições herdadas, como na síndrome da quilomicronemia familiar (SQF) (Tipo I de Fredrickson), na hiperlipidemia familiar combinada (Tipo IIa), na disbetalipoproteinemia ou hiperlipidemia remanescente (Tipo III), na hipertrigliceridemia familiar (Tipo IV), na quilomicronemia multifatorial (SQM) (Tipo V)^91 - 94^ e ainda nas lipodistrofias (LD) generalizadas e lipodistrofia parcial familiar.^95^

A SQF é um distúrbio autossômico recessivo que afeta aproximadamente 1 a 10 indivíduos por milhão e é mais frequentemente causada por variantes patogênicas ou provavelmente patogênicas bialélicas no gene LPL que codifica a enzima lipoproteína lipase ( *LPL* ). Variantes nos genes *GPIHBP1* ( *glycosylphosphatidylinositol anchored high-density lipoprotein binding protein 1* ), *LMF1* ( *lipase maturation fator 1* ), *APOA5* (apolipoproteína A5) e *APOC2* (apolipoproteína C2) também foram identificadas como causais para essa condição.^96 - 98^ Além disso, a SQF pode se apresentar com duas variantes distintas, bialélicas ou digênicas.

Caracteriza-se por um risco aumentado de pancreatite aguda recorrente.^99 , 100^

##### 3.3.1.2. Diagnóstico Clínico e Laboratorial da Quilomicronemia Familiar

As manifestações clínicas das formas monogênicas de quilomicronemia ocorrem, em geral, na infância ou no início da vida adulta, e são comuns atrasos no diagnóstico, fazendo com que este ocorra na vida adulta, quando as complicações já estão estabelecidas.^90^ Dores abdominais recorrentes (50%),^96^ episódios de pancreatite recorrente (50%),^101^ hepatoesplenomegalia,^102^ xantomas eruptivos (17-33%)^96^ e lipemia *retinalis* (30%) são observados em pacientes com níveis maiores de TG.^102^ Manifestações neurológicas, como fadiga, confusão mental, irritabilidade e déficits cognitivos ou *mental fog* , estão entre os sintomas mais comumente descritos entre os pacientes acometidos com SQF, comprometendo sua qualidade de vida.^102 , 103^

##### 3.3.1.3. Escores Diagnósticos

O escore de Moulin *et al* .^104^ utiliza como critério de seleção a presença de TG (> 885 mg/dL em jejum e fora da fase aguda) e pontua na presença de valores elevados de TG, afastadas causas secundárias ( Quadro 3.1 ). Esse escore foi testado em coortes de pacientes com confirmação genética de SQF e na SQM, e validado em outras coortes. Recomenda-se que os escores sejam utilizados na triagem para o teste genético ou quando este não é disponível.

**Quadro 3.1:** Escore de Moulin para suspeição da síndrome de quilomicronemia familiar^104^

Critério		Escore
Valores de TG em jejum > 885 mg/dL em pelo menos três dosagens consecutivas ( * )		+5
Valores de TG em jejum > 1.770 mg/dL em pelo menos uma ocasião		+1
Valores prévios de TG <177 mg/dL em pelo menos uma ocasião		-5
Ausência de fatores secundários † (exceto gestação ‡ e uso de etinilestradiol)		+2
História de pancreatite		+1
Dor abdominal recorrente inexplicada		+1
Sem história de hiperlipidemia familiar combinada		+1
Sem resposta à terapia hipolipemiante (redução de TG < 20%)		+1
Idade do início dos sintomas		
	< 40 anos	+1
	< 20 anos	+2
	< 10 anos	+3
**Escore SQF**		
	**Muito provável**	≥ **10**
	**Improvável**	≤ **9**
	**Muito improvável**	≤ **8**

* Medidas realizadas com pelo menos um mês de diferença;

† Uso de álcool, diabetes, síndrome metabólica, hipotireoidismo, corticoterapia, e uso de outras drogas;

‡ Se o diagnóstico for feito durante a gestação, uma segunda dosagem deve ser solicitada para confirmação diagnóstica no pós-parto. SQF: síndrome da quilomicronemia familiar; TG: triglicérides.

##### 3.3.1.4. Diagnóstico Diferencial

Em adultos, os principais diagnósticos diferenciais da SQF são a SQM^92^ (hiperlipoproteinemia tipo V de Fredrickson), que inclui variantes em heterozigose nos cinco genes canônicos para SQF ou alto escore poligênico, agravada por comorbidades ou causas secundárias de hipertrigliceridemia (HTG).^92^ Outro diagnóstico diferencial da SQF são as LD, caracterizadas pela perda seletiva de tecido adiposo e que podem cursar com HTG grave e pancreatite. As LD herdadas são distúrbios raros, que podem se manifestar ao nascimento (forma congênita generalizada) ou apresentar perda de gordura em fases mais tardias da vida. Nas formas parciais, a suspeita diagnóstica deve ser considerada na presença de HTG moderada a grave, associada à medida de prega cutânea da coxa < 22 mm em mulheres ou < 10 mm em homens, e/ou casos de diabetes com necessidade de uso de insulina subcutânea em doses diárias > 2 UI/kg.^95^

##### 3.3.1.5. Diagnóstico Genético

A SQF pode ser causada por variantes patogênicas ou provavelmente patogênicas bialélicas nos genes *LPL, GPIHBP1, LMF1* , *APOA5* ou *APOC2* . Pode também se apresentar na forma bialélica, com duas variantes distintas, ou ainda na forma digênica.^105^ Os painéis genéticos utilizados incluem os cinco genes canônicos para SQF, além de genes relacionados à LD, fibrose cística, pancreatites e o gene LIPA, responsável pela LALD.

##### 3.3.1.6. Atividade da Lipoproteína Lipase

A atividade da LPL está muito reduzida em pacientes com SQF que apresentam uma variante bialélica no gene *LPL* , ou naqueles com variantes bialélicas com perda de função nos demais genes canônicos para SQF, ou ainda naqueles com variantes digênicas.^106^ Habitualmente, a atividade dessa enzima está reduzida a menos de 25% nos pacientes com SQF.

##### 3.3.1.7. Outros Exames Diagnósticos

A quilomicronemia autoimune pode ser causada pela presença de anticorpos anti-GPIHBP1, caracterizada por HTG intermitente e associada a condições autoimunes prévias, incluindo presença de anticorpos antinucleares.^107^

**Table:** 

Recomendação	Força da recomendação	Certeza da evidência
Recomenda-se a utilização de escores clínicos para diagnóstico de SQF.	FORTE	ALTA
Em circunstâncias ideais, o teste genético é o método recomendado para confirmação diagnóstica de SQF.	FORTE	ALTA
O painel genético para SQF deve incluir o sequenciamento dos genes LPL, GPIHBP1, LMF1, APOA5 e APOC2.	FORTE	ALTA

SQF: Sindrome da Quilomicronemia Familiar.

## 4. Estratificação de Risco

### 4.1. Estratificação do Risco Cardiovascular

A estratificação do risco cardiovascular é a base para a tomada de decisão clínica na prevenção da doença cardiovascular, guiando diferentes condutas terapêuticas, incluindo o manejo do LDL-c. Adequar a intensidade do tratamento ao risco absoluto de eventos é fundamental para garantir os benefícios aos indivíduos de maior risco e evitar tratamentos excessivos ou desnecessários naqueles de risco mais baixo.

**Figura 4.1:**
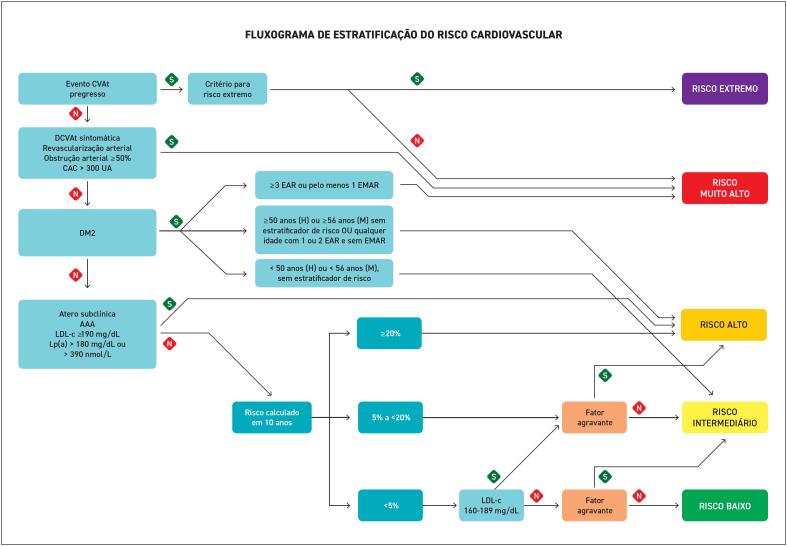
Estratificação do risco cardiovascular aterosclerótico.

Apesar do risco cardiovascular ser uma variável contínua, o estabelecimento de categorias de risco facilita a formulação de recomendações e a instituição do tratamento mais adequado ( Tabela 4.1 ). Dessa forma, esta diretriz recomenda que o risco cardiovascular aterosclerótico seja categorizado em baixo, intermediário, alto, muito alto e extremo ( Figura 4.1 ). Os indivíduos que já apresentaram um evento cardiovascular são os que têm risco mais elevado (risco muito alto ou extremo) ( Tabela 4.1 e 4.2 ). Na ausência de doença cardiovascular manifesta, a categoria de risco será determinada pela presença ou não de aterosclerose subclínica em exames de imagem e por inúmeros fatores de risco (tradicionais e agravantes). Em particular, indivíduos com diabetes mellitus (DM) merecem uma estratificação de risco diferenciada, devido ao alto poder aterogênico do DM. Da mesma forma, reconhecendo o papel da exposição cumulativa às lipoproteínas aterogênicas ao longo da vida na predisposição a eventos, esta diretriz recomenda que indivíduos com LDL-c ≥ 190 mg/dL sejam classificados como de risco alto, enquanto aqueles com LDL-c entre 160 e 189 mg/dL sejam considerados de risco intermediário. Para os adultos sem evento cardiovascular pregresso ou aterosclerose subclínica, sem DM e com LDL-c < 160 mg/dL, a categorização do risco cardiovascular aterosclerótico pode ser definida pelo uso de equações ou escores de risco.

**Tabela 4.1:** Categorias de risco

Categoria de risco	Definição
**Baixo**	Escore de risco calculado < 5% em 10 anos. Ausência de agravantes de risco. Ausência de DM2.
**Intermediário**	Escore de risco calculado 5 a < 20% em 10 anos, na ausência de agravantes de risco. Risco calculado baixo (< 5% em 10 anos) com presença de agravante de risco. DM2 com < 50 anos homem, < 56 anos mulher, sem EAR e sem EMAR. LDL-c > 160-189 mg/dL.
**Alto**	Escore de risco calculado ≥ 20% em 10 anos. Pacientes classificados como risco intermediário, com presença de fator agravante de risco. Portadores de aterosclerose subclínica: placa carotídea < 50%; CAC > 100 UA ou percentil > 75; placas ateroscleróticas na angiotomografia de coronárias < 50%; aneurisma de aorta abdominal. LDL-c ≥ 190 mg/dL. DM2 homem ≥ 50 anos, mulher ≥ 56 anos, 1 ou 2 EAR, sem EMAR. Lipoproteína(a) > 180 mg/dL (> 390 nmol/L).
**Muito alto**	Doença aterosclerótica significativa (coronária, cerebrovascular ou vascular periférica com obstrução ≥ 50%) ou evento aterosclerótico cardiovascular prévio manifesto. CAC > 300 UA. DM2 com EMAR ou 3 ≥ EAR.
**Extremo** *	Histórico de múltiplos eventos cardiovasculares ateroscleróticos maiores ou um evento cardiovascular aterosclerótico maior E com duas ou mais condições de alto risco.

* Risco extremo definido como histórico de múltiplos eventos cardiovasculares ateroscleróticos maiores ou 1 evento cardiovascular aterosclerótico maior e pelo menos 2 condições de alto risco. DM2: Diabetes Mellitus tipo 2; EAR: estratificadores de risco; EMAR: estratificadores de muito alto risco; UA: Unidades Agatston.

**Tabela 4.2:** Critérios para risco cardiovascular extremo

**Eventos cardiovasculares ateroscleróticos maiores**	Síndrome coronária aguda recente (nos últimos 12 meses) Histórico de infarto do miocárdio Histórico de AVC isquêmico Doença arterial periférica sintomática (histórico de claudicação com índice tornozelo-braquial < 0,85 ou revascularização ou amputação prévia)
**Condições de alto risco**	Idade ≥ 65 anos Hipercolesterolemia familiar Histórico de cirurgia de revascularização do miocárdio anterior ou intervenção coronária percutânea fora do(s) evento(s) cardiovascular(es) aterosclerótico(s) maior(es) DM Hipertensão arterial Doença renal crônica (TFGe 15–59 mL/min/1,73 m2) Tabagismo atual LDL-c persistentemente elevado (≥ 100 mg/dL) apesar de terapia com estatina em dose máxima tolerada e ezetimiba Evento agudo aterosclerótico com menos de 2 anos

AVC: acidente vascular cerebral; DM: diabetes mellitus; LDL-c: colesterol da lipoproteína de baixa densidade; TFGe: taxa de filtração glomerular estimada.

### 4.2. Escores de Risco Cardiovascular

Os escores de risco cardiovascular são utilizados para estimar risco de evento cardiovascular em determinado prazo de tempo. Embora muitos escores de risco cardiovascular tenham sido desenvolvidos em diferentes regiões do mundo, ainda não dispomos de um escore de risco derivado de dados da população brasileira. Alguns escores, como o da Organização Mundial da Saúde^108^ e o Globorisk-LAC,^109^ têm a vantagem de terem sido calibrados para a população brasileira e podem ser utilizados na avaliação do risco cardiovascular.

Nos EUA, foi publicada em 2024 uma nova equação de risco – o escore PREVENT ( https://professional.heart.org/en/guidelines-and-statements/prevent-calculator ) – derivada de mais de 3 milhões de indivíduos de diversas coortes e validada em outros mais de 3 milhões de indivíduos. Esse escore destina-se a indivíduos entre 30 e 79 anos de idade sem doença cardiovascular prévia. Além dos fatores de risco tradicionais (sexo, idade, CT, HDL-c, pressão arterial sistólica, uso de medicamento anti-hipertensivo, DM e tabagismo), ele incorpora uso de estatina, índice de massa corpórea e taxa de filtração glomerular estimada, sendo opcionais as variáveis hemoglobina glicada e razão albumina-creatina urinária. O escore PREVENT estima o risco de evento cardiovascular aterosclerótico (morte coronária, infarto do miocárdio não fatal ou AVC), o de insuficiência cardíaca (IC) e o risco cardiovascular total, em 10 anos e em 30 anos. O modelo básico das equações PREVENT para risco aterosclerótico mostrou discriminação satisfatória em amostra de validação (estatística C 0,774 em mulheres e 0,736 em homens).^110 , 111^ Considerando sua robustez e contemporaneidade, e diante da ausência de uma equação de risco derivada da população brasileira, esta diretriz recomenda a avaliação do risco de evento cardiovascular aterosclerótico em 10 anos pelo escore PREVENT para definir a necessidade de terapia hipolipemiante.

### 4.3. Fatores Agravantes do Risco Cardiovascular

**Figura 4.2:**
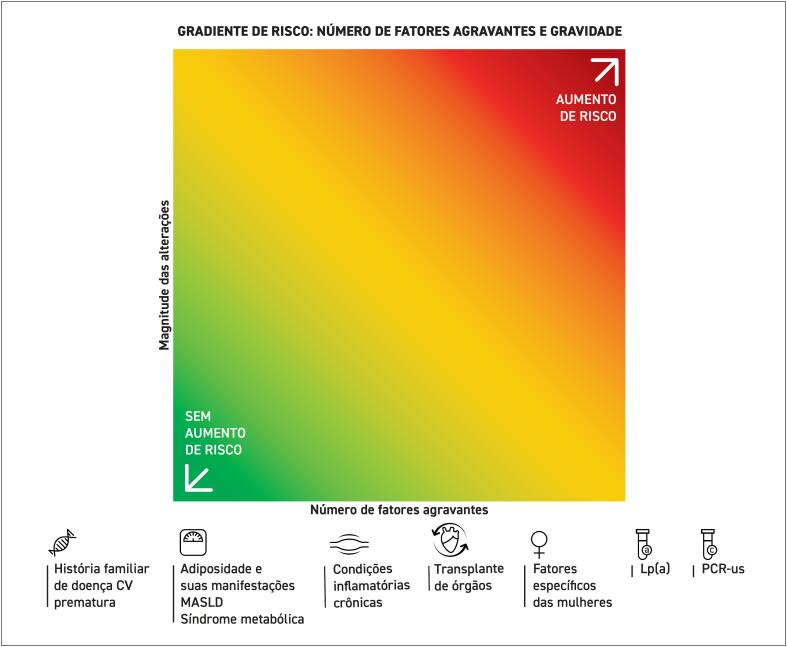
Representação esquemática do uso de fatores agravantes na avaliação do risco cardiovascular. Os fatores agravantes devem ser inseridos no contínuo do risco cardiovascular. Quanto maior o número de fatores e a intensidade das alterações, maior deve ser a alteração do risco em relação àquele calculado por escores tradicionais.

Fatores agravantes de risco são marcadores não contemplados nos escores tradicionais que fornecem uma informação adicional para refinar a avaliação do risco cardiovascular, o que pode ajudar na tomada de decisões clínicas. São particularmente úteis nos casos em que há incerteza sobre a melhor conduta médica a ser adotada. Esses fatores podem ser identificados a partir da história clínica ou de exames complementares ( Tabela 4.3 ), e seu desempenho na avaliação do risco pode ser determinado por métricas como a área sob a curva ROC (discriminação) ou índices de reclassificação do risco. A valorização de fatores agravantes na prática clínica requer um julgamento crítico: quanto maior o número e a intensidade de fatores agravantes, maior a conveniência de se reestratificar para cima o risco ( Figura 4.2 ). A população com risco calculado intermediário é a que mais se beneficia do uso dos fatores agravantes, por ser o estrato onde é mais frequente a reclassificação do risco. Dessa forma, recomenda-se a pesquisa ativa de agravantes de risco nessa população. No entanto, os fatores agravantes também podem ser empregados em indivíduos de risco estimado baixo em 10 anos, principalmente sob a perspectiva de risco ao longo da vida.

**Tabela 4.3:** Fatores agravantes do risco cardiovascular

Categoria de risco	Definição
**História familiar de doença cardiovascular prematura**	Parente de 1º grau com evento em idade < 55 anos (sexo masculino) ou < 65 anos (sexo feminino)
**Adiposidade e suas manifestações**	Adiposidade Esteatose hepática, especialmente formas mais graves (por exemplo, com fibrose) ou associada a fatores cardiometabólicos Síndrome metabólica
**Condições inflamatórias crônicas**	Artrite reumatoide Psoríase Lúpus eritematoso sistêmico Doenças inflamatórias intestinais (retocolite ulcerativa e doença de Crohn) Infecção crônica pelo HIV
**Transplante de órgãos**	Por exemplo, coração, fígado, rim
**Fatores agravantes de risco específicos das mulheres**	Menarca precoce (≤ 12 anos) ou tardia (≥ 17 anos) Distúrbios durante a gestação (pré-eclâmpsia, eclâmpsia, hipertensão gestacional, diabetes gestacional) Parto prematuro Restrição de crescimento intrauterino Abortos de repetição (≥ 3 perdas gestacionais espontâneas) Menopausa precoce (< 40 anos)
**Lipoproteína(a)**	≥ 50 mg/dL ou ≥ 125 nmol/L
**Proteína C-reativa ultrassensível**	≥ 2,0 mg/L

#### 4.3.1 História Familiar de Doença Cardiovascular Prematura

Comparados com participantes sem doença cardiovascular parental, aqueles com pelo menos um dos pais com doença cardiovascular prematura (idade de início < 55 anos no pai ou < 65 anos na mãe) tiveram maior risco de eventos, com razão de chances ajustada de 2,0 (IC95% 1,2-3,1) para homens e 1,7 (IC95% 0,9-3,1) para mulheres.^112^

#### 4.3.2. Adiposidade e suas Manifestações

A obesidade, definida por um índice de massa corporal (IMC) igual ou superior a 30 kg/m², está consistentemente associada a um aumento do risco de eventos cardiovasculares maiores, incluindo doença arterial coronariana, IC e AVC. Metanálises, envolvendo grandes populações, demonstraram que indivíduos com sobrepeso ou obesidade apresentam risco significativamente maior de desenvolver doença arterial coronariana e de mortalidade cardiovascular.^113^ Esse risco é ainda mais elevado em pessoas com DM ou síndrome metabólica.

A obesidade visceral, avaliada pela circunferência da cintura (CC), está significativamente associada a um aumento do risco de eventos cardiovasculares maiores. A CC é uma medida robusta da adiposidade central e representa um fator de risco relevante para DCV, independentemente do IMC.^114^ Metanálises de estudos de coorte demonstram que um aumento de 1 desvio padrão na CC (aproximadamente 12,6 cm) está associado a uma razão de risco ( *hazard ratio* ) de 1,27 (IC95% 1,20-1,33) para eventos cardiovasculares fatais e não fatais, ajustada por idade, sexo e tabagismo.^115^ Esse risco é observado em diferentes populações e em ambos os sexos, sendo ligeiramente maior nas mulheres. Esses dados reforçam a importância da CC como um parâmetro essencial na avaliação e no manejo do risco cardiovascular. O conceito atual de adiposidade é definido por IMC elevado associado a um parâmetro antropométrico (circunferência abdominal, relação cintura/quadril, relação cintura/altura) elevado ou dois parâmetros antropométricos elevados.^116 , 117^

A obesidade visceral está fortemente associada à manifestação de síndrome metabólica (SM) e de esteatoses, incluindo a esteatose hepática. Considerada isoladamente, a SM é um fator agravante bem estabelecido para eventos cardiovasculares maiores em indivíduos sem doença cardiovascular pregressa. Caracteriza-se por um conjunto de alterações – aumento da CC, TG elevados, HDL-c reduzido, pressão arterial elevada e glicemia de jejum elevada – que, em conjunto, aumentam o risco de DCVA. Diversas metanálises demonstraram que a SM está associada a um risco duas vezes maior de doença cardiovascular e 1,5 vez maior de mortalidade por todas as causas, independentemente da definição utilizada.^118^

A esteatose hepática, especialmente na forma de doença hepática esteatótica associada à Disfunção Metabólica (MASLD), está consistentemente associada a um risco aumentado de eventos cardiovasculares maiores. Diversos estudos de coorte demonstraram que indivíduos com MASLD ou doença hepática gordurosa não alcoólica apresentam maiores *hazard ratio* para infarto do miocárdio, AVC, IC congestiva e doença cardiovascular em geral, em comparação com aqueles sem esteatose. As *hazard ratio* ajustadas variam de 1,12 a 1,75, com associações mais fortes observadas em pacientes com confirmação histológica da doença ou com esteatose hepática independente das características da doença arterial coronariana. Metanálises reforçam essa associação.^119^

Diante do exposto, a associação entre obesidade e risco cardiovascular é bem comprovada e tem sua magnitude ampliada à medida que se desenvolve obesidade visceral, esteatoses e SM. A associação com aterogênese se dá de forma múltipla e redundante entre essas manifestações. Por isso, consideramos a SM como fator agravante para reclassificação de risco e os demais achados como parâmetros para definir a condição clínica relacionada à obesidade e para definir a eficácia das intervenções dirigidas à obesidade ou esteatose.

#### 4.3.3. Condições Inflamatórias Crônicas

Diversas condições inflamatórias e autoimunes crônicas, como artrite reumatoide,^120^ psoríase,^121^ lúpus eritematoso sistêmico,^122^ doenças inflamatórias intestinais (retocolite ulcerativa e doença de Crohn)^123^ e infecção crônica pelo HIV,^124^ associam-se de maneira independente a um risco aumentado de diferentes manifestações de DCV, incluindo doença aterosclerótica. Os mecanismos propostos são diversos, envolvendo não só a frequente concomitância de fatores de risco clássicos e efeitos adversos de tratamentos, mas sobretudo a presença de um ambiente pró-inflamatório sistêmico que promove disfunção endotelial e acelera a aterogênese.

Exemplificando, uma metanálise com mais de 500.000 casos e quase 30 milhões de indivíduos-controle mostrou uma associação da psoríase com doença cardiovascular total ( *odds ratio* 1,4; IC95% 1,2-1,7), doença cardíaca isquêmica ( *odds ratio* 1,5; IC95% 1,2-1,9) e doença vascular periférica ( *odds ratio* 1,5; IC95% 1,2-1,8).^125^ No caso das pessoas vivendo com HIV, uma grande revisão sistemática mostrou uma razão de risco de 2,16 (IC95% 1,68-2,77) de desenvolver doença cardiovascular em relação aos indivíduos sem HIV.^126^ Dessa forma, equações tradicionais de risco cardiovascular podem subestimar o risco verdadeiro em pessoas com doenças inflamatórias e autoimunes, justificando a sua inclusão no rol de fatores agravantes.

#### 4.3.4. Transplante de Órgãos

Indivíduos submetidos a transplante de órgãos, como coração, fígado e rim, apresentam risco aumentado de diferentes modalidades de doença cardiovascular, incluindo doença arterial coronariana, em relação à população geral. Os mecanismos parecem ser multifatoriais: frequente presença de fatores de risco tradicionais (hipertensão arterial, DM, dislipidemia), efeitos metabólicos de imunossupressores, como corticosteroides, inflamação crônica e ativação imunológica, levando à disfunção endotelial, aterosclerose acelerada e estado pró-trombótico.^127^ Estas observações reforçam considerar a condição pós-transplante de órgão como um fator agravante do risco cardiovascular.

#### 4.3.5. Fatores Agravantes de Risco Específicos das Mulheres

A diretriz ressalta a importância de considerar vários fatores específicos da mulher na análise do risco cardiovascular.

##### 4.3.5.1. Idade da Menarca

A idade da menarca é um fator de risco cardiovascular não tradicional, mas relevante, na avaliação do risco em mulheres. Tanto a menarca precoce (geralmente definida como ≤ 12 anos) quanto a menarca tardia (≥ 17 anos) estão associadas a um aumento do risco de eventos cardiovasculares e mortalidade por todas as causas, com maior robustez de evidências para o risco associado à menarca precoce.^128^

##### 4.3.5.2. Distúrbios Durante a Gestação e Parto Prematuro

Distúrbios hipertensivos durante a gestação (pré-eclâmpsia, eclâmpsia e hipertensão gestacional), diabetes gestacional, parto prematuro e restrição de crescimento intrauterino são associados a aumento do risco cardiovascular futuro. Uma metanálise de dados de mais de 6,4 milhões de mulheres (258.000 com pré-eclâmpsia) demonstrou que a pré-eclâmpsia estava independentemente associada a um risco quatro vezes maior de IC incidente e um risco duas vezes maior de doença coronária. Da mesma forma, mulheres com diabetes gestacional têm um risco duas vezes maior de eventos cardiovasculares (razão de risco 1,98; IC95% 1,57-2,50).^129^

O parto prematuro tem um impacto imediato físico e emocional, estando associado a aumento do risco de hospitalização materna por DCV, incluindo infarto do miocárdio, AVC e outras hospitalizações cardiovasculares.^130^

##### 4.3.5.3. Abortos de Repetição

A ocorrência de abortos recorrentes (três ou mais perdas gestacionais espontâneas) está associada a um aumento significativo do risco cardiovascular materno no longo prazo, incluindo doença arterial coronariana, AVC e hipertensão. O risco é mais pronunciado em mulheres jovens (< 35 anos) e aumenta progressivamente com o número de perdas gestacionais.^131^ Portanto, o histórico de abortos recorrentes deve ser considerado um marcador independente de risco cardiovascular em mulheres, devendo chamar a atenção para vigilância clínica e intervenções preventivas precoces, para modificação de fatores de risco tradicionais e acompanhamento cardiometabólico ao longo da vida.

##### 4.3.5.4. Menopausa Precoce

A menopausa precoce (definida como menopausa antes dos 45 anos, especialmente antes dos 40 anos) está associada a um aumento do risco de DCV, incluindo doença arterial coronariana, IC, AVC, doença arterial periférica e eventos cardiovasculares maiores,^132^ devendo ser incorporada à estratificação de risco e à tomada de decisão sobre intervenções preventivas.

### 4.4. Exames Complementares

#### 4.4.1. Lipoproteína(a)

A associação da Lp(a) com risco cardiovascular é bem documentada por diversos estudos de coorte prospectivos. O estudo *Emerging Risk Factors Collaboration* (ERFC) analisou o impacto da elevação da Lp(a) sobre risco de eventos cardiovasculares em indivíduos sem doença cardiovascular prévia. As taxas de evento coronário nos terços superior e inferior das distribuições de Lp(a), respectivamente, foram 5,6 (IC95% 5,4-5,9) por 1.000 pessoas-ano e 4,4 (IC95% 4,2-4,6) por 1.000 pessoas-ano. A razão de risco para evento coronário, ajustado apenas para idade e sexo, foi de 1,16 (IC95% 1,11-1,22) por concentração usual de Lp(a) 3,5 vezes maior (ou seja, por um desvio-padrão), e de 1,13 (IC95% 1,09-1,18) após ajuste adicional para lípides e outros fatores de risco convencionais. As razões de risco ajustadas correspondentes foram 1,10 (IC95% 1,02-1,18) para AVC isquêmico e 1,01 (IC95% 0,98-1,05) para o agregado de mortalidade não-vascular.^133^ Dados de outra coorte do Reino Unido ( *UK Biobank* ) mostrou que a relação entre Lp(a) e doença aterosclerótica cardiovascular pareceu linear em toda a distribuição, com uma razão de risco de 1,11 (IC95% 1,10-1,12) por incremento de 50 nmol/L).^64^

O nível de Lp(a) maior ou igual a 50 mg/dL ou 125 nmol/L indica um agravante de risco, elevando o risco de baixo risco para intermediário ou de intermediário para alto. Níveis muito elevados, acima de 180 mg/dL ou 390 nmol/L, indicam alto risco de evento cardiovascular.^65^

#### 4.4.2. Proteína C-Reativa Ultrassensível

A inflamação tem um papel central na fisiopatologia da doença aterosclerótica. A proteína C-reativa (PCR) sérica, especialmente quando mensurada por ensaios ultrassensíveis, é um biomarcador de inflamação sistêmica associada com desfechos cardiovasculares, incluindo infarto do miocárdio e AVC em inúmeros estudos epidemiológicos. No estudo *Framingham Offspring* , a PCR ultrassensível gerou melhora líquida na reclassificação de risco aos fatores tradicionais de 5,6% para evento cardiovascular (p = 0,014) e 11,8% para evento coronário (p = 0,009).^134^ Uma revisão sistemática relatou que análises de quatro grandes coortes foram consistentes em encontrar evidências de que a inclusão de PCR melhora a estratificação de risco entre pessoas com risco inicialmente intermediário.^135^ Dessa forma, a diretriz recomenda considerar PCR ultrassensível ≥ 2,0 mg/L como agravante de risco.

Uma análise recente do *Women´s Health Study,* que avaliou cerca de 27.000 mulheres inicialmente saudáveis, mostrou que a medida única combinada de PCR ultrassensível, LDL-c e Lp(a) foi preditora de eventos cardiovasculares incidentes durante um período de 30 anos de seguimento. Esse dado sugere que o uso desses biomarcadores pode auxiliar na estratificação de risco ao longo da vida, adicionando potencial benefício aos modelos tradicionais de avaliação de risco.^136^ Dois grandes estudos subsequentes ratificaram o poder preditivo dessa combinação de biomarcadores em homens e mulheres, a despeito do uso de hipolipemiantes.^137 , 138^ Além do potencial de predição de risco desses três parâmetros combinados, a demonstração de benefício a partir da redução de suas concentrações [com o benefício cardiovascular da redução das concentrações de Lp(a) ainda não demonstrado] sugere a importância do início de estratégias preventivas precoces mediante valores elevados dessas variáveis.

#### 4.4.3. *Troponinas* Cardíacas de Alta Sensibilidade

As troponinas cardíacas I e T são biomarcadores circulantes específicos com reconhecido papel no diagnóstico e prognóstico das síndromes coronárias agudas. Em indivíduos com síndrome coronária crônica e na população geral, os ensaios de troponina cardíaca de alta sensibilidade (Tn-as) podem detectar injúria miocárdica de baixo grau, que também detém valor prognóstico. Diversos estudos observacionais prospectivos em populações saudáveis demonstraram que níveis elevados de Tn-as, mesmo dentro da faixa considerada normal, estão independentemente associados a desfechos cardiovasculares, incluindo morte cardiovascular, doença arterial coronária e AVC,^139^ fornecendo valor incremental à estratificação de risco cardiovascular além dos fatores de risco tradicionais.^140^ A Tn-as pode também ser usada em conjunto com a calcificação arterial coronária (CAC): no *Multi-Ethnic Study of Atherosclerosis* (MESA), a incidência de eventos ateroscleróticos foi similar em indivíduos com Tn-as indetectável ou com CAC = 0 no momento basal, enquanto aqueles com Tn-as detectável e CAC > 0 apresentaram a maior taxa de eventos ( *hazard ratio* 3,50 em relação ao grupo de referência com hs-cTnT indetectável e CAC = 0).^141^

Apesar de seu potencial, o uso da Tn-as na estratificação de risco cardiovascular em indivíduos saudáveis apresenta limitações importantes. Como as concentrações séricas de Tn-as são muito baixas (da ordem de pg/mL), variações significativas podem ocorrer dentro da faixa de normalidade com base na imprecisão dos ensaios. Existem também variações fisiológicas relacionadas ao sexo e à idade, com valores geralmente mais elevados em homens e em idosos. Além disso, não está bem definido o ponto de corte para discriminar com precisão indivíduos de maior ou menor risco cardiovascular, embora algumas propostas tenham sido feitas. Para uma marca específica de ensaio para dosagem de hs-cTn, Farmakis *et al* . sugeriram considerar alto risco quando a hs-cTnI for > 12 ng/L em homens ou > 10 ng/L em mulheres, baixo risco quando < 6 ng/L em homens ou < 4 ng/L em mulheres, e risco intermediário para valores entre esses limites.^139^

#### 4.4.4. Peptídeo Natriurético Tipo B e Peptídeo Natriurético Tipo B N-Terminal NT-proBNP

Estudos prospectivos de base populacional demonstraram que níveis elevados do peptídeo natriurético tipo B (BNP) ou de seu fragmento N-terminal (NT-proBNP) predizem risco aumentado para uma ampla gama de eventos clínicos, incluindo morte por todas as causas, morte cardiovascular, IC, fibrilação atrial, AVC, ataque isquêmico transitório e o desfecho composto por doença arterial coronária e AVC, independentemente de outros fatores de risco.^142 , 143^ O NT-proBNP também demonstrou valor incremental na discriminação e reclassificação do risco cardiovascular, quando adicionado a modelos baseados em fatores de risco tradicionais.^143^ No entanto, ainda não há limiares bem definidos para reestratificar o risco com base nos níveis de NT-proBNP. Da mesma forma, não há ensaios clínicos randomizados que guiem intervenções terapêuticas baseadas nesse biomarcador ou estudos de custo-efetividade que sustentem seu uso rotineiro na prática clínica. Considerando que a associação entre BNP e desfechos cardiovasculares é mais forte para IC e que a relação específica com eventos coronários nem sempre foi observada,^142^ esta diretriz não recomenda a dosagem de BNP ou NT-proBNP para a estratificação do risco cardiovascular aterosclerótico no indivíduo assintomático ou sem doença cardiovascular pregressa.

### 4.5. Marcadores de Doença Aterosclerótica Subclínica

Exames de imagem que demonstram a presença de aterosclerose subclínica podem ser utilizados como um indício da própria doença aterosclerótica em evolução. Obviamente existem diferentes graus dessa apresentação, inseridos dentro de um contínuo, e, quanto maior a presença e carga da doença subclínica, maior o risco de evento cardiovascular. As duas principais ferramentas para a identificação de aterosclerose subclínica são o escore de cálcio da artéria coronária (CAC) e o ultrassom das artérias carótidas com avaliação da presença de placa carotídea. O CAC pode ser quantificado de forma objetiva na tomografia computadorizada de artérias coronárias. Já a presença de placa carotídea, para a qual existem diferentes definições na literatura, pode depender da avaliação do operador.

#### 4.5.1. Escore de Cálcio Coronário

O CAC representa a parte calcificada da placa de ateroma na artéria coronária. Quanto maior o CAC, maior a carga total de placa e maior o risco de evento cardiovascular aterosclerótico. A ausência de CAC (CAC = 0) é um forte marcador negativo do risco cardiovascular, associando-se à baixa taxa de eventos cardiovasculares e mortalidade no decorrer de 10 anos. O prognóstico favorável do CAC = 0 é observado mesmo em pessoas idosas ou naquelas com múltiplos fatores de risco, mas é menos evidente em indivíduos com DM, tabagistas ou com história familiar de doença aterosclerótica prematura.^144^ Por outro lado, indivíduos com CAC > 300 unidades Agatston (UA) apresentam risco de eventos cardiovasculares semelhante aos daqueles que já apresentaram um evento clínico aterosclerótico,^145^ devendo, portanto, ser classificados como de *risco muito alto* . Aqueles com CAC > 100 UA ou percentil > 75 para sexo e idade devem ser classificados como de *risco alto* . Nos portadores de HF, o CAC > 100 UA indica *risco muito alto* ( Tabela 4.4 ).

**Tabela 4.4:** Escore de cálcio da artéria coronária na estratificação de risco cardiovascular

Indivíduos com risco calculado intermediário, idade > 40 anos e LDL-c entre 70-159 mg/dL apresentam benefício da quantificação do CAC, quando disponível
Indivíduos com CAC > 100 UA ou percentil > 75 para sexo e idade devem ser classificados como de alto risco cardiovascular
Indivíduos com CAC > 300 UA devem ser classificados como de muito alto risco cardiovascular
Indivíduos com diabetes mellitus e CAC entre 10 e 300 UA devem ser classificados como de alto risco cardiovascular
Portadores de HF com CAC > 100 UA devem ser classificados como de muito alto risco cardiovascular

UA: unidades Agatston; CAC: escore de cálcio coronariano; HF: hipercolesterolemia familiar.

**Figura 4.3:**
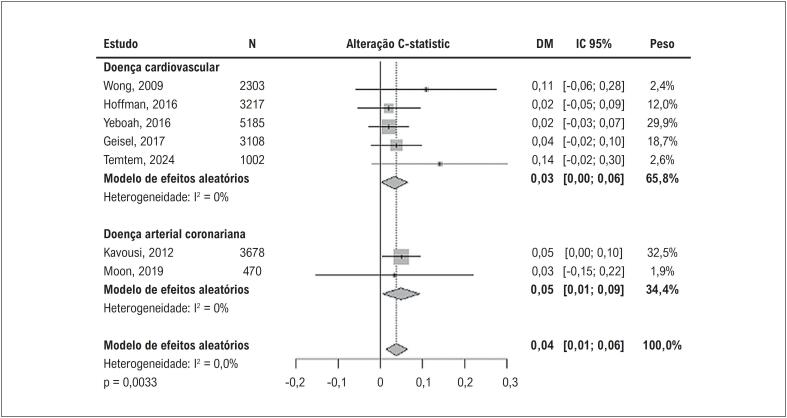
Metanálise avaliando a alteração da estatística-C com a adição do escore de cálcio coronário ao escore de risco cardiovascular tradicional para eventos cardiovasculares adversos maiores.^146^

**Figura 4.4:**
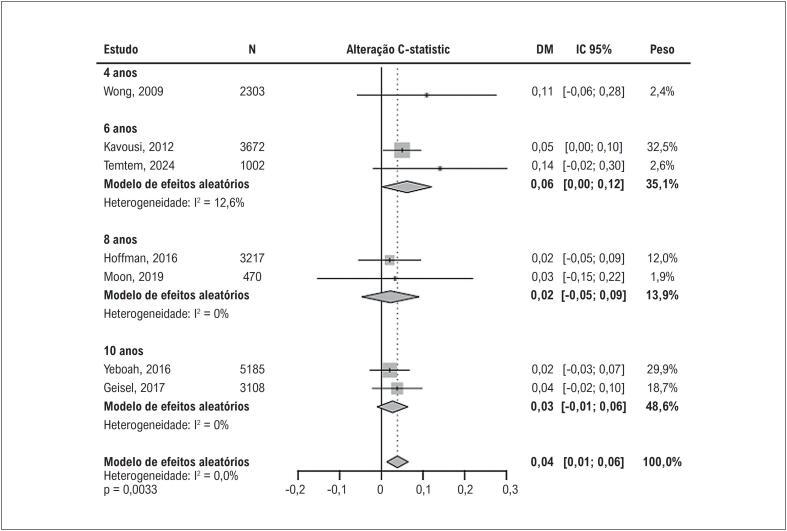
Metanálise avaliando a reclassificação de risco considerando a adição do escore de cálcio coronário ao escore de risco cardiovascular tradicional.^146^

Uma revisão sistemática e metanálise foi realizada, incluindo estudos que avaliaram o desempenho do CAC frente a escores de risco tradicionais. Independentemente da modelagem, a metanálise mostrou associação consistente entre aumento do CAC e maior risco de eventos cardiovasculares. A adição de CAC ao escore de risco tradicional aumentou em 0,04 unidade a discriminação de risco comparado ao escore de risco tradicional apenas (DM 0,04; IC95% 0,01-0,06; I^2^ 0%; p = 0,0033; moderada certeza da evidência; Figura 4.3 ).^146^ O desfecho de reclassificação de risco considerando a adição de CAC ao escore de risco cardiovascular tradicional foi analisado nos seis estudos incluídos e avaliado pelo *net reclassification improvement* (NRI). A adição de CAC ao escore de risco tradicional aumentou em 0,33 unidade a reclassificação de risco (escala de 0 a 1), comparado ao escore de risco tradicional apenas (DM 0,33; IC95% 0,17-0,49; I^2^ 87,5%; p < 0,001; moderada certeza da evidência; Figura 4.4 ). Essas informações suportam a recomendação para o uso do CAC, quando disponível, em indivíduos com idade > 40 anos, LDL-c entre 70-159 mg/dL e risco calculado intermediário (ou mesmo baixo, com histórico familiar de doença aterosclerótica prematura), para decidir sobre a necessidade e a intensidade da terapia hipolipemiante.^146^

Embora estudos de custo-efetividade conduzidos no Reino Unido, Canadá^147^ e EUA^148^ recomendem o uso de CAC em pacientes em risco intermediário (risco em 10 anos pelas *pooled cohort equations* 5-7,5% ou pelo QRISK3 10-20%) e advoguem o uso de estatinas de alta intensidade em pacientes com CAC ≥ 1 e a não indicação de estatina em pacientes com CAC = 0, um estudo brasileiro sugere o uso de estatinas de moderada intensidade em pacientes com CAC ≥ 1, usando a população do estudo MESA e microssimulação baseada em custos brasileiros.^149^

#### 4.5.2. Ultrassom das Artérias Carótidas

Sendo uma ferramenta de baixíssimo risco e relativa disponibilidade, o ultrassom de diferentes leitos arteriais, como as artérias carótidas e os membros inferiores, pode ser empregado para a detecção de aterosclerose subclínica.^150 - 152^ Em relação à predição de risco cardiovascular, a modalidade mais estudada é o ultrassom das artérias carótidas, que permite a análise de duas variáveis principais: a espessura íntima-média carotídea (EIMC) e a presença de placa aterosclerótica. A placa carotídea pode ser definida pelos seguintes critérios: 1) qualquer espessamento focal considerado de origem aterosclerótica e que invada o lúmen de qualquer segmento da artéria carótida (placa do tipo protuberante); ou 2) no caso de aterosclerose difusa da parede do vaso, quando a EIMC for ≥ 1,5 mm em qualquer segmento da artéria carótida (placa do tipo difusa).^150^

Uma metanálise de 14 estudos mostrou que um aumento de um desvio-padrão na EIMC da artéria carótida comum associou-se a risco aumentado de AVC ( *hazard ratio* 1,32; IC95% 1,27-1,38), infarto do miocárdico ( *hazard ratio* 1,27; IC95% 1,22-1,33) e desfecho composto de AVC e infarto ( *hazard ratio* 1,28; IC95% 1,19-1,37). No entanto, a capacidade da EIMC de discriminar o risco além dos fatores de risco do escore de Framingham não se mostrou relevante: aumento da estatística-C de 0,757 para 0,759, e o NRI foi baixo: 0,8% para toda população e 3,6% para população de risco intermediário.^151^ Diante da falta de padronização da medida da EIMC e o baixo poder de reclassificação de risco, no momento não existem evidências que embasem o uso da EIMC como agravante de risco na prática clínica.

Por outro lado, a presença de placa carotídea em indivíduos assintomáticos é melhor preditor de eventos cardiovasculares do que a EIMC. Uma metanálise de 11 estudos populacionais mostrou que a placa carotídea, comparada à EIMC, teve melhor desempenho para a predição de infarto do miocárdio (área sob a curva ROC 0,64 *versus* 0,61).^152^ Uma análise de coorte sueca mostrou que as melhorias da estatística-C e do NRI com adição da placa ao SCORE2 para eventos ocorridos nos primeiros 10 anos foram de 2,20% e 46,1%, respectivamente (ambos p < 0,0001).^153^ Portanto, a presença de placa carotídea fornece informação incremental em relação ao uso isolado de escore clínico.

### 4.6. Estratificação do Risco Cardiovascular no Diabetes *Mellitus*

O DM2 está associado a um risco cardiovascular duas vezes maior em comparação à população sem DM. Trata-se de um fator de risco independente não apenas para doenças ateroscleróticas, como infarto do miocárdio e AVC isquêmico, mas também para IC e fibrilação atrial.^154 , 155^ Na avaliação do risco cardiovascular em indivíduos com DM2, além dos fatores de risco tradicionais, devem ser consideradas variáveis específicas relacionadas ao diabetes, como a duração do diabetes (risco substancialmente aumentado quando ≥ 10 anos), níveis de hemoglobina glicada e presença de lesão de órgãos-alvo. Esta diretriz propõe a classificação do risco cardiovascular em indivíduos com DM2 em quatro categorias (riscos intermediário, alto, muito alto e extremo), com base na idade do paciente e na presença ou não de estratificadores de risco ou critérios de risco extremo ( Tabelas 4.5 , 4.6 , 4.7 , 4.8 ).

**Tabela 4.5:** Estratificação do risco cardiovascular no diabetes *mellitus*

Categoria de risco	DM2	DM1	Condição necessária
**Intermediário**	Homem < 50 anos Mulher < 56 anos	Usar a calculadora ST1RE se < 20 anos de duração	Sem EAR Sem EMAR Sem critério de extremo risco
**Alto**	Homem ≥ 50 anos Mulher ≥ 56 anos		1 ou 2 EAR Sem EMAR Sem critério de extremo risco
	DM1 e DM2: qualquer idade se EAR		
**Muito alto**	Qualquer idade se EMAR		EMAR ou ≥ 3 EAR
**Extremo**	Qualquer idade se critério de extremo risco		Critério de extremo risco*

* Critério de extremo risco: histórico de múltiplos eventos cardiovasculares ateroscleróticos maiores ou 1 evento cardiovascular aterosclerótico maior e pelo menos 2 condições de alto risco (Quadro 8). DM1: diabetes *mellitus* tipo 1; DM2: diabetes *mellitus* tipo 2; ST1RE: Steno Type 1 Risk Engine. Adaptado de Izar et al.^157^

**Tabela 4.6:** Estratificadores de alto risco no diabetes *mellitus*

Tradicionais	
	DM2 há mais de 10 anos
	História familiar de doença arterial coronária prematura
	Síndrome metabólica definida pelo IDF
	Hipertensão arterial tratada ou não
	Tabagismo ativo
	Neuropatia autonômica cardiovascular incipiente
	Retinopatia diabética não proliferativa leve
**Renais**	
	Doença renal estratificada como risco alto (ver Quadro 4.5)
**Doença aterosclerótica subclínica**	
	Escore de cálcio coronário entre 10-300 UA
	Placa carótida < 50%
	Angiotomografia coronária computadorizada com placa aterosclerótica <50%
	Aneurisma da aorta abdominal

DM2: diabetes *mellitus* tipo 2; IDF: International Diabetes Federation; UA: unidades Agatston; EAR: estratificador de alto risco; EMAR: estratificador de muito alto risco. Adaptado de Izar et al.^157^

**Tabela 4.7:** Risco cardiovascular de acordo com os estratificadores renais de alto risco e de muito alto risco no diabetes *mellitus*

Estágios da DRD TFG (mL/min/1.73m^2^)			Normal	Moderadamente aumentada (microalbuminúria)	Muito aumentada (macroalbuminúria)
			< 30 mg/g	30-299 mg/g	≥300 mg/g
**G1**	Normal ou alta	≥ 90	Ver idade, EAR e EMAR	Risco alto	Risco muito alto
**G2**	Levemente reduzida	89-60		Risco alto	Risco muito alto
**G3a**	Leve a moderadamente reduzida	59-45	Risco alto	Risco alto	Risco muito alto
**G3b**	Moderadamente reduzida	44-30	Risco alto	Risco muito alto	Risco muito alto
**G4-G5**	Muito reduzida ou Falência renal	<30	Risco muito alto	Risco muito alto	Risco muito alto

DRD: doença renal do diabetes; TFG: taxa de filtração glomerular. EAR: estratificador de alto risco; EMAR: estratificador de muito alto risco. Adaptado de Izar et al.^157^

**Tabela 4.8:** Estratificadores de muito alto risco no diabetes *mellitus*

Sem evento cardiovascular aterosclerótico manifesto prévio de muito alto risco – (EMAR-1)	
	Três ou mais EAR.
	DM1 com duração maior que 20 anos, diagnosticado após os 18 anos de idade.
	Estenose maior do que 50% em qualquer território vascular.
	EMAR renal (ver Quadro 5).
	Hipercolesterolemia grave: CT > 310 mg/dL ou LDL-c > 190 mg/dL.
	Neuropatia autonômica cardiovascular instalada: dois testes autonômicos cardiovasculares (TAC) alterados para NAC.
	Retinopatia diabética não proliferativa moderada-severa ou severa, proliferativa, ou evidência de progressão.
**Evento cardiovascular aterosclerótico manifesto prévio – (EMAR-2)**	
	Síndrome coronariana aguda: infarto agudo do miocárdio ou angina instável.
	Infarto agudo do miocárdio antigo ou angina estável.
	Acidente vascular cerebral aterotrombótico ou ataque isquêmico transitório (AIT).
	Revascularização coronariana, carotídea, renal ou periférica.
	Insuficiência vascular periférica ou amputação de membros.

CT: colesterol total; DM1: diabetes *mellitus* tipo 1; DM2: diabetes mellitus tipo 2; EAR: estratificador de alto risco; EMAR: estratificador de muito alto risco; LDL-c: colesterol da lipoproteína de baixa densidade. Adaptado de Izar et al.^157^

Para indivíduos com DM tipo 1, com menos de 20 anos de duração e sem estratificadores de risco nem critérios de risco extremo, a diretriz recomenda o uso do Steno Type 1 Risk Engine para a predição de um primeiro evento cardiovascular ( https://steno.shinyapps.io/T1RiskEngine/ )^156^ ( Tabela 4.5 ).

### 4.7. As Categorias do Risco Cardiovascular Aterosclerótico

A diretriz preconiza não utilizar a divisão binária "prevenção primária" e "secundária", mas reconhece o risco cardiovascular dentro do chamado contínuo de risco, ou seja, o risco cardiovascular como uma variável contínua que tem, em uma extremidade, o *risco baixo* do indivíduo jovem sem manifestação de evento cardiovascular e sem fatores de risco e, na outra ponta, o *risco extremo* do indivíduo portador de múltiplos eventos cardiovasculares recorrentes ( Figura Central ).

As definições das categorias de risco preconizadas por esta diretriz são descritas na Tabela 4.3

### 4.8. Particularidades da Estratificação do Risco Cardiovascular em Idosos

A avaliação do risco cardiovascular em pessoas idosas apresenta algumas particularidades. Inicialmente, é bem reconhecido que a força da associação entre fatores de risco e eventos cardiovasculares atenua com o envelhecimento. Por outro lado, como a idade é o fator com maior peso na determinação do risco, o risco absoluto de eventos cardiovasculares se eleva com o envelhecimento. Em segundo lugar, escores de risco antigos não levavam em consideração o risco competitivo do indivíduo falecer por uma causa não cardiovascular, o que frequentemente superestimava o risco e, consequentemente, o benefício predito de intervenções terapêuticas em idosos. Em terceiro lugar, a maioria das equações de risco foi desenvolvida para populações com idade < 80 anos. O escore PREVENT, por exemplo, destina-se a indivíduos até os 79 anos de idade,^110 , 111^ embora tenha mostrado bom desempenho em pessoas mais idosas em um estudo.^158^

Na Europa, o SCORE2-OP foi desenvolvido para estimar o risco cardiovascular em indivíduos com idade ≥ 70 anos.^159^ O escore foi calibrado para quatro regiões, de acordo com as taxas de mortalidade cardiovascular dos países europeus, sendo que as taxas das localidades de moderado risco são as que mais se aproximam da mortalidade brasileira. Segundo o SCORE2-OP para regiões europeias de moderado risco, o risco de morte cardiovascular, infarto do miocárdio não fatal ou AVC não fatal em 10 anos para pessoas com idade ≥ 80 anos é invariavelmente ≥ 20%, fazendo desta população um subgrupo de alto risco.

### 4.9. Particularidades da Estratificação do Risco Cardiovascular em Adultos Jovens

Sendo a idade o principal fator determinante do risco cardiovascular, indivíduos jovens frequentemente apresentam risco calculado em 10 anos baixo, mesmo com fatores de risco descontrolados. O escore PREVENT adotado nesta diretriz não foi desenvolvido para indivíduos com menos de 30 anos de idade;^110 , 111^ portanto, essa faixa etária permanece carente de um modelo de avaliação de risco. Mesmo os modelos de estimativa de risco ao longo da vida não contemplam indivíduos muito jovens: o *lifetime risk,* preconizado pela American Heart Association, inclui indivíduos a partir dos 50 anos de idade,^59^ e o LIFE-CVD2, a partir de 35 anos de idade.^161^

Além dos fatores de risco convencionais, esta diretriz propõe a avaliação de fatores agravantes de risco nos adultos menores de 30 anos de idade. A depender do tipo e intensidade do fator agravante, o indivíduo pode ser reclassificado para uma categoria de risco mais elevado, principalmente considerando as consequências de longo prazo.

Para adultos jovens com idade ≥ 30 anos, o cálculo do risco cardiovascular em 30 anos pelo escore PREVENT pode ser oportuno. Embora não existam limiares definidos para a categorização do risco, a estimativa em 30 anos pode ser utilizada com o objetivo de aumentar a conscientização sobre os fatores de risco e a motivação do paciente em relação ao seu tratamento.

### 4.10. Estratificação do Risco Cardiovascular na Infância e Adolescência

O risco de doença cardiovascular em crianças e adolescentes pode ser estratificado pela exposição a fatores de risco tradicionais (HF homozigótica ou heterozigótica, hipertensão arterial, obesidade grave e DM2). Além disso, a presença de doenças subjacentes (como por exemplo, DM1, doença renal crônica, tratamento de câncer infantil, condições inflamatórias crônicas, como artrite inflamatória juvenil) pode tornar o paciente mais vulnerável aos efeitos adversos dos fatores de risco tradicionais, justificando terapias agressivas para o seu controle, visando a redução do risco cardiovascular.^162^ A presente diretriz propõe o modelo de estratificação de risco proposto pela American Heart Association em 2019, dividindo as crianças em categorias de risco de acordo com doenças de base^162^ ( Tabela 4.9 ).

**Table:** 

Recomendação	Força da recomendação	Certeza da evidência
Para indivíduos entre 30-79 anos, sem doença cardiovascular prévia estabelecida, recomenda-se a favor do uso de equação de risco para estimar o risco de evento cardiovascular aterosclerótico em 10 anos.	FORTE	ALTA
Para indivíduos entre 30-79 anos, sem doença cardiovascular prévia estabelecida, recomenda-se a favor do uso do escore PREVENT para a estimar o risco de evento cardiovascular aterosclerótico.	FORTE	ALTA
Para a população de risco calculado intermediário, recomenda-se a favor do uso de fatores agravantes para reclassificar o risco, independentemente da faixa etária.	FORTE	ALTA
Para a população de risco calculado baixo ou com idade entre 18-30 anos, o uso de fatores agravantes pode ser empregado para reclassificar o risco.	FORTE	BAIXA
Para indivíduos classificados inicialmente como de risco intermediário, com idade > 40 anos e LDL-c entre 70-159 mg/dL, o escore de cálcio coronário pode ser útil para decidir sobre a necessidade e a intensidade da terapia hipolipemiante.	FORTE	MODERADA
Para indivíduos classificados inicialmente como de risco baixo, com idade > 40 anos e LDL-c entre 70-159 mg/dL, o escore de cálcio coronário é razoável para aqueles com histórico familiar de DCVA prematura para definir a necessidade e a intensidade da terapia hipolipemiante.	CONDICIONAL	MODERADA

PREVENT: Predicting Risk of Cardiovascular Disease Events; LDL-c: colesterol da lipoproteína de baixa densidade; DCVA: doença cardiovascular aterosclerótica.

**Tabela 4.9:** Estratificação do risco cardiovascular na infância e adolescência de acordo com doenças de base

Categoria de risco	Doenças de base
**Alto**	HF homozigótica, DM1, DM2, doença renal terminal, doença de Kawasaki com aneurismas persistentes, vasculopatia de transplante de órgão sólido, sobrevivente de câncer infantil (receptor de células-tronco)
**Médio**	Obesidade grave, HF heterozigótica, hipertensão arterial confirmada, coarctação de aorta, Lp(a) elevada, DRC pré-diálise, EA, sobrevivente de câncer infantil (radioterapia torácica)
**Em risco**	Obesidade, resistência à insulina com comorbidades (dislipidemia, DHGNA, SOP), hipertensão arterial do avental branco, CMH e outras miocardiopatias, hipertensão pulmonar, condições inflamatórias crônicas (AIJ, LES, DII, HIV), translocação de artéria coronária para artérias coronárias anômalas ou transposição das grandes artérias, câncer infantil (apenas quimioterapia cardiotóxica), doença de Kawasaki com aneurismas regredidos

AIJ: artrite reumatoide juvenil; CMH: cardiomiopatia hipertrófica; DHGNA: doença hepática gordurosa não alcoólica; DII: doença inflamatória intestinal; DM1: diabetes *mellitus* tipo 1; DM2: diabetes *mellitus* tipo 2; DRC: doença renal crônica; EA: estenose aórtica; HF: hipercolesterolemia familiar; LES: lúpus eritematoso sistêmico; Lp(a): lipoproteína(a); SOP: síndrome dos ovários policísticos.

## 5. Metas de Tratamento

### 5.1. Meta Primária e Coprimária: Colesterol de Lipoproteína de Baixa Densidade e Colesterol Não Associado à Lipoproteína de Alta Densidade

Embora reconheçamos que as metas de colesterol definidas não tenham sido sistematicamente testadas, mas derivadas de subanálises dos ensaios clínicos randomizados (ECR) com base no LDL-c alcançado, acreditamos que a definição de alvos claros possa permitir a personalização do tratamento, alinhando-o ao risco cardiovascular individual. Além disso, a utilização de metas pode facilitar a comunicação entre médico e paciente, favorecendo a adesão ao tratamento e contribuindo para a redução da inércia terapêutica ( Tabela 5.1 ).

**Tabela 5.1:** Metas recomendadas de acordo com o risco cardiovascular

Categoria de risco cardiovascular	Meta de LDL-c (mg/dL)	% redução do LDL-c	Meta de colesterol não-HDL (mg/dL)	Meta de ApoB (mg/dL)
	Meta primária	Meta primária	Meta coprimária	Meta secundária
**Baixo risco**	< 115	≥ 30%	< 145	< 100
**Risco intermediário**	< 100	≥ 30%	< 130	< 90
**Alto risco**	< 70	≥ 50%	< 100	<70
**Muito alto risco**	< 50	≥ 50%	< 80	< 55
**Risco extremo**	< 40	≥ 50%	< 70	< 45

As evidências provenientes de estudos de intervenção e meta-regressões são claras e indiscutíveis quanto à redução do risco cardiovascular mediada pela diminuição do LDL-c.^163 - 169^ Essa redução é proporcional à magnitude da diminuição do LDL-c: para cada 39 mg/dL (1 mmol/L) de redução de LDL-c, o risco relativo de eventos cardiovasculares maiores é reduzido em aproximadamente 20 a 25%, sem demonstração de um limite inferior de LDL-c abaixo do qual não haja benefício adicional.^166^ Com base nesses dados, esta diretriz recomenda, além das metas específicas, a adoção de uma redução percentual mínima do LDL-c.

O não-HDL-c, obtido pela subtração direta dos níveis de HDL-c da concentração total de colesterol plasmático, apresenta forte correlação com os níveis séricos de ApoB circulante.^57 , 170 - 172^ Por esse motivo, é considerado um marcador mais fidedigno da carga aterogênica associada às lipoproteínas ricas em lipídios do que a concentração isolada de LDL-c, especialmente em indivíduos com hipertrigliceridemia.^171 - 173^ Nessas condições – nas quais as partículas de LDL encontram-se enriquecidas com TG – a concentração de colesterol presente nas partículas de LDL pode estar relativamente reduzida, levando à subestimação da carga aterogênica total conferida tanto pela LDL quanto por outras lipoproteínas aterogênicas.^174^ Assim, o colesterol não-HDL tem sido reconhecido como um preditor mais acurado do risco cardiovascular nesses contextos clínicos.^175 - 177^ A meta recomendada para o colesterol não-HDL, com o objetivo de reduzir o risco de eventos cardiovasculares ateroscleróticos, é definida como um valor 30 mg/dL superior à meta estabelecida para o LDL-c em cada categoria de risco cardiovascular.^55^ Esse parâmetro é considerado um alvo terapêutico adicional, a ser perseguido após o alcance da meta de LDL-c preconizada para o respectivo estrato de risco.^55^

Assim, esta diretriz estabelece metas para o LDL-c como alvo terapêutico primário e para o colesterol não-HDL como alvo coprimário, ambas definidas conforme a categoria de risco cardiovascular.

Em consonância com as evidências de ensaios clínicos randomizados, estudos genéticos e dados epidemiológicos, recomenda-se a introdução de terapia farmacológica em indivíduos classificados como de baixo risco, com níveis de LDL-c persistentemente acima de 145 mg/dL, apesar das medidas de estilo de vida.^178 - 188^

Abaixo, seguem recomendações para intervenção terapêutica.

- Pacientes de risco baixo a intermediário: recomenda-se uma redução do LDL-c igual ou superior a 30%.
- Pacientes de risco alto, muito alto ou extremo: a meta terapêutica é uma redução do LDL-c igual ou superior a 50%.

Nesse contexto, recomenda-se preferencialmente o uso de estatinas de alta potência ou a adoção de terapia combinada. Considerando a variabilidade interindividual na resposta às estatinas,^189^ é comum a necessidade de associação com outros agentes hipolipemiantes para atingir as metas propostas.

### 5.2. Recomendações de Metas de acordo com a Estratificação de Risco Cardiovascular ( Figura 5.1 )

**Figura 5.1:**
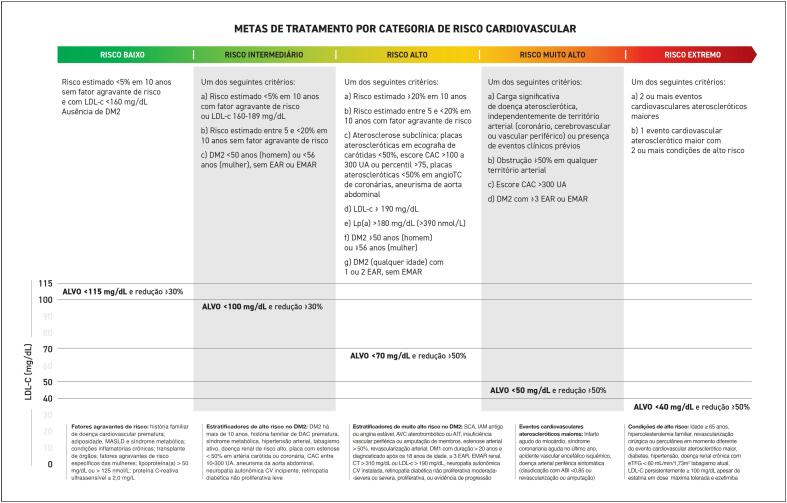
Recomendações de Metas de acordo com a Estratificação de Risco Cardiovascular.

#### 5.2.1. Indivíduos de Risco Baixo

Uma metanálise baseada em dados individuais de ECR com estatinas demonstrou que, em indivíduos categorizados como de baixo risco, houve uma redução absoluta de 11 eventos vasculares maiores por 1.000 pessoas para cada diminuição de 38,7 mg/dL no LDL-c ao longo de 5 anos. Esse benefício supera amplamente quaisquer riscos inerentes à terapia com estatina.^190^

Ainda que estratificados como de baixo risco, qualificam-se para início de terapia hipolipemiante os indivíduos que, afastados fatores secundários, mantêm o LDL-c ≥ 145 mg/dL a despeito de mudanças no estilo de vida.^178 - 188^

Neste cenário, recomenda-se:

- Redução do LDL-c ≥ 30%
- Meta primária: LDL-c < 115 mg/dL
- Meta coprimária: não-HDL-c < 145 mg/dL

#### 5.2.2. Indivíduos de Risco Intermediário

Neste cenário, recomenda-se:

- Redução do LDL-c ≥ 30%
- Meta primária do LDL-c: < 100 mg/dl
- Meta coprimária do não-HDL-c: < 130 mg/dl

#### 5.2.3. Indivíduos de Risco alto

Para indivíduos classificados como de alto risco cardiovascular, esta diretriz recomenda terapia hipolipemiante de alta intensidade. Desse modo, o uso preferencial de estatina de alta potência ou terapia combinada (estatina associada à ezetimiba) é indicado.

Neste cenário, recomenda-se:

- Redução do LDL-c ≥ 50%
- Meta primária do LDL-c < 70 mg/dl
- Meta coprimária do não-HDL-c < 100 mg/dl

#### 5.2.4. Indivíduos de Risco Muito Alto

A meta do LDL-c é < 50 mg/dL, e do não-HDL-c, < 80 mg/dl. Esta recomendação foi fundamentada sobretudo no estudo IMPROVE-IT ( *IMProved Reduction of Outcomes: Vytorin Efficacy International Trial* ), no qual a terapia combinada de sinvastatina com ezetimiba, ao atingir média de LDL-c de 53 mg/dL, promoveu redução adicional do risco cardiovascular quando comparada ao grupo monoterapia (média de LDL-c de 69 mg/dL).^165^

Em pacientes com DCVA estabelecida e, portanto, de muito alto risco cardiovascular, a combinação de estatina de moderada potência com ezetimiba demonstrou ser não inferior ao uso de estatina de alta potência na redução de eventos cardiovasculares. No mesmo estudo, a terapia combinada foi associada a uma maior taxa de alcance das metas recomendadas de LDL-c e à menor proporção de descontinuação da terapia hipolipemiante por intolerância.^191^ Subsequentemente, foi demonstrada redução adicional do risco cardiovascular com o acréscimo de terapia anti-PCSK9 ( *proprotein convertase subtilisin–kexin type 9* ) em portadores de DCVA com dislipidemia residual, a despeito do uso de estatinas, com ou sem ezetimiba. No estudo FOURIER ( *Evolocumab and Clinical Outcomes in Patients with Cardiovascular Disease* ), a redução do LDL-c mediada pelo evolocumabe (mediana atingida de 30 mg/dL) promoveu redução de 15% no risco relativo de eventos cardiovasculares maiores após seguimento mediano de 2,2 anos.^168^ No ODYSSEY OUTCOMES ( *Alirocumab and Cardiovascular Outcomes after Acute Coronary Syndrome* ), o uso de alirocumabe em pacientes pós-síndrome coronária aguda (1 a 12 meses antes da randomização) reduziu os níveis de LDL-c para uma mediana de 53 mg/dL, resultando em redução de 15% no risco relativo de desfechos cardiovasculares clinicamente relevantes em período mediano de acompanhamento de 2,8 anos.^169^

Neste cenário, recomenda-se:

- Redução do LDL-c ≥ 50%
- Meta primária do LDL-c < 50 mg/dl
- Meta coprimária do não-HDL-c < 80 mg/dl

#### 5.2.5. Indivíduos de Risco Cardiovascular Extremo

Pacientes com DCVA, mesmo após um episódio de síndrome coronariana aguda, apresentam risco residual variável para novos desfechos cardiovasculares. Uma subanálise do estudo ODYSSEY OUTCOMES, que estratificou os participantes conforme a categorização de risco proposta pela Diretriz de Dislipidemias do American College of Cardiology/American Heart Association, demonstrou uma taxa de eventos cardiovasculares de 20,4% no subgrupo com histórico de múltiplos eventos prévios, em contraste com 5,6% entre aqueles sem critérios de maior gravidade.^192^ De forma semelhante, diversas subanálises do estudo FOURIER evidenciaram uma incidência aumentada de eventos cardiovasculares em subgrupos com características de maior risco, como: doença coronariana extensa, múltiplos eventos cardiovasculares prévios, níveis elevados de Lp(a), presença de diabetes *mellitus* , doença arterial periférica e doença polivascular. Como era esperado, considerando que o benefício absoluto da intervenção terapêutica é proporcional ao risco basal, esses pacientes obtiveram maior benefício clínico com o alcance de metas lipídicas mais rigorosas e com o uso de terapias de maior custo,^193 - 197^ refletido por um menor número necessário para tratar (NNT) e, consequentemente, melhor relação custo-efetividade. Uma análise secundária do estudo FOURIER demonstrou uma relação linear monotônica entre os níveis de LDL-c alcançados e a incidência de eventos cardiovasculares adversos, com redução progressiva do risco observada até concentrações séricas inferiores a 20 mg/dL. Esses achados reforçam o princípio de que, em termos de prevenção cardiovascular, "quanto mais baixo o LDL-c, melhor".^198^ Ademais, até o presente momento, não há evidências consistentes de eventos adversos associados a níveis muito baixos de LDL-c.^199 - 203^ Com base nesse conjunto de evidências, esta diretriz recomenda, para indivíduos classificados como de risco cardiovascular extremo, metas terapêuticas mais rigorosas: LDL-c < 40 mg/dL e colesterol não-HDL < 70 mg/dL.

Neste cenário, recomenda-se:

- Redução do LDL-c ≥ 50%
- Meta primária do LDL-c > 40 mg/dl
- Meta coprimária do não-HDL-c < 70 mg/dl

### 5.3. Apolipoproteína B

A dosagem dos níveis séricos de ApoB é amplamente reconhecida como a medida mais acurada do risco cardiovascular associado a lipoproteínas aterogênicas. Isso se deve ao fato de que cada partícula lipoproteica potencialmente aterogênica presente na circulação – incluindo LDL, VLDL, Lp(a), remanescentes de VLDL e quilomícrons – contém exatamente uma molécula de ApoB. Assim, a quantificação direta da ApoB reflete com precisão o número total de partículas aterogênicas plasmáticas. Além disso, sua determinação pode ser realizada independentemente do estado de jejum, o que facilita sua aplicação clínica. Vale destacar que os quilomícrons contêm uma isoforma truncada da ApoB, a ApoB-48, enquanto as demais partículas aterogênicas contêm ApoB-100.^7 , 59 , 204 , 205^

Atualmente, o custo da dosagem de ApoB ainda não é coberto pela maioria dos planos de saúde e tampouco está disponível na rede pública por meio do Sistema Único de Saúde. Como alternativa viável, a estimativa do colesterol não-HDL apresenta boa correlação com a concentração sérica de ApoB e pode ser facilmente obtida a partir de qualquer painel lipídico convencional, uma vez que seu cálculo requer apenas a subtração dos níveis de HDL-c da concentração total de colesterol. Essa abordagem representa uma ferramenta prática e acessível para estimar a carga aterogênica em contextos clínicos nos quais a dosagem direta de ApoB não está disponível.^57 , 170 , 171^ Entretanto, metas de ApoB podem ser utilizadas para pacientes que já atingiram suas metas de LDL-c e não-HDL-c. A equivalência entre as metas de LDL-c e de ApoB^206^ encontra-se a seguir:

- LDL-c 40 mg/dL ∼ ApoB 45 mg/dL
- LDL-c 50 mg/dL ∼ ApoB 55 mg/dL
- LDL-c 70 mg/dL ∼ ApoB 70 mg/dL
- LDL-c 100 mg/dL ∼ ApoB 90 mg/dL
- LDL-c 115 mg/dL ∼ ApoB 100 mg/dL

### 5.4. Colesterol da Lipoproteína de Alta Densidade (HDL-c)

Não são propostas metas para o HDL-c. Embora estudos epidemiológicos tenham demonstrado uma forte associação entre baixos níveis de HDL-c e maior incidência de eventos cardiovasculares,^207^ não há evidências robustas que indiquem que essa relação seja causal. Em estudos de randomização mendeliana, indivíduos geneticamente predispostos a apresentar níveis mais elevados de HDL-c não tem risco reduzido de aterosclerose.^208^ Além disso, terapias destinadas a elevar os níveis de HDL-c não apresentaram benefício consistente na redução de eventos cardiovasculares.^209 - 214^ Assim, o HDL-c deve ser utilizado como um marcador de risco cardiovascular, mas não como um alvo terapêutico.

### 5.5. Triglicérides

As lipoproteínas ricas em TG e, especialmente, os remanescentes de seu metabolismo contribuem para o risco residual da doença vascular aterosclerótica. A concentração sérica de TG é um marcador de risco residual aterosclerótico^215 , 216^ e acompanha o aumento na prevalência de condições como obesidade e diabetes tipo 2, situações em que esses níveis se encontram leve a moderadamente elevados.^217 , 218^

Níveis séricos de TG ≥ 150 mg/dL em jejum ou ≥ 175 mg/dL no estado pós-prandial são considerados anormais^173^ e, portanto, a meta a ser atingida é expressa por níveis séricos abaixo destes limites. Entretanto, é imperativo ter em mente que as metas para LDL-c e colesterol não-HDL têm prioridade em pacientes com hipertrigliceridemia leve a moderada.^91^

Além das medidas de estilo de vida, eficazes na redução dos TG, esta diretriz recomenda, quando necessária a terapia medicamentosa, o uso preferencial de fármacos voltados à redução do colesterol não-HDL, como as estatinas de alta intensidade e a ezetimiba. É importante ressaltar que a adequação do controle glicêmico contribui para a redução dos níveis séricos de TG. Nesse particular, fármacos com comprovado benefício clínico, como inibidores da SGLT2 e agonistas dos receptores GLP1 (aGLP1) podem ser usados com o objetivo concomitante de controle glicêmico e redução do risco cardiovascular. Mesmo em não diabéticos, um aGLP1 em doses elevadas (semaglutida 2,4 mg) demonstrou redução dos TG e do risco cardiovascular em pacientes com DCV estabelecida e sobrepeso ou obesidade.^219^

Hipertrigliceridemia grave, definida como TG séricos ≥ 500 mg/dL (ou ≥ 885 mg/dL segundo alguns autores), associa-se a risco de pancreatite aguda. Neste caso, recomenda-se o uso de fármacos mais eficazes na redução dos TG, como os fibratos, e o alvo terapêutico é manter os níveis séricos abaixo de 500 mg/dL.

### 5.6. Lipoproteína(a)

Até o presente momento, não há nenhum tratamento especificamente voltado à redução dos níveis séricos de Lp(a). Estudos prospectivos de fase 3 em andamento poderão fornecer dados consistentes sobre a relação entre a magnitude da redução dos níveis de Lp(a) e seu efeito sobre desfechos cardiovasculares em pacientes de alto ou muito alto risco cardiovascular.^220^

Esta diretriz, no entanto, reforça a importância da avaliação da Lp(a), ao menos uma vez na vida, em todos os indivíduos. Ainda que o impacto da redução da Lp(a) na diminuição do risco seja, até o momento, incerto e que alvos terapêuticos não estejam definidos, a documentação de Lp(a) elevada pode contribuir para a estratificação do risco cardiovascular^221 , 222^ e, desse modo, auxiliar na adoção ou intensificação de estratégias voltadas ao controle dos demais fatores de risco modificáveis.

**Table:** 

Recomendação	Força da recomendação	Certeza da evidência
Em indivíduos de risco cardiovascular extremo, recomenda-se a favor das metas de LDL-c < 40 mg/dL e de não-HDL-c < 70 mg/dL.	FORTE	MODERADA
Em indivíduos de risco cardiovascular muito alto, recomenda-se a favor das metas de LDL-c < 50 mg/dL e de não-HDL-c < 80 mg/dL.	FORTE	ALTA
Em indivíduos de risco cardiovascular alto, recomenda-se a favor das metas de LDL-c < 70 mg/dL e de não-HDL-c < 100 mg/dL.	FORTE	ALTA
Em indivíduos de risco cardiovascular intermediário, recomenda-se a favor das metas de LDL-c < 100 mg/dL e de não-HDL-c < 130 mg/dL.	FORTE	ALTA
Em indivíduos de risco cardiovascular baixo, recomenda-se a favor das metas de LDL-c < 115 mg/dL e de não-HDL-c < 145 mg/dL.	FORTE	MODERADA
Em indivíduos de risco cardiovascular alto, muito alto ou extremo, recomenda-se a favor de uma redução percentual do LDL-c de pelo menos 50%.	FORTE	ALTA
Em indivíduos de risco cardiovascular baixo ou intermediário, recomenda-se a favor de uma redução percentual do LDL-c de pelo menos 30%.	FORTE	ALTA
Em todos os indivíduos, especialmente naqueles com níveis de LDL-c ou não-HDL-c acima da meta, recomenda-se a favor de intervenções em medidas de estilo de vida.	FORTE	ALTA
Em indivíduos de risco cardiovascular alto, muito alto ou extremo, recomenda-se a favor de terapia farmacológica associada a medidas de vida.	FORTE	ALTA
Em indivíduos de risco cardiovascular alto, muito alto ou extremo, com LDL-c ou não-HDL-c persistentemente acima da meta, recomenda-se a favor da intensificação da terapia farmacológica associada a medidas de estilo de vida.	FORTE	ALTA
Em indivíduos de risco cardiovascular baixo ouintermediário, com LDL-c ou não-HDL-c persistentemente ≥ 30 mg/dL acima da meta, recomenda-se a favor do início ou intensificação da terapia farmacológica associada a medidas de estilo de vida.	FORTE	ALTA
Em indivíduos com níveis de LDL-c e não-HDL-c dentro da meta estabelecida, recomenda-se a favor de considerar meta de ApoB para optar sobre intensificação terapêutica.	FORTE	MODERADA

## 6. Tratamento Não Farmacológico

### 6.1. Recomendações de Estilo de Vida para Melhorar o Perfil Lipídico

#### 6.1.1. Aspectos Nutricionais

A tendência recente é de enfatizar uma abordagem qualitativa e não quantitativa de macronutrientes, priorizando padrões alimentares minimamente processados e ricos em fibras, redução do consumo de açúcares adicionados e carboidratos refinados.^223^

Abaixo, estão as evidências a respeito dos principais macronutrientes da dieta.

#### 6.1.2. Carboidratos

No estudo *The Prospective Urban Rural Epidemiology* (PURE), a ingestão elevada de carboidratos (> 60% da energia total) esteve associada a um maior risco de mortalidade por todas as causas.^224^ De modo semelhante, o estudo *Atherosclerosis Risk in Communities* (ARIC) demonstrou que dietas contendo entre 50 e 55% da energia total proveniente de carboidratos foram associadas a menor risco de mortalidade total, e dietas com altos teores de carboidratos refinados (açúcares e farinhas processadas) estiveram associadas a um maior risco de doenças CV e metabólicas.^224 , 225^ Conforme demonstrado em recente metanálise de coortes prospectivas, dietas extremas (< 40% ou > 65% da energia proveniente de carboidratos) estiveram associadas a um aumento da mortalidade geral.^226^

Recomenda-se reduzir ou eliminar açúcares adicionados e farinhas refinadas^224 , 226^ e priorizar alimentos com baixo índice glicêmico (por exemplo, leguminosas, grãos integrais, frutas com casca) e fontes de carboidratos ricos em fibras.^227^

#### 6.1.3. Gorduras

A substituição de gorduras saturadas por insaturadas, como as mono (por exemplo, azeite de oliva e abacate) e poli-insaturadas (como peixes gordurosos e sementes de linhaça), demonstrou redução de LDL-c e prevenção de DCV. Em contrapartida, substituir gorduras saturadas por carboidratos refinados favorece o aumento dos TG e a redução do HDL-c, o que também está associado a um risco cardiovascular aumentado.^55 , 228 , 229^

#### 6.1.4. Fibras Solúveis

As fibras solúveis são caracterizadas por sua capacidade de absorver água e formar um gel viscoso no trato gastrointestinal, o que suprime a formação de micelas e atrasa o esvaziamento gástrico. Isso impacta na redução da absorção de colesterol, no aumento da excreção de ácidos biliares e na modulação da microbiota intestinal, levando a efeitos hipocolesterolêmicos significativos.^230 - 232^

A recomendação é a ingestão mínima de fibras totais de 25 g/dia,^233^ incluindo, por exemplo, β-glucana (aveia),^231^ *psyllium* ; leguminosas e frutas com casca.

**Table:** 

Recomendações dietéticas para o tratamento das dislipidemias	% do valor calórico total (VCT)	Força da recomendação	Certeza da evidência
Gorduras totais	20-35%^228^	FORTE	ALTA
Gorduras saturadas	< 7%^55^	FORTE	ALTA
Gorduras trans	Não ingerir^228^	FORTE	ALTA
Ácidos graxos monoinsaturados	15%^229^	FORTE	ALTA
Ácidos graxos poli-insaturados	5-10%^229^	FORTE	ALTA
Fibras	25 g/dia	CONDICIONAL	MODERADA
Carboidratos totais	50-55%^223 , 224^	FORTE	ALTA

### 6.2. Cessação do Tabagismo

O tabagismo é responsável por 50% de todas as mortes evitáveis em fumantes, sendo metade delas relacionadas à DCV aterosclerótica.^234^ O risco de DCV em fumantes com menos de 50 anos de idade é cinco vezes maior do que em não fumantes e está associado a maior incidência de AVC, doença arterial periférica (DAP) e morte súbita cardíaca.^235^ O tabagismo contribui para o desenvolvimento da aterosclerose por múltiplos mecanismos fisiopatológicos: disfunção endotelial por redução da biodisponibilidade do óxido nítrico (NO) endotelial; aumento da produção de espécies reativas de oxigênio (ERO), promovendo oxidação do LDL-c; aumento dos níveis circulantes de marcadores inflamatórios e estímulo de estado pró-trombótico; além de impactar na diminuição de HDL-c.^236 , 237^ A cessação do tabagismo promove benefícios cardiovasculares imediatos e progressivos, independentemente da idade ou do tempo de tabagismo prévio. Após 1 ano sem fumar, há redução do risco de IAM em aproximadamente 50%.^238^

A abordagem deve ser multifatorial e inclui estratégias comportamentais e farmacológicas. Programas de aconselhamento individual ou em grupo têm eficácia na redução da taxa de tabagismo. A indicação do tratamento farmacológico depende da avaliação do grau de dependência do paciente, sendo que o teste de Fagerström pode ser usado com esse objetivo.^239^ Essa abordagem inclui terapia de reposição de nicotina, por meio da utilização de adesivos, gomas e pastilhas. A bupropiona, um inibidor da recaptação de dopamina e noradrenalina, auxilia na redução dos sintomas de abstinência, contribuindo para um manejo mais eficaz.

**Table:** 

Recomendação	Força da recomendação	Certeza da evidência
Cessação do tabagismo tem impacto na redução no desenvolvimento de aterosclerose e, consequentemente, do risco cardiovascular.	FORTE	ALTA

### 6.3. Controle de Peso

Em pessoas com obesidade, o padrão lipídico é caracterizado por TG elevados, HDL-c baixo e LDL-c de característica pró-aterogênica, pequena e densa. Esses fatores, em associação ao aumento da gordura visceral e à resistência à insulina, enquadram muitos pacientes nos critérios para síndrome metabólica.^240^

O tratamento não farmacológico da obesidade requer uma abordagem constante e centrada no paciente, e estima-se que intervenções comportamentais multiprofissionais necessitam, minimamente, de 6 meses para promover mudanças no estilo de vida. A perda de peso entre 8 e 10% está diretamente relacionada à redução de 5-10 mg/dL de LDL-c; redução de 20-30 mg/dL de TG e aumento de 3-5 mg/dL de HDL-c.^240 , 241^ Essas evidências também foram avaliadas no ensaio *Look Ahead* ^242^ em pacientes com diabetes, sobrepeso ou obesidade. A redução de 5 a 10% de peso em 1 ano foi associada a uma redução de 40 mg/dL de TGs e a um aumento no HDL-c de 5 mg/dL, enquanto a redução no LDL-c foi modesta e não estatisticamente significativa. No entanto, a manutenção da perda de peso a longo prazo é desafiadora, com reganho recorrente após 2 anos de acompanhamento.^243^

A adoção de hábitos alimentares saudáveis desde a infância previne a obesidade e doenças crônicas associadas.^244^ Estudos recentes têm abordado diferentes dietas e comportamentos, com foco na modificação dos hábitos alimentares e no impacto de estratégias como a restrição calórica, que tem se mostrado eficaz para redução do peso corporal em 6 meses.^245 , 246^ Contudo, os efeitos da redução do peso sobre os fatores de risco cardiovascular se dão na maior parte das vezes a longo prazo.^247^ A abordagem multidisciplinar, que envolve psicólogo, nutricionista, educador físico e profissionais da saúde mental, tem-se mostrado eficaz.^248^

**Table:** 

Recomendação	Força da recomendação	Certeza da evidência
A redução do peso através de medidas não farmacológicas é recomendada para aumento dos níveis de HDL-c, redução de triglicérides e redução menos pronunciada de LDL-c.	FORTE	ALTA

### 6.4. Espiritualidade

Há um conjunto de evidências que demonstram forte relação entre espiritualidade, religiosidade e os processos de saúde, adoecimento e cura, compondo junto dos aspectos físicos, psicológicos e sociais a visão integral do ser humano.^249^ Espiritualidade e religiosidade são recursos utilizados pelos pacientes no enfrentamento das doenças e do sofrimento. Entender qual a relevância, identificar demandas e prover adequado suporte espiritual e religioso beneficiam tanto pacientes como a equipe multidisciplinar e o próprio sistema de saúde. A espiritualidade é expressa através de crenças, valores, tradições e prática.^250^

Evidências científicas disponíveis descrevem que religiosidade e espiritualidade estão associadas a menores prevalências de tabagismo, menor consumo de álcool, menor sedentarismo/maior atividade física, melhor adesão nutricional e farmacológica nas dislipidemias, hipertensão arterial, obesidade e diabetes *mellitus* .^251 , 252^

**Table:** 

Recomendação	Força da recomendação	Certeza da evidência
A abordagem espiritualidade e religiosidade deve fazer parte da consulta médica pelo seu impacto em saúde cardiovascular.	CONDICIONAL	MODERADA

### 6.5. Prática de Atividades Físicas

O comportamento sedentário está associado a um risco aumentado de várias doenças crônicas importantes e à mortalidade, enquanto o exercício físico é um componente essencial na prevenção e tratamento das DCV.^253^ O impacto em lipídeos é observado pela elevação dos níveis de HDL-c e benefício moderado nos níveis de LDL-c, embora o efeito nos níveis absolutos de LDL-c seja menos acentuado.^254^ Além disso, há redução consistente da concentração plasmática de TG.^255^

Adultos devem acumular ao menos 150 minutos por semana de atividade física aeróbica de intensidade moderada ou 75 minutos de atividade vigorosa, podendo se beneficiar de períodos de atividade moderada/intensa.^256^

Os exercícios de resistência estão associados a menores riscos de eventos cardiovasculares totais e mortalidade por todas as causas. Recomenda-se de uma a três séries de 8 a 12 repetições em uma intensidade de 60 a 80% da capacidade máxima de uma repetição, com frequência de pelo menos 2 dias por semana, em uma variedade de 8 a 10 exercícios diferentes envolvendo cada grupo muscular principal.^256^

Para adultos mais velhos ou indivíduos em retomada após interrupção da prática física, sugere-se começar com uma série de 10-15 repetições a 40-50% de uma repetição máxima. Além disso, recomenda-se que idosos realizem atividades físicas multicomponentes que combinem exercícios aeróbicos, de fortalecimento muscular e de equilíbrio para prevenir quedas.^257 , 258^

Para adultos fisicamente inativos, atividades físicas de leve intensidade, mesmo que apenas 15 minutos por dia, têm o potencial de trazer benefícios.^257 - 259^

Antes de iniciar um programa de exercícios, recomenda-se uma avaliação clínica. Caso esteja disponível, um teste ergométrico realizado na vigência da medicação cardiovascular pode determinar a intensidade dos exercícios, com base na frequência cardíaca. O American College of Sports Medicine propõe que, para atividades leves a moderadas, a intensidade deva ser de 50 a 70% da frequência cardíaca pico, enquanto atividades moderadas a intensas devem ter uma intensidade de 70 a 85% da frequência cardíaca pico para aqueles já bem adaptados.^260^

**Table:** 

Recomendação	Força da recomendação	Certeza da evidência
Adultos devem acumular ao menos 150 minutos por semana de atividade física aeróbica de intensidade moderada ou 75 minutos de atividade vigorosa, podendo se beneficiar de combiná-las.	FORTE	ALTA

### 6.6. Ingesta Alcoólica

Estudos epidemiológicos sugerem que o consumo elevado está associado a um risco aumentado de AVC, DAC e IC, enquanto se observa uma relação inversa (em escala log-linear) com o risco de infarto do miocárdio.^261^ Estudos de randomização mendeliana sugerem que o menor risco para resultados cardiovasculares é observado em indivíduos abstêmios e que qualquer quantidade de álcool aumentaria a PA e o IMC.^262 , 263^

O limite superior considerado seguro para o consumo de bebidas alcoólicas é de aproximadamente 100 g de álcool por semana, tanto para homens quanto para mulheres.^261^ Estudos sobre biomarcadores mostram que o álcool moderado (principalmente com o vinho tinto) pode elevar o HDL-c e adiponectina, reduzir o fibrinogênio^264 , 265^ e melhorar marcadores de inflamação e hemostasia.^266^ Por outro lado, consumo elevado (> 30 g/dia) associa-se ao aumento de TG e CT. No metabolismo glicídico, o consumo leve de álcool está ligado a menor risco de diabetes tipo 2, com uma relação em U consistente em ambos os sexos; entretanto, essa associação é perdida com doses superiores a 50-60 g/dia e não depende do tipo de bebida.^267 , 268^

Os efeitos deletérios do consumo elevado de álcool são amplamente documentados. Assim, as recomendações devem ser cautelosas, uma vez que conclusões definitivas ainda não foram alcançadas.

**Table:** 

Recomendação	Força da recomendação	Certeza da evidência
Não é recomendado a ingestão de álcool para prevenção e tratamento da aterosclerose.	FORTE	ALTA

### 6.7. Suplementos Dietéticos e Alimentos Funcionais em Dislipidemias

Estes alimentos podem ser considerados como uma estratégia adicional para pacientes que não atingem as metas lipídicas apenas com mudanças no estilo de vida. A evidência clínica é variável e mais estudos bem desenhados são necessários para confirmar a eficácia e segurança desses suplementos.

Os principais alimentos funcionais estudados são:

- **Proteína de soja:** acredita-se que a proteína de soja diminua a absorção de colesterol e aumente a excreção de esteroides fecais.^269^
- **Fitosteróis:** os fitosteróis competem com o colesterol na absorção intestinal. Sua eficácia depende da dose, do nível basal de LDL-c e do veículo alimentar utilizado.^270^ As diretrizes revisadas da European Society of Cardiology e da European Atherosclerosis Society^55^ sobre dislipidemias passaram a recomendar o uso de esteróis vegetais como parte das mudanças no estilo de vida para redução do colesterol. No entanto, evidências genéticas recentes indicam um potencial efeito aterogênico desses compostos, reforçando a necessidade de ensaios clínicos randomizados com desfechos cardiovasculares robustos antes de uma recomendação ampla, conforme destacado pela Deutsche Gesellschaft für Kardiologie.^271^
- **Chá verde:** o consumo de chá verde teria efeitos protetores na saúde vascular, melhorando a função endotelial.^272^ Os mecanismos de ação propostos incluem bloqueio da absorção intestinal de colesterol^228^ e aumento da excreção fecal de ácidos graxos.^273^
- **Gergelim ou sesame:** contém ácidos graxos insaturados, vitamina E e lignanas, que podem ter efeitos favoráveis nos lipídios e na saúde cardiovascular, reduzindo os níveis de TG.^274^
- **Probióticos:** os probióticos podem reduzir os níveis de CT e LDL-c, além de melhorar o IMC e marcadores inflamatórios. Acredita-se que o principal mecanismo de ação seja a diminuição da circulação entero-hepática de sais biliares.^275^ Uma redução significativa de 13,6% nos níveis plasmáticos de CT, 8,4% de LDL-c, 5,5% de aumento no HDL-c, 12,8% de redução na relação LDL-c/HDL-c, 9,0% de redução nos TG e 11,3% de redução no LDL oxidado foram observadas com a utilização de Lactobacillus plantarum.^276^
- **Arroz de levedura vermelha (RYR):** o produto fermentado sintetizado pela levedura Monascus purpureus contém monacolinas, que são os principais compostos bioativos com efeitos hipolipemiantes.277 O arroz vermelho fermentado (RYR) contém monacolina K, um composto semelhante à lovastatina, sendo amplamente utilizado para controle do colesterol. Estudos indicam que o RYR apresenta variações na composição fitoquímica e pode ter efeito sinérgico entre monacolinas e outros compostos, inibindo a HMG-CoA redutase de forma mais eficaz que a lovastatina isolada. Além disso, sua toxicidade celular é menor, especialmente em células musculares. Apesar dos resultados promissores, mais pesquisas são necessárias para compreender seus mecanismos e validar sua eficácia clínica.^278^
- **Óleo de peixe:** rico em ácidos graxos ômega-3 marinhos, como o ácido eicosapentaenoico (EPA) e o ácido docosahexaenoico (DHA), o óleo de peixe é recomendado em diretrizes clínicas para pacientes com hipertrigliceridemia.^279^ O óleo de peixe reduz os níveis de TG ao inibir a síntese e a taxa de renovação de TG.^280^ Os resultados dos estudos específicos para tratamento com ômega 3 serão abordados no capítulo específico de tratamento.

**Table:** 

Recomendação	Força da recomendação	Certeza da evidência
Recomenda-se a favor de considerar suplementação * dietética no manejo das dislipidemias.	CONDICIONAL	MODERADA

* São consideradas suplementações com impacto nos níveis de LDL-c o arroz de levedura vermelho, probióticos, fitoesteróis e para redução de TG, óleo de peixe (EPA/DHA).

## 7. Tratamento Farmacológico

O tratamento das dislipidemias tem passado por uma notável evolução nas últimas décadas. Embora os fatores de risco lipídicos para DCV dependam em parte do estilo de vida, o ótimo controle dos lipídios frequentemente exige intervenções farmacológicas adicionais. Tradicionalmente, as estatinas têm sido a base do tratamento, com eficácia comprovada na redução dos níveis de LDL-c e na prevenção de eventos cardiovasculares. Associadas a outras medicações orais, como ezetimiba e, mais recentemente, inibidores da ATP citrato liase, essas terapias têm oferecido controle adicional do perfil lipídico.

Contudo, o avanço da biotecnologia possibilitou o desenvolvimento de terapias injetáveis de longa duração, como a terapia anti-PCSK9 e os RNA-interferentes, que permitiram uma redução ainda mais significativa do LDL-c, com conveniência posológica e boa tolerabilidade. Paralelamente, novas terapias têm emergido, com foco em alvos específicos como a Lp(a), a apolipoproteína C-III e a proteína angiopoietina-like 3, expandindo as possibilidades terapêuticas para além do LDL-c.

Mais recentemente, a terapia gênica e as abordagens de edição genética começaram a se delinear como uma nova fronteira no manejo das dislipidemias, com potencial para modificar permanentemente vias metabólicas envolvidas na regulação lipídica. Esses avanços refletem um novo paradigma no tratamento da dislipidemia, permitindo intervenções cada vez mais personalizadas e eficazes na prevenção de DCV ateroscleróticas.

**Figura 7.1:**
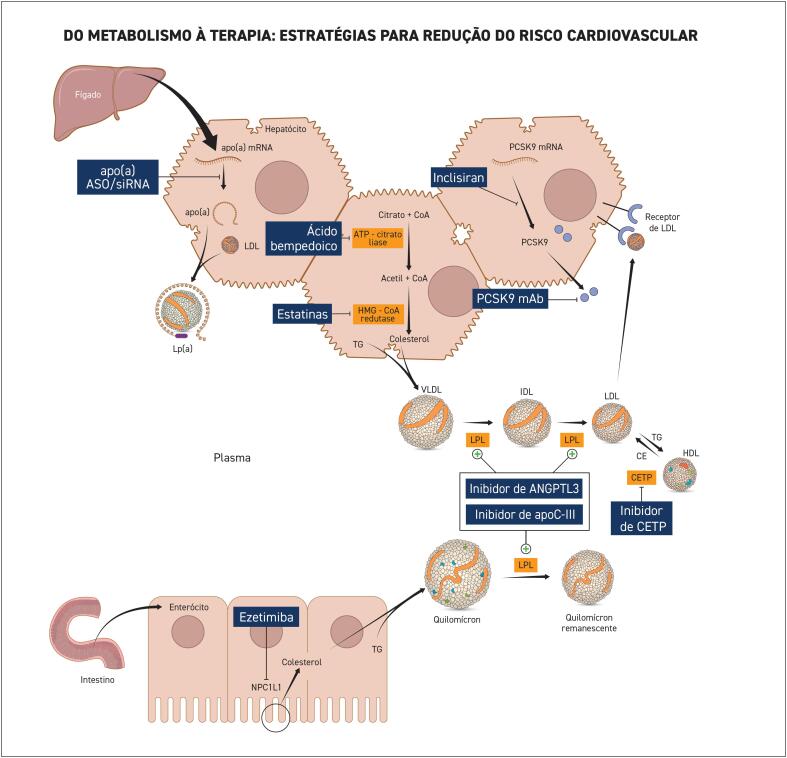
Alvos moleculares dos principais tratamentos para a dislipidemia. CE: colesterol esterificado; LPL: lipoproteína lipase; TG: triglicerídeos.

A Figura 7.1 ilustra os alvos moleculares chave para intervenções terapêuticas na dislipidemia.

### 7.1. Estatinas

A redução do LDL-c pelas estatinas permanece sendo a terapia mais validada para diminuir a incidência de eventos cardiovasculares e a mortalidade, tanto em prevenção primária quanto secundária.^55^ Estatinas são inibidores competitivos do 3-hidroxi-3-meillglutaril coenzima A (HMG-CoA) redutase, que afeta a etapa de controle da taxa de síntese do colesterol. A inibição da HMG-CoA redutase leva a um aumento da expressão do receptor hepático de LDL (LDLR) e aumento da depuração do LDL da circulação. Da mesma maneira, a ação das estatinas pode potencialmente influenciar todo o conjunto das lipoproteínas circulantes que interagem com o LDLR, como a LDL, a VLDL e os remanescentes de quilomícrons.

O grau de redução do LDL-c é dependente da dose e varia entre as diferentes estatinas. Uma terapia de alta intensidade é baseada em uma estatina potente utilizada em altas doses e que, em média, reduz o LDL-c em 50% ou mais; já a terapia de moderada intensidade é definida como aquela onde é esperada uma redução do LDL-c entre 30 e 50%.^37^ Importante ressaltar que existe uma variação interindividual considerável na redução do LDL-c com a mesma dose do medicamento. Respostas deficientes ao tratamento com estatinas podem ser explicadas por variabilidade genética, mas também podem ser causadas por má adesão ao tratamento.

Metanálises de estudos randomizados e controlados demonstram que, para cada redução de 39 mg/dL no nível de LDL-c, estatinas diminuem a mortalidade por todas as causas em 10% e conferem reduções relativas nos eventos cardiovasculares (síndrome coronária aguda, morte coronária, necessidade de revascularização do miocárdio ou AVC) em torno de 22%.^281^ Além disso, estatinas mais potentes conferem uma redução nos eventos cardiovasculares cerca de 15% superior àquela observada com regimes menos potentes.

Com base nessas evidências, a estatina é indicada como a primeira opção terapêutica para reduzir risco cardiovascular em pacientes com dislipidemia. Recomenda-se repetir o perfil lipídico cerca de 4 semanas após o início do tratamento ou após o ajuste de dose das estatinas. Após o atingimento das metas lipídicas, sugere-se repetir o perfil lipídico anualmente, com exceção dos casos em que há suspeita de má aderência ou efeitos adversos.

Efeitos colaterais são incomuns. Sintomas musculares são os mais frequentes: uma metanálise recente, que incluiu mais de 180 estudos observacionais ou randomizados, observou que o percentual de miopatia secundária ao uso de estatinas é de 9%.^282^ Tais sintomas, quando ocorrem, geralmente aparecem nas primeiras semanas de tratamento, embora possam surgir anos após o início da medicação. Importante ressaltar que, na maioria das vezes, irão desaparecer poucos dias após a suspensão da estatina. Variam desde mialgia, com ou sem elevação da creatinoquinase (CK), até a rabdomiólise. No entanto, o risco de grave injúria muscular é inferior a 0,1%. A dosagem de CK deve ser avaliada antes do início do tratamento, mas sua verificação rotineira não é recomendada após o início do uso de estatina, exceto se ocorrerem sintomas musculares (dor, sensibilidade, rigidez, cãibra, fraqueza e fadiga localizada ou generalizada) ou introdução de fármacos que possam interagir.

Estatinas geralmente podem ser utilizadas em indivíduos com hepatopatia leve a moderada, já que o risco de injúria hepática grave é muito baixo. A avaliação basal das enzimas hepáticas, especialmente alanina aminotransferase (TGP), é recomendada antes do início da terapia com estatina. A partir daí, não há recomendação para verificação regular, a menos que ocorram sintomas ou sinais sugerindo hepatotoxicidade (fadiga ou fraqueza, perda de apetite, dor abdominal, urina escura ou aparecimento de icterícia). Elevações leves da TGP (até três vezes o valor de referência) ocorrem em 0,5 a 2,0% dos pacientes que fazem uso de estatinas, mais frequentemente associada ao uso de drogas potentes ou em altas doses. Nesse contexto, não é recomendado suspender a medicação, mas as enzimas hepáticas devem ser reavaliadas em 4 a 6 semanas. Por outro lado, quando houver elevação significativa da TGP (acima de três vezes o valor de referência, em duas ocasiões consecutivas) recomenda-se suspender a estatina ou reduzir a dose.

Tem sido observado um aumento da glicemia e maior risco de início de DM tipo 2 secundário ao uso de estatinas. Uma elevação menor, não clinicamente relevante, da hemoglobina glicada (HbA1c) também pode ocorrer. O risco é maior naqueles que fazem uso de estatinas mais potentes e em doses elevadas, bem como em idosos e em indivíduos com fatores de risco para diabetes, como excesso de peso ou resistência à insulina. A estimativa é que, para que houvesse um novo caso de diabetes, seria necessário tratar 255 pessoas com estatinas pelo período de 4 anos. Dessa maneira, fica claro que a redução absoluta do risco de DCV nesses pacientes supera os possíveis efeitos adversos de um pequeno aumento na incidência de diabetes.^283^

### 7.2. Ezetimiba

A ezetimiba é um inibidor seletivo da proteína NPC1L1, responsável pela absorção intestinal de colesterol. Quando combinada às estatinas, oferece redução adicional do LDL-c, contrabalançando o aumento compensatório da absorção intestinal de colesterol induzido pela inibição da síntese hepática.

Um ensaio clínico randomizado evidenciou que a adição de ezetimiba à sinvastatina em pacientes com síndrome coronariana aguda reduziu eventos cardiovasculares.^165^ Foram randomizados 18.144 pacientes com síndrome coronariana aguda para sinvastatina isolada ou em associação com ezetimiba, sendo acompanhados por uma mediana de 6 anos. A combinação resultou em uma redução significativa de eventos cardiovasculares maiores ( *hazard ratio* 0,936; IC95% 0,89-0,99; p = 0,016), proporcional à redução adicional de LDL-c (∼17 mg/dL). O benefício foi mais pronunciado em subgrupos de maior risco, como pacientes com diabetes e idade avançada.^284^ Além disso, a ezetimiba é uma opção eficaz, segura e de baixo custo em pacientes com intolerância parcial ou total às estatinas, sendo preferível a terapia anti-PCSK9 em diversos contextos clínicos, principalmente por questões de custo e adesão.

A combinação de uma dose baixa de estatina potente (rosuvastatina 10 mg) com ezetimiba foi não inferior à monoterapia com rosuvastatina 20 mg em reduzir eventos cardiovasculares no estudo RACING; além disso, a combinação foi associada a menor taxa de descontinuação.^191^ Estudos reforçam a boa tolerabilidade da combinação de ezetimiba com rosuvastatina.^285^

### 7.3. Novas Abordagens que Têm como Alvo o RNA Mensageiro

Uma modalidade terapêutica inovadora, que tem como alvo o RNA mensageiro (RNAm) para reduzir a síntese de proteínas, já está sendo utilizada para reduzir a síntese da proproteína convertase subtilisina/quexina tipo 9 (PCSK9) hepática e está sendo testada em ensaios clínicos no tratamento de outras dislipidemias, como lipoproteínas ricas em triglicérides (TG) e para reduzir a Lp(a). Em relação às terapias tradicionais, esse tratamento baseado no RNAm possui vantagens, como a possibilidade de atuação específica na síntese da proteína de interesse e a aplicação em longos intervalos, de até 6 meses. As duas principais classes de drogas para reduzir a produção de proteínas pela ação no RNAm são:

1. os oligonucleotídeos anti-sentido de fita simples (ASO, do inglês *antisense oligonucleotides* );
2. os RNA interferentes pequenos de fita dupla (siRNA, do inglês *small interfering RNA* ).

Ambas as classes de medicações são administradas por via parenteral, atuando no citoplasma ou núcleo das células do tecido alvo, com ligação específica a uma sequência no RNAm de interesse. Essa ligação leva à destruição do RNAm alvo, com redução da tradução da proteína codificada.

### 7.4. Terapia anti-PCSK9

A terapia anti- PCSK9 foram a primeira classe de fármacos a reduzir drasticamente os níveis de LDL-c em usuários de estatinas. A principal função da proteína PCSK9 é degradar os receptores de LDL nos hepatócitos. Quando a PCSK9 é inibida, os receptores de LDL intactos retornam à superfície celular, capturando mais partículas de LDL da circulação e diminuindo os níveis de LDL-c.

Entre a terapia anti-PCSK9, os anticorpos monoclonais evolocumabe e alirocumabe, aplicados de forma subcutânea a cada 2 a 4 semanas, reduzem o LDL-c em cerca de 55 a 60% em usuários de estatinas.^168 , 169^ Em dois ensaios clínicos que incluíram pacientes com doença aterosclerótica em uso de estatinas, evolocumabe e alirocumabe reduziram a taxa de eventos cardiovasculares maiores em 15%, com benefícios mais acentuados após o primeiro ano de tratamento.^168 , 169^ Pacientes que atingiram níveis extremamente baixos de LDL-c apresentaram benefícios cardiovasculares ainda mais pronunciados.^198 , 286^ Não foram identificados efeitos colaterais significativos ou alterações cognitivas em até 8,4 anos de seguimento, mesmo em pacientes com LDL-c abaixo de 25 mg/dL.^203 , 286 , 287^

Apesar desses resultados, as terapias hipolipemiantes continuam subutilizadas, ressaltando a necessidade de estratégias alternativas para promover maior adesão. Nesse contexto, a inclisirana, um siRNA que inibe a produção da PCSK9, pode oferecer conveniência posológica por ser administrado a cada 6 meses, de forma subcutânea, em um centro de saúde. A inclisirana proporciona uma redução do LDL-c em aproximadamente 50 a 55% em usuários de estatinas, uma redução substancial e sustentada, sem efeitos colaterais significativos com a medicação.^288^ Estudos em andamento visam a avaliar se o uso de inclisirana se traduz em redução de desfechos cardiovasculares em pacientes com doença aterosclerótica estabelecida.

Embora ainda não disponham de estudos com desfechos cardiovasculares duros publicados, sua eficácia na redução sustentada do LDL-c justifica sua inclusão entre as terapias anti-PCSK9. Considerando a relação causal estabelecida entre níveis de LDL-c e risco cardiovascular, essa classe pode ser considerada uma opção válida no manejo de pacientes de alto risco. Contudo, recomenda-se distinção nos níveis de evidência.

A despeito da eficácia e segurança comprovadas das terapias anti-PCSK9, o alto custo do tratamento pode inviabilizar seu uso, especialmente no Brasil. Nesse sentido, sugere-se priorizar o uso de terapia anti-PCSK9 em pacientes com risco alto de eventos ateroscleróticos ou intolerantes à estatina.

### 7.5. Ácido Bempedoico

O ácido bempedoico é um agente hipolipemiante empregado no tratamento da hipercolesterolemia, especialmente em pacientes que apresentam intolerância às estatinas ou que requerem terapias complementares para alcançar os objetivos lipídicos desejados.^289 , 290^ O ácido bempedoico é o primeiro medicamento oral dentro da categoria dos inibidores da ATP-citrato liase hepática (ACL).^291^ Este fármaco se destaca por sua capacidade de inibir a biossíntese do colesterol, atuando de maneira semelhante às estatinas, mas em uma etapa metabólica anterior no processo. Ao interferir precocemente na via metabólica do colesterol, o ácido bempedoico promove uma redução eficaz dos níveis de LDL-c. Essa redução é alcançada por meio da regulação da síntese de receptores de LDL, consequência da diminuição da concentração intracelular de colesterol.

Uma das principais vantagens do ácido bempedoico é sua aplicabilidade em pacientes que apresentam intolerância ou resposta inadequada às estatinas, oferecendo uma alternativa viável e eficaz no manejo da hipercolesterolemia.^292^ Além disso, a sua atuação em uma etapa metabólica distinta pode potencializar os efeitos de terapias combinadas, proporcionando um controle mais abrangente dos níveis lipídicos. Essa característica torna o ácido bempedoico um componente valioso na estratégia de tratamento das dislipidemias, especialmente em casos que requerem intervenções adicionais para atingir metas previamente propostas.

O estudo CLEAR Outcomes investigou a eficácia do ácido bempedoico na redução do risco cardiovascular em pacientes intolerantes a estatinas.^293^ Este ensaio clínico randomizado, duplo-cego e controlado por placebo incluiu 13.970 pacientes que não podiam ou não queriam tomar estatinas devido a efeitos adversos inaceitáveis. Os pacientes foram designados para receber ácido bempedoico 180 mg diariamente ou placebo. Após um seguimento médio de 40,6 meses, o ácido bempedoico reduziu significativamente os níveis de colesterol LDL em comparação com o placebo, com uma diferença de 29,2 mg/dL. Além disso, a incidência de eventos cardiovasculares adversos maiores foi significativamente menor no grupo do ácido bempedoico em comparação com o grupo placebo (11,7% *versus* 13,3%; razão de risco 0,87; IC95% 0,79-0,96; p = 0,004). O benefício do medicamento foi proporcional à queda do LDL-c, de forma similar ao encontrado nos estudos com estatinas.

### 7.6. Inibidores da Proteína de Transferência de Ésteres de Colesterol e Terapias para Aumentar o Colesterol HDL

Ensaios clínicos randomizados que visaram a elevar os níveis de HDL-c geraram resultados predominantemente nulos. Inibidores da proteína de transferência de ésteres de colesterol (CETP) elevaram significativamente o HDL-c, mas não reduziram eventos cardiovasculares além da magnitude esperada pela redução do LDL-c.^209 - 212^ Da mesma forma, niacina elevou os níveis de HDL-c, mas não reduziu eventos cardiovasculares.^213 , 214^ Mais recentemente, o obicetrapibe, um inibidor de CETP de última geração, oral, de baixa dose e administração diária, em desenvolvimento para o tratamento da dislipidemia, do risco cardiovascular e da doença de Alzheimer, está revertendo a maré de resultados amplamente negativos para a inibição de CETP e está a caminho de ser o primeiro inibidor de CETP disponível para uso clínico. Diferentemente de compostos anteriores da classe, demonstrou reduções significativas de LDL-c, não-HDL-C, ApoB, Lp(a) e partículas pequenas de LDL, além de elevação de HDL funcional (pré-β HDL, ApoA-1, ApoE).

No estudo BROADWAY (fase 3), em pacientes com DCVA ou HeFH sob terapia máxima, reduziu o LDL-c em ∼30%.^294^ O BROOKLYN (fase 3) mostrou reduções de 36–41% em HeFH, com mais de 50% atingindo LDL-c <70 mg/dL.^295^ O PREVAIL (fase 3, em andamento) avalia impacto sobre desfechos cardiovasculares maiores em >9.500 pacientes com DCVA e LDL-c não controlado.

Mais recentemente, uma intervenção que buscava melhorar a função do HDL, e não apenas os níveis séricos de HDL-c, também não reduziu eventos cardiovasculares.^296^ Não há evidências de que aumentar o HDL-c reduza a incidência de eventos cardiovasculares.

### 7.7. Fibratos

Fibratos estimulam os receptores nucleares alfa ativados por proliferadores dos peroxissomas (PPAR-α). Esse estímulo leva ao aumento da produção e da ação da LPL, responsável pela hidrólise intravascular dos TG, e à redução da ApoC-III, responsável pela inibição da LPL. Esse estímulo também leva a maior síntese da ApoA-I e, consequentemente, de HDL. Reduz as taxas séricas de TG de 30 a 60%.

Embora dois grandes estudos clínicos realizados antes da chegada das estatinas ao mercado, o Helsinki Heart Study^297^ e o VA-HIT,^298^ tenham demonstrado redução significativa do risco cardiovascular com o uso de genfibrozila, o mesmo não ocorreu com o uso de fenofibrato nos estudos FIELD^299^ e ACCORD^300^ ou com bezafibrato no estudo BIP.^301^ O estudo ACCORD avaliou o uso de fenofibrato, em comparação a placebo, em uma população de diabéticos dislipidêmicos que já estavam em uso de sinvastatina. Não houve redução de eventos cardiovasculares fatais, infarto do miocárdio ou AVC.^300^ Entretanto, uma subanálise do grupo de 941 pacientes com hipertrigliceridemia (TG ≥ 204 mg/dL) e HDL-c baixo (HDL-c < 35 mg/dL) evidenciou redução de eventos cardiovasculares nos pacientes que receberam fibrato. Outros estudos mostraram benefício especificamente nesse subgrupo com TG alto e HDL-c baixo. Entretanto, era necessário um estudo clínico randomizado que avaliasse especificamente essa população.

O estudo PROMINENT avaliou o uso de pemafibrato, um fibrato mais potente tanto na redução de TG quanto no incremento de HDL-c, na redução de desfechos cardiovasculares em 10.497 pacientes diabéticos, com TG entre 200 e 499 mg/dL e HDL-c ≤ 40 mg/dL. Mais de 95% dos pacientes estavam em uso de estatina, a maioria com tratamento de alta intensidade. Essa nova droga não demonstrou uma redução significativa nos eventos cardiovasculares em comparação com o placebo, apesar de uma redução nos níveis de TG e de outras lipoproteínas.^302^ Portanto, apesar das evidências vindas de análises *post hoc* ou análises secundárias de outros grandes estudos com fibratos, o estudo PROMINENT deixou claro não haver indicação formal para o uso de fibratos na redução do risco cardiovascular em pacientes de alto risco já em uso de estatinas.

Por outro lado, os fibratos continuam sendo recomendados como primeira linha de tratamento para pacientes com TG ≥ 500 mg/dL, com o objetivo principal de reduzir o risco de pancreatite aguda. Nessa população, a redução de TGs com fibratos pode ser expressiva, e o tratamento deve ser iniciado mesmo na ausência de evidência robusta de benefício cardiovascular. O fenofibrato é preferido quando há necessidade de associação com estatinas, devido ao menor risco de miopatia. Com genfibrozila, essa associação é proscrita, dada a alta incidência de efeitos adversos musculares graves, como rabdomiólise.^303^

### 7.8. Ômega-3

Os ácidos graxos ômega-3, especialmente o EPA e o DHA, têm sido amplamente estudados por sua capacidade de reduzir os níveis de TG no sangue. No entanto, os benefícios clínicos desses ácidos graxos variam conforme a população estudada, a dose administrada e a formulação utilizada.

O estudo REDUCE-IT demonstrou que o icosapenta etila (IPE), uma forma purificada de EPA, reduziu significativamente eventos cardiovasculares maiores em pacientes com doença cardiovascular estabelecida ou DM tipo 2 em uso de estatinas e com níveis de TG em jejum entre 135 e 499 mg/dL.^304^ Nesse estudo, a redução de risco cardiovascular foi desproporcional à redução de TG, indicando mecanismo alternativo de redução de risco. A formulação de IPE na dose diária de 4 g não está disponível no Brasil.

Em contraste, o estudo STRENGTH, que avaliou uma combinação de EPA e DHA em pacientes de alto risco cardiovascular, não mostrou redução significativa nos eventos cardiovasculares, suscitando debates sobre as diferenças entre as formulações purificadas e combinadas.^305^ Outros ensaios que utilizaram suplementos genéricos de EPA e DHA apresentaram resultados inconsistentes na redução de eventos cardiovasculares, apesar de reduzirem os níveis de TG.^306 - 310^

Para pacientes com TG ≥ 500 mg/dL, que não foram incluídos no REDUCE-IT, o uso de ômega-3 tem respaldo principalmente na redução do risco de pancreatite. Estudos anteriores demonstraram que doses de 2 a 4 g/dia de EPA + DHA podem reduzir os níveis de TG em até 45% nessa população. Embora os benefícios cardiovasculares diretos ainda não estejam bem estabelecidos nesse grupo, diretrizes como as da American Heart Association e da Endocrine Society recomendam o uso de ômega-3 como parte da estratégia terapêutica para controle lipídico e prevenção de complicações metabólicas.^311 , 312^

Em geral, os ômega-3 apresentam um perfil de segurança favorável, com efeitos adversos geralmente leves, como desconforto gastrointestinal e sabor residual. No entanto, podem apresentar efeitos antiplaquetários, o que exige monitorização em combinação com outros tratamentos antitrombóticos, e aumento na incidência de fibrilação atrial.

### 7.9. Inibidores da Apolipoproteína C-III

A apolipoproteína C-III (ApoC-III) é uma glicoproteína que desempenha um papel fundamental na regulação dos níveis de TG. A ApoC-III inibe a atividade da LPL, enzima responsável pela hidrólise dos TG, e diminui a depuração hepática das lipoproteínas ricas em TG, resultando em um aumento dos TG séricos. Variantes de perda de função no gene que codifica a ApoC-III estão associadas a níveis reduzidos de TG e a menor risco cardiovascular.^313 , 314^

O ASO volanesorsena atua no RNA mensageiro e impede a síntese da proteína ApoC-III, reduzindo os níveis de TG. Em pacientes com TG ≥ 500 mg/dL de diferentes etiologias, a volanesorsena reduziu a chance de pancreatite em 82%, além de promover uma redução relativa de esteatose hepática em 24 a 53%.^315 , 316^ Apesar de resultados promissores, a trombocitopenia emergiu como evento adverso frequente.^317^ Atualmente, o fármaco está aprovado no Brasil para adultos com síndrome da quilomicronemia familiar (SQF), uma condição caracterizada por mutações em genes relacionados à LPL.

Recentemente, a olezarsena, ASO de nova geração, também demonstrou eficácia em pacientes com SQF, reduzindo os níveis de TG em até 44% em relação a placebo e diminuindo o risco de pancreatite em 88%.^318^ Em pacientes com hipertrigliceridemia predominantemente moderada, a olezarsena reduziu os níveis de TG em 49 a 53% em relação a placebo.^319^

Já a plozasirana, um siRNA que impede a síntese da ApoC-III, diminuiu os níveis de TG em até 62% em relação a placebo em pacientes com hipertrigliceridemia moderada ou grave, e reduziu a chance de pancreatite em 83% em pacientes com quilomicronemia.^320 - 322^ Nesses estudos iniciais, eventos adversos atribuídos à olezarsena ou à plozasirana foram incomuns, sendo os mais notáveis reação no local da injeção subcutânea, elevação discreta de transaminases e, no caso da plozasirana, hiperglicemia. Trombocitopenia não foi reportada como evento adverso relacionado a essas medicações. Tanto a olezarsena quanto a plozasirana estão em fase de testes e não estão aprovadas para uso no Brasil.

### 7.10. Inibidores da Proteína Angiopoietina-like 3

A proteína angiopoietina-like 3 (ANGPTL3) é um inibidor endógeno da lipase lipoproteica. As proteínas da família ANGPTL são consideradas relevantes para o metabolismo lipoproteico e, assim, têm sido pesquisadas como alvo farmacológico para possível redução do risco cardiovascular aterosclerótico. Sua perda de função está associada a menor risco de DAC, enquanto o ganho de função associou-se a maior risco de DAC.^323^ A perda de função do gene da ANGPTL3 está associada a reduções de TG, LDL-c e HDL-c. A redução na concentração de LDL-c ocorre de forma independente do receptor de LDL.

O evinacumabe é um anticorpo monoclonal que atua na inibição da ANGPTL3, para reduzir a concentração de lipoproteínas remanescentes ricas em TG, com potencial aterogênico. Como não depende do receptor de LDL para reduzir o LDL-c, pode ser utilizado no tratamento auxiliar na redução dos níveis de LDL-c em pacientes com HoFH.^324 , 325^ Essa condição genética rara resulta em níveis extremamente elevados de LDL-c desde a infância, aumentando significativamente o risco de DCV precoces. Dessa forma, o evinacumabe é aprovado para o tratamento de pacientes adultos e pediátricos a partir dos 5 anos de idade com HoFH que não atingem a meta de LDL-c apesar do uso das doses máximas de terapias hipolipemiantes. Sua posologia é de 15 mg por quilograma de peso corporal, administrado por infusão intravenosa ao longo de 60 minutos a cada 4 semanas. Seus efeitos adversos mais comuns incluem sintomas gripais, como febre e dor de cabeça, reações no local da infusão e sintomas gastrointestinais.

Estudo recente com um ASO foi interrompido por aumento das enzimas hepáticas e acúmulo de gordura hepática.^326^ Outros três estudos com siRNA estão em desenvolvimento.

### 7.11. Inibidores da Lipoproteína(a)

A apolipoproteína(a) (apo(a)) é um componente da Lp(a), a qual está relacionada a IAM, AVC e estenose valvar aórtica. Até o momento, não existe um tratamento específico aprovado para reduzir os níveis de Lp(a). As principais estratégias em desenvolvimento são baseadas em terapias silenciadoras de RNA, com destaque para os ASO e os siRNA, que visam a reduzir a produção hepática da apo(a).

### 7.12. CRISPR e Terapias Gênicas

Tecnologias de edição genética, como CRISPR-Cas9, estão sendo exploradas como abordagens de "cura genética" para dislipidemias hereditárias. Em modelos animais, a edição do gene PCSK9 com uma única aplicação resultou em reduções sustentadas de LDL-c (> 60%) por mais de 1 ano. Embora ainda experimental, essa tecnologia pode representar uma revolução no tratamento das dislipidemias.

**Table:** 

Recomendação	Força da recomendação	Certeza da evidência
Em indivíduos com indicação de terapia hipolipemiante, recomenda-se a favor da estatina como primeira opção de tratamento.	FORTE	ALTA
Em indivíduos sem tratamento com meta de redução de LDL-c de 50% ou mais, recomenda-se a favor da associação de estatina com ezetimiba como alternativa à estatina de alta intensidade.	FORTE	ALTA
Em indivíduos que não atingem o alvo a despeito de estatina em dose máxima tolerada, recomenda-se a favor de intensificação terapêutica com ezetimiba ou terapia anti-PCSK9.	FORTE	ALTA
Recomenda-se a favor do uso de inclisirana como alternativa aos anticorpos monoclonais inibidores da PCSK9, como evolocumabe ou alirocumabe.	FORTE	MODERADA
Em indivíduos intolerantes a estatinas e que não atingem o alvo a despeito de ezetimiba, recomenda-se a favor de intensificação terapêutica com ácido bempedoico.	FORTE	ALTA
Em indivíduos com diagnóstico de Hipercolesterolemia Familiar homozigótica que não atingem a meta de LDL-c apesar do uso das doses máximas de terapias hipolipemiantes, recomenda-se a favor do uso de evinacumabe a partir dos 5 anos de idade.	FORTE	MODERADA
Recomenda-se contra o tratamento farmacológico com o objetivo de aumentar o HDL-c.	FORTE	ALTA
Em indivíduos com hipertrigliceridemia (≥ 150 mg/dL) em que a terapia hipolipemiante é indicada para redução de risco cardiovascular, recomenda-se a favor das estatinas como terapia de escolha.	FORTE	ALTA
Em indivíduos com triglicérides entre 150 e 499 mg/dL que possuem DCVA ou alto risco cardiovascular, recomenda-se a favor de icosapenta etila (4 g/dia) para reduzir a incidência de eventos cardiovasculares maiores, embora não esteja disponível no Brasil.	CONDICIONAL	MODERADA
Em indivíduos com triglicérides entre 150 e 499 mg/dL que possuem DCVA ou alto risco cardiovascular, recomenda-se contra formulações de EPA com DHA para prevenir eventos cardiovasculares.	FORTE	ALTA
Em indivíduos com triglicérides ≥ 500 mg/dL de forma persistente a despeito de medidas de estilo de vida, recomenda-se a favor do uso de fibrato para reduzir o risco de pancreatite.	FORTE	MODERADA
Em adultos portadores da Síndrome da Quilomicronemia Familiar (SQF) com triglicérides ≥ 500 mg/dL, recomenda-se a favor de uso da volanesorsena para reduzir risco de pancreatite	FORTE	MODERADA

DCVA: doença cardiovascular aterosclerótica; LDL-c: colesterol da lipoproteína de baixa densidade; PCSK9: proproteína convertase subtilisina/quexina tipo 9; SQF: Síndrome da Quilomicronemia Familiar.

### 7.13. Terapia Combinada

A estratégia de tratamento usualmente proposta para atingir as metas de LDL-c baseia-se no uso escalonado de terapias hipolipemiantes: inicia-se com estatina, geralmente de alta intensidade, na mais alta dose recomendada ou tolerada, seguida da adição sequencial de ezetimiba e, se necessário, de terapia anti-PCSK9. No entanto, há um grande distanciamento entre as recomendações das diretrizes e a prática clínica, e registros apontam baixa taxa de controle do LDL-c, inércia terapêutica e má adesão ao tratamento.^25 , 327^ Diante desse desafio, o paradigma do tratamento tem migrado do conceito de *estatinas* de alta intensidade para o de *tratamento hipolipemiante* de alta intensidade, com ênfase na combinação terapêutica desde o início.^328 , 329^ Essa abordagem visa a reduzir o risco cardiovascular de forma mais rápida e eficaz, especialmente em indivíduos de maior risco cardiovascular.

Estudos demonstram que a eficácia na redução do LDL-c pode ser significativamente ampliada com o uso de terapias combinadas:

- **Estatina de alta potência + Ezetimiba:** Redução de LDL-c de até 65%, sendo uma estratégia amplamente recomendada em pacientes de alto risco.
- **Estatina de alta potência + Ezetimiba + anti-PCSK9:** Reduções superiores a 85%, representando o regime mais potente disponível atualmente.
- **Estatina de moderada potência + Ezetimiba:** Quando estatina de alta potência não é tolerada, a combinação de estatina de intensidade moderada com ezetimiba pode alcançar reduções de 20–25% adicionais.
- **Estatina de moderada potência + ácido bempedoico + Ezetimiba:** Alternativa eficaz para pacientes intolerantes a estatinas de alta intensidade, com reduções de até 60%.
- **Ácido bempedoico + Ezetimiba + anti-PCSK9:** Estratégia não estatínica que pode atingir reduções superiores a 75%, especialmente útil em casos de intolerância ou contraindicação ao uso de estatinas.

**Figura 7.2:**
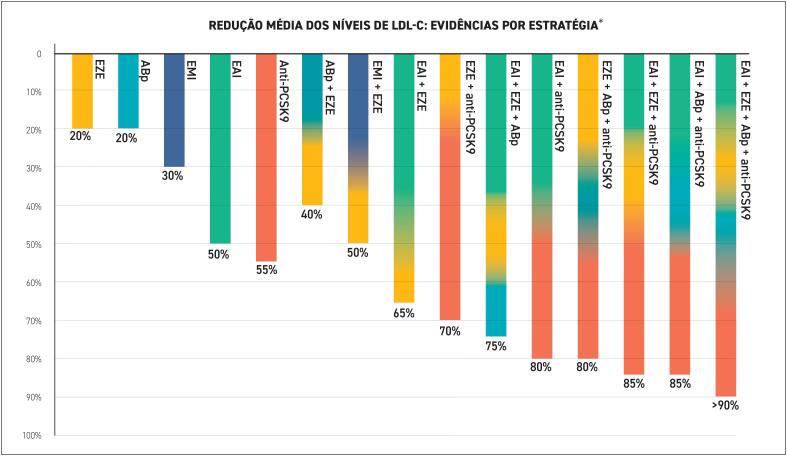
Eficácia da terapia hipolipemiante na redução do LDL-c.^78^ EMI: estatina de moderada intensidade; EAI: estatina de alta intensidade; EZE: ezetimiba; ABp: ácido bempedóico; anti-PCSK9: terapia anti-proteína convertase subtilisina/queuxina tipo 9. *Valores aproximados.

Outras combinações terapêuticas também são possíveis, conforme ilustrado na Figura 7.2 , que apresenta diferentes esquemas de associação entre agentes hipolipemiantes, adaptados às necessidades clínicas e ao perfil de tolerância dos pacientes.

Essas combinações refletem o conceito de tratamento hipolipemiante de alta intensidade, que prioriza intervenções farmacológicas sinérgicas desde o início, com o objetivo de superar barreiras clínicas e otimizar o controle lipídico em populações de risco elevado.

#### 7.13.1. Benefícios da Terapêutica Combinada

Registros demonstram que a maioria dos pacientes continua a não atingir as metas recomendadas de LDL-c, apesar do amplo arsenal terapêutico disponível.^25 , 27 , 28 , 327 , 330^ No estudo REACT, envolvendo 2.364 pacientes de alto risco cardiovascular no Brasil, 77% estavam em uso de estatinas, mas 20 a 30% apresentavam níveis de LDL-c ≥ 100 mg/dL.^331^

#### 7.13.2. Combinação de Estatina e Ezetimiba

O estudo IMPROVE-IT concluiu que, quando adicionada à sinvastatina 40 mg, a ezetimiba 10 mg resultou em uma redução adicional de 24% dos níveis de LDL-c e, de forma proporcional, em uma redução de 7,2% na taxa de eventos vasculares maiores em comparação com a monoterapia com estatina ao longo de 7 anos. Registros indicam que a monoterapia com estatinas de alta intensidade reduz, em média, o LDL-c em aproximadamente 50%; quando associadas à ezetimiba, essa redução passa a 65%.^332^

Além disso, em uma análise de simulação do estudo DA VINCI com 2.482 pacientes que não atingiram os seus alvos de LDL-c, concluiu-se que a otimização da monoterapia com estatinas seria insuficiente para o atingimento de metas na maioria dos casos. Por outro lado, a adição de ezetimiba dobraria a proporção de indivíduos que atingiriam as suas metas, independentemente da categoria de risco cardiovascular.^333^

Corroborando esses dados, o ensaio clínico RACING demonstrou que a combinação de rosuvastatina 10 mg com ezetimiba foi não inferior à rosuvastatina 20 mg em monoterapia para a redução de desfechos cardiovasculares maiores. Entre as duas estratégias hipolipemiantes de alta intensidade – uma com estatina de moderada intensidade + ezetimiba e outra com estatina de alta intensidade –, a estratégia combinada resultou em maior atingimento de metas de LDL-c ao longo do tempo e menor incidência de suspensão de tratamento.^191^

#### 7.13.3. Combinação de Estatina e Terapia Direcionada para PCSK9

No estudo FOURIER, o evolocumabe, uma terapia dirigida à PCSK9, reduziu o LDL-c em 59% e o risco de eventos cardiovasculares maiores em 15% durante um seguimento de 26 meses em pacientes já em uso de doses otimizadas de estatinas.^168^ No estudo ODYSSEY Outcomes, o alirocumabe reduziu o LDL-c em até 62,7% e eventos cardiovasculares maiores em 15% em pacientes em uso de estatina de alta intensidade.^169^

Mais recentemente, uma estratégia de implementação com inclisirana, administrada imediatamente após falha em atingir LDL-c < 70 mg/dL apesar do uso de estatina em dose máxima tolerada, demonstrou efetividade superior ao tratamento usual: 81,8% dos pacientes atingiram LDL-c < 70 mg/dL e 71,6% atingiram LDL-c < 55 mg/dL, em comparação a 22,2% e 8,9% no grupo de tratamento usual.^334^

#### 7.13.4. Combinação de Ezetimiba e Ácido Bempedoico

Um estudo de fase 3, randomizado e controlado em pacientes de alto risco cardiovascular, sendo que cerca de 65% faziam uso prévio de estatinas, demonstrou que a ezetimiba combinada com ácido bempedoico reduziu os níveis de LDL-c em 38% comparado à redução de 17% e 23%, respectivamente, com adição isolada do ácido bempedoico ou da ezetimiba.^335^

#### 7.13.5. Combinação de Estatina, Ezetimiba e Terapia Direcionada para PCSK9

A utilização conjunta de estatina de alta intensidade, ezetimiba e terapia direcionada à PCSK9 pode levar a uma redução de aproximadamente 85% nos níveis de LDL-c. Essa estratégia pode ser particularmente apropriada em pacientes de risco muito alto, extremo, ou com hipercolesterolemia grave que não atingem as metas com terapia dupla.

#### 7.13.6. Combinação de Estatina, Ezetimiba e Ácido Bempedoico

Em uma análise de simulação envolvendo 105.577 pacientes com risco cardiovascular elevado (n = 28.677) ou muito elevado (n = 76.900), 88% estavam em monoterapia com estatinas. A adição sequencial de ezetimiba e de ácido bempedoico aumentou significativamente a taxa de atingimento das metas de LDL-c, de 11,2% com estatina isolada para 33,1% com estatina + ezetimiba, e para 61,9% com a adição do ácido bempedoico.^336^

**Table:** 

Recomendação	Força da recomendação	Certeza da evidência
Em indivíduos de alto risco cardiovascular, recomenda-se a favor da terapia inicial com estatina de alta intensidade e ezetimiba para atingir a meta terapêutica.	FORTE	ALTA
Em indivíduos de muito alto risco cardiovascular, recomenda-se a favor da terapia inicial com estatina de alta intensidade e ezetimiba, e potencialmente terapia anti-PCSK9, para atingir a meta terapêutica.	FORTE	ALTA
Em indivíduos de extremo risco cardiovascular, recomenda-se a favor do uso de terapia inicial com estatina de alta intensidade, ezetimiba e terapia anti-PCSK9 para atingir a meta terapêutica.	FORTE	ALTA
Em indivíduos que não atingirem a meta terapêutica com estatina de alta intensidade e ezetimiba, recomenda-se a favor de adicionar terapia anti-PCSK9 ou ácido bempedoico, conforme a meta terapêutica.	FORTE	ALTA
Em indivíduos com HF ou LDL-c ≥ 190 mg/dL, recomenda-se a favor do uso de estatina de alta intensidade + ezetimiba como terapia inicial para atingir a meta terapêutica.	FORTE	ALTA
Em indivíduos com intolerância à estatina, recomenda-se a favor da terapia combinada adaptada (por exemplo, ácido bempedoico com ezetimiba ou terapia anti-PCSK9 com ou sem ezetimiba), conforme meta terapêutica.	FORTE	ALTA

HF: hipercolesterolemia familiar; LDL-c: colesterol da lipoproteína de baixa densidade; PCSK9: proproteína convertase subtilisina/quexina tipo 9.

## 8. Manejo da Intolerância à Estatina

Intolerância às estatinas é tipicamente manifesta como queixas musculares, que podem incluir dores, fraqueza, cãibras. A importância desse tema reside na associação entre a não aderência ou interrupção do tratamento com estatina e o risco de desenvolvimento de doença cardiovascular.

### 8.1. Definição

Intolerância às estatinas é a incapacidade de tolerar várias estatinas, em qualquer dose, por aparecimento de sintomas e/ou alterações laboratoriais, e que leva à necessidade da interrupção do tratamento. Os sintomas e/ou alterações laboratoriais devem desaparecer com a suspensão e reaparecer após reintrodução da mesma ou de outra estatina e não podem ser atribuídos a interações medicamentosas ou a condições que sabidamente aumentam a chance do aparecimento da intolerância.

### 8.2. Prevalência

De acordo com metanálise,^282^ a prevalência da intolerância às estatinas é significativamente menor em estudos clínicos randomizados do que em estudos de coorte, variando de 4 até 21%. São também maiores as prevalências em populações em prevenção primária em comparação com as em prevenção secundária, sendo maiores com o aumento da idade, no sexo feminino, em asiáticos e negros e na presença de obesidade, DM, hipotireoidismo, doença hepática e disfunção renal, com o uso concomitante de medicações antiarrítmicas, bloqueadores dos canais de cálcio, e com dose elevada de estatina.^282^ A conclusão dessa análise, que envolveu mais de 4 milhões de pacientes, sugere que a prevalência da intolerância às estatinas é frequentemente superestimada e enfatiza a necessidade da cuidadosa avaliação dos pacientes com potenciais sintomas relacionados à intolerância às estatinas.^282^

### 8.3. Diagnóstico

Vários critérios para o diagnóstico da intolerância às estatinas têm sido propostos; entretanto, todos se baseiam em critérios clínicos e exames laboratoriais.^337^ Até o momento, não existem biomarcadores sensíveis ou específicos da intolerância às estatinas. Assim sendo, para esse diagnóstico, é necessária a exclusão de outras causas possíveis, garantindo clara relação causal entre o uso da estatina e as reações adversas.^337^ Nos casos em que há suspeita da intolerância, quatro elementos fundamentais devem ser identificados para a sua definição:^337^

1. **manifestação clínica** – envolve sintomas subjetivos como mialgia, miastenia ou cãibras e/ou anormalidades de testes laboratoriais (CK, TGO ou TGP);
2. **tipo e dose da estatina empregada** – os pacientes devem ser incapazes de tolerar pelo menos dois tipos de estatina, com uma delas sendo tomada na menor dose diária (por exemplo, atorvastatina 10 mg, rosuvastatina 5 mg, simvastatina 5 mg, pitavastatina 1 mg, pravastatina 10 mg, fluvastatina 20 mg, lovastatina 20 mg);
3. **tempo e causalidade** – as reações adversas devem ocorrer após o início do uso da estatina ou após o aumento de dose, melhorar após interrupção da tomada e reaparecer quando a estatina é reiniciada;
4. **exclusão de outras causas possíveis** – a possibilidade de que as reações adversas estejam relacionadas a outras doenças ou interações medicamentosas deve ser baixa.

### 8.4. Efeito Nocebo

O efeito nocebo consiste na manifestação de sintomas adversos não atribuíveis farmacologicamente ao tratamento, mas sim decorrentes de expectativas negativas do paciente em relação à terapia. No caso específico, esse efeito pode precipitar a descontinuidade do uso de estatinas. A etiologia do efeito nocebo está vinculada à antecipação de malefícios induzida por diversos fatores, tais como a comunicação de potenciais efeitos adversos pelo médico prescritor, o detalhamento presente em termos de consentimento informado para ensaios clínicos, a exposição a informações de fontes não clínicas, como a internet e as mídias sociais, e a observação da sintomatologia em outros pacientes.^338^

### 8.5. Sintomas Musculares

É comum o emprego do termo sintomas musculares relacionados às estatinas (SMRE) em referência aos sintomas musculares associados às estatinas (dores, cãibras ou fraqueza), mas o termo não implica necessariamente a estatina como fator causal. Os sintomas musculares são tipicamente bilaterais e simétricos e exclusivos dos músculos esqueléticos.^339^

#### 8.5.1. Características Clínicas, Classificação e Manejo dos Sintomas Musculares Relacionados às Estatinas

**Figura 8.1:**
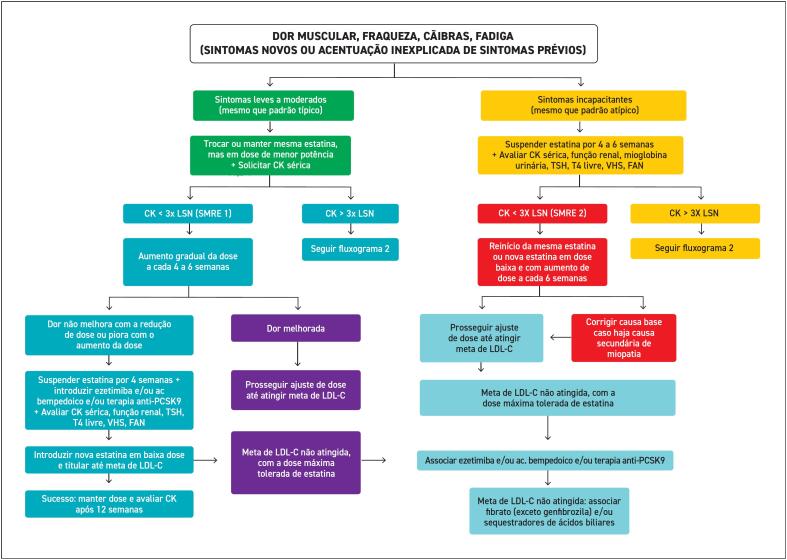
Fluxograma da investigação e diagnóstico provável de sintomas musculares associados a estatinas.

O manejo clínico da SMRE deve ser baseado tanto na presença de sintomas musculares como na elevação da CK, tendo como princípio os sete padrões de SMRE adotados por esta diretriz. Os padrões de SMRE abrangem desde elevações assintomáticas de CK até 3x LSN ( **SMRE 0** ), mialgia tolerável ( **SMRE 1** ) e intolerável ( **SMRE 2** ), miopatia moderada ( **SMRE 3** ) e grave ( **SMRE 4** ) até rabdomiólise ( **SMRE 5** ) e miosite necrotizante autoimune ( **SMRE 6** ). O reconhecimento dos distintos fenótipos e graus de gravidade ajuda a tornar o manejo clínico mais prático, conforme as Figuras 8.1 e 8.2 .

**Figura 8.2:**
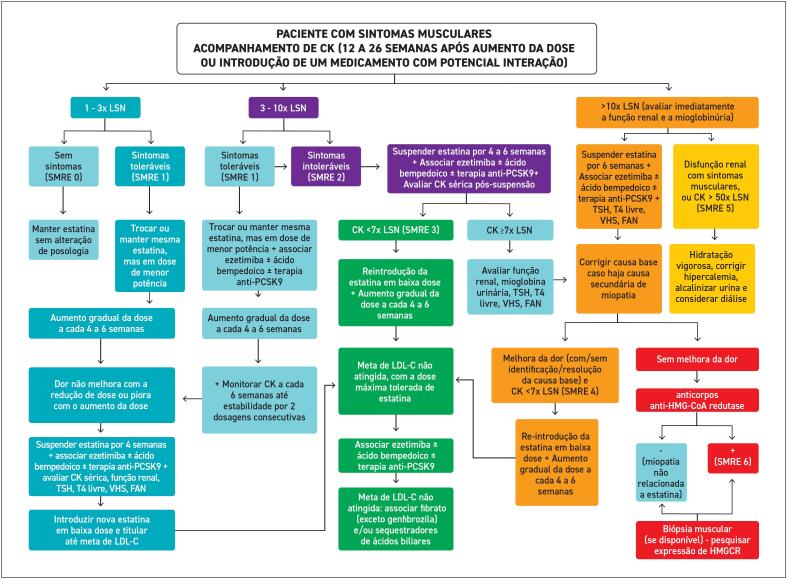
Fluxograma do diagnóstico específico e manejo dos sintomas musculares associados a estatinas.

Ao ponderar sobre um caso potencial de SMRE, faz-se importante: (i) valorizar todas as queixas musculares (dor, fraqueza ou cãibras), não apenas dor muscular, levando em conta o histórico de queixas musculares prévias, comorbidades e uso de outros fármacos; (ii) reconhecer a temporalidade usual entre o início da terapia com estatina e o início dos sintomas musculares, que é habitualmente entre 4 e 12 semanas, mas que também pode raramente ocorrer após mais de 1 ano ou se instalar abruptamente após aumento da dose de estatina ou administração de fármaco ou alimento que induza competição farmacocinética.^340^ Em geral, (iii) o padrão de dor muscular e fraqueza ocorre de forma simétrica e proximal e afeta grandes grupos musculares como nádegas, coxas, panturrilhas e musculatura dorsal. As queixas musculares tendem a ser mais frequentes em pacientes que praticam atividades físicas.

Na presença de dor muscular intolerável, deve-se sempre medir a CK sérica imediatamente. Havendo níveis de CK > 7x LSN ou persistentemente > 3x LSN, deve-se também avaliar os níveis de hormônios tireoidianos (TSH, T4 livre), velocidade de hemossedimentação (VHS) e fator antinuclear (FAN). Na presença de sintomas intoleráveis, deve-se sempre pedir em conjunto a dosagem sérica de ureia e creatinina e mioglobinúria. Havendo causa secundária que possa explicar os SMRE por estatinas, deve-se tentar corrigir a causa e reiniciar a estatina em doses baixas, com aumento progressivo de dose.

#### 8.5.2. Sintomas Musculares Toleráveis e Intoleráveis

Mais frequentemente, as queixas musculares ocorrem sem elevação substancial da CK.^341^ O passo mais importante na presença de tais sintomas é diferenciar a sua tolerabilidade, pois o seu impacto pode variar muito entre indivíduos, especialmente naqueles com comorbidades como hipotireoidismo, doenças do colágeno, fibromialgia e outras.

Nos casos de dor muscular tolerável com ou sem elevação de CK ( **SMRE 0** ) ou de até 3x LSN ( **SMRE 1** ), pode-se considerar a redução temporária de dose ou mudança da estatina, mas sem maiores preocupações adicionais ( Figura 8.1 ). Caso haja elevação da CK entre 3 e 7 vezes o LSN com sintomas toleráveis, será necessária a redução de dose seguida de monitorização mais cautelosa da CK ( Figura 8.2 ). Na presença de sintomas intoleráveis e elevação da CK ( **SMRE 2** ), a suspensão da estatina passa a ser necessária e deve motivar investigação mais ampla ( Figuras 8.1 e 8.2 ). A presença de queixas musculares novas que atinjam a suspeição clínica para SMRE deve, portanto, motivar a solicitação da CK sérica. Em geral, os casos menos graves de SMRE por estatinas ( **SMRE 0 a 4** ) cursam de forma autolimitada e não deixam sequelas permanentes.

#### 8.5.3. Elevação da CK

Durante a terapia com estatinas, elevações transitórias de CK podem ocorrer mesmo em pacientes assintomáticos, mas sem significado clínico. Por essa razão, no curso da terapia com estatinas, a dosagem rotineira de CK não é recomendada exceto quando se introduz novo fármaco ou se eleva a dose de uma estatina. Entre os pacientes que necessitarem nova dosagem, aqueles assintomáticos com pequenas elevações de CK (< 3x LSN) ( **SMRE 0** ) não necessitam suspender a estatina ou mudar sua posologia (Fluxogramas 1 e 2). Naqueles assintomáticos que tiverem elevação de CK de 3 a 7x LSN ( **SMRE 1** ), o período de suspensão não se faz necessário, podendo-se reiniciar a mesma estatina ou uma nova estatina em baixa dose, com ajuste de dose a cada 4 a 6 semanas.

Aprofundando sobre esse mesmo ponto de vista, a presente Diretriz da Sociedade Brasileira de Cardiologia recomenda a suspensão transitória da estatina e monitorização periódica das taxas de CK a cada 4 a 6 semanas, caso haja elevação da CK entre 3 e 7x o LSN na presença de sintomas musculares intoleráveis ( **SMRE 2** ). Naqueles que apresentarem CK entre 3 e 7x LSN na presença de sintomas toleráveis ou ausência de sintomas ( **SMRE 1** ), recomenda-se a troca para regime de baixa intensidade e monitorização mais cuidadosa da CK a cada 4 a 6 semanas.

Entretanto, independentemente da presença de sintomas, se taxas de CK > 7x LSN forem observadas, a suspensão da estatina deve ser realizada por 4 a 6 semanas, seguida de nova dosagem de CK e reavaliação. Caso não haja redução da CK para abaixo de 7x LSN em até 6 semanas sem estatinas ( **SMRE 4 ou SMRE 6** ), o paciente deverá ser avaliado quanto à presença de causa secundária por meio de avaliação clínica e laboratorial mais minuciosa, incluindo investigação da função renal, hormônios tireoidianos (TSH, T4 livre), VHS e FAN. Caso não haja melhora da dor com a suspensão da estatina e não sejam encontradas causas associadas, deve-se considerar o diagnóstico diferencial entre miopatia autoimune ( **SMRE 6** ) e miopatia não relacionada a estatinas.

Naqueles que reduzirem as taxas séricas de CK após suspensão, mas durante a reintrodução e/ou titulação da dose de estatina haja nova elevação da CK acima de 7x LSN (sendo excluídas causas secundárias, como exercício, hipotireoidismo ou doença muscular metabólica), deve-se usar a mesma estatina em dose menor ou estatina alternativa. Em seguida, adiciona-se terapia não estatínica com a finalidade de atingir a meta de LDL-c.

#### 8.5.4. Rabdomiólise

A rabdomiólise ( **SMRE 5** ) é o evento muscular adverso mais grave durante a terapia com estatinas, podendo gerar necrose muscular, distúrbios hidroeletrolíticos graves, lesão renal aguda, coagulopatia, choque e morte. O critério mais comumente verificado é a dor muscular acompanhada de aumento abrupto da CK em mais de 10x O LSN, embora mais raramente a rabdomiólise possa se manifestar com perda de força muscular ou sintomas musculares discretos associados a aumento de mais de 40 a 50x LSN na CK. Portanto, para efeito de critério diagnóstico, considera-se rabdomiólise quando há aumento assintomático de CK > 50x LSN ou quando há dor muscular associada a CK > 10x LSN e disfunção renal (elevação da creatinina sérica ≥0,5 mg/dL) e mioglobinúria.

#### 8.5.5. Miopatia Necrotizante Autoimune por Estatinas

Caracteristicamente, a miopatia necrotizante autoimune ( **SMRE 6** ) está relacionada ao aparecimento de autoanticorpos séricos dirigidos contra a 3-hidroxi-3-metilglutaril-coenzima A redutase (HMGCR), o alvo farmacológico das estatinas. Na maioria dos casos de miopatia associada à estatina, os sintomas desaparecem após a descontinuação do tratamento. Entretanto, em casos excepcionalmente raros, a miopatia autoimune pode aparecer. No exame físico, os pacientes que apresentam esse problema tipicamente se queixam de fraqueza muscular, como por exemplo dificuldade de levantar da cadeira, subir escadas e levantar objetos pesados. Na avaliação laboratorial, a taxa sérica de CK é muito elevada (acima de 2.000 UI/L) e persistente, mesmo após o abandono da estatina.

O diagnóstico é feito na presença de autoanticorpos contra a HMG-CoA redutase no contexto da exposição a uma estatina, sintomas musculares e elevação persistente da CK.^342 , 343^ Na biópsia muscular, os achados histológicos principais são a necrose de células musculares e a infiltração inflamatória.^343^ O tratamento envolve corticosteroide e imunossupressão.^343^ Ao contrário das outras formas de SMRE, os sintomas na **SMRE 6** não melhoram após a suspensão da estatina. Porém, por se tratar do mecanismo causal, a exposição ao fármaco deve ser logo evitada.

### 8.6. Causas Associadas à Intolerância às Estatinas

Muitos fatores podem influenciar o desenvolvimento de efeitos adversos associados às estatinas, variando desde farmacológicos até atributos inerentes aos pacientes^343^ – alguns deles são listados na Tabela 8.1 . Na Tabela 8.2 , há informações especiais sobre as interações medicamentosas que podem potencialmente se associar ou induzir sintomas musculares em usuários de estatinas.

**Tabela 8.1:** Causas associadas à intolerância às estatinas

Fator de risco	Descrição e justificativa fisiopatológica	Implicações clínicas e manejo
**A. Idade avançada**	Com o envelhecimento, ocorre um declínio fisiológico da função de órgãos vitais. A redução da taxa de filtração glomerular renal e a menor eficiência do metabolismo hepático (enzimas do citocromo P450) alteram a farmacocinética da estatina, levando a um aumento da sua concentração plasmática e do tempo de exposição ao fármaco.	Iniciar com doses mais baixas (start low, go slow). Monitorar a função renal (creatinina e TFG) e hepática. Avaliar cuidadosamente a polifarmácia, comum nessa população.
**B. Sexo feminino**	Mulheres, em geral, possuem menor massa corporal e volume de distribuição do que os homens, o que pode resultar em concentrações plasmáticas mais elevadas da estatina com a mesma dose. Fatores hormonais também podem influenciar o metabolismo e a sensibilidade muscular.	Considerar o ajuste de dose com base no peso e na massa corporal. Estar atento a queixas musculares, mesmo que sutis.
**C. Etnia asiática**	Populações do leste asiático (por exemplo, chineses e japoneses) apresentam maior prevalência de polimorfismos genéticos em transportadores hepáticos (como o OATP1B1, codificado pelo gene SLCO1B1) e em enzimas metabolizadoras. Isso diminui a captação hepática e/ou o clearance da estatina, resultando em maior exposição sistêmica.	Usar doses iniciais mais conservadoras, especialmente com rosuvastatina e atorvastatina. A dose inicial de rosuvastatina recomendada é de 5 mg. A titulação deve ser cautelosa.
**D. Hipotireoidismo**	O hipotireoidismo não controlado é, por si só, uma causa de miopatia, com sintomas como dor muscular, cãibras e elevação da creatina quinase (CK). A condição também pode reduzir o metabolismo das estatinas, potencializando a toxicidade. Os sintomas se sobrepõem e se confundem.	Sempre dosar o TSH antes de iniciar a estatina e em pacientes que desenvolvem sintomas musculares durante o tratamento. A correção da disfunção tireoidiana pode resolver os sintomas.
**E. Consumo de álcool**	O consumo excessivo e crônico de álcool pode causar miopatia alcoólica e também sobrecarrega o metabolismo hepático. Essa combinação aumenta a vulnerabilidade do músculo aos efeitos da estatina e pode prejudicar a metabolização do fármaco.	Investigar o padrão de consumo de álcool na anamnese. Orientar a moderação ou cessação. Monitorar enzimas hepáticas (TGO/TGP) e CK em pacientes com consumo de risco.
**F. Doenças neuromusculares**	Pacientes com doenças neuromusculares preexistentes (por exemplo, esclerose lateral amiotrófica e distrofias) possuem uma reserva funcional muscular diminuída e podem ter seus sintomas exacerbados ou desmascarados pelo uso de estatinas.	O uso de estatinas deve ser extremamente criterioso. A decisão deve ser compartilhada com um neurologista, pesando rigorosamente os riscos e benefícios cardiovasculares.
**G. Doença renal crônica**	A insuficiência renal (especialmente TFG < 30-60 mL/min) reduz significativamente o clearance de estatinas hidrofílicas (pravastatina, rosuvastatina) e seus metabólitos, aumentando o risco de acúmulo e toxicidade.	Ajustar a dose da estatina conforme a taxa de filtração glomerular. Atorvastatina e fluvastatina são consideradas mais seguras, por terem menor eliminação renal.
**H. Doença hepática**	O fígado é o principal local de metabolismo da maioria das estatinas. Doenças hepáticas ativas ou insuficiência hepática comprometem esse processo, elevando os níveis séricos do fármaco.	Estatinas são contraindicadas em doença hepática ativa ou descompensada. Em hepatopatias crônicas estáveis, usar com cautela, em doses baixas e com monitoramento de transaminases.
**I. Exercício físico excessivo**	A prática de exercícios extenuantes ou muito acima do nível de condicionamento do indivíduo causa microlesões musculares e elevação da CK. Esse efeito pode ser confundido com SMRE ou, em casos raros, ser potencializado pela estatina, aumentando o risco de rabdomiólise.	Orientar o paciente a iniciar programas de exercício de forma gradual. Em caso de sintomas após exercício intenso, considerar uma pausa na estatina e reavaliar a CK após a recuperação.
**J. História pessoal/familiar**	Uma história prévia de intolerância a uma estatina aumenta a chance de intolerância a outra. A história familiar sugere uma predisposição genética para SMRE, como polimorfismos em genes metabolizadores.	Considerar o uso de doses menores e estatinas com menor risco de miopatia (por exemplo, pravastatina e fluvastatina) ou terapias não estatínicas. A reintrodução deve ser feita com cautela.
**K. Baixa massa corpórea**	Similar ao fator "sexo feminino", indivíduos com baixo peso ou fragilidade (sarcopenia) têm menor volume de distribuição para o fármaco, o que leva a concentrações plasmáticas mais altas e maior risco de toxicidade.	A dosagem deve ser individualizada e conservadora. Evitar doses altas de estatinas de alta potência.
**L. Polimorfismo SLCO1B1**	O gene SLCO1B1 codifica o transportador de ânions orgânicos OATP1B1, responsável pela captação das estatinas pelo fígado. Polimorfismos de função reduzida (variantes genéticas) diminuem essa captação, aumentando a concentração da estatina no sangue e no músculo.	Esse é o principal preditor genético de miopatia por sinvastatina, mas também afeta outras estatinas. Na prática, a testagem genética não é rotina, mas a suspeita aumenta com história familiar positiva ou intolerância a múltiplas estatinas.
**M. Dose alta de estatina**	O risco de SMRE é diretamente proporcional à dose e à potência da estatina. Doses altas (por exemplo, sinvastatina 80 mg – proscrita; atorvastatina 80 mg; rosuvastatina 40 mg) aumentam significativamente a exposição sistêmica ao fármaco.	Utilizar a menor dose eficaz para atingir a meta de LDL-c. Considerar a associação com um fármaco não estatínico (por exemplo, ezetimiba) para evitar a necessidade de altas doses de estatina.
**N. Efeito nocebo**	A ampla divulgação de informações negativas sobre estatinas na mídia e internet pode criar uma expectativa de efeitos adversos no paciente. Esse efeito nocebo pode fazer com que o paciente atribua qualquer dor muscular à medicação ou até mesmo induzir sintomas reais.	Validar a queixa do paciente. Fornecer informações claras e baseadas em evidências sobre os reais riscos e os imensos benefícios da terapia. Em estudos, muitos pacientes que relatam SMRE em braços abertos não apresentam sintomas em ensaios duplo-cegos.
**O. Interações medicamentosas**	Muitas drogas inibem as enzimas do citocromo P450 (principalmente a CYP3A4), responsáveis pelo metabolismo da atorvastatina, lovastatina e sinvastatina. O uso concomitante eleva drasticamente os níveis da estatina. Exemplos são os antifúngicos azólicos, antibióticos macrolídeos, fibratos (especialmente genfibrozila), inibidores de protease e amiodarona.	Realizar uma revisão completa da prescrição do paciente antes de iniciar ou ajustar a estatina. Optar por estatinas com vias metabólicas diferentes (pravastatina, rosuvastatina e fluvastatina) se a interação for inevitável. Consultar tabelas de interação medicamentosa.

**Tabela 8.2:** Tabela de interações medicamentosas de estatinas

Classe do medicamento interagente	Mecanismo de interação e estatinas afetadas	Risco clínico e recomendações de manejo
**1. Anticoagulantes**	Varfarina: inibição do CYP2C9 (fluvastatina e rosuvastatina) ou deslocamento de ligação proteica (lovastatina).^354^ DOACs: ausência de interações clinicamente significativas.^355^	Varfarina: aumenta o risco de sangramento. É mandatório monitorar o INR de forma intensificada ao iniciar ou ajustar a dose da maioria das estatinas (exceção: pravastatina). DOACs (dabigatrana, apixabana, rivaroxabana e edoxabana): podem ser coadministrados com segurança.
**2. Antifúngicos azólicos**	Inibição potente do CYP3A4, bloqueando o metabolismo de sinvastatina, lovastatina e atorvastatina.^356^	Risco elevado de miopatia/rabdomiólise. Contraindicado: itraconazol + sinvastatina/lovastatina (aumento de até 20x na exposição).^357^ Alternativas seguras: pravastatina (sem metabolismo via CYP3A4),^358^ fluvastatina ou rosuvastatina (usar com monitoramento).
**3. Agentes antirretrovirais (ARV)**	Modulação do CYP3A4:^359^ inibidores de protease (IP): inibem o metabolismo das estatinas. NNRTI (efavirenz): induz o metabolismo das estatinas.	Estatinas de escolha (mais seguras): pravastatina e pitavastatina. Com inibidores de protease (IP): – evitar: sinvastatina, lovastatina; – desaconselhada: atorvastatina com IPs potentes (por exemplo, ritonavir); – cautela: rosuvastatina (exposição muito elevada com lopinavir/ritonavir). Com efavirenz (NNRTI): reduz a concentração de atorvastatina, sinvastatina e pravastatina. Monitorar eficácia e ajustar dose se necessário.^360^
**4. Bloqueadores dos canais de cálcio (BCC)**	Inibição do metabolismo (CYP3A4) de sinvastatina e lovastatina por verapamil e diltiazem. A amlodipina também interage.^361 , 362^	Risco aumentado de miopatia. Sinvastatina: dose máxima de 20 mg/dia com verapamil ou amlodipina. Lovastatina: dose máxima de 40 mg/dia com verapamil.
**5. Agentes antiarrítmicos (amiodarona)**	Inibidor do CYP3A4 e P-gp, afetando principalmente sinvastatina e lovastatina.^363^	Risco aumentado de miopatia. Sinvastatina: dose máxima de 20 mg/dia. Lovastatina: dose máxima de 40 mg/dia. Sem ajuste necessário: atorvastatina, rosuvastatina, pravastatina, fluvastatina e pitavastatina.
**6. Imunossupressores**	Inibição do CYP3A4 e do transportador OATPB1 por ciclosporina, tacrolimus, everolimus e sirolimus.^364 , 365^	Risco significativamente elevado de miopatia/rabdomiólise. Evitar: lovastatina, sinvastatina, pitavastatina. Usar com dose limitada (se indispensável): – fluvastatina ≤40 mg/dia – pravastatina ≤20 mg/dia – rosuvastatina ≤5 mg/dia – atorvastatina > 10 mg/dia exige monitoramento rigoroso (CK, sintomas).
**7. Macrolídeos**	Inibição potente do CYP3A4 por claritromicina e eritromicina, afetando sinvastatina, lovastatina e atorvastatina.^366^	Risco aumentado de miotoxicidade. Evitar: sinvastatina, lovastatina, atorvastatina em associação com eritromicina e claritromicina. Alternativa segura: azitromicina não possui interação significativa e pode ser usada com qualquer estatina. Estatinas não afetadas: rosuvastatina, fluvastatina e pravastatina.
**8. Fibratos**	Genfibrozil: inibe a glucuronidação das estatinas, aumentando a concentração sérica.^303^ Fenofibrato: perfil de interação mais seguro.	Risco aumentado de miopatia. Genfibrozil: – evitar associação com lovastatina, pravastatina e sinvastatina; – considerar com cautela atorvastatina, pitavastatina, rosuvastatina; – seguro: fluvastatina. Fenofibrato: associação com qualquer estatina é considerada razoável quando clinicamente indicada.

### 8.7. Manejo do Paciente com Intolerância às Estatinas

É aconselhável monitorar os pacientes quanto à ocorrência de SMRE, particularmente durante os primeiros meses do início da terapia com estatina e em subsequentes aumentos de doses. A determinação da CK deve ser realizada se ocorrerem sintomas. Pacientes com fatores de risco para a ocorrência de SMRE merecem monitoração mais de perto por apresentarem maior risco de desenvolvimento de rabdomiólise.

O tratamento com estatina deve ser suspenso imediatamente se a taxa de CK for maior que 10 vezes o limite superior da normalidade ou quando a miopatia for suspeitada ou diagnosticada. Se houver aumento moderado nas taxas de CK (entre 3 e 10 vezes o limite superior da normalidade), é recomendada a monitoração das taxas de CK semanalmente. A dosagem da CK deve ser solicitada antes do início da terapia com estatina, o que possibilitará referência do valor basal, particularmente em indivíduos com alto risco de eventos adversos musculares, como aqueles com antecedentes de intolerância à estatina, antecedentes familiares de miopatia ou uso concomitante de fármacos que aumentem o risco de miopatia. Entretanto, não é recomendada dosagem regular da CK na ausência de sintomas, alterações da terapia ou presença de comorbidades.

A avaliação basal das enzimas hepáticas (ALT e AST) deve ser realizada antes do início da terapia com estatina. Durante o tratamento, deve-se avaliar a função hepática quando ocorrerem sintomas ou sinais sugerindo hepatotoxicidade (fadiga ou fraqueza, perda de apetite, dor abdominal, urina escura ou aparecimento de icterícia). Se houver anormalidade das taxas dessas enzimas, devem ser excluídos quaisquer fatores de risco e avaliada a correlação com o uso da estatina. Se as taxas séricas de ALT/AST forem maiores que 3 vezes o limite superior da normalidade, com aumento concomitante da bilirrubina total, deve-se considerar redução da dose ou suspensão da estatina. Se o aumento for menor que 3 vezes o limite superior da normalidade, sugere-se observação, com a dose original ou reduzida, ou mudança para estatina com outra via metabólica (por exemplo, pravastatina).

Pequena porcentagem de pacientes apresenta aumento de enzimas hepáticas, particularmente ALT e AST, com pouco ou nenhum efeito sobre a gama glutamil transferase, a fosfatase alcalina ou as bilirrubinas. Aumentos das transaminases pelas estatinas geralmente ocorrem nos primeiros 6 meses de tratamento, são assintomáticos e revertem com a redução da dose ou interrupção do tratamento. Aumentos isolados das transaminases, na ausência de aumento das taxas de bilirrubinas, não têm sido ligados, clínica ou histologicamente, a evidências de lesão hepática aguda ou crônica.^344^

#### 8.7.1. Interrupção e Reinício do Uso da Estatina

A interrupção da terapia com estatina em pacientes sob risco cardiovascular está associada a um aumento de eventos adversos cardiovasculares maiores e mortalidade.^345^ Em função disso, todas as tentativas de convencer os pacientes a continuarem o tratamento devem ser feitas. O primeiro passo na tentativa de manter o tratamento é parar a estatina (se ainda estiver em uso) e permitir um período de *washout* de 2 semanas, observando se os sintomas desaparecem.

Em paralelo à retirada da estatina, é recomendável prescrever terapia hipolipemiante não estatínica, como ezetimiba e/ou ácido bempedoico e/ou terapia anti-PCSK9, a fim de reduzir os riscos atribuíveis à elevação do LDL-c, caso a retirada da estatina seja feita sem complementação terapêutica.^346 , 347^ Recomenda-se esclarecer aos pacientes que a ezetimiba, ácido bempedoico e terapia anti-PCSK9 não pertencem à classe das estatinas, a fim de mitigar equívocos que possam comprometer a adesão ao tratamento.^348^

Depois desse período, pode-se tentar a mesma estatina em menor dose ou outra estatina.^345^ A mudança para uma estatina que é metabolizada por outra via pode funcionar para alguns pacientes – por exemplo, atorvastatina, que é lipofílica e principalmente eliminada pelo fígado, pode ser trocada para uma hidrofílica, como rosuvastatina ou pravastatina, eliminadas em parte pelos rins, ou pela pitavastatina, que é lipofílica, mas eliminada pelo fígado por meio de outras vias.

Embora essa prática não seja fundamentada por estudos clínicos, para pacientes muito relutantes em reiniciar a estatina ou que já apresentaram sintomas com mais de duas estatinas com tomadas diárias, pode-se tentar a dosagem intermitente com uma estatina com meia-vida estendida (rosuvastatina, atorvastatina ou pitavastatina).

Após o reinício da estatina, para obtenção das metas preconizadas, outros medicamentos que atuam sobre o perfil lipídico podem ser associados a doses menores da estatina ou, eventualmente, utilizados isoladamente, no caso de absoluta intolerância a qualquer dose de estatina ( Figuras 8.1 , 8.2 e 8.3 ).

**Figura 8.3:**
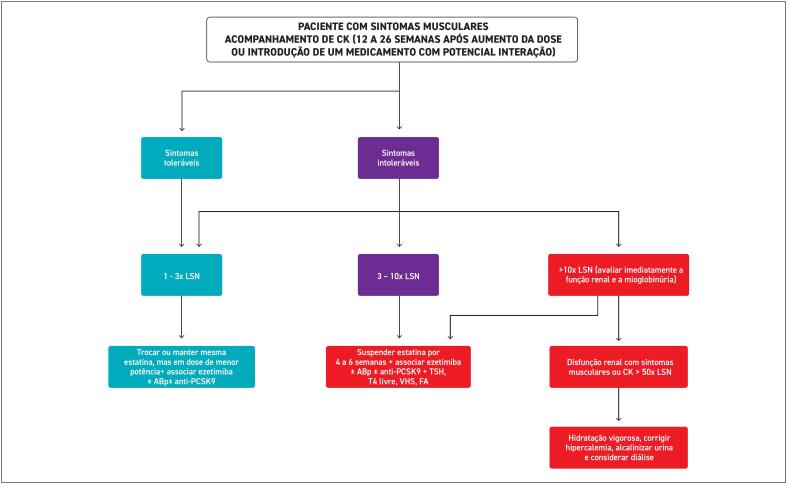
Fluxograma simplificado.

#### 8.7.2. Utilização de Produtos que não têm Comprovação de Benefícios

A deficiência de vitamina D tem sido associada ao aparecimento de sintomas musculares associados às estatinas; entretanto, a reposição não traz melhora do quadro muscular.^349^

A coenzima Q10 (CoQ10) é um composto essencial para a produção de energia nas mitocôndrias das células e atua como antioxidante. O uso de estatinas pode levar à redução dos níveis de CoQ10 no organismo, o que se associaria a fadiga e dores musculares. Isso ocorre porque as estatinas inibem a enzima HMG-CoA redutase, responsável pela síntese do colesterol, mas que também participa da produção da CoQ10.^350 , 351^ Embora o racional para seu uso indique possível benefício em reduzir SMRE, metanálises e estudos maiores^350 - 353^ não demonstraram esse benefício e, portanto, não há evidência suficiente para indicação de suplementação de coenzima Q10 em pacientes tratados com estatinas.

#### 8.7.3. Interações medicamentosas das estatinas

##### 8.7.3.1. Anticoagulantes

A interação medicamentosa entre estatinas e varfarina é relatada, sendo mediada principalmente pela inibição da via metabólica CYP2C9, com destaque para a fluvastatina e rosuvastatina, ou pelo deslocamento da ligação proteica, como ocorre com a lovastatina. A maioria das estatinas, exceto a pravastatina, apresenta potencial para essa interação.^354^ Por outro lado, estudos não evidenciaram interações clinicamente significativas entre a varfarina e a atorvastatina ou pitavastatina. Da mesma forma, não foram demonstradas interações relevantes com os anticoagulantes orais diretos (dabigatrana, apixabana, rivaroxabana e edoxabana).^355^ Para pacientes em uso de varfarina, é mandatório monitorar a razão normalizada internacional (INR) de forma mais frequente ao se iniciar uma estatina ou ao realizar um ajuste de sua dose.

##### 8.7.3.2. Antifúngicos Azólicos

Os antifúngicos azólicos atuam como inibidores da isoenzima CYP3A4, o que resulta em bloqueio metabólico das estatinas metabolizadas por esta via. Essa interação eleva as concentrações plasmáticas da estatina, aumentando o risco de miopatia e rabdomiólise.^356^ A coadministração de itraconazol com sinvastatina ou lovastatina é contraindicada devido ao aumento expressivo na exposição a essas estatinas (aumento de até 20 vezes na AUC), elevando criticamente o risco de toxicidade musculoesquelética.^357^

Em contraste, a pravastatina, por não ser metabolizada pela via CYP3A4, não apresenta interação significativa com o itraconazol.^358^ A rosuvastatina, quando combinada com fluconazol, apresenta um aumento na AUC e Cmax sem relevância clínica demonstrada. Portanto, em pacientes sob tratamento com antifúngicos azólicos, a pravastatina e, com menor grau de evidência, a fluvastatina ou rosuvastatina (com monitoramento) representam alternativas terapêuticas mais seguras.

##### 8.7.3.3. Agentes Antirretrovirais

O manejo da dislipidemia em pacientes com HIV é cada vez mais prevalente devido ao aumento da sobrevida, que acarreta maior risco cardiovascular. A terapia antirretroviral (ARV) apresenta interações medicamentosas clinicamente significativas com as estatinas, primariamente pela modulação do CYP3A4 por inibidores de protease (IP) e inibidores não nucleosídeos da transcriptase reversa ( *non-nucleoside reverse transcriptase inhibitors* , NNRTI).^359^

A pravastatina, por seu metabolismo via sulfatação, e a pitavastatina são as estatinas de escolha e mais seguras nesse cenário. Contudo, a pravastatina pode necessitar de ajuste de dose (aumento) na presença de IPs como nelfinavir ou ritonavir. Em contrapartida, o uso de sinvastatina e lovastatina deve ser evitado em combinação com IPs. A atorvastatina também é desaconselhada com IPs potentes (por exemplo, ritonavir e atazanavir). A rosuvastatina exige cautela, pois sua combinação com lopinavir/ritonavir ou atazanavir/ritonavir demonstrou elevar substancialmente sua exposição sistêmica (AUC e Cmax). De forma distinta, o efavirenz (um NNRTI) atua como indutor enzimático, diminuindo a concentração de atorvastatina, sinvastatina e pravastatina, o que exige monitoramento clínico e potencial ajuste de dose para garantir a eficácia hipolipemiante.^360^

##### 8.7.3.4. Bloqueadores dos Canais de Cálcio

O diltiazem pode causar aumento da Cmax da sinvastatina em 3,6 vezes e da AUC em 5 vezes, e da AUC da lovastatina em 3,5 vezes, com risco aumentado de miopatia.^361^ Recomenda-se ajuste de dose em pacientes tratados com verapamil e sinvastatina (máximo 20 mg) ou lovastatina (máximo 40 mg).

O antagonista do cálcio di-hidropiridínico (amlodipina) produz aumento na Cmax e na AUC da sinvastatina.^362^ O FDA recomenda que as doses de sinvastatina não devem exceder 20 mg por dia quando prescrita em combinação com qualquer dose de amlodipina, devido ao fato de doses mais elevadas estarem associadas a risco aumentado de miopatia e/ou rabdomiólise.

##### 8.7.3.5. Agentes Antiarrítmicos

A amiodarona é um inibidor do CYP3A4 (irreversível), podendo causar interação quando usada concomitantemente com estatinas metabolizadas pelo CYP450 ou substratos da P-gp. Houve relatos de toxicidade entre a amiodarona e estatinas que são substratos do CYP3A4, particularmente a sinvastatina. Assim, um aumento de 75% na AUC e Cmax da sinvastatina foi demonstrado quando coadministrada com amiodarona.^363^

Nenhum ajuste de dose é necessário para atorvastatina, rosuvastatina, pravastatina, fluvastatina e pitavastatina quando coadministrados concomitantemente com amiodarona. A lovastatina não deve exceder 40 mg por dia quando prescrita em combinação com amiodarona, enquanto a sinvastatina deve ser limitada a, no máximo, 20 mg por dia.

##### 8.7.3.6. Imunossupressores

A coadministração de estatinas com inibidores da calcineurina (ciclosporina, tacrolimus) e inibidores de mTOR (everolimus, sirolimus) eleva significativamente o risco de miopatia e rabdomiólise. A interação ocorre por inibição do CYP3A4 e do transportador OATPB1, aumentando a exposição sérica às estatinas. Clinicamente, recomenda-se evitar a associação com lovastatina, sinvastatina e pitavastatina. Quando a terapia é indispensável, pode-se optar por fluvastatina (limitada a 40 mg/dia), pravastatina (≤ 20 mg/dia) ou rosuvastatina (≤ 5 mg/dia). Doses de atorvastatina superiores a 10 mg/dia exigem monitoramento rigoroso da CK e de sintomas de toxicidade muscular.^364 , 365^

##### 8.7.3.7. Macrolídeos

Macrolídeos, especialmente claritromicina e eritromicina, são os inibidores mais potentes da isoenzima CYP3A4, seguidos pelo inibidor fraco roxitromicina e, finalmente, pela azitromicina. CYP3A4 é uma isoenzima que metaboliza sinvastatina, lovastatina e atorvastatina, o que aumenta suas concentrações plasmáticas e o risco de miotoxicidade. Rosuvastatina, fluvastatina e pravastatina não são significativamente afetadas por essa interação. De todos os macrolídeos, a azitromicina pode ser usada com todas as estatinas. A eritromicina aumenta os níveis séricos de sinvastatina e atorvastatina.^366^ Assim, sinvastatina, lovastatina e atorvastatina devem ser evitadas em associação à eritromicina e claritromicina, pelo potencial risco aumentado de miopatia.

##### 8.7.3.8. Interações entre Agentes Hipolipemiantes

A associação de estatinas e fibratos, embora eficaz, eleva o risco de miopatia. A interação é particularmente crítica com a genfibrozila, que inibe a glucuronidação das estatinas, aumentando sua concentração sérica. A combinação de genfibrozila com lovastatina, pravastatina e sinvastatina deve ser evitada.^304^ Sua associação com atorvastatina, pitavastatina e rosuvastatina pode ser considerada se clinicamente indicada, apesar de resultar em um pequeno aumento na concentração da estatina. A fluvastatina não apresenta interação farmacocinética com a genfibrozila, podendo ser usada em combinação sem restrições de dose. Em contraste, o fenofibrato demonstra um perfil de segurança superior, sendo sua associação com qualquer estatina considerada razoável quando houver indicação clínica

**Table:** 

Recomendação	Força da recomendação	Certeza da evidência
Em pacientes nos quais se pretende iniciar estatina, recomenda-se a favor da dosagem basal de CK e de enzimas hepáticas (ALT e AST), especialmente em indivíduos de alto risco para eventos musculares ou hepatotoxicidade.	CONDICIONAL	BAIXA
Em pacientes recebendo estatinas, recomenda-se contra dosagens rotineiras de CK e de enzimas hepáticas na ausência de sintomas musculares, de sinais de hepatotoxicidade ou de alterações na terapia.	FORTE	MODERADA
Em pacientes recebendo estatinas, recomenda-se a favor da dosagem de CK na presença de sintomas musculares graves, e da dosagem de enzimas hepáticas na presença de sinais de hepatotoxicidade.	FORTE	MODERADA
Em pacientes que não toleram a dose sugerida de estatina, recomenda-se a favor de estratégias alternativas para atingir a meta de redução do LDL-c, incluindo a redução da frequência de administração, a troca para outra estatina ou a combinação de outros agentes hipolipemiantes.	FORTE	ALTA
Em pacientes nos quais se opta pela suspensão da estatina, recomenda-se a favor do início imediato da terapia hipolipemiante não estatínica (por exemplo, ezetimiba, ácido bempedoico ou terapia anti-PCSK9) durante o período de suspensão, com o objetivo de mitigar o risco cardiovascular decorrente da elevação do LDL-c.	FORTE	ALTA
Em pacientes recebendo estatinas, recomenda-se contra a reposição de vitamina D com o objetivo de mitigar sintomas musculares associados à estatina.	FORTE	ALTA
Em pacientes recebendo estatinas, recomenda-se contra a suplementação rotineira de coenzima Q10 com o objetivo de mitigar sintomas musculares associados à estatina.	FORTE	MODERADA

CK: Creatina Quinase; ALT: Alanina aminotransferase; AST: Aspartato aminotransferase; LDL-c: colesterol da lipoproteína de baixa densidade; PCSK9: proproteína convertase subtilisina/quexina tipo 9.

## 9. Dislipidemia em Populações Específicas: Considerações para o Manejo Clínico

### 9.1. Insuficiência Cardíaca

A dislipidemia é uma comorbidade comum em pacientes com insuficiência cardíaca (IC), especialmente na presença de doença aterosclerótica. O controle adequado dos lipídios é essencial, pois pode influenciar significativamente a evolução clínica desses pacientes. As estatinas são recomendadas para indivíduos com IC de etiologia isquêmica, devido à sua eficácia na redução de eventos cardiovasculares. No entanto, em pacientes com IC com fração de ejeção reduzida (ICFER) sem doença aterosclerótica estabelecida, os benefícios das estatinas na redução da mortalidade ainda são incertos.

Importante destacar que a interrupção do uso de estatinas em pacientes com IC – mesmo naqueles com doença avançada ou descompensada – não é recomendada. Não há evidências robustas que justifiquem essa conduta, e estudos observacionais sugerem aumento da mortalidade e das hospitalizações após a suspensão do tratamento, especialmente em idosos.^367 - 370^

A prevalência de IC, dislipidemia e doença coronariana é particularmente elevada na população idosa. Nesses pacientes, a polifarmácia é uma preocupação frequente e pode levar à descontinuação inadvertida de estatinas e outros hipolipemiantes. No entanto, essa interrupção pode aumentar o risco de eventos cardiovasculares e mortalidade, conforme demonstrado em estudos observacionais.^369^

Para pacientes que não atingem as metas de LDL-c com estatinas, especialmente aqueles com risco cardiovascular elevado, o uso de terapias adicionais como ezetimiba e terapia anti-PCSK9 deve ser considerado.

Em conclusão, o manejo da dislipidemia na IC deve ser individualizado, com ênfase na continuidade da terapia hipolipemiante, sobretudo em pacientes com doença aterosclerótica. A suspensão das estatinas não é recomendada, dado o risco aumentado de eventos adversos. Em idosos, é fundamental avaliar cuidadosamente a polifarmácia para evitar a interrupção inadequada do tratamento. Estudos futuros são necessários para esclarecer o papel de novas terapias hipolipemiantes nesse cenário clínico.^371^

**Table:** 

Recomendação	Força da recomendação	Certeza da evidência
Pacientes com IC e doença aterosclerótica estabelecida devem manter o uso de estatinas para reduzir o risco de eventos cardiovasculares ateroscleróticos.	FORTE	MODERADA
Para população com IC em uso prévio de estatina, recomenda-se contra a suspensão da mesma caso o paciente apresente sobrevida clinicamente aceitável.	FORTE	MODERADA
Pacientes com ICFER sem doença aterosclerótica podem utilizar estatinas, desde não haja contraindicação e considerando individualização do tratamento.	CONDICIONAL	MODERADA
Terapia anti-PCSK9 devem ser mantidos para pacientes com IC de alto risco cardiovascular quando as metas de LDL-c não forem alcançadas com estatinas e ezetimiba.	FORTE	MODERADA

IC: insuficiência cardíaca; ICFER: insuficiência cardíaca com fração de ejeção reduzida; LDL-c: colesterol da lipoproteína de baixa densidade; PCSK9: proproteína convertase subtilisina/quexina tipo 9.

### 9.2. Pessoas vivendo com o HIV

Pessoas vivendo com HIV (PVHIV) apresentam quase o dobro do risco de DCV em comparação com a população geral, devido a fatores como inflamação crônica, efeitos adversos da terapia antirretroviral (TARV) e alta prevalência de fatores de risco tradicionais.^124^ Apesar da disponibilidade de terapias eficazes para redução do LDL-c, atingir as metas lipídicas nessa população continua sendo um desafio significativo. As estatinas continuam sendo a base do tratamento para redução do LDL-c em PVHIV; no entanto, seu uso é frequentemente dificultado por interações medicamentosas com a TARV, levando a efeitos adversos aumentados e baixa adesão^372^ ( Tabela 9.1 ). O estudo REPRIEVE demonstrou uma redução de 35% nos eventos cardiovasculares com o uso de pitavastatina, destacando seu papel na prevenção primária.^372^ No entanto, desafios como a intolerância às estatinas e a subutilização da terapia permanecem, com estudos mostrando que apenas 37% dos PVHIV de alto risco recebem terapia hipolipemiante adequada.^124^

**Tabela 9.1:** Perfil de segurança e interações de estatinas em pacientes com HIV

Estatina	Dose recomendada	Interação com TARV	Comentários principais
**Pitavastatina**	4 mg/dia	Baixa	Estatina mais segura com TARV; destaque no estudo REPRIEVE, com redução de 35% em eventos CV.^372^
**Rosuvastatina**	10-20 mg/dia	Moderada	Potente; usar com cautela com inibidores de protease.
**Atorvastatina**	10-20 mg/dia	Moderada	Ajustar dose com TARV; evitar doses altas com ritonavir.
**Pravastatina**	20-40 mg/dia	Baixa	Menor potência, mas segura com maioria dos antirretrovirais.
**Sinvastatina**	Evitar	Alta	Contraindicada com inibidores de protease (por exemplo, ritonavir e cobicistate).

O ezetimiba também tem sido subutilizado, com apenas 3,3% dos pacientes recebendo esta terapia, apesar de seu perfil de segurança favorável.^373^ Além disso, calculadoras de risco cardiovascular muitas vezes subestimam o risco em PVHIV, exigindo abordagens individualizadas que incorporem fatores específicos do HIV, como ativação imunológica e exposição à TARV.^374^ O evolocumabe, um inibidor de PCSK9, surgiu como uma alternativa promissora para PVHIV com intolerância às estatinas ou risco residual. O estudo BEIJERINCK demonstrou uma redução de 56,9% nos níveis de LDL-c, com 72,5% dos pacientes alcançando uma redução ≥ 50%.^373^ Além disso, o evolocumabe reduziu os níveis de Lp(a), um fator provavelmente importante em PVHIV, tendo em vista o processo inflamatório do estado de infecção crônica pelo vírus.^373^

Atingir as metas de LDL-c em PVHIV permanece um desafio clínico relevante, marcado por barreiras como interações medicamentosas, baixa adesão ao tratamento e subutilização das opções terapêuticas disponíveis. Apesar da importância consolidada das estatinas como base do tratamento hipolipemiante, a incorporação de novas estratégias – especialmente as terapias anti-PCSK9 – representa uma alternativa promissora para otimizar os desfechos cardiovasculares nessa população de alto risco. O avanço terapêutico, aliado à personalização do cuidado, pode contribuir significativamente para a superação dessas limitações e para a redução do risco residual.^375 , 376^

**Table:** 

Recomendação	Força da recomendação	Certeza da evidência
Para PVHIV, as estatinas devem ser consideradas como terapia de primeira linha para a redução do LDL-c e do risco cardiovascular, de acordo com a meta pertinente. A escolha da estatina deve levar em consideração risco de interação medicamentosa.	FORTE	ALTA
Para PVHIV com intolerância às estatinas ou redução insuficiente do LDL-c, o ezetimiba deve ser adicionado.	FORTE	MODERADA
Para PVHIV, a terapia anti-PCSK9, como o evolocumabe, devem ser considerados em PVHIV com alto risco cardiovascular e controle inadequado do LDL-c, apesar da terapia máxima.	FORTE	MODERADA

PVHIV: Pessoas Vivendo com HIV; LDL-c: colesterol da lipoproteína de baixa densidade; PCSK9: proproteína convertase subtilisina/quexina tipo 9.

### 9.3. Diabetes

A DM é um distúrbio metabólico que frequentemente predispõe a DCV, tornando a DCV uma das principais causas de morbidade e mortalidade em pacientes com DM tipo 1 e tipo 2 (DM1 e DM2). Dados recentes indicam que a DM *per se* aumenta o risco de DCV em cerca de 2 vezes, em média, mas o risco está sujeito a uma grande variação, dependendo da população e da terapia preventiva adotada.^377 , 378^ É fundamental destacar que indivíduos com DM e DAC apresentam um risco significativamente maior de eventos cardiovasculares futuros. No caso da DM2, o risco de DCVA é amplamente influenciado pela presença de lesões em órgãos-alvo, como nefropatia (microalbuminúria), neuropatia ou retinopatia, com o risco aumentando conforme o número de condições patológicas presentes.^379^

Um bom controle glicêmico na DM1 leva a menos eventos cardiovasculares. No entanto, a presença de DCV em doentes com DM2 é independente do controle glicêmico intensivo.^380^ Além da resistência e deficiência de insulina, a dislipidemia é uma comorbidade frequente em pacientes portadores de diabetes e pode influenciar significativamente sua evolução clínica. A elevação dos TGs ou os baixos níveis de HDL-c em jejum ou no estado pós-prandial são observados em cerca de metade das pessoas com DM2^381^ e também estão frequentemente presentes em pessoas com adiposidade abdominal, resistência à insulina ou tolerância à glicose diminuída.^382^

#### 9.3.1. Caraterísticas Específicas da Dislipidemia na Resistência à Insulina e no Diabetes Mellitus de Tipo 2

A dislipidemia diabética é um conjunto de alterações lipídicas e das lipoproteínas plasmáticas que estão metabolicamente inter-relacionadas. O aumento das grandes partículas de VLDL no DM2 inicia uma sequência de eventos que gera remanescentes aterogênicos, partículas de LDL pequenas e densas e partículas HDL ricas em TG.^383^ As alterações de composição das partículas de LDL e HDL têm impacto nas suas funções. Observamos um aumento dos níveis de ApoC-III, que impede, assim, a depuração das lipoproteínas ricas em TG e dos remanescentes. Isso resulta em um tempo de permanência prolongado dessas partículas na circulação,^382^ tornando-as mais aterogênicas. Esse catabolismo defeituoso das lipoproteínas ricas em TG parece ser um fator mais importante para a elevação dos TGs plasmáticos do que o aumento da taxa de produção, levando, assim, a um excesso de partículas remanescentes. Em conjunto, esse perfil também é caracterizado por um aumento de partículas contendo ApoB. É importante notar que as lipoproteínas ricas em TG – incluindo os quilomícrons, as VLDL e os seus remanescentes – transportam uma única molécula de ApoB, tal como as partículas de LDL. Por conseguinte, a natureza maligna da dislipidemia diabética nem sempre é revelada pelas medidas lipídicas utilizadas na prática clínica, uma vez que os níveis de LDL-c podem permanecer dentro dos limites da normalidade. Assim, o colesterol não-HDL ou a ApoB são melhores marcadores das lipoproteínas ricas em TG e dos remanescentes.^43^

#### 9.3.2. Tratamento para Dislipidemia em Pacientes Diabéticos

Mudanças no estilo de vida

- Dieta: a adoção de uma dieta balanceada, rica em ácidos graxos insaturados (como os encontrados em azeite de oliva, nozes e abacate) e com baixo teor de gorduras saturadas e trans, pode ajudar a melhorar os níveis lipídicos. A ingestão adequada de fibras também é importante para reduzir os níveis de LDL e TG;
- Exercício físico: a atividade física regular, especialmente exercícios aeróbicos e de resistência, pode aumentar o HDL e diminuir os TG. O exercício também ajuda a melhorar a sensibilidade à insulina;
- Perda de peso: a redução de peso, particularmente a redução da gordura visceral, pode melhorar o perfil lipídico e a resistência à insulina.

#### 9.3.3. Tratamento Medicamentoso

- **Estatinas:** o LDL-c é o principal alvo da terapia hipolipemiante em pacientes com DM. Estudos realizados especificamente em pessoas com DM2, bem como subgrupos de indivíduos com DM em grandes ensaios com estatinas, têm demonstrado de forma consistente benefícios significativos da estatina na redução de desfechos CV em pessoas com DM2.384 A terapêutica com estatinas reduz em torno de 23% a incidência de eventos cardiovasculares graves em 5 anos para cada redução de 38,4 mg/dL, independentemente do nível inicial de LDL-c ou de outras características basais, segundo a metanálise do CTT.
- **Ezetimiba:** este medicamento pode ser usado em conjunto com estatinas para reduzir ainda mais os níveis de LDL, especialmente em pacientes com alto risco cardiovascular. A ezetimiba reduz o colesterol LDL em 24% e, quando adicionada à terapia com estatinas, diminui o risco de eventos vasculares maiores.385 A redução do risco relativo de eventos cardiovasculares é proporcional à redução absoluta de LDL-c e consistente com o impacto das estatinas. O subgrupo de pacientes com DM no IMPROVE-IT teve uma taxa mais elevada de eventos vasculares maiores do que os doentes sem DM (46% versus 31%, respectivamente). A ezetimiba pareceu ser particularmente eficaz em pacientes com DM, com uma redução do risco relativo de 15% (IC95%, 6%-22%) e uma redução do risco absoluto de 5,5%.284
- **Terapia anti-PCSK9:** para pacientes diabéticos com dislipidemia grave e alto risco cardiovascular, terapia anti-PCSK9, como o alirocumabe e evolocumabe, são opções terapêuticas eficazes para reduzir o colesterol LDL. Essas medicações reduzem os níveis de LDL-c em 60% e diminuem o risco de eventos vasculares maiores.168 No estudo FOURIER, a redução do risco relativo de eventos cardiovasculares foi semelhante em pacientes com e sem DM. No estudo FOURIER, a redução do risco relativo de eventos vasculares maiores foi semelhante em pacientes com e sem DM, porém, devido ao maior risco basal em pacientes com DM, as reduções absolutas de risco são maiores em pacientes com DM (redução absoluta de 2,7% em eventos vasculares maiores ao longo de 3 anos).196 Os mesmos benefícios também foram demonstrados para pacientes diabéticos após SCA no estudo ODYSSEY.197 Mais recentemente, o uso de inclisirana proporcionou uma redução substancial e sustentada do colesterol LDL-c em todos os estratos glicêmicos e de IMC.386
- **Fibratos:** os fibratos, agonistas do receptor-α ativado por proliferadores de peroxissoma, são potentes fármacos modificadores dos lípides. Os seus principais efeitos são a redução dos TG e o aumento dos níveis de HDL-c. Vários estudos controlados não demonstraram os seus benefícios na redução do risco cardiovascular, especialmente como "complemento" da terapêutica com estatinas. No entanto, análises subsequentes de grandes ensaios clínicos, metanálises e provas do mundo real propuseram o seu potencial em populações específicas de doentes com dislipidemia aterogênica e síndrome metabólica.387 Recentemente, em pacientes com DM2, hipertrigliceridemia leve a moderada e níveis baixos de colesterol HDL e LDL, a incidência de eventos cardiovasculares não foi menor entre os que receberam pemafibrato do que entre os que receberam placebo, embora o pemafibrato tenha reduzido os níveis de triglicérides, colesterol VLDL, colesterol remanescente e apolipoproteína C-III.303 Embora com resultado neutro, há indicadores que sugerem um potencial terapêutico nas complicações isquêmicas microvasculares associadas à doença arterial periférica, com análises subsequentes que demonstraram uma redução na incidência de ulceração isquêmica ou gangrena dos membros inferiores.388 Dados emergentes do PROMINENT e de estudos experimentais também sugerem benefícios com o pemafibrato na doença hepática não alcoólica e na microangiopatia associada à diabetes, que merecem um estudo mais aprofundado.389 O estudo LENS avaliou papel do fenofibrato em portadores de retinopatia diabética leve. Foram incluídos 1151 participantes randomizados para uso de fenofibrato versus placebo. Durante mediana de 4 anos, houve progressão de retinopatia em 131 (22,7%) de 576 participantes do grupo fenofibrato versus 168 (29,2%) de 575 do grupo placebo (HR: 0,73, IC 95% 0,58-0,091, p=0,006).390
- **Ácidos graxos ômega-3:** os suplementos de ômega-3 podem ser usados para reduzir TG elevados e melhorar o perfil lipídico em pacientes com diabetes. Os ácidos graxos ômega-3, especialmente o EPA, são estudados por sua capacidade de reduzir os níveis de TG. O estudo REDUCE-IT mostrou que o icosapenta etila (IPE), uma forma purificada de EPA, reduziu eventos cardiovasculares em pacientes diabéticos tipo 2 com doença cardiovascular estabelecida e níveis de TG entre 135 e 499 mg/dL.305 Embora outros estudos com EPA não tenham mostrado resultados consistentes, o REDUCE-IT é o mais relevante para essa população. A dose de 4 g/dia de IPE reduziu de forma significativa os eventos cardiovasculares em diabéticos, sendo considerada eficaz, mas com a recomendação de monitoramento devido aos efeitos antiplaquetários do suplemento.

Na presente diretriz, incluímos um fluxograma de risco e tratamento específico para pacientes com diabetes, reconhecendo que se trata de uma população com risco cardiovascular elevado e características clínicas particulares. O tratamento hipolipemiante nessa população tem impacto mais significativo, dado o risco absoluto aumentado de eventos cardiovasculares. Por isso, recomendamos direcionamento para os capítulos de estratificação de risco, definição de metas terapêuticas e estratégias de tratamento. Destacamos como metas principais o LDL-c e, como coprimária, o não-HDL-c, além de enfatizar a importância da dosagem de ApoB, especialmente em pacientes com diabetes, devido à maior presença de partículas aterogênicas que contêm ApoB.

**Table:** 

Recomendação	Força da recomendação	Certeza da evidência
Para população com diabetes *mellitus* , a estatina é o hipolipemiante de escolha inicial para pacientes com LDL-c acima da meta estabelecida.	FORTE	ALTA
Para população com diabetes *mellitus* , ezetimiba pode ser utilizado para os indivíduos que permanecem com LDL-c acima da meta, a despeito de terapia com estatina em dose máxima tolerada.	FORTE	MODERADA
Para população com diabetes *mellitus* , a terapia anti-PCSK9 pode ser utilizado para os indivíduos que permanecem com LDL-c acima da meta, a despeito de terapia com estatina em dose máxima tolerada e ezetimiba.	FORTE	MODERADA
Para população com diabetes *mellitus* e retinopatia leve, o fenofibrato pode ser utilizado para redução da progressão da retinopatia diabética.	FORTE	MODERADA

LDL-c: colesterol da lipoproteína de baixa densidade; PCSK9: proproteína convertase subtilisina/quexina tipo 9.

### 9.4. Hipotireoidismo

O hipotireoidismo é uma disfunção endócrina comum, que afeta entre 4 e 10% da população na forma subclínica e cerca de 0,3 a 0,4% na forma clínica. É mais prevalente em mulheres, com uma razão de até 4:1 em relação aos homens.^391 , 392^ Pode ser classificado como clínico (TSH elevado com T4 livre reduzido) ou subclínico (TSH elevado com T4 livre normal). Ambas as formas impactam significativamente o metabolismo lipídico e o risco cardiovascular.

Os hormônios tireoidianos, especialmente o T3, regulam a expressão hepática dos receptores de LDL (LDL-R) e a atividade da lipase lipoproteica (LPL). No hipotireoidismo, a redução da expressão do LDL-R resulta em menor depuração de LDL-c e consequente elevação de seus níveis plasmáticos.^393^ A redução da LPL compromete a remoção de TG, contribuindo para hipertrigliceridemia. Também se observa aumento da Lp(a) e acúmulo de LDL pequenas e densas, com maior potencial aterogênico.^394 , 395^

Essas alterações contribuem para um perfil lipídico pró-aterogênico, mesmo em pacientes com hipotireoidismo subclínico. O estado pró-inflamatório associado ao hipotireoidismo, somado à disfunção endotelial, também cria um ambiente favorável ao desenvolvimento de DCV.

Estudos demonstram que TSH persistentemente elevado está associado ao aumento da espessura médio-intimal carotídea, rigidez arterial e maior incidência de eventos cardiovasculares, como infarto do miocárdio e AVC.^396^ A dislipidemia induzida por hipotireoidismo também pode ser menos responsiva às estatinas até que a função tireoidiana esteja normalizada.^397^

O tratamento com levotiroxina está indicado em todos os casos de hipotireoidismo clínico. No subclínico, a reposição hormonal deve ser considerada quando o TSH é ≥ 10 mUI/L ou em pacientes com sintomas típicos, doença cardiovascular estabelecida, diabetes ou múltiplos fatores de risco.^398^ O tratamento também deve ser considerado em pacientes com hipotireoidismo subclínico, com TSH entre 4,5 e 9,9 mUI/L, associado à dislipidemia.^399^ É importante monitorar os exames laboratoriais periodicamente (geralmente após 6 a 8 semanas) para ajustar a dosagem e avaliar a resposta ao tratamento com levotiroxina. A normalização da função tireoidiana promove, em sua maioria, a melhora do perfil lipídico, com redução notável dos níveis de LDL e do CT.^399^

Importante ressaltar que enquanto os níveis hormonais não estiverem normalizados, pacientes podem ter uma maior vulnerabilidade a efeitos colaterais secundários às estatinas, como mialgia (dor muscular) ou, em casos raros, rabdomiólise. A normalização dos níveis de TSH e T4 livre com reposição hormonal geralmente reduz esses riscos, tornando o uso de estatinas mais seguro.

A decisão de iniciar estatinas deve ser individualizada, considerando o risco cardiovascular global, a resposta ao tratamento do hipotireoidismo e o monitoramento rigoroso dos efeitos colaterais.

Por outro lado, o hipertireoidismo geralmente cursa com um perfil hipolipidêmico, com redução de CT, LDL-c e TG, devido ao aumento da expressão de LDL-R e do metabolismo hepático de lipoproteínas.^393^ No entanto, pode haver também queda do HDL-c, atribuída à maior atividade da CETP.

**Table:** 

Recomendação	Força da recomendação	Certeza da evidência
Em pacientes com hipotireoidismo clínico e dislipidemia, recomenda-se a favor da reposição hormonal com levotiroxina, visando a normalizar o TSH e melhorar o perfil lipídico.	FORTE	ALTA
Em pacientes com hipotireoidismo clínico e dislipidemia, recomenda-se a favor do uso de estatinas em pacientes cuja dislipidemia persiste após normalização da função tireoidiana, principalmente em presença de risco cardiovascular elevado.	FORTE	MODERADA
Em pacientes com hipotireoidismo subclínico e dislipidemia, recomenda-se a favor do uso de levotiroxina se o TSH estiver entre 4,5 e 9,9 mUI/L e houver sintomas de hipotireoidismo ou alto risco cardiovascular.	FORTE	MODERADA
Em pacientes com hipotireoidismo subclínico e dislipidemia, recomenda-se a favor do uso de levotiroxina se o TSH estiver acima de 10 mUI/L.	FORTE	ALTA

### 9.5. Doença Renal Crônica

A doença renal crônica (DRC) é reconhecida como um importante fator de risco cardiovascular independente. A associação entre DRC e doença cardiovascular representa uma das principais causas de morbimortalidade entre os pacientes dialíticos e transplantados renal.^400^ As dislipidemias em pacientes com DRC apresentam características distintas das observadas na população geral. Essas alterações no metabolismo lipídico são caracterizadas principalmente pela redução da atividade da lipase lipoproteica, hipertrigliceridemia, acúmulo de lipoproteínas remanescentes (VLDL e IDL aumento de LDL), alterações na composição e função do HDL. Essas alterações do perfil lipídico, somadas à inflamação crônica e estresse oxidativo, acabam por aumentar significantemente o risco de aterogênese, mesmo em indivíduos com valores normais de CT ou LDL-c, o que dificulta a estratificação de risco com parâmetros tradicionais.^401 , 402^

Dessa forma, o manejo terapêutico é fundamental para a redução do risco cardiovascular, da progressão da doença renal e da inflamação crônica, além de contribuir indiretamente para o controle de doenças associadas, como diabetes e a síndrome metabólica.

A estratégia terapêutica principal requer o uso de estatinas, com ou sem ezetimiba, focando na redução do risco cardiovascular absoluto, a despeito de metas específicas de LDL-c, especialmente em pacientes com redução da taxa de filtração glomerular.^55 , 403 - 405^ A combinação de estatina com ezetimiba resultou na redução adicional de LDL-c em pacientes pré-dialíticos.^406 , 407^ Em se tratando de pacientes dialíticos, quando optado por diálise peritoneal, admite-se a introdução de estatina em pacientes de muito alto risco cardiovascular, pois o metabolismo lipídico é preservado, o que pode indicar maior benefício da terapia medicamentosa.^55 , 400 , 401^ Em pacientes em programa de hemodiálise, o benefício da introdução da medicação hipolipemiante é controverso, além do potencial risco de interação e eventos adversos. Entretanto, naqueles indivíduos em uso prévio de terapia recomenda-se a sua manutenção.^400 , 406 - 409^ Na subpopulação de transplantados renais, o risco cardiovascular é elevado pelos fatores de risco clássicos somados aos efeitos adversos dos imunossupressores. O uso de estatinas está associado à redução de eventos cardiovasculares e mortalidade.^401^ Estatinas como sinvastatina e atorvastatina possuem metabolismo hepático via CYP3A4, o que pode gerar interações relevantes com imunossupressores, como a ciclosporina, recomendando-se o uso preferencial de estatinas com menor risco de interação, como pravastatina, fluvastatina ou rosuvastatina.^401^ Terapias lipídicas diferentes das estatinas carecem de dados robustos na população com DRC. Outras intervenções, como dieta balanceada com baixo teor de gordura saturada, cessação do tabagismo, atividade física regular e controle de peso, têm benefícios comprovados sobre o perfil lipídico, a função endotelial e o controle de comorbidades associadas; por isso, devem fazer parte da orientação do paciente.^55 , 401 , 403 , 404^

**Table:** 

Recomendação	Força da recomendação	Certeza da evidência
Em indivíduos com DRC estágio 1-3 e risco CV aumentado, recomenda-se a favor do uso de estatinas de alta potência para reduzir risco CV.	FORTE	ALTA
Em indivíduos com DRC estágio 1-3 e risco CV aumentado que não atingiram as metas, recomenda-se a favor da associação de ezetimiba às estatinas de alta potência.	FORTE	MODERADA
Em indivíduos com DRC estágio 4-5 não dialítico, recomenda-se a favor de iniciar estatina de alta potência, associada ou não ao uso de ezetimiba.	FORTE	ALTA
Em indivíduos com DRC em programa de diálise, sem doença cardiovascular estabelecida, recomenda-se contra o início de estatinas.	FORTE	ALTA

LDL-c: colesterol da lipoproteína de baixa densidade; PCSK9: proproteína convertase subtilisina/quexina tipo 9.

### 9.6. Obesidade

A dislipidemia e a obesidade são entidades clínicas de alta prevalência global, associadas à significativa morbidade e aumento do risco de eventos cardiovasculares maiores. A coexistência dessas condições potencializa distúrbios metabólicos, promovendo um cenário pró-aterogênico, inflamatório e propício ao desenvolvimento de doença aterosclerótica macrovascular.

A associação de obesidade e dislipidemia multiplica o risco de doença arterial coronariana, AVC, IC e doença arterial periférica.^410 , 411^ O padrão lipídico característico do paciente obeso – hipertrigliceridemia, HDL-c baixo e formação de LDL pequenas e densas – compõe o fenótipo lipídico mais associado à síndrome metabólica e à aterogênese acelerada.^412^ O excesso de tecido adiposo, principalmente visceral, promove resistência à insulina, mediada por adipocinas inflamatórias, como TNF-α e IL-6, e pela diminuição da adiponectina.^413^ Essa resistência leva ao aumento da lipólise, elevação de ácidos graxos livres circulantes e influxo hepático dessas moléculas, resultando em aumento da produção de VLDL e hipertrigliceridemia.^413^ Estudos apontam que a redução de 5 a 10% no peso corporal já acarreta impactos significativos no perfil lipídico e glicêmico.^414^

Intervenções não farmacológicas são imprescindíveis, constituindo a primeira linha terapêutica. Recomenda-se dieta hipocalórica balanceada, reduzida em gorduras saturadas (menos de 7% das calorias), aumento da ingestão de fibras solúveis e fitoesteróis, além da prática de pelo menos 150 minutos por semana de exercício aeróbico em intensidade moderada.^415^

A necessidade de iniciar hipolipemiante depende do perfil lipídico e do risco cardiovascular calculado. As estatinas reduzem de forma comprovada a morbimortalidade cardiovascular e devem ser a base do tratamento na presença de LDL-c elevado, independentemente da presença de obesidade.^416^ A ezetimiba pode ser associada à estatina quando necessário atingir alvos mais rigorosos de LDL-c.^165^ A terapia anti-PCSK9 são indicados a pacientes de muito alto risco ou com HF com LDL-c refratário, apesar do tratamento máximo tolerado. Os fibratos são utilizados prioritariamente frente à hipertrigliceridemia igual ou superior a 500 mg/dL, para redução do risco de pancreatite.^303^

Agonistas do receptor de GLP-1, como liraglutida e semaglutida, além de promoverem perda ponderal significativa, são associados à melhora do perfil lipídico e contribuem para reduções discretas, mas consistentes, de TG e CT.^417^ Evidência recente do estudo SELECT demonstrou que, em pacientes com doença cardiovascular estabelecida e muito alto risco cardiovascular, o uso de semaglutida 2,4 mg resultou em uma redução significativa de eventos cardiovasculares maiores, independentemente da perda de peso, reforçando seu papel como agente cardioprotetor, além do efeito sobre o peso corporal.^219 , 418^

A cirurgia bariátrica continua sendo a intervenção mais eficaz para perda de peso sustentada e melhora significativa dos parâmetros metabólicos, incluindo normalização ou redução expressiva dos níveis de LDL-c e TG, aumento de HDL-c, além de melhorar a resistência à insulina.^4^^19^

**Table:** 

Recomendação	Força da recomendação	Certeza da evidência
Em indivíduos com dislipidemia secundária à obesidade, recomenda-se a favor de intervenções não farmacológicas, que constituem a linha no tratamento da dislipidemia nessa condição.	FORTE	ALTA
Em indivíduos com dislipidemia secundária à obesidade, recomenda-se a favor do uso de estatinas como base do tratamento farmacológico.	FORTE	ALTA
Em indivíduos com dislipidemia secundária à obesidade, recomenda-se a favor do uso de fibratos quando os triglicérides estiverem ≥ 500 mg/dL, visando à redução do risco de pancreatite.	FORTE	MODERADA
Em indivíduos com dislipidemia secundária à obesidade, recomenda-se contra o uso de fibratos para redução do risco cardiovascular ou para reduzir o risco de pancreatite quando os triglicérides estiverem < 500 mg/dL.	FORTE	ALTA
Em indivíduos com dislipidemia secundária à obesidade, recomenda-se a favor do uso de análogos do GLP-1 por seu efeito duplo (perda de peso e redução de eventos cardiovasculares).	FORTE	ALTA
Em indivíduos com dislipidemia secundária à obesidade, recomenda-se a favor da cirurgia bariátrica para melhorar o perfil lipídico e reduzir eventos cardiovasculares.	FORTE	MODERADA

### 9.7. Idosos

O risco de DCV aumenta com a idade,^420^ bem como o risco de mortalidade não cardiovascular.. O tratamento de fatores de risco (FR) para DCV deve ser cuidadosamente avaliado para equilibrar os benefícios e riscos nessa população, que podem diferir em indivíduos com expectativa de vida limitada.^421 , 422^ Importante salientar que idosos geralmente apresentam maior risco de eventos adversos e efeitos colaterais de medicamentos.^423 , 424^ Assim, é fundamental identificar aqueles indivíduos que podem se beneficiar do tratamento preventivo.

A decisão quanto ao tratamento hipolipemiante dos idosos deve levar em conta certas particularidades, como a farmacocinética dos medicamentos, menores evidências de benefícios clínicos em determinadas faixas etárias e elevada prevalência de aterosclerose subclínica naqueles com mais de 65 anos de idade.^425^

As concentrações de CT são mais elevadas até a sexta década de vida, caindo ligeiramente com o avançar da idade. Em geral, mesmo nas dislipidemias de caráter genético, não ocorrem grandes elevações de CT, TG e LDL-c, sendo frequentes as dislipidemias secundárias a hipotireoidismo, DM, intolerância à glicose, obesidade, síndrome nefrótica e uso de medicamentos, como diuréticos tiazídicos e bloqueadores beta-adrenérgicos não seletivos. A associação entre altas concentrações de colesterol e risco aumentado de DAC em adultos de meia idade e no início da terceira idade se enfraquece com o progredir da idade,^426^ consequência de fragilidade ou eventos competitivos. No *Prospective Studies Collaboration* , com resultados de 61 estudos prospectivos observacionais, evidenciou-se a associação entre CT e mortalidade vascular em 900.000 indivíduos sem história de doença vascular, dos 40 aos 89 anos. A redução de risco relativo de doença isquêmica cardíaca para cada redução de 39 mg/dL-c do colesterol total é menor nas faixas etárias mais avançadas, porém o como a taxa absoluta desse evento aumentou com a idade mais velha, houve maior diferença absoluta na faixa etária mais avançada.

A despeito de maiores evidências sobre o papel das dislipidemias na patogênese da aterosclerose e da DAC em estudos observacionais e experimentais em indivíduos de meia-idade,^427^ estudos posteriores forneceram informações importantes, que podem auxiliar na decisão de tratar também a população de idosos.^428^ No idoso, o tratamento da dislipidemia deve considerar o estado geral e mental do paciente, as condições socioeconômicas, o apoio familiar, as comorbidades presentes e os outros fármacos em uso, que podem interagir com os hipolipemiantes e, assim, influenciar a adesão e a manutenção da terapêutica.^429^

### 9.8. Tratamento Não Farmacológico

As orientações para terapia não farmacológica devem obedecer aos mesmos princípios de indicação aplicados aos não idosos, observando-se as necessidades de aporte calórico, proteico e vitamínico, com recomendação da prática de atividade física.

### 9.9. Tratamento Farmacológico

As estatinas constituem os fármacos de escolha nesta população. Evidências de estudos em idosos, como o *PROspective Study of Pravastatin in the Elderly at Risk* , com a pravastatina,^430^ do *Heart Protection Study* , com um grande número de indivíduos acima de 65 anos de idade,^431^ análise exploratória do estudo JUPITER ( *Justification for the Use of statins in Prevention: an Intervention Trial Evaluating Rosuvastatin* )^432^ em idosos com 70 anos ou mais, do HOPE-3, onde a metade dos pacientes tinha > 65 anos,^433^ e a metanálise do CTT ( *Cholesterol Treatment Trialists* ) demonstraram redução do risco relativo de eventos vasculares nesse subgrupo de pacientes recebendo terapia com estatina.^166^

O EWTOPIA 75 ( *Ezetimiba Lipid-Lowering Trial on Prevention of Atherosclerotic Cardiovascular Disease in 75 or Older* ) avaliou a eficácia do tratamento com ezetimiba na prevenção de eventos cardiovasculares em idosos >75 anos, mostrando redução do desfecho primário, dos desfechos secundários e de revascularização coronária, sem diferenças para morte por todas as causas ou AVC.^434^

O estudo STAREE ( *Statins in Reducing Events in the Elderly* )^435^ está investigando os efeitos da terapia com estatinas na prevenção primária de eventos cardiovasculares e na redução da mortalidade em idosos saudáveis acima de 70 anos, com resultados previstos para 2025.

A *US Preventive Services Task Force* afirma que não há evidências suficientes para iniciar tratamento de dislipidemias em adultos acima de 75 anos sem história de DCV prévia.^436^

O SCORE2-OP ( *Systematic Coronary Risk Evaluation 2 – Older Persons* ), utilizado pela European Society of Cardiology para estimar o risco cardiovascular em indivíduos >70 anos, pode auxiliar na tomada de decisão terapêutica para essa população.^159^

Assim, recomenda-se que o tratamento da dislipidemia em idosos seja individualizado, levando em consideração fatores como comorbidades, expectativa de vida e possíveis interações medicamentosas. O tratamento deve ser recomendado para idosos com alto risco ou muito alto risco, e as metas são semelhantes às dos não idosos.

**Table:** 

Recomendação	Força da recomendação	Certeza da evidência
Após os 75 anos de idade, recomenda-se a favor da individualização das doses dos hipolipemiantes, de acordo com a fragilidade, presença de comorbidades, expectativa de vida e o uso de polifarmácia.	FORTE	MODERADA

### 9.10. Crianças

A aterosclerose é um processo progressivo que se inicia ainda na infância, sendo agravado por fatores como colesterol elevado, obesidade e síndrome metabólica – condições que contribuem para o aumento do risco cardiovascular na idade adulta. O conhecimento sobre as dislipidemias em crianças tem evoluído de forma significativa, permitindo diagnósticos mais precoces e abordagens terapêuticas mais eficazes. Importante destacar que, nessa faixa etária, as metas de LDL-c são diferentes das aplicadas à população adulta, sendo mais brandas e ajustadas à fase de desenvolvimento. O objetivo central permanece a prevenção precoce, com segurança e efetividade, evitando tanto a progressão da aterosclerose quanto efeitos adversos no crescimento e maturação metabólica.

#### 9.10.1. Perfil Lipídico na Infância

Em crianças e adolescentes, a análise deve ser feita após jejum de 8 a 9 horas, incluindo CT, HDL-c, TG e LDL-c, que pode ser calculado ou medido diretamente.^437^

#### 9.10.2. Rastreamento

Rastreamento universal: entre 9 e 11 anos e entre 17 e 21 anos, preferencialmente em jejum, independentemente de histórico familiar.^438^

Rastreamento seletivo: entre 2 e 8 anos e 12 e 16 anos para crianças com fatores de risco como:^438^

- histórico familiar de hipercolesterolemia ou doença cardiovascular prematura;
- sobrepeso/obesidade;
- diabetes, hipertensão ou tabagismo.

#### 9.10.3. Dislipidemias Primárias

São genéticas, presentes desde cedo, e dividem-se em monogênicas ou poligênicas. As principais formas são:

- Hipercolesterolemia Familiar Heterozigótica (HFHe);^87^
- doença genética dominante, com elevação do LDL-c desde o nascimento;
- afeta 1 em cada 250-300 pessoas e está entre as causas hereditárias mais comuns de doença cardiovascular;439
- é causada por mutações nos genes do receptor de LDL, ApoB, PCSK9, entre outros.4^39 , 440^

Diagnóstico: LDL-c > 190 mg/dL ou > 160 mg/dL com histórico familiar positivo. O teste genético é útil, mas pode ser substituído por avaliação fenotípica. O LDL-c deve ser medido pelo menos 2 vezes durante 3 meses.^87^

Tratamento: inclui dieta, atividade física e estatinas, que devem ser iniciadas entre 8 e 10 anos (ou mais cedo em casos graves). Estatinas reduzem o LDL-c em média 32% e são seguras. Estudos demonstram que o início precoce reduz drasticamente eventos cardiovasculares futuros. A meta terapêutica é LDL-c < 130 mg/dL.^441 , 442^

Terapias adicionais:

- ezetimiba reduz LDL-c em até 27% e pode ser associado às estatinas;^443^
- terapia anti-PCSK9 (como evolocumabe e alirocumabe) são indicados em casos resistentes ou com intolerância às estatinas;
- evolocumabe reduz o LDL-c em aproximadamente 44% em crianças e adolescentes. Ambos são aprovados para uso pediátrico.^444^

#### 9.10.4. Hipercolesterolemia Familiar Homozigótica

- Forma grave e rara (1:300.000), causada por mutações herdadas dos dois pais;^84^
- LDL-c geralmente > 400 mg/dL, com xantomas precoces e alto risco de doença cardiovascular ainda na infância;^84^
- Diagnóstico: baseado em níveis extremamente altos de LDL-c, sinais clínicos e histórico familiar. O teste genético confirma a condição e orienta o tratamento e rastreamento familiar;^84^
- Tratamento:
  – início precoce com estatinas e ezetimiba (a partir dos 2 anos);^84^
  – LDL-aférese é indicada antes dos 5 anos, especialmente nos casos graves;^84^
  – terapia anti-PCSK9, se a resposta for maior do que lomitapida (ainda não disponível para crianças), e evinacumabe são opções emergentes;^84^
- Meta de LDL-c < 115 mg/dL, sendo mais baixa em casos com DCVA estabelecida, embora difícil de atingir.^84^

#### 9.10.5. Hipertrigliceridemias

Decorrem da produção aumentada de VLDL ou redução na lipólise. Níveis de TG entre 175-885 mg/dL são leves a moderados; acima de 885 mg/dL são graves. Causas secundárias incluem dieta inadequada, doenças endócrinas, medicamentos e álcool.^313 , 445^

O tratamento inclui: intervenções de estilo de vida – dieta e atividade física; e farmacoterapia – estatinas (reduzem TG até 30%) a partir dos 10 anos; fibratos e ômega-3 podem ser usados em TG > 400 mg/dL.^445^

#### 9.10.6. Hipertrigliceridemia Monogênica (Hipertrigliceridemias Graves)

SQF: doença rara, recessiva, causada por mutações na LPL e genes relacionados. Manifesta-se na infância com TG > 1.000 mg/dL, pancreatite recorrente, lipemia retiniana e xantomas. O tratamento inclui dieta com restrição severa de gordura (8 a 10% das calorias) e uso de ácidos graxos de cadeia média.^97^

SQM: causada por múltiplos genes e agravada por fatores como diabetes, obesidade e certos medicamentos. É mais comum que a SQF. Responde bem a mudanças no estilo de vida e tratamento das comorbidades.^97^

#### 9.10.7. Dislipidemias Secundárias

Causadas por doenças ou medicamentos. As mais comuns incluem:^437^

- DM tipo 1 e 2;
- hipotireoidismo;
- doença renal crônica;
- lúpus;
- uso de isotretinoína, corticoides, contraceptivos orais;
- doença hepática, entre outras.

O tratamento da condição de base costuma normalizar o perfil lipídico.

#### 9.10.8. Indicação do Uso de Estatinas de acordo com o Risco nas Dislipidemias Secundárias, Condições de Alto Risco ou Presença de Fatores de Risco (Valores de Corte para Iniciar Tratamento)

Crianças > 10 anos:

- LDL-c ≥ 130 mg/dL com múltiplos fatores de risco (diabetes, hipertensão);
- LDL-c ≥ 160 mg/dL com um único fator de risco, como histórico familiar de doença cardíaca prematura, ou múltiplos fatores de risco (hipertensão que não requer tratamento e obesidade);
- LDL-c ≥ 190 mg/dL sem fatores de risco.

**Table:** 

Recomendação	Força da recomendação	Certeza da evidência
Para população pediátrica, recomenda-se a favor da dosagem do perfil lipídico completo de forma universal entre 9 e 11 anos de idade.	FORTE	MODERADA
Para população pediátrica com fatores de risco (citados no texto), recomenda-se a favor de dosagem do perfil lipídico completo a partir dos 2 anos de idade.	FORTE	MODERADA
Para população pediátrica, recomenda-se a favor de estimular modificação de estilo de vida com orientação nutricional, controle de peso e atividade física compatível com a idade e quadro clínico.	FORTE	ALTA
Para população pediátrica que está acima da meta de LDL-c, a despeito de modificação do estilo de vida, recomenda-se a favor da terapia hipolipemiante com estatina a partir dos 8 anos de idade.	FORTE	MODERADA
Para população pediátrica que está acima da meta de LDL-c, a despeito de modificação do estilo de vida, recomenda-se a favor da terapia por ezetimiba a partir dos 6 anos de idade e terapia combinada com estatina a partir dos 8 anos de idade.	CONDICIONAL	BAIXA
Para população pediátrica, de acordo com avaliação médica e risco do paciente e conforme o nível do LDL-c, que já esteja em tratamento com estatina e ezetimiba, recomenda-se a favor de considerar uso de evolocumabe a partir dos 10 anos de idade ou alirocumabe a partir dos 8 anos de idade.	FORTE	MODERADA

### 9.11. Transplantados

Os avanços nas terapias contra rejeição nos pacientes transplantados aumentaram significativamente sua sobrevida, tornando as DCV a principal causa de mortalidade.^446^ Nesse contexto, a dislipidemia tem papel central, sendo altamente prevalente em pacientes submetidos a transplantes de órgãos sólidos. Estima-se que ocorra em até 80% dos transplantados renais, 70% dos hepáticos e 50% dos cardíacos, enquanto a prevalência na população geral gira em torno de 35%.^447 , 448^

Embora fatores como predisposição genética e comorbidades pré-existentes contribuam para as alterações lipídicas, o principal fator causal é o uso de imunossupressores, especialmente os inibidores da calcineurina (como ciclosporina e tacrolimo) e os corticoides. Esses agentes promovem elevação do LDL-c e TG, além de estarem associados à hipertensão arterial, resistência à insulina, hiperglicemia e DM. Enquanto as drogas como azatioprina parecem ter impacto neutro sobre o metabolismo lipídico, os efeitos de terapias mais recentes, como anticorpos monoclonais e policlonais, ainda carecem de dados robustos.^448 - 450^

A ciclosporina, aprovada em 1983, revolucionou a imunossupressão, mas afeta negativamente o metabolismo do colesterol: inibe a CYP27A1, reduzindo a síntese de ácidos biliares e a excreção hepática de colesterol; interfere na ligação do LDL-c ao seu receptor; reduz a atividade da lipase lipoproteica; além de afetar diretamente a função das células beta pancreáticas, induzindo apoptose e hipossecreção de insulina. A dislipidemia causada é semelhante à induzida pelos corticoides, mas com maior elevação de LDL-c.^451^

As DCV são a principal causa de morte após transplantes cardíacos e renais e a segunda após transplantes hepáticos.^448 - 450^ No transplante cardíaco, destaca-se a vasculopatia do enxerto, forma agressiva de aterosclerose caracterizada por hiperplasia concêntrica da íntima arterial e redução progressiva da luz vascular, sendo responsável por até 10% das mortes.^450^

Embora não sejam automaticamente classificados como categoria de risco específico, os pacientes transplantados (especialmente de órgãos sólidos como rim, coração e fígado) apresentam risco cardiovascular significativamente aumentado.

Dessa maneira, o uso de estatinas é fortemente recomendado, exceto nos primeiros 1 a 2 meses após o transplante hepático. A sugestão é iniciar com doses baixas, devido ao risco de interações medicamentosas. Importante enfatizar que o uso de sinvastatina, lovastatina e pitavastatina deve ser evitado nos pacientes que usam ciclosporina. No caso dessa associação, o FDA também recomenda precaução com a atorvastatina, embora estudos sugiram que 10 mg/dia podem ser seguros. Doses iniciais típicas incluem rosuvastatina 5 mg, pravastatina 40 mg ou fluvastatina 40 mg, com titulação gradual.^446^ As combinações de lovastatina, sinvastatina ou pitavastatina com everolimo, tacrolimo ou sirolimo também devem ser evitadas devido ao risco de rabdomiólise e toxicidade hepática.^452 , 443^

A ezetimiba é considerada alternativa segura.^55^ Até o momento, terapia anti-PCSK9 ainda não foram testados extensivamente nessa população, embora, teoricamente, não apresentem interações medicamentosas relevantes, pois não utilizam o citocromo P450. No entanto, estudos clínicos de segurança são necessários.^451^

O manejo da dislipidemia em transplantados exige individualização, pois os próprios medicamentos que prolongam a vida são os que causam distúrbio lipídico. O uso criterioso das estatinas, com atenção às interações, é fundamental. Controlar a dislipidemia deve ser prioridade, pois negligenciar esse aspecto pode comprometer a sobrevida a longo prazo desses pacientes.

**Table:** 

Recomendação	Força da recomendação	Certeza da evidência
Recomenda-se a favor de considerar todos os pacientes transplantados como tendo um agravante de risco cardiovascular.	FORTE	MODERADA
Em todos os pacientes transplantados, recomenda-se a favor de medir o perfil lipídico cerca de 2 a 3 meses após o transplante.	FORTE	MODERADA
Em pacientes transplantados, recomenda-se a favor de utilizar estatinas como primeira linha de tratamento da dislipidemia para reduzir eventos cardiovasculares.	FORTE	ALTA
Em pacientes transplantados, recomenda-se contra o uso de sinvastatina ou lovastatina associado a ciclosporina, tacrolimo, sirolimo ou everolimo.	FORTE	ALTA
Em pacientes transplantados utilizando ciclosporina recomenda-se a favor de utilizar dose máxima de 5 mg de rosuvastatina ou 10 mg de atorvastatina para evitar interação medicamentosa.	FORTE	MODERADA
Em pacientes transplantados em uso de imunossupressores, recomenda-se a favor do uso de estatinas com menor risco de rabdomiólise, como pravastatina, fluvastatina ou rosuvastatina.	FORTE	MODERADA

**Tabela 9.2:** Estatinas utilizadas em crianças e adolescentes

Medicamento	Potência	Aprovado pelo Food and Drug Administration	Idade	Dose	Redução média de LDL-c
**Atorvastatina**	Alta	Sim	≥ 10 anos	10-20 mg/dia	40%
**Fluvastatina**	Baixa	Sim	≥ 10 anos	20-80 mg/dia	34%
**Lovastatina**	Baixa	Sim	≥ 10 anos	10-40 mg/dia	17-37%
**Pitavastatina**	Alta	Sim	≥ 8 anos	1-4 mg/dia	23-39%
**Pravastatina**	Baixa	Sim	≥ 8 anos	20 mg/dia (8-13 anos)	23-33%
				40 mg/dia (14-18 anos)	
**Rosuvastatina**	Alta	Sim	≥ 7 anos	5-20 mg/dia	38-50%
**Sinvastatina**	Moderada		≥ 10 anos	10-40 mg/dia	31-41%

**Tabela 9.3:** Diversos hipolipemiantes para tratamento das dislipidemias em crianças e adolescentes

Medicamento	Potência	Aprovado pelo Food and Drug Administration	Idade	Dose	Redução média do LDL-c e TG %
**Ezetimiba**	Baixa	Sim *	> 6 anos	10 mg/dia	LDL-c ↓25-30%
**Resinas de troca**	Baixa	Sim	< ou > 10 anos	2-8 g/dia	LDL-c ↓15% a 30%
**Terapia anti- PCSK9 (evolocumabe)**	Alta	Sim	≥ 8 anos	420 mg/uma vez ao mês 140 mg/15 dias	LDL-c ↓45%
**Fibratos**	Moderada	Não	≥ 10 anos	NA	TG ↓25%
**Ômega 3**	Moderada	NA	A partir de 7 anos	1-4 g/dia	TG ↓20%
**Fitosteróis**	Baixa	NA	A partir dos 2 anos	Até 2 g/dia	LDL-c ↓10 a 15%

NA: não aplicável.

* FDA aprovou uso de ezetimiba ≥10 anos, porém a presente diretriz indica ≥ 6 anos de idade por conta de estudo clinico randomizado mostrando segurança e eficácia a partir dessa faixa etária.^443^

### 9.12 Doenças Hepáticas Crônicas

As estatinas são contraindicadas em pacientes com doença hepática descompensada ou insuficiência hepática aguda, sendo seguras na redução dos níveis lipídicos em pacientes com doença hepática compensada.^454 - 456^

#### 9.12.1. Esteatose Hepática Associada à Disfunção Metabólica

##### 9.12.1.1. Definição

A esteatose hepática associada à disfunção metabólica (MASLD) é definida como esteatose hepática em adultos que tenham pelo menos um fator de risco cardiometabólico: sobrepeso/obesidade, diabetes tipo 2, pré-diabetes, hipertensão arterial sistêmica ou dislipidemia aterogênica, na ausência de causas secundárias.^457^

##### 9.12.1.2. Prevalência e Risco Cardiovascular

É altamente prevalente e associada a aumento do risco de eventos cardiovasculares maiores.

##### 9.12.1.3. Redução do Risco Cardiovascular

É necessário tratar as comorbidades associadas à MASLD, como HAS, DM, obesidade e dislipidemia.^457^ As estatinas são a primeira escolha, pois tratam a dislipidemia aterogênica presente na MASLD, além de reduzir a morbidade e mortalidade por DCVA nesses pacientes.

##### 9.12.1.4. Desfechos Hepáticos

Estudo de coorte sugere que as estatinas na MASLD reduziram o risco de mortalidade por todas as causas, de descompensação hepática e de carcinoma hepatocelular (CHC).^458^ O uso de estatinas também foi associado à redução da progressão da rigidez hepática, um indicador da progressão da fibrose, tanto em pacientes com quanto em pacientes sem doença hepática crônica avançada.^459^ Metanálises e revisões sistemáticas indicam que as estatinas podem melhorar os testes de função hepática, diminuir o grau de esteatose e fibrose em usuários de estatinas, provavelmente mediada por mecanismos anti-inflamatórios e antifibróticos.^458^

##### 9.12.1.5. Segurança

As estatinas não devem ser suspensas em pacientes com doença hepática crônica estável e transaminases normais ou modestamente elevadas (até três vezes o limite superior da normalidade), pois não causam progressão da doença hepática. Nesses pacientes, o monitoramento das provas de função hepática deve ser realizado periodicamente.^339^

Ezetimiba: o uso de ezetimiba para melhorar evolução de MASLD ainda é controverso, porém a terapia combinada com estatina deve ser feita segundo indicações habituais para alcance de metas terapêuticas.^460^ Os dados a respeito de seu uso na insuficiência hepática ainda são limitados; dessa forma, deve-se evitar em pacientes com graus mais avançados de disfunção hepática como Child-Pugh B e C.

#### 9.12.2. Colestases Intra-Hepáticas

A colangite biliar primária (CBP) é uma doença hepática colestática autoimune, caracterizada por inflamação crônica associada à dislipidemia.^461^ Níveis elevados de CT, em grande parte, ocorrem devido à elevação da lipoproteína X, que não é aterogênica e não impacta o risco cardiovascular. O uso de hipolipemiantes é indicado na presença de fatores que contribuem para elevação do risco cardiovascular nessa população.

As estatinas não são contraindicadas em pacientes com CBP com doença hepática compensada, mas não devem ser utilizadas na doença hepática descompensada.^462^ Não há evidências do efeito das estatinas na redução dos marcadores de colestase intra-hepática na CBP.^462^ Estudos têm demonstrado segurança no uso de ezetimiba em pacientes com CBP, e associações com estatinas devem ser consideradas em pacientes com CBP que apresentem fatores de risco para DCV.^461^

#### 9.12.3. Cirrose Hepática

Embora se acreditasse que a cirrose protegia contra a doença aterosclerótica, sabe-se que a prevalência de doença arterial coronariana em pacientes com cirrose pode ser maior do que na população em geral. O risco cardiovascular varia de acordo com a etiologia da hepatopatia e é maior na cirrose causada por álcool, HCV (vírus da hepatite C) e NASH.^463^ As estatinas são seguras e podem ser usadas em pacientes com cirrose Child-Pugh classe A e B.^463 - 465^

Na cirrose Child-Pugh classe C, o uso é desaconselhado, pois a insuficiência hepática moderada a grave reduz o metabolismo do fármaco, elevando seus níveis séricos, o que predispõe a efeitos adversos.^463 , 464^ Evidências dos benefícios das estatinas na redução da hipertensão porta e descompensação hepática em pacientes com cirrose são escassas, não sendo indicadas para essa finalidade de tratamento.^463^ O impacto das estatinas nos desfechos hepáticos em pacientes com cirrose está sendo avaliado em estudos randomizados em andamento.

#### 9.12.4. Carcinoma Hepatocelular

Estudos caso-controle, feitos predominantemente em coortes de pacientes com hepatite viral, demonstraram redução de 25% na incidência de carcinoma hepatocelular (CHC) no grupo exposto às estatinas lipofílicas, comparado aos não expostos.^463^ Metanálises sugerem que a terapia com estatinas está associada à redução da incidência de CHC, mas dados prospectivos randomizados ainda são necessários

**Table:** 

Recomendação	Força da recomendação	Certeza da evidência
O uso de estatinas pode ser indicado para pacientes com esteatose hepática, na presença de enzimas hepáticas alteradas (até três vezes os valores de referência da normalidade), para redução do risco cardiovascular.	FORTE	MODERADA
O uso de estatinas pode ser indicado para pacientes com esteatose hepática, na presença de enzimas hepáticas alteradas (até três vezes os valores de referência da normalidade), para melhora da evolução hepática.	FORTE	MODERADA
A doença hepática crônica compensada (Child-Pugh A e B) não é considerada contraindicação absoluta para a início ou manutenção da terapia com estatina e terapia anti-PCSK9.	FORTE	MODERADA

### 9.13. Síndrome Cronária Aguda

O controle intensivo do colesterol após uma síndrome coronariana aguda (SCA) é um dos pilares da prevenção em indivíduos classificados como de muito alto risco, dentro do *continuum* de risco cardiovascular. Nas últimas décadas, evidências robustas têm reforçado a importância de iniciar precocemente terapias hipolipemiantes potentes, com impacto comprovado na redução de eventos cardiovasculares recorrentes e na mortalidade.

Em 2005, Fonarow demonstrou que o uso de estatina nas primeiras 24 horas de internação por SCA reduz complicações precoces e mortalidade hospitalar.^466^ Em 2008, Pitt *et al.* mostraram que os níveis de LDL-c variam minimamente nos primeiros 4 dias após o evento, validando a dosagem precoce do colesterol como guia terapêutico confiável.^467^

Com base nessas observações, consolidou-se a prática de iniciar o tratamento lipídico ainda durante a hospitalização. Estudos com inibidores da PCSK9 (iPCSK9), como alirocumabe e evolocumabe, mostram que níveis de LDL-c abaixo de 40 mg/dL estão associados à redução sustentada de eventos aterotrombóticos, com bom perfil de segurança.^203^ Dessa forma, fica claro que quanto mais intensiva for a redução do LDL-c, maior será o benefício clínico.

O registro SWEDEHEART mostrou que pacientes que atingiram a meta de colesterol não-HDL dentro de 2 meses e a mantiveram em longo prazo, apresentaram menor risco, diferentemente daqueles que tiveram tratamento escalonado lento.^468^

As diretrizes da AHA/ACC recomendam o uso sequencial e racional de três classes de fármacos: estatinas de alta potência, ezetimiba e terapia anti-PCSK9. A escolha do momento de escalonamento depende do nível basal de LDL-c e do uso prévio de estatinas.^403 , 469^ A European Society of Cardiology propõe uma abordagem semelhante, com início imediato de estatina potente, independentemente do LDL-c basal, seguido por reavaliação em 4 a 6 semanas. Se a meta de LDL-c não for alcançada, adiciona-se ezetimiba e, se necessário, terapia anti-PCSK9.^55^

A estratégia proposta pelo comitê de Acute Cardiovascular Care da ESC – *strike early and strike strong* – defende o início de estatina + ezetimiba já durante a fase aguda da SCA, com rápida adição de terapia anti-PCSK9 em pacientes de muito alto risco. Essa proposta baseia-se em três pilares: (1) risco elevado de eventos cardiovasculares nos primeiros 90 dias; (2) a abordagem escalonada tradicional pode atrasar em até 12 semanas o alcance das metas; e (3) estudos de imagem mostram regressão da aterosclerose e estabilização da placa com uso precoce de alirocumabe e evolocumabe.^332^

Apesar das claras evidências, a implementação na prática clínica permanece subótima. Barreiras como inércia clínica, preocupações infundadas com estatinas, disparidades geográficas, custo elevado das terapias anti-PCSK9 e variações nos sistemas de saúde contribuem para a baixa adesão. Essas falhas são observadas mesmo em países com boa estrutura de saúde pública.

No futuro, o manejo lipídico na SCA pode incluir terapias emergentes, como a inclisirana, que atua via interferência de RNA e oferece potencial para uso precoce e efeito prolongado. Além disso, estratégias para tratar o risco residual – como o controle da hipertrigliceridemia e da Lp(a) elevada após atingir a meta de LDL-c – devem contribuir para intervenções mais completas em prevenção secundária.

Nesta diretriz, optamos por recomendar o início precoce da terapia combinada para o controle lipídico na SCA, com estatina de alta potência associada à ezetimiba já durante a hospitalização. Essa estratégia visa reduzir a inércia terapêutica, aumentar a taxa de atingimento das metas de LDL-c e promover uma redução mais eficaz do risco cardiovascular. A reavaliação lipídica deve ocorrer entre 4 e 6 semanas, momento em que se indica a introdução de terapias anti-PCSK9 caso as metas de LDL-c não tenham sido alcançadas. Em indivíduos com risco cardiovascular extremo, essa abordagem combinada desde o início da internação é especialmente recomendada.^470^

**Table:** 

Recomendação	Força da recomendação	Certeza da evidência
Em pacientes com SCA recomenda-se a favor da dosagem do perfil lipídico precocemente (preferencialmente em até 24 horas do evento agudo) como base para decisões terapêuticas.	FORTE	MODERADA
Em pacientes com SCA, recomenda-se a favor de dosar o perfil lipídico dentro de 4 a 6 semanas após a alta hospitalar.	FORTE	MODERADA
Em pacientes com SCA, recomenda-se a favor de iniciar estatinas potentes nas primeiras 24h após internação por SCA.	FORTE	ALTA
Em pacientes com SCA, recomenda-se a favor de iniciar estatinas potentes associadas à ezetimiba ainda na fase aguda, além de considerar precocemente terapia anti-PCSK9 em indivíduos com risco muito alto ou extremo, como estratégia intensiva voltada à rápida redução dos níveis de LDL-c, minimização da inércia terapêutica e maior probabilidade de alcançar as metas lipídicas recomendadas.	FORTE	MODERADA

SCA: síndrome coronariana aguda; PCSK9: proproteína convertase subtilisina/quexina tipo 9; LDL-c: colesterol da lipoproteína de baixa densidade.

### 9.14. Doenças Imunomediadas

Pacientes com doenças imunomediadas, como artrite reumatoide (AR), lúpus eritematoso sistêmico (LES), espondiloartrites e psoríase, apresentam risco cardiovascular significativamente aumentado,^471^ comparável ao de pacientes com DM tipo 2. A inflamação sistêmica típica dessas enfermidades, além de causar disfunção endotelial e acelerar a aterosclerose, promove modificações qualitativas e quantitativas nas lipoproteínas. Observa-se oxidação do LDL-c e disfunção do HDL-c, tornando-os pró-aterogênicos mesmo quando os níveis séricos estão dentro da normalidade.^472^ Este "paradoxo lipídico" pode mascarar o risco aterosclerótico e retardar o início de medidas preventivas.

Além disso, o uso de medicamentos também pode contribuir para a dislipidemia secundária. Glicocorticoides reduzem o HDL-c e aumentam os TG e o LDL-c.^473^ Imunossupressores, como a ciclosporina, também podem elevar o CT e os TG.

Algumas enfermidades apresentam peculiaridades que contribuem para a dislipidemia e o risco cardiovascular. A psoríase, por exemplo, está associada a maior prevalência de obesidade, hipertensão arterial e diabetes.

Outro aspecto que deve ser considerado é a intensidade da doença e seu controle em longo prazo. Por não considerar o contexto inflamatório, o risco cardiovascular de pacientes com doenças imunomediadas avaliado através de escores tradicionais, como o Framingham, frequentemente é subestimado. Por isso, abordagens ajustadas para essas populações têm sido propostas.^474^

De modo geral, condições como LES, AR ativa e psoríase moderada a grave devem ser consideradas fatores agravantes do risco cardiovascular, mesmo na ausência de outros elementos clássicos de risco. A depender do contexto clínico, pode-se considerar a individualização do risco por meio de exames como a tomografia para escore de cálcio.

O manejo adequado da dislipidemia exige abordagem multifatorial, combinando controle da doença de base, mudanças no estilo de vida e tratamento farmacológico específico. O primeiro pilar do tratamento é o controle da atividade inflamatória, uma vez que melhora o perfil lipídico e reduz o risco CV global.^475^ Agentes como metotrexato e hidroxicloroquina tendem a melhorar o perfil lipídico indiretamente, via controle da inflamação.

O tratamento farmacológico da dislipidemia em pacientes com doenças imunomediadas segue diretrizes específicas, adaptadas ao risco aumentado desses pacientes. As metas terapêuticas geralmente são mais agressivas do que na população geral e dependem também da presença de fatores agravantes como nefropatia, uso prolongado de corticoides ou histórico de eventos cardiovasculares.^55^

As estatinas são a primeira escolha, devido à sua eficácia comprovada na redução de eventos cardiovasculares e por seus efeitos pleiotrópicos anti-inflamatórios.^476^ Em pacientes com intolerância ou resposta inadequada às estatinas, pode-se utilizar ezetimiba ou, em casos de risco muito alto, terapia anti-PCSK9.^477^

Além do tratamento farmacológico, mudanças no estilo de vida são essenciais. Essas intervenções são particularmente importantes, uma vez que pacientes com doenças imunomediadas apresentam maior prevalência de fatores de risco associados, como sedentarismo e resistência insulínica.

**Table:** 

Recomendação	Força da recomendação	Certeza da evidência
Em indivíduos com artrite reumatoide, com critérios de alto risco para a doença imunomediada, recomenda-se a favor de considerar agravante de risco cardiovascular.	FORTE	MODERADA
Em indivíduos com doenças imunomediadas, recomenda-se a favor do controle adequado da atividade inflamatória como estratégia essencial para reduzir o risco cardiovascular.	FORTE	MODERADA
Em indivíduos com doenças imunomediadas, recomenda-se a favor do uso de CAC para estratificação do risco em pacientes com risco intermediário.	CONDICIONAL	ALTA
Em indivíduos com doenças imunomediadas recomenda-se a favor do uso de estatinas como primeira linha no tratamento da dislipidemia.	FORTE	MODERADA
Em indivíduos com doenças imunomediadas: Quando houver intolerância ou resposta inadequada às estatinas, recomenda-se a favor de adicionar ezetimiba ou, se risco muito alto, terapia anti-PCSK9.	FORTE	MODERADA

CAC: escore de cálcio coronariano.

### 9.15. Gestação

Durante a gravidez, as alterações transitórias (2º e 3º trimestres da gestação) do perfil lipídico incluem elevação do CT e LDL-c (30-50%), TG (50-100%), HDL-c (20-40%).^478^

#### 9.15.1. Dislipidemia da Gestação em Mulheres Normolipidêmicas

As dislipidemias transitórias da gestação são as mais prevalentes (20 a 30% das gestantes) e, quando precoces (1º trimestre), elevam as lipoproteínas ricas em TG e HDL-c baixo, aumentando o risco de pré-eclâmpsia, diabetes gestacional, hipertensão arterial sustentada no pós-parto e doença aterosclerótica a longo prazo.^479^ No feto, essas alterações se correlacionam à prematuridade, baixo peso ao nascer e macrossomia.^480^

#### 9.15.2. Dislipidemia da Gestação em Mulheres Dislipidêmicas

HFHe: é a dislipidemia genética mais frequente, caracterizada por LDL-c elevado e risco de DAC precoce.^481^ Na gestação, aumentos adicionais de LDL-c são bem significativos, uma vez que a terapia hipolipemiante é interrompida durante a gravidez e amamentação. Mesmo com elevações pronunciadas do LDL-c, o prognóstico materno-fetal não difere entre as gestantes com e sem HFHe.(4) Não há evidências de que os anos de tratamento descontinuado das gestantes com HFHe aumentem o risco cardiovascular.^482^

Hipercolesterolemia familiar homozigótica é muito mais rara e resulta da herança de duas variantes patogênicas que causam elevações acentuadas do LDL-c, com predisposição à aterosclerose e doença valvar ou supravalvar aórtica precoces. Durante a gravidez, as alterações hormonais e a interrupção da terapia hipolipemiante elevam ainda mais os valores de LDL-c, dificultando o manejo lipídico e seu impacto sobre o risco materno-fetal. Eventos cardíacos maternos durante a gravidez são raros nessas pacientes, e não há estudos prospectivos que avaliem o risco de morbidade ou mortalidade cardiovascular, após interrupção dos hipolipemiantes durante o curto período gestacional.^482^

Hipertrigliceridemias: a hipertrigliceridemia gestacional grave é definida por concentrações de TG plasmáticos > 1.000 mg/dL, causadas por alterações genéticas monogênicas ou poligênicas, ou a presença de fatores secundários (por exemplo, diabetes descompensada). Os riscos maternos incluem pancreatite aguda, síndrome da hiperviscosidade e pré-eclâmpsia.^483^

#### 9.15.3. Lipoproteína(a)

A Lp(a) é um fator de risco para trombose arterial e venosa. Na gestação, pode se elevar entre a 10ª e a 35ª semanas de gravidez, em 20 a 30% das gestantes.^484^ Em concentrações elevadas, a Lp(a) está associada a um risco aumentado de complicações maternas, como a pré-eclâmpsia, e neonatais, como o parto prematuro. Uma das justificativas para esse risco é sua semelhança estrutural com o plasminogênio, que pode favorecer mecanismos pró-trombóticos e inflamatórios contribuindo para esses desfechos.^484^

#### 9.15.4. Tratamento Medicamentoso

##### 9.15.4.1. Estatinas

Segurança: em série de relatos de casos, foi descrita elevada incidência de más formações estruturais, incluindo o sistema nervoso e esquelético, em bebês expostos a estatinas lipofílicas durante o primeiro trimestre de gestação.^485^

O FDA atribuiu a classificação X para estatinas, proibindo seu uso. Recomendava-se que as gestantes interrompessem seu uso enquanto tentassem engravidar, durante a gravidez e durante a amamentação.^486^

##### 9.15.4.2. Novas Evidências

Estudos observacionais, revisões sistemáticas e metanálises não demonstraram aumentos nas taxas de malformações congênitas ou outros danos em mulheres expostas a estatinas no período gestacional. Revisões sistemáticas e metanálise em mulheres com hiperlipidemia ou com comorbidades e risco de pré-eclâmpsia não demonstraram aumento nas taxas de malformações congênitas ou outros danos em gestantes expostas a estatinas.^487 - 490^

Na hipercolesterolemia familiar homozigótica, estudo retrospectivo comparou gestantes que mantiveram o uso de estatinas na gravidez com aquelas que interromperam seu uso. Não se observaram diferenças nos desfechos gerais da gestação, complicações cardiovasculares ou presença de malformação congênita entre os dois grupos.^491^

Novo posicionamento do FDA: nas gestantes que apresentam colesterol e risco cardiovascular significativamente elevados, o FDA argumenta que "os benefícios das estatinas podem incluir a prevenção de eventos cardiovasculares graves ou potencialmente fatais em um pequeno grupo de pacientes de risco muito alto".^486^

Recomendação: a manutenção das estatinas durante a gestação deve ser indicada apenas para pacientes de muito alto risco e/ou portadoras de HF. Essa decisão deve ser compartilhada entre médico e paciente, considerando a dificuldade de se avaliar os riscos maternos em se suspendendo a medicação e os potenciais riscos fetais em se mantendo a medicação, enfatizando que dados de segurança das estatinas na gravidez evoluíram, mas ainda são extremamente escassos

Se mantida, as estatinas devem ser interrompidas no primeiro trimestre da gestação e reintroduzidas no terceiro trimestre. A estatina com maior evidência de segurança é a pravastatina.

##### 9.15.4.3. Resinas ou Sequestradoras de Sais Biliares

As resinas de troca podem ser utilizadas na gravidez e não se associam a maior risco de anormalidades congênitas. Tendem a ser mal toleradas e afetar a absorção de vitaminas lipossolúveis.^492^ Há relatos de hematomas subdurais em fetos, decorrentes de deficiência de vitamina K em mães em uso crônico de colestiramina por colestase intra-hepática.^492^

##### 9.15.4.4. Aférese de Lipoproteínas

A remoção extracorpórea de lipoproteínas contendo ApoB por aférese, se disponível, é o tratamento preferencial para HF homozigótica.^493^

##### 9.15.4.5. Ezetimiba

Não recomendado na gestação e amamentação.^494^

Novos medicamentos: terapias anti-PCSK9 (evolocumabe, alirocumabe, inclisiran), inibidores da ANGPTL3 (evinacumabe), ácido bempedoico e lomitapida não são recomendados na gestação e amamentação.

Fibratos não recomendados na gestação;^494^ porém, existem poucos relatos de casos, principalmente a partir do segundo semestre de gestação. Recomenda-se considerar fenofibrato no 2º trimestre da gestação se TG > 880 mg/dL.

##### 9.15.4.6. Ômega-3

Recomendação: os ácidos graxos ômega-3 podem ser uma opção eficaz para pacientes com hipertrigliceridemia grave, especialmente aqueles com risco de pancreatite (TG > 880 mg/dL).^494^

**Table:** 

Recomendação	Força da recomendação	Certeza da evidência
Para gestantes com dislipidemia relacionadas à gestação ou outras formas de dislipidemia primária ou secundária, recomenda-se a favor de seguir dieta com baixo teor de gordura, alto teor de fibras solúveis e carboidratos de baixo índice glicêmico.	FORTE	MODERADA
Para mulheres em planejamento de engravidar, em uso prévio de estatina, recomenda-se a favor de interromper a estatina 60 dias antes da concepção.	FORTE	MODERADA
Para gestantes em uso de estatina, recomenda-se a favor de suspensão imediata do medicamento, devendo o mesmo ser reiniciado após o período de amamentação.	FORTE	MODERADA
Para gestantes de muito alto risco, recomenda-se a favor de individualização terapêutica e decisão compartilhada sobre o reinício da estatina no 3º trimestre da gestação.	CONDICIONAL	MODERADA
Para população gestante com hipercolesterolemia, recomenda-se a favor do uso de resinas de troca.	CONDICIONAL	BAIXA
Para gestantes e lactantes, recomenda-se contra uso da ezetimiba, terapias anti-PCSK9, inibidores da ANGPTL3 (como evinacumabe), ácido bempedoico e lomitapida.	FORTE	MODERADA
Para gestante com triglicérides acima de 880 mg/dL a despeito de modificação de estilo de vida, recomenda-se a favor do uso de fenofibrato no 2º trimestre da gestação.	CONDICIONAL	BAIXA
Para gestante com triglicérides acima de 880 mg/dL a despeito de modificação de estilo de vida, recomenda-se a favor do uso de ácidos graxos ômega-3.	CONDICIONAL	BAIXA

PCSK9: proproteína convertase subtilisina/quexina tipo 9.

### 9.16. Mulher

As mulheres compartilham diversos fatores de risco tradicionais com os homens; no entanto, esses fatores podem ter impacto distinto nas mulheres, devido a diferenças biológicas e socioculturais. Além disso, há fatores de risco específicos do sexo feminino, frequentemente sub-reconhecidos na prática clínica,^14 , 495 , 496^ que devem ser considerados fatores agravantes de risco,^497^ conforme detalhado no capítulo de estratificação de risco.

Ao longo da vida, o perfil lipídico das mulheres apresenta padrões distintos, influenciados por fatores hormonais e fisiológicos. Durante a fase reprodutiva jovem, variações do perfil lipídico são observadas ao longo do ciclo menstrual, com LDL-c atingindo pico na fase folicular e menor na lútea; o uso de anticoncepcionais orais pode elevar o CT e TG. Na gravidez, há elevação fisiológica significativa de TG (até 2 vezes) e CT (∼ 1,5 vez). Após a menopausa, a queda estrogênica promove aumento de LDL-c e TG, elevando o risco aterosclerótico.

A terapia hormonal oral na menopausa reduz o LDL-c e aumenta o HDL-c, mas pode elevar os TG, especialmente com preparações com estrogênios conjugados equinos ou 17β-estradiol associados a progestagênios, como acetato de medroxiprogesterona. A via transdérmica tem efeito neutro nos TG e menor impacto hepático, preservando o efeito sobre o HDL-c.

Considerando que as mulheres apresentam fatores de risco específicos relacionados a alterações hormonais e a fases como gestação e menopausa, a identificação e a consideração de agravantes de risco são fortemente recomendadas por esta diretriz e fundamentais para uma estratificação de risco personalizada. Fatores como dislipidemia persistente, histórico familiar precoce e aterosclerose subclínica devem orientar a adoção de metas terapêuticas efetivas, sem distinção de abordagem entre homens e mulheres. Independentemente do sexo, o tratamento intensivo deve ser indicado conforme a categoria de risco, visando à redução do LDL-c até os níveis preconizados pelas diretrizes, com uso de estatinas potentes, terapia combinada com ezetimiba e, quando necessário, terapias anti-PCSK9. Essa equidade na condução terapêutica é essencial para superar a inércia clínica e as barreiras históricas de subtratamento, ampliando de forma efetiva a proteção cardiovascular na população feminina.

**Table:** 

Recomendação	Força da recomendação	Certeza da evidência
Para mulheres classificadas como de risco cardiovascular baixo ou intermediário, recomenda-se a favor do uso clínico de agravantes de risco, com o objetivo de refinar a estratificação de risco e orientar decisões terapêuticas mais intensivas.	FORTE	MODERADA
Para mulheres classificadas como de alto risco, muito alto risco ou risco extremo, recomenda-se a favor da adoção de terapia intensiva e combinada.	FORTE	ALTA

## 10. Conclusão

A Diretriz Brasileira de Dislipidemias e Prevenção da Aterosclerose de 2025 representa um avanço decisivo rumo à redução efetiva do risco cardiovascular em todas as fases da vida. Com base em sólida evidência científica, o documento extrapola a abordagem tradicional, focada exclusivamente na dislipidemia e incorpora uma visão mais ampla da prevenção da DCVA, integrando estratégias de implementação, ferramentas de imagem e biomarcadores inovadores.

**Figura 10.1:**
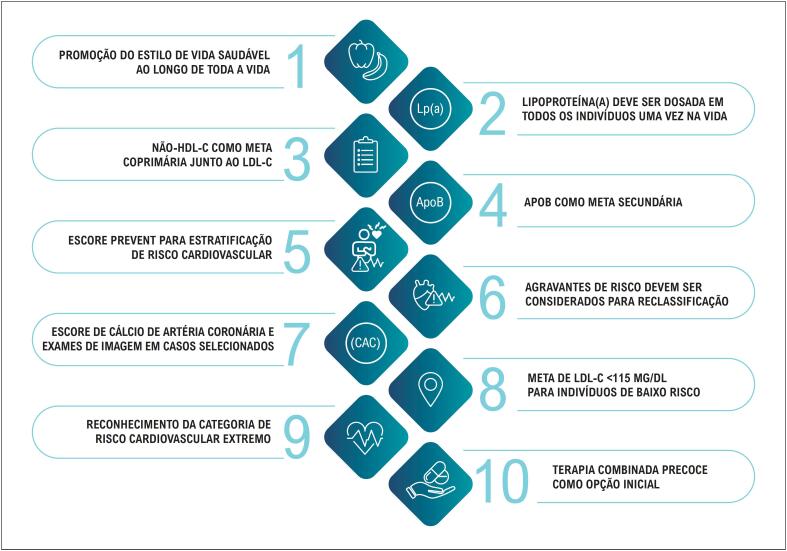
Mensagens-chave da Diretriz.

As 10 mensagens-chave ( Figura 10.1 ) desta diretriz consolidam os pilares da prevenção moderna: desde a promoção do estilo de vida saudável e a avaliação de risco com novos marcadores, como Lp(a) e ApoB, até o reconhecimento de perfis de risco específicos, incluindo agravantes de risco e a categoria de risco cardiovascular extremo e incorporando metas terapêuticas mais agressivas e individualizadas. A redução da meta de LDL-c em indivíduos com baixo risco a curto prazo reconhece a importância do controle precoce e sustentado dos níveis de LDL-c. A inclusão de ferramentas como o escore PREVENT e o CAC aprimora a capacidade de reclassificação e personalização do cuidado.

Neste novo cenário, a terapia hipolipemiante evolui para um arsenal mais robusto e estratégico, com ênfase na redução de risco cardiovascular, e não apenas na correção laboratorial da lipidemia. A recomendação de combinação precoce de estatina, ezetimiba, anti-PCSK9 e, em casos selecionados, ácido bempedoico reflete a urgência de intervenções intensivas e seguras, que aumentem a eficácia terapêutica, sobretudo em contextos de baixa adesão, intolerância a estatinas e dificuldade em atingir metas lipídicas.

Ao reconhecer a aterosclerose como uma condição progressiva e silenciosa, iniciada precocemente e influenciada por múltiplos fatores – sociais, genéticos e clínicos – esta diretriz reforça o compromisso com uma abordagem contínua e integrada ao longo do curso de vida. Mais do que um documento técnico, propõe-se como instrumento de transformação da prática clínica brasileira, promovendo equidade, atualização científica e impacto direto na saúde populacional.

Por fim, a Diretriz Brasileira de Dislipidemias e Prevenção da Aterosclerose reconecta o tratamento à sua verdadeira finalidade: prevenir eventos cardiovasculares, salvar vidas e oferecer uma vida melhor e mais longa às próximas gerações.
