# Supplementary material for: Diretriz Brasileira de Dislipidemias e Prevenção da Aterosclerose – 2025
Source: Arq Bras Cardiol. 2025 Oct 13;122(9):e20250640. [Article in Portuguese] doi: 10.36660/abc.20250640 (PMC12674852; doi:10.36660/abc.20250640)
Supplement: Translation

# Translation: Brazilian Guideline on Dyslipidemias and Prevention of Atherosclerosis – 2025

**DOI:** 10.36660/abc.20250640i

**Authors:** Fabiana Hanna Rached, Marcio Hiroshi Miname, Viviane Zorzanelli Rocha, André Zimerman, Fernando Henpin Yue Cesena, Andrei Carvalho Sposito, Raul Dias dos Santos, Paulo Eduardo Ballvé Behr, Henrique Tria Bianco, Renato Jorge Alves, André Arpad Faludi, Elaine dos Reis Coutinho, Francisco Antonio Helfenstein Fonseca, Luiz Sérgio Fernandes de Carvalho, Adriana Bertolami, Aloísio Marchi da Rocha, Ana Paula Marte, Antonio Carlos Palandri Chagas, Bruno Caramelli, Carisi Anne Polanczyk, Carlos Eduardo dos Santos Ferreira, Carlos Vicente Serrano, Daniel Branco de Araujo, Emilio Hideyuki Moriguchi, Fausto J. Pinto, Humberto Graner Moreira, Isabela de Carlos Back, Jose Rocha Faria, Kleisson Antônio Pontes Maia, Marcelo Chiara Bertolami, Marcelo Heitor Vieira Assad, Maria Cristina de Oliveira Izar, Mauricio Alves Barreto, Natasha Slhessarenko Fraife Barreto, Pedro Gabriel Melo de Barros e Silva, Pedro Pimentel, Raul Cavalcante Maranhão, Sergio Emanuel Kaiser, Valeria Arruda Machado, Jose Francisco Kerr Saraiva

**Affiliations:** 1 São Paulo SP Brazil Instituto do Coração (Incor) do Hospital das Clínicas da Faculdade de Medicina da Universidade de São Paulo (HCFMUSP), São Paulo, SP – Brazil; 2 São Paulo SP Brasil Hospital Israelita Albert Einstein, São Paulo, SP – Brasil; 3 Porto Alegre RS Brazil Hospital Moinhos de Vento, Porto Alegre, RS – Brazil; 4 Porto Alegre RS Brazil Universidade Federal do Rio Grande do Sul, Porto Alegre, RS – Brazil; 5 São Paulo SP Brazil Instituto Dante Pazzanese de Cardiologia, São Paulo, SP – Brazil; 6 Campinas SP Brazil Universidade Estadual de Campinas (UNICAMP), Campinas, SP – Brazil; 7 São Paulo SP Brazil Universidade de São Paulo (USP), São Paulo, SP – Brazil; 8 Porto Alegre RS Brazil Santa Casa de Porto Alegre, Porto Alegre, RS – Brazil; 9 São Paulo SP Brazil Universidade Federal de São Paulo (UNIFESP), São Paulo, SP – Brazil; 10 São Paulo SP Brazil Irmandade da Santa Casa de Misericórdia de São Paulo (ISCMSP), São Paulo, SP – Brazil; 11 São Paulo SP Brazil Faculdade de Ciências Médicas da Santa Casa de São Paulo (FCMSCSP), São Paulo, SP – Brazil; 12 Campinas SP Brazil Pontifícia Universidade Católica de Campinas, Campinas, SP – Brazil; 13 Jundiaí SP Brazil Clarity Healthcare Desenvolvimento de Software LTDA, Jundiaí, SP – Brazil; 14 Santo André SP Brazil Centro Universitário Faculdade de Medicina do ABC, Santo André, SP – Brazil; 15 Porto Alegre RS Brazil Hospital de Clínicas de Porto Alegre, Porto Alegre, RS – Brazil; 16 Lisboa Portugal Faculdade de Medicina da Universidade de Lisboa, Lisboa – Portugal; 17 Goiânia GO Brazil Universidade Federal de Goiás (UFG), Goiânia, GO – Brazil; 18 Florianópolis SC Brazil Universidade Federal de Santa Catarina, Florianópolis, SC – Brazil; 19 Curitiba PR Brazil Pontifícia Universidade Católica do Paraná, Curitiba, PR – Brazil; 20 Belo Horizonte MG Brazil Faculdade Ciências Médicas de Minas Gerais, Belo Horizonte, MG – Brazil; 21 Rio de Janeiro RJ Brazil Instituto Nacional de Cardiologia (INC), Rio de Janeiro, RJ – Brazil; 22 Salvador BA Brazil Escola Bahiana de Medicina e Saúde Pública, Salvador, BA – Brazil; 23 Salvador BA Brazil Hospital Ana Nery, Universidade Federal da Bahia (UFBA), Salvador, BA – Brazil; 24 Salvador BA Brazil Hospital Fundação Bahiana de Cardiologia e Combate ao Câncer, Salvador, BA – Brazil; 25 Cuiabá MT Brazil Diagnósticos da América (DASA), Cuiabá, MT – Brazil; 26 Cuiabá MT Brazil Universidade Federal de Mato Grosso (UFMT), Cuiabá, MT – Brazil; 27 São Paulo SP Brazil Hospital do Coração (Hcor), São Paulo, SP – Brazil; 28 São Paulo SP Brazil Centro Universitário São Camilo, São Paulo, SP – Brazil; 29 São Paulo SP Brazil Brazilian Clinical Research Institute, São Paulo, SP – Brazil; 30 Porto Alegre RS Brazil Hospital Nossa Senhora Conceição de Porto Alegre, Porto Alegre, RS – Brazil; 31 Rio de Janeiro RJ Brazil Universidade do Estado do Rio de Janeiro (UERJ), Rio de Janeiro, RJ – Brazil

**Table:** 

Brazilian Guideline on Dyslipidemias and Prevention of Atherosclerosis – 2025	
The report below lists declarations of interest as reported to the SBC by the experts during the period of the development of these statement, 2024/2025.	
Expert	Type of relationship with industry
Adriana Bertolami	Financial declaration A - Economically relevant payments of any kind made to (i) you, (ii) your spouse/partner or any other person living with you, (iii) any legal person in which any of these is either a direct or indirect controlling owner, business partner, shareholder or participant; any payments received for lectures, lessons, training instruction, compensation, fees paid for participation in advisory boards, investigative boards or other committees, etc. from the brazilian or international pharmaceutical, orthosis, prosthesis, equipment and implants industry: - Daiichi Sankyo.
Aloísio Marchi da Rocha	Financial declaration A - Economically relevant payments of any kind made to (i) you, (ii) your spouse/partner or any other person living with you, (iii) any legal person in which any of these is either a direct or indirect controlling owner, business partner, shareholder or participant; any payments received for lectures, lessons, training instruction, compensation, fees paid for participation in advisory boards, investigative boards or other committees, etc. from the brazilian or international pharmaceutical, orthosis, prosthesis, equipment and implants industry: - Amgen: Repatha; AstraZeneca: Forxiga; Bayer: Xarelto, FiriHIGH; BMS: Camzyos; Boehringer Ingelheim: Jardiance; Daiichi Sankyo: Nustendi; GSK: Shingrix; Novartis: Entresto, Sybrava; NovoNordisk: Ozempic; Pfizer: Tafamidis, Amiloidose; Servier: Vastarel, Triplixam; Viatris: Inspra. B - Research funding under your direct/personal responsibility (directed to the department or institution) from the brazilian or international pharmaceutical, orthosis, prosthesis, equipment and implants industry: - Astrazeneca: Baxduo; Bayer: Finerinona; Lilly: Tirzepatida; Novartis: Entresto; Novo Nordisk: Ziltivequimabe. Other relationships Funding of continuing medical education activities, including travel, accommodation and registration in conferences and courses, from the brazilian or international pharmaceutical, orthosis, prosthesis, equipment and implants industry: - Lilly; AstraZeneca; NovoNordisk; Servier.
Ana Paula Marte	Financial declaration A - Economically relevant payments of any kind made to (i) you, (ii) your spouse/partner or any other person living with you, (iii) any legal person in which any of these is either a direct or indirect controlling owner, business partner, shareholder or participant; any payments received for lectures, lessons, training instruction, compensation, fees paid for participation in advisory boards, investigative boards or other committees, etc. from the brazilian or international pharmaceutical, orthosis, prosthesis, equipment and implants industry: - Zerbini Foundation: InCor Teaching; Ultragenyx: Evinacumab; PTC therapeutics: Volanesorsen.
André Arpad Faludi	Nothing to be declared
André Zimerman	Financial declaration A - Economically relevant payments of any kind made to (i) you, (ii) your spouse/partner or any other person living with you, (iii) any legal person in which any of these is either a direct or indirect controlling owner, business partner, shareholder or participant; any payments received for lectures, lessons, training instruction, compensation, fees paid for participation in advisory boards, investigative boards or other committees, etc. from the brazilian or international pharmaceutical, orthosis, prosthesis, equipment and implants industry: - Novartis: Sybrava.
Andrei C. Sposito	Financial declaration A - Economically relevant payments of any kind made to (i) you, (ii) your spouse/partner or any other person living with you, (iii) any legal person in which any of these is either a direct or indirect controlling owner, business partner, shareholder or participant; any payments received for lectures, lessons, training instruction, compensation, fees paid for participation in advisory boards, investigative boards or other committees, etc. from the brazilian or international pharmaceutical, orthosis, prosthesis, equipment and implants industry: - Lilly, Novo Nordisk, Daiichi Sankyo. B - Research funding under your direct/personal responsibility (directed to the department or institution) from the brazilian or international pharmaceutical, orthosis, prosthesis, equipment and implants industry: - AstraZeneca. Other relationships Funding of continuing medical education activities, including travel, accommodation and registration in conferences and courses, from the brazilian or international pharmaceutical, orthosis, prosthesis, equipment and implants industry: - Daiichi, Novo Nordisk.
Antonio Carlos Palandri Chagas	Financial declaration A - Economically relevant payments of any kind made to (i) you, (ii) your spouse/partner or any other person living with you, (iii) any legal person in which any of these is either a direct or indirect controlling owner, business partner, shareholder or participant; any payments received for lectures, lessons, training instruction, compensation, fees paid for participation in advisory boards, investigative boards or other committees, etc. from the brazilian or international pharmaceutical, orthosis, prosthesis, equipment and implants industry: - Institute of Vita.
Bruno Caramelli	Nothing to be declared
Carisi Anne Polanczyk	Financial declaration A - Economically relevant payments of any kind made to (i) you, (ii) your spouse/partner or any other person living with you, (iii) any legal person in which any of these is either a direct or indirect controlling owner, business partner, shareholder or participant; any payments received for lectures, lessons, training instruction, compensation, fees paid for participation in advisory boards, investigative boards or other committees, etc. from the brazilian or international pharmaceutical, orthosis, prosthesis, equipment and implants industry: - AstraZeneca; Amgen; Abbott: HeartMate; Bayer: Xarelto, Lipidil; Baxter; Pfizer; BMS; Roche. Novartis: Inclisiran; Organon; Sanofi. B - Research funding under your direct/personal responsibility (directed to the department or institution) from the brazilian or international pharmaceutical, orthosis, prosthesis, equipment and implants industry: - AstraZeneca, Amgen, Abbott, Bayer, Novartis, Sanofi, Roche: research not related to specific products. C - Personal research funding paid by the brazilian or international pharmaceutical, orthosis, prosthesis, equipment and implants industry: - Bayer: Xarelto, Lipidil; Pfizer; BMS; Roche; Organon; Sanofi. No specific area. Other relationships Funding of continuing medical education activities, including travel, accommodation and registration in conferences and courses, from the brazilian or international pharmaceutical, orthosis, prosthesis, equipment and implants industry: - AstraZeneca; Amgen; Bayer: Lipidil; Pfizer; BMS; Sanofi. Courses without specific products. Any economically relevant equity interest in companies in the healthcare or education industry or in any companies competing with or supplying to SBC: - Health consultancy area: PEV.
Carlos Eduardo dos Santos Ferreira	Nothing to be declared
Carlos Vicente Serrano Junior	Nothing to be declared
Daniel Branco de Araujo	Financial declaration A - Economically relevant payments of any kind made to (i) you, (ii) your spouse/partner or any other person living with you, (iii) any legal person in which any of these is either a direct or indirect controlling owner, business partner, shareholder or participant; any payments received for lectures, lessons, training instruction, compensation, fees paid for participation in advisory boards, investigative boards or other committees, etc. from the brazilian or international pharmaceutical, orthosis, prosthesis, equipment and implants industry: - Novo Nordisk: Wegovy; Sanofi: Zinpass Eze; Daiichi Sankyo: Nustendi; AstraZeneca: Forxiga; EMS: Linadib; Boehringer: Jardiance. B - Research funding under your direct/personal responsibility (directed to the department or institution) from the brazilian or international pharmaceutical, orthosis, prosthesis, equipment and implants industry: - MSD: MK0616. Other relationships Funding of continuing medical education activities, including travel, accommodation and registration in conferences and courses, from the brazilian or international pharmaceutical, orthosis, prosthesis, equipment and implants industry: - Sanofi: Zinpass Eze.
Elaine dos Reis Coutinho	Financial declaration A - Economically relevant payments of any kind made to (i) you, (ii) your spouse/partner or any other person living with you, (iii) any legal person in which any of these is either a direct or indirect controlling owner, business partner, shareholder or participant; any payments received for lectures, lessons, training instruction, compensation, fees paid for participation in advisory boards, investigative boards or other committees, etc. from the brazilian or international pharmaceutical, orthosis, prosthesis, equipment and implants industry: - Novartis: Sybrava; Biolab: Repatha; Daiichi Sankyo: Nustendi. Other relationships Funding of continuing medical education activities, including travel, accommodation and registration in conferences and courses, from the brazilian or international pharmaceutical, orthosis, prosthesis, equipment and implants industry: - Novartis: Sybrava; Biolab: Repatha; Daiichi Sankyo: Nustendi. Employment relationship with the brazilian or international pharmaceutical, orthosis, prosthesis, equipment and implants industry, as well as any employment relationship with health insurance companies or medical audit companies (including part-time jobs) in the year to which your declaration refers: - Unimed Campinas.
Emilio Hideyuki Moriguchi	Financial declaration A - Economically relevant payments of any kind made to (i) you, (ii) your spouse/partner or any other person living with you, (iii) any legal person in which any of these is either a direct or indirect controlling owner, business partner, shareholder or participant; any payments received for lectures, lessons, training instruction, compensation, fees paid for participation in advisory boards, investigative boards or other committees, etc. from the brazilian or international pharmaceutical, orthosis, prosthesis, equipment and implants industry: - Biolab: Repatha; Biolab: Livalo; Novartis: Sybrava. B - Research funding under your direct/personal responsibility (directed to the department or institution) from the brazilian or international pharmaceutical, orthosis, prosthesis, equipment and implants industry: - Human Life CORD Japan inc.: Sarcopenia. Other relationships Funding of continuing medical education activities, including travel, accommodation and registration in conferences and courses, from the brazilian or international pharmaceutical, orthosis, prosthesis, equipment and implants industry: - Biolab: Repatha; Biolab: Livalo; Novartis Sybrava.
Fabiana Hanna Rached	Financial declaration A - Economically relevant payments of any kind made to (i) you, (ii) your spouse/partner or any other person living with you, (iii) any legal person in which any of these is either a direct or indirect controlling owner, business partner, shareholder or participant; any payments received for lectures, lessons, training instruction, compensation, fees paid for participation in advisory boards, investigative boards or other committees, etc. from the brazilian or international pharmaceutical, orthosis, prosthesis, equipment and implants industry: - Novo Nordisk: semaglutide; Novartis: inclisiran; Daiichi Sankyo: bempedoic acid. Other relationships Funding of continuing medical education activities, including travel, accommodation and registration in conferences and courses, from the brazilian or international pharmaceutical, orthosis, prosthesis, equipment and implants industry: - Novo Nordisk: semaglutide; Novartis: inclisiran; Daiichi Sankyo: bempedoic acid.
Fausto J. Pinto	Financial declaration A - Economically relevant payments of any kind made to (i) you, (ii) your spouse/partner or any other person living with you, (iii) any legal person in which any of these is either a direct or indirect controlling owner, business partner, shareholder or participant; any payments received for lectures, lessons, training instruction, compensation, fees paid for participation in advisory boards, investigative boards or other committees, etc. from the brazilian or international pharmaceutical, orthosis, prosthesis, equipment and implants industry: - Boehringer Ingelheim; Daichi Sankyo; Novartis; Servier; CSL Vifor; Zydus. B - Research funding under your direct/personal responsibility (directed to the department or institution) from the brazilian or international pharmaceutical, orthosis, prosthesis, equipment and implants industry: - Abbott; Biosensors; Medtronic; Novartis; Pfizer. C - Personal research funding paid by the brazilian or international pharmaceutical, orthosis, prosthesis, equipment and implants industry: - Boehringer Ingelheim; Daiichi Sankyo; Medtronic; Novartis; Novo Nordisk; Servier; CSL Vifor.
Fernando Henpin Yue Cesena	Nothing to be declared
Francisco Antonio Helfenstein Fonseca	Financial declaration A - Economically relevant payments of any kind made to (i) you, (ii) your spouse/partner or any other person living with you, (iii) any legal person in which any of these is either a direct or indirect controlling owner, business partner, shareholder or participant; any payments received for lectures, lessons, training instruction, compensation, fees paid for participation in advisory boards, investigative boards or other committees, etc. from the brazilian or international pharmaceutical, orthosis, prosthesis, equipment and implants industry: - Libbs: Artag, antiplatelet. B - Research funding under your direct/personal responsibility (directed to the department or institution) from the brazilian or international pharmaceutical, orthosis, prosthesis, equipment and implants industry: - Libbs: Plenance, dyslipidemia. C - Personal research funding paid by the brazilian or international pharmaceutical, orthosis, prosthesis, equipment and implants industry: - Hypera: Addera, vitamin D. Other relationships Funding of continuing medical education activities, including travel, accommodation and registration in conferences and courses, from the brazilian or international pharmaceutical, orthosis, prosthesis, equipment and implants industry: - NovoNordisk: Wegovy, GLP-1 Analog.
Henrique Tria Bianco	Other relationships Funding of continuing medical education activities, including travel, accommodation and registration in conferences and courses, from the brazilian or international pharmaceutical, orthosis, prosthesis, equipment and implants industry: - Novo Nordisk: semaglutide, diabetes.
Humberto Graner Moreira	Financial declaration A - Economically relevant payments of any kind made to (i) you, (ii) your spouse/partner or any other person living with you, (iii) any legal person in which any of these is either a direct or indirect controlling owner, business partner, shareholder or participant; any payments received for lectures, lessons, training instruction, compensation, fees paid for participation in advisory boards, investigative boards or other committees, etc. from the brazilian or international pharmaceutical, orthosis, prosthesis, equipment and implants industry: - Pfizer: amyloidosis and immunizations; Novo Nordisk: obesity and inflammation; Novartis: dyslipidemia; Daichii-Sankyo: dyslipidemia; Bayer: cardio-oncology. Other relationships Funding of continuing medical education activities, including travel, accommodation and registration in conferences and courses, from the brazilian or international pharmaceutical, orthosis, prosthesis, equipment and implants industry: - Novo Nordisk: obesity.
Isabela de Carlos Back	Other relationships Funding of continuing medical education activities, including travel, accommodation and registration in conferences and courses, from the brazilian or international pharmaceutical, orthosis, prosthesis, equipment and implants industry: - Ultragenyx: Evckeeza.
José Francisco Kerr Saraiva	Financial declaration C - Personal research funding paid by the brazilian or international pharmaceutical, orthosis, prosthesis, equipment and implants industry: - Bayer: finerinone; Novo Nordisk: semaglutide; AstraZeneca: Zirconium cyclosilicate, dapagliflozin; Amgen: evolocumab; Boehringer Ingelheimer: empagliflozin; Lilly: tirzepatide, atorvastatin viatris; Daichii Sankyo: bempedoic acid/Edoxaban; Mantecorp: rosuvastatin. Other relationships Funding of continuing medical education activities, including travel, accommodation and registration in conferences and courses, from the brazilian or international pharmaceutical, orthosis, prosthesis, equipment and implants industry: - Bayer: finerinone; Novo Nordisk: Semaglutide; AstraZeneca: Zirconium cyclosilicate, dapagliflozin; Amgen: evolocumab; Boehringer Ingelheimer: empagliflozin; Lilly: tirzepatide, atorvastatin viatris; Daichii Sankyo: bempedoic acid/edoxaban
Jose Rocha Faria Neto	Financial declaration A - Economically relevant payments of any kind made to (i) you, (ii) your spouse/partner or any other person living with you, (iii) any legal person in which any of these is either a direct or indirect controlling owner, business partner, shareholder or participant; any payments received for lectures, lessons, training instruction, compensation, fees paid for participation in advisory boards, investigative boards or other committees, etc. from the brazilian or international pharmaceutical, orthosis, prosthesis, equipment and implants industry: - Aché: CAD and dyslipidemia; Daiichi Sankyo: CAD and dyslipidemia; Libbs: CAD and dyslipidemia; Novartis: dyslipidemia; AstraZeneca: diabetes; Lilly: diabetes and obesity; Novo Nordisk: diabetes and obesity; Sanofi and Medley: dyslipidemia; Bayer: cardiovascular risk. Other relationships Funding of continuing medical education activities, including travel, accommodation and registration in conferences and courses, from the brazilian or international pharmaceutical, orthosis, prosthesis, equipment and implants industry: - Novo Nordisk; Bayer; Daiichi Sankyo; AstraZeneca.
Kleisson Antônio Pontes Maia	Financial declaration A - Economically relevant payments of any kind made to (i) you, (ii) your spouse/partner or any other person living with you, (iii) any legal person in which any of these is either a direct or indirect controlling owner, business partner, shareholder or participant; any payments received for lectures, lessons, training instruction, compensation, fees paid for participation in advisory boards, investigative boards or other committees, etc. from the brazilian or international pharmaceutical, orthosis, prosthesis, equipment and implants industry: - Novartis: hypercholesterolemia; GSK: vaccines; Biolab: hypertension; Lilly: diabetes, obesity; Novo Nordisk: diabetes, obesity; Servier: coronary heart disease. B - Research funding under your direct/personal responsibility (directed to the department or institution) from the brazilian or international pharmaceutical, orthosis, prosthesis, equipment and implants industry: - Lilly: Lp(a). Other relationships Funding of continuing medical education activities, including travel, accommodation and registration in conferences and courses, from the brazilian or international pharmaceutical, orthosis, prosthesis, equipment and implants industry: - Servier: coronary heart disease; Lilly: diabetes; Novo Nordisk: diabetes, obesity; Viatris: hypercholesterolemia.
Luiz Sérgio Fernandes de Carvalho	Nothing to be declared
Marcelo Chiara Bertolami	Financial declaration A - Economically relevant payments of any kind made to (i) you, (ii) your spouse/partner or any other person living with you, (iii) any legal person in which any of these is either a direct or indirect controlling owner, business partner, shareholder or participant; any payments received for lectures, lessons, training instruction, compensation, fees paid for participation in advisory boards, investigative boards or other committees, etc. from the brazilian or international pharmaceutical, orthosis, prosthesis, equipment and implants industry: - Abbott: Lipidil.
Marcelo Heitor Vieira Assad	Financial declaration A - Economically relevant payments of any kind made to (i) you, (ii) your spouse/partner or any other person living with you, (iii) any legal person in which any of these is either a direct or indirect controlling owner, business partner, shareholder or participant; any payments received for lectures, lessons, training instruction, compensation, fees paid for participation in advisory boards, investigative boards or other committees, etc. from the brazilian or international pharmaceutical, orthosis, prosthesis, equipment and implants industry: - AstraZeneca: Forxiga; BAYER: FiriHIGH; Biolab: Repath; Boerhringer Ingelheim: Glyxambi; Daiichy Sankyo: Benicar, Nustendi; EMS: Bramicar; GSK: Shingrix; Libbs: Stanglit; Lilly: Mounjaro; Novo Nordisk: Wegovy; Novartis: Sybrava; Pfizer: Prevenar 20; Viatris: Lipitor, Inspra. B - Research funding under your direct/personal responsibility (directed to the department or institution) from the brazilian or international pharmaceutical, orthosis, prosthesis, equipment and implants industry: - AMGEN: Olpasirana. Other relationships Funding of continuing medical education activities, including travel, accommodation and registration in conferences and courses, from the brazilian or international pharmaceutical, orthosis, prosthesis, equipment and implants industry: - Bayer: FiriHIGH; Daiichi Sankyo: Benicar; Novo Nordisk: Wegovy.
Marcio Hiroshi Miname	Financial declaration A - Economically relevant payments of any kind made to (i) you, (ii) your spouse/partner or any other person living with you, (iii) any legal person in which any of these is either a direct or indirect controlling owner, business partner, shareholder or participant; any payments received for lectures, lessons, training instruction, compensation, fees paid for participation in advisory boards, investigative boards or other committees, etc. from the brazilian or international pharmaceutical, orthosis, prosthesis, equipment and implants industry: - Novartis: dyslipidemia; Find: dyslipidemia; Libbs: dyslipidemia B - Research funding under your direct/personal responsibility (directed to the department or institution) from the brazilian or international pharmaceutical, orthosis, prosthesis, equipment and implants industry: - Kowa: Pemafibrato.
Maria Cristina de Oliveira Izar	Financial declaration A - Economically relevant payments of any kind made to (i) you, (ii) your spouse/partner or any other person living with you, (iii) any legal person in which any of these is either a direct or indirect controlling owner, business partner, shareholder or participant; any payments received for lectures, lessons, training instruction, compensation, fees paid for participation in advisory boards, investigative boards or other committees, etc. from the brazilian or international pharmaceutical, orthosis, prosthesis, equipment and implants industry: - Amgen: Repatha; Amryt Pharma: Lojuxta; AstraZeneca: Dapagliflozina; Aché: Trezor, Trezete; Biolab: Livalo, Posicor, Repatha; Abbott: Lipidil; EMS: Rosuvastatina; Eurofarma: Rosuvastatina; Sanofi: Praluent, Zympass, Zympass Eze, Efluelda; Libbs: Plenance, Plenance Eze; NovoNordisk: Ozempic; Servier: Acertamlo, Acertalix; PTCBio: Waylivra; Ultragenyx: Evkeeza; Alnylam: AMVUTTRA; GSK: Shingrix, Arexvy. B - Research funding under your direct/personal responsibility (directed to the department or institution) from the brazilian or international pharmaceutical, orthosis, prosthesis, equipment and implants industry: - PTCBio: Waylivra; Amgen: Repatha; Novartis: Inclisiran, Pelacarsen; NovoNordisk: Ziltivekimab. Other relationships Funding of continuing medical education activities, including travel, accommodation and registration in conferences and courses, from the brazilian or international pharmaceutical, orthosis, prosthesis, equipment and implants industry: - Novo Nordisk: Diabetes, Ziltivekimab; GSK: vaccines. Any other interest — financial or other — that should be declared considering the position taken in SBC that has not been expressly listed above: - Member of the Management Committee of the Hipertri Brasil Network.
Maurício Alves Barreto	Financial declaration A - Economically relevant payments of any kind made to (i) you, (ii) your spouse/partner or any other person living with you, (iii) any legal person in which any of these is either a direct or indirect controlling owner, business partner, shareholder or participant; any payments received for lectures, lessons, training instruction, compensation, fees paid for participation in advisory boards, investigative boards or other committees, etc. from the brazilian or international pharmaceutical, orthosis, prosthesis, equipment and implants industry: - Industry classes: Novo Nordisk: Rybelsus; DM2 and Wegovy: obesity; Novartis: Sybrava, dyslipidemia; Biolab: Repatha, dyslipidemia; Libbs: Plenance Eze, dyslipidemia; Merck: Contrave, obesity; scientific writing: Aché, dyslipidemia; clinical research: principal investigator in studies sponsored by Amgen. (OCEAN Outcomes). Arrowhead pharmaceuticals (SHASTA-3). Other relationships Funding of continuing medical education activities, including travel, accommodation and registration in conferences and courses, from the brazilian or international pharmaceutical, orthosis, prosthesis, equipment and implants industry: - Novo Nordisk: Rybelsus, DM2.
Natasha Slhessarenko Fraife Barreto	Nothing to be declared
Paulo Eduardo Ballvé Behr	Financial declaration A - Economically relevant payments of any kind made to (i) you, (ii) your spouse/partner or any other person living with you, (iii) any legal person in which any of these is either a direct or indirect controlling owner, business partner, shareholder or participant; any payments received for lectures, lessons, training instruction, compensation, fees paid for participation in advisory boards, investigative boards or other committees, etc. from the brazilian or international pharmaceutical, orthosis, prosthesis, equipment and implants industry: - Novartis: Sybrava; Novartis: Cosentyx; Biolab: Livalo; Aché: Trezete; PTC: Volanesorsena; Libbs: Zinpass; Novo Nordisk: Ozempic; Daiichi Sankyo: Nustendi; Amgen: Olpasiran. Other relationships Funding of continuing medical education activities, including travel, accommodation and registration in conferences and courses, from the brazilian or international pharmaceutical, orthosis, prosthesis, equipment and implants industry: - Novartis: Sybrava; Daiichi: Nustendi; Novo Nordisk: Ozempic.
Pedro Gabriel Melo de Barros e Silva	Nothing to be declared
Pedro Pimentel Filho	Financial declaration A - Economically relevant payments of any kind made to (i) you, (ii) your spouse/partner or any other person living with you, (iii) any legal person in which any of these is either a direct or indirect controlling owner, business partner, shareholder or participant; any payments received for lectures, lessons, training instruction, compensation, fees paid for participation in advisory boards, investigative boards or other committees, etc. from the brazilian or international pharmaceutical, orthosis, prosthesis, equipment and implants industry: - Clinical research in Cardiology with participation in studies by companies such as Amgen, Bayer, AstraZeneca, Janssen, Lilly. B - Research funding under your direct/personal responsibility (directed to the department or institution) from the brazilian or international pharmaceutical, orthosis, prosthesis, equipment and implants industry: - Amge: Evolocumab; AstraZeneca: Forxiga; MSD: MK 606 Bayer: Finerinone; Janssen: Milvexian; all in the cardiovascular area. Other relationships Funding of continuing medical education activities, including travel, accommodation and registration in conferences and courses, from the brazilian or international pharmaceutical, orthosis, prosthesis, equipment and implants industry: - Daiichi Sankyo: bempedoic acid.
Raul Cavalcante Maranhão	Nothing to be declared
Raul Dias dos Santos Filho	Financial declaration A - Economically relevant payments of any kind made to (i) you, (ii) your spouse/partner or any other person living with you, (iii) any legal person in which any of these is either a direct or indirect controlling owner, business partner, shareholder or participant; any payments received for lectures, lessons, training instruction, compensation, fees paid for participation in advisory boards, investigative boards or other committees, etc. from the brazilian or international pharmaceutical, orthosis, prosthesis, equipment and implants industry: - Amgen; Novartis;, Arrowhead; Ionis; Torrent, Sanofi; Daiichi Sankyo; Aché: hypolipidemic agents; Novo Nordisk, Eli-Lilly: hypoglycemic agents. B - Research funding under your direct/personal responsibility (directed to the department or institution) from the brazilian or international pharmaceutical, orthosis, prosthesis, equipment and implants industry: - Amgen; Arrowhead, Ionis; Eli-Lilly: lipid-lowering drugs.
Renato Jorge Alves	Financial declaration A - Economically relevant payments of any kind made to (i) you, (ii) your spouse/partner or any other person living with you, (iii) any legal person in which any of these is either a direct or indirect controlling owner, business partner, shareholder or participant; any payments received for lectures, lessons, training instruction, compensation, fees paid for participation in advisory boards, investigative boards or other committees, etc. from the brazilian or international pharmaceutical, orthosis, prosthesis, equipment and implants industry: - Novartis: Inclisirana; Viatris: Sertraline; Aché: Trezet; Mantecorp: Coledue R; Server: Acertil; Libbs: Plenance; GSK: Vaccines; EMS: Valsartan. Other relationships Funding of continuing medical education activities, including travel, accommodation and registration in conferences and courses, from the brazilian or international pharmaceutical, orthosis, prosthesis, equipment and implants industry: - Novartis: Inclisirana; Mantecorp: Coledue R.
Sergio Emanuel Kaiser	Financial declaration A - Economically relevant payments of any kind made to (i) you, (ii) your spouse/partner or any other person living with you, (iii) any legal person in which any of these is either a direct or indirect controlling owner, business partner, shareholder or participant; any payments received for lectures, lessons, training instruction, compensation, fees paid for participation in advisory boards, investigative boards or other committees, etc. from the brazilian or international pharmaceutical, orthosis, prosthesis, equipment and implants industry: - Bayer: vericiguat and FiriHIGH; Daiichi Sankyo: Nustendi, Lixiana and Benicar; Novo Nordisk: Wegovy; Biolab: Repatha; Libbs:, Naprix; Astrazeneca: Forxiga and Selozok - Novartis, Sybrava, Boehringer Ingelheim: Jardiance; Pharmacochemistry: Rosuvastatin. Other relationships Funding of continuing medical education activities, including travel, accommodation and registration in conferences and courses, from the brazilian or international pharmaceutical, orthosis, prosthesis, equipment and implants industry: - Novo Nordisk: Wegovy; Daiichi Sanlyo: Nustendi; Astrazeneca: heart failure; Bayer: FiriHIGH; Novartis: Sybrava.
Valéria Arruda Machado	Financial declaration A - Economically relevant payments of any kind made to (i) you, (ii) your spouse/partner or any other person living with you, (iii) any legal person in which any of these is either a direct or indirect controlling owner, business partner, shareholder or participant; any payments received for lectures, lessons, training instruction, compensation, fees paid for participation in advisory boards, investigative boards or other committees, etc. from the brazilian or international pharmaceutical, orthosis, prosthesis, equipment and implants industry: - Abbott: nutrition.
Viviane Zorzanelli Rocha	Financial declaration A - Economically relevant payments of any kind made to (i) you, (ii) your spouse/partner or any other person living with you, (iii) any legal person in which any of these is either a direct or indirect controlling owner, business partner, shareholder or participant; any payments received for lectures, lessons, training instruction, compensation, fees paid for participation in advisory boards, investigative boards or other committees, etc. from the brazilian or international pharmaceutical, orthosis, prosthesis, equipment and implants industry: - Abbott: Lipidyl; Aché: Thirteen; Biolab: Repatha; Daichii-Sankyo: Nustendi; Lilly: Mounjaro; Novartis: Sybrava; Novo-Nordisk: Wegovy; Ultragenyx: Evkeeza. Other relationships Funding of continuing medical education activities, including travel, accommodation and registration in conferences and courses, from the brazilian or international pharmaceutical, orthosis, prosthesis, equipment and implants industry: - Novartis: Sybrava; Novo Nordisk: Wegovy.

## Table of Contents

**Preamble** 13
**1. Introduction** 15
**1.1. The Brazilian Population Perspective** 15
**1.2. Expanded Focus: From Dyslipidemia to Prevention of Atherosclerosis** 15
**1.2.1. Early Phase – Childhood and Adolescence** 15
**1.2.2. Intermediate Phase – Young to Middle-Aged Adults** 15
**1.2.3. Late Phase – Older Adults and Patients with Established Clinical Disease** 15
**1.3. A Stratified Model for Prevention of Atherosclerotic Cardiovascular Disease** 15
**1.4. Key Points of the 2025 Brazilian Guideline on Dyslipidemias and Prevention of Atherosclerosis** 15
**1.5. Strength of Recommendation and Certainty of Evidence** 16
**1.6. Summary of Recommendations** 17
**2. Epidemiology** 27
**2.1. Average Plasma Lipid Levels and Prevalence of Dyslipidemia** 27
**2.2. Cardiovascular Mortality Attributable to Increased Low-density Lipoprotein Cholesterol** 29
**2.3. Data on Treatment and Target Achievement** 29
**3. Diagnosis** 31
**3.1. Laboratory Assessment of Lipid Parameters and Apolipoproteins** 31
**3.1.1. Pre-Analytical and Analytical Phases** 31
**3.1.1.1. Pre-Analytical Phase** 31
**3.1.1.2. Analytical Phase** 32
**3.1.1.2.1. Research-Restricted Methods** 32
**3.1.1.2.2. Conventional Methods – Routine Laboratory Practice** 32
**3.1.1.2.2.1. Colorimetric Enzymatic Methods** 32
**3.1.1.2.2.2. Point of Care Testing** 32
**3.1.1.2.2.3. Calculation of Low-Density Lipoprotein Cholesterol** 33
**3.1.1.2.2.4. Measurement of Non-High-Density Lipoprotein Cholesterol** 33
**3.1.1.2.2.5. Measurement of Apolipoprotein B** 33
**3.1.1.2.2.6. Lipoprotein(a)** 34
**3.1.1.2.2.7. Reference Values for the Lipid Profile** 35
**3.2. Genetic Diagnosis of Dyslipidemias** 35
**3.2.1. Genetically-Based Hypercholesterolemias** 35
**3.2.1.1. Considerations for Requesting Genetic Testing** 35
**3.3. Diagnosis of Hypertriglyceridemia** 38
**3.3.1. Familial Chylomicronemia Syndrome** 38
**3.3.1.1. Definition** 38
**3.3.1.2. Clinical and Laboratory Diagnosis of Familial Chylomicronemia Syndrome** 38
**3.3.1.3. Diagnostic Scores** 38
**3.3.1.4. Differential Diagnosis** 38
**3.3.1.5. Genetic Diagnosis** 38
**3.3.1.6. Lipoprotein Lipase Activity** 39
**3.3.1.7. Other Diagnostic Tests** 39
**4. Risk Stratification** 39
**4.1. Cardiovascular Risk Stratification** 39
**4.2. Cardiovascular Risk Scores** 41
**4.3. Cardiovascular Risk Enhancers** 41
**4.3.1. Family History of Premature Cardiovascular Disease** 42
**4.3.2. Adiposity and its Manifestations** 42
**4.3.3. Chronic Inflammatory Conditions** 43
**4.3.4. Organ Transplantation** 43
**4.3.5. Women-Specific Cardiovascular Risk-Enhancing Factors** 43
**4.3.5.1. Age at Menarche** 43
**4.3.5.2. Pregnancy-Related Disorders and Preterm Birth** 43
**4.3.5.3. Recurrent Miscarriages** 43
**4.3.5.4. Premature Menopause** 44
**4.4. Additional Tests** 44
**4.4.1. Lipoprotein(a)** 44
**4.4.2. High-Sensitivity C-Reactive Protein** 44
**4.4.3. High-Sensitivity Cardiac Troponins** 44
**4.4.4. B-Type Natriuretic Peptide and N-Terminal Pro-B-Type Natriuretic Peptide** 44
**4.5. Markers of Subclinical Atherosclerotic Disease** 45
**4.5.1. Coronary Artery Calcium Score** 45
**4.5.2. Carotid Artery Ultrasound** 45
**4.6. Cardiovascular Risk Stratification in Diabetes** 47
**4.7. Categories of Atherosclerotic Cardiovascular Risk** 47
**4.8. Particularities of Cardiovascular Risk Stratification in Older Adults** 48
**4.9. Particularities of Cardiovascular Risk Stratification in Young Adults** 48
**4.10. Cardiovascular Risk Stratification in Childhood and Adolescence** 49
**5. Treatment Targets** 50
**5.1. Primary and Co-Primary Target: Low-Density Lipoprotein Cholesterol and Non-High-Density Lipoprotein Cholesterol** 50
**5.2. Recommendations for Targets According to Cardiovascular Risk Stratification** 50
**5.2.1 Individuals at Intermediate Risk** 50
**5.2.2. Individuals at Intermediate Risk** 51
**5.2.3. Individuals at High Risk** 51
**5.2.4. Individuals at Very High Risk** 51
**5.2.5. Individuals at Extreme Risk** 52
**5.3. Apolipoprotein B** 52
**5.4. High-Density Lipoprotein Cholesterol** 52
**5.5. Triglycerides** 52
**5.6. Lipoprotein(a)** 53
**6. Nonpharmacological Treatment** 53
**6.1. Lifestyle Recommendations to Improve Lipid Profile** 53
**6.1.1. Nutritional Aspects** 53
**6.1.2. Carbohydrates** 53
**6.1.3. Fats** 54
**6.1.4. Soluble Fiber** 54
**6.2. Smoking Cessation** 54
**6.3. Management of Weight** 54
**6.4. Spirituality** 55
**6.5. Physical Activity** 55
**6.6. Alcohol Intake** 55
**6.7. Dietary Supplements and Functional Foods in Dyslipidemia** 56
**7. Pharmacological Treatment** 56
**7.1. Statins** 56
**7.2. Ezetimibe** 58
**7.3. Novel Messenger RNA-Targeting Therapies** 58
**7.4. Anti-Proprotein Convertase Subtilisin/Kexin Type 9 Therapy** 58
**7.5. Bempedoic Acid** 59
**7.6. Cholesteryl Ester Transfer Protein Inhibitors and High-Density Lipoprotein Cholesterol–Raising Therapies** 59
**7.7. Fibrates** 59
**7.8. Omega-3 Fatty Acids** 60
**7.9. Apolipoprotein C-III Inhibitors** 60
**7.10. Angiopoietin-Like Protein 3 Inhibitors** 60
**7.11. Lipoprotein(a) Inhibitors** 61
**7.12. Clustered Regularly Interspaced Short Palindromic Repeats and Gene Therapies** 61
**7.13. Combination Therapy** 62
**7.13.1. Benefits of Combination Therapy** 62
**7.13.2. Statin and Ezetimibe Combination** 62
**7.13.3 Statin and Proprotein Convertase Subtilisin/Kexin Type 9-Targeted Therapy Combination** 62
**7.13.4. Ezetimibe and Bempedoic Acid Combination** 62
**7.13.5. Statin, Ezetimibe, and Proprotein Convertase Subtilisin/Kexin Type 9-Targeted Therapy Combination** 63
**7.13.6. Statin, Ezetimibe, and Bempedoic Acid Combination** 63
**8. Management of Statin Intolerance** 64
**8.1. Definition** 64
**8.2. Prevalence** 64
**8.3. Diagnosis** 64
**8.4. Nocebo Effect** 64
**8.5. Muscle Symptoms** 64
**8.5.1. Clinical Characteristics, Classification, and Management of Statin-Associated Muscle Symptoms** 64
**8.5.2. Tolerable and Intolerable Muscle Symptoms** 65
**8.5.3. Creatine Kinase Elevation** 65
**8.5.4. Rhabdomyolysis** 66
**8.5.5. Statin-Induced Immune-Mediated Necrotizing Myopathy** 67
**8.6. Factors Associated with Statin Intolerance** 67
**8.7. Management of Statin-Intolerant Patients** 67
**8.7.1. Discontinuation and Reintroduction of Statin Therapy** 67
**8.7.2. Use of Products Without Proven Benefit** 68
**8.7.3. Drug interactions of statins** 68
**8.7.3.1. Anticoagulants** 68
**8.7.3.2. Azole Antifungals** 72
**8.7.3.3. Antiretroviral Agents** 72
**8.7.3.4. Calcium Channel Blockers** 72
**8.7.3.5. Antiarrhythmic Agents** 72
**8.7.3.6. Immunosuppressants** 72
**8.7.3.7. Macrolides** 72
**8.7.3.8 Interactions Between Lipid-Lowering Agents** 72
**9. Dyslipidemia in Specific Populations: Clinical Management Considerations** 73
**9.1. Heart Failure** 73
**9.2. People Living with HIV** 74
**9.3. Diabetes** 74
**9.3.1. Specific Characteristics of Dyslipidemia in Insulin Resistance and Type 2 Diabetes** 75
**9.3.2. Treatment of Dyslipidemia in Patients with Diabetes** 75
**9.3.3. Pharmacological Treatment** 75
**9.4. Hypothyroidism** 76
**9.5. Chronic Kidney Disease** 77
**9.6. Obesity** 78
**9.7. Older Individuals** 79
**9.8. Nonpharmacological Treatment** 79
**9.9. Pharmacological Treatment** 79
**9.10. Children** 80
**9.10.1. Lipid Profile in Childhood** 80
**9.10.2. Screening** 80
**9.10.3. Primary Dyslipidemias** 80
**9.10.4. Homozygous Familial Hypercholesterolemia** 80
**9.10.5. Hypertriglyceridemias** 80
**9.10.6. Monogenic Hypertriglyceridemia (Severe Hypertriglyceridemias)** 80
**9.10.7. Secondary Dyslipidemias** 80
**9.10.8. Statin Therapy is Indicated Based on risk in Secondary Dyslipidemias, Particularly in High-Risk Conditions or in the Presence of Risk Factors (Threshold Values for Initiating Treatment)** 81
**9.11. Transplant Recipients** 81
**9.12. Chronic Liver Diseases** 81
**9.12.1. Metabolic Dysfunction-Associated Steatotic Liver Disease** 83
**9.12.1.1. Definition** 83
**9.12.1.2. Prevalence and Cardiovascular Risk** 83
**9.12.1.3. Reducing Cardiovascular Risk** 83
**9.12.1.4. Liver Outcomes** 83
**9.12.1.5. Safety** 83
**9.12.1.6. Intrahepatic Cholestasis** 83
**9.12.1.7. Hepatic Cirrhosis** 83
**9.12.1.8. Hepatocellular Carcinoma** 84
**9.13. Acute Coronary Syndrome** 84
**9.14. Immune-Mediated Diseases** 85
**9.15. Pregnancy** 86
**9.15.1. Gestational Dyslipidemia in Normolipidemic Women** 86
**9.15.2. Gestational Dyslipidemia in Women with Pre-Existing Dyslipidemia** 86
**9.15.3. Lipoprotein(a)** 86
**9.15.4. Pharmacological Treatment** 86
**9.15.4.1. Statins** 86
**9.15.4.2. New Evidence** 86
**9.15.4.3. Bile Acid Sequestrants** 87
**9.15.4.4. Lipoprotein Apheresis** 87
**9.15.4.5. Ezetimibe** 87
**9.15.4.6. Omega-3 Fatty Acids** 87
**9.16. Women** 87
**10. Conclusion** 88
**References** 89

## Preamble

Despite major advances in understanding the pathophysiology of atherosclerosis and its consequences, as well as the development of new preventive therapies, ASCVD remains the leading cause of death worldwide.^1 , 2^The global rise in obesity and diabetes often leads to dyslipidemia, which is characterized by low levels of high-density lipoprotein cholesterol, high levels of non-high-density lipoprotein cholesterol, and high triglyceride levels.^3 , 4^Of particular concern is the increasing prevalence of ASCVD in low- and middle-income countries, where most of the global population resides. This disease tends to manifest in these populations about a decade or earlier than in high-income countries.^5^

The importance of clinical guidelines in helping health professionals deliver more effective management and improved outcomes in various clinical situations has been well-documented. Adherence to guidelines improves patient prognosis. Furthermore, guidelines provide a framework that informs the development of public health policies based on solid scientific evidence. They also identify barriers to implementing best practices and offer potential solutions adapted to different regions. The Brazilian Society of Cardiology's current Guideline on Cholesterol Management and Prevention of Atherosclerosis is a prime example of this. It serves as a valuable tool for clinicians dealing with these conditions and for policymakers responsible for implementing measures to reduce the population's burden of lipid metabolism disorders.

Today, the role of elevated blood cholesterol levels in the global burden of atherosclerotic disease is well established. Lowering low-density lipoprotein cholesterol (LDL-c) has been consistently shown to be beneficial across the continuum of cardiovascular risk, with individuals at higher risk of atherothrombotic events — such as myocardial infarction, stroke, revascularization, or cardiovascular death — achieving greater absolute risk reductions from LDL-c decrease. The timing and duration of treatment in the course of atherosclerotic disease are critical and should clearly inform health policies and, above all, their implementation. Nevertheless, most patients worldwide do not achieve the recommended LDL-c decrease needed to minimize their individual ASCVD risk. This is largely due to the underuse of high-intensity statins as first-line therapy, insufficient use of combination strategies, and poor adherence to lipid-lowering regimens — all of which contribute to persistently high cumulative cholesterol exposure. Thus, the importance of early combination therapy is increasingly recognized to achieve better outcomes and have a more significant impact on reducing the atherosclerotic burden.^6^

It is also well established that LDL-c reduction benefits not only individuals with high cholesterol levels but also those at higher risk for the main clinical manifestations of atherosclerosis.^7^Therefore, LDL-c decrease should be considered for all high-risk individuals,^8^including those with i) established or previous ASCVD; ii) high-risk conditions such as diabetes, chronic kidney disease, tobacco use, or hypertension, which increase ASCVD risk even in the absence of concurrent lipid abnormalities; iii) extreme elevations of LDL-c with a genetic basis, such as heterozygous familial hypercholesterolemia (FH); iv) high overall cardiovascular risk due to the combined effects of multiple risk factors; v) isolated elevations of atherogenic lipoproteins, including triglyceride-rich lipoproteins (commonly referred to as atherogenic dyslipidemia) or elevated lipoprotein(a) (Lp(a)); and vi) high subclinical coronary atherosclerotic burden.^9^

The treatment and control of dyslipidemias remain one of the major medical challenges of our time. The availability of up-to-date, well-designed guidelines, such as the current document, provides an essential tool to help achieve the goal of reducing the burden of cardiovascular diseases — and particularly ASCVD.

**Fausto J Pinto**

## 1. Introduction

ASCVD remains the leading cause of death worldwide, despite significant advances in the understanding of its pathophysiology and the development of preventive therapies.^10^In Brazil, the same pattern holds: ASCVD is the condition with the greatest impact on morbidity and mortality, affecting millions of people and placing a heavy burden on the health care system.^11^

Particularly concerning is the rising prevalence of ASCVD in low- and middle-income countries, such as Brazil, where most of the global population lives.^3^In such settings, ASCVD often manifests at least a decade earlier than in high-income countries, affecting not only individual health but also economic productivity during the most active years of life. The burden further extends to family members and caregivers, compounding the social impact of ASCVD.

Atherosclerosis is a chronic, silent, progressive disease that begins in childhood. Histopathological studies have shown that early changes in the arterial wall, such as fatty streaks, may appear as early as the first decade of life. Therefore, ASCVD should not be viewed solely as a disease of adulthood or old age, but rather as a continuous process with multiple opportunities for intervention and prevention throughout life.^12 , 13^

Thus, updated guidelines tailored to the Brazilian context are essential for developing strategies to prevent, diagnose early, and effectively treat ASCVD at all stages of life ( Central Illustration ).

### 1.1. The Brazilian Population Perspective

With an estimated population of 218,56 million in 2025, Brazil has an age distribution that poses specific challenges for CVD prevention. Estimates indicate that:

- Around 25% of the population (about 54 million) are under the age of 20;
- Around 54% (about 118 million) are between 20 and 60 years old;
- And 21% (about 45 million) are over the age of 60.

This distribution underscores the need for preventive strategies tailored to each stage of life and to the degree of atherosclerosis progression, with an emphasis on early, continuous, targeted interventions.

### 1.2. Expanded Focus: From Dyslipidemia to Prevention of Atherosclerosis

Although dyslipidemia is a central factor in the development of atherosclerosis, the current guideline adopts a broader approach, with the primary goal of preventing ASCVD. According to data from the GBD (Global Burden of Disease) study, increased LDL-c and hypertension are the two main risk factors responsible for the highest number of CV deaths in Brazil.^14^Thus, the guideline proposes a life-course model of action with specific and graduated interventions:

#### 1.2.1. Early Phase – Childhood and Adolescence

- Primordial prevention focused on promoting healthy habits and preventing the onset of risk factors.
- Screening for genetic dyslipidemias, such as FH, with early intervention in children and adolescents.
- No clinical manifestations, but the possibility of initial histopathological changes in the arterial wall.

#### 1.2.2. Intermediate Phase – Young to Middle-Aged Adults

- Frequent presence of subclinical atherosclerotic disease (SAD) detectable by imaging methods (eg, carotid ultrasound, coronary artery calcium [CAC] score).
- Indication for early and intensive intervention through lifestyle modification and, when necessary, pharmacologic therapy.
- Objective: to halt disease progression and reduce the lifetime risk of major CV events.

#### 1.2.3. Late Phase – Older Adults and Patients with Established Clinical Disease

- Presence of manifest ASCVD, such as acute myocardial infarction (MI), stroke, or peripheral arterial disease (PAD).
- Need for intensive treatment and aggressive therapeutic targets, especially for lipid control.
- Emphasis on individuals at higher cardiovascular risk within the continuum and on reducing the recurrence of cardiovascular events.

### 1.3. A Stratified Model for Prevention of Atherosclerotic Cardiovascular Disease

Atherosclerosis must be understood as a chronic, progressive, and early-onset condition that demands a preventive approach across the entire life course. The current guideline proposes a model of care that goes beyond isolated dyslipidemia control, incorporating individualized risk stratification, the use of emerging biomarkers (eg, Lp(a), apolipoprotein B [ApoB], and high-sensitivity C-reactive protein [hs-CRP]), as well as imaging tools for the detection of subclinical atherosclerosis, such as CAC scoring, and the adoption of evidence-based therapeutic targets.

By recognizing the heterogeneity of the Brazilian population and the complexity of ASCVD, the current guideline reinforces the importance of integrated public policies, health education, and equitable access to effective diagnostic and therapeutic strategies.

### 1.4. Key Points of the 2025 Brazilian Guideline on Dyslipidemias and Prevention of Atherosclerosis

The 2025 Brazilian Guideline on Dyslipidemias and Prevention of Atherosclerosis updates and expands upon the concepts of the 2017 version, maintaining a focus on risk stratification while adopting a more refined and personalized approach. The focus is centered on the continuum of cardiovascular risk, beginning with the early and accurate identification of aggravating factors — such as a family history of premature cardiovascular disease and obesity, among others — and progressing toward stratification into very high and extreme risk categories, where therapeutic approaches should be more intensive. This assessment includes not only classical risk factors but also biomarkers, such as Lp(a), ApoB, and hs-CRP, as well as imaging tools for the detection of subclinical atherosclerosis, such as CAC scoring ( Box 1.1 ).

**Box 1.1:** Top 10 Key Messages from the 2025 Brazilian Guideline on Dyslipidemias and Prevention of Atherosclerosis

No.	Key Message	Description
**1**	Promotion of healthy lifestyle throughout life	The foundation of CV prevention is the early and sustained adoption of healthy habits, such as a balanced diet, regular physical activity, and risk factor control
**2**	One-time measurement of Lp(a)	A single lifetime measurement of Lp(a) is recommended for all adults to identify increased residual risk
**3**	Non-HDL-c as a co-primary target	Non-HDL-c is recognized as a co-primary treatment target alongside LDL-c, especially in patients with HTG
**4**	ApoB as a secondary target	ApoB is recommended as a complementary target, particularly when LDL-c and non-HDL-c are within target levels
**5**	Use of the PREVENT score for risk stratification	PREVENT is the preferred tool for risk stratification in adults without known ASCVD
**6**	Consideration of risk enhancers for reclassification	Factors such as increased Lp(a), chronic inflammatory diseases, and family history should be considered for risk reclassification and treatment intensification
**7**	Use of CAC scoring and imaging in selected cases	In intermediate-risk patients, tests such as the CAC score may help guide therapeutic decisions
**8**	LDL-c target < 115 mg/dL for individuals at low CV risk	Pharmacologic treatment should be considered if LDL-c remains ≥ 145 mg/dL, even in individuals at low CV risk
**9**	Recognition of extreme CV risk category	A new category with an LDL-c target of <40 mg/dL for patients at extremely high risk
**10**	Early combination therapy as an initial strategy	Early initiation of statin + ezetimibe ± anti-PCSK9itherapies may be appropriate for patients at high, very high, or extreme risk

CV: cardiovascular; Non-HDL-c: non HDL cholesterol; Lp(a): Lipoprotein (a); ApoB: apolipoprotein B; ASCVD: atherosclerotic cardiovascular disease; CAC: coronary artery calcium; CV: cardiovascular; HTG: hypertriglyceridemia; LDL-c: low-density lipoprotein cholesterol; Lp(a): lipoprotein(a); Non-HDL-c: non-high-density lipoprotein cholesterol; PCSK9: proprotein convertase subtilisin/kexin type 9; PREVENT: Predicting risk of cardiovascular disease events.

Based on this expanded stratification, therapeutic targets are more intensive: LDL-c levels < 50 mg/dL for very high-risk patients and < 40 mg/dL for those at extreme risk. Combination therapy is now recommended as first-line treatment in these groups, including statins combined with ezetimibe, anti-PCSK9 (proprotein convertase subtilisin/kexin type 9) therapy, and bempedoic acid (BA) — particularly in cases of statin intolerance or the need for further cardiovascular (CV) risk reduction.

Another important advancement is the structured management of statin intolerance, which is often underestimated in clinical practice. The guideline offers clear algorithms for diagnosis and effective therapeutic alternatives.

Finally, recognizing the specific challenges faced in Brazil — such as low treatment adherence and difficulty achieving therapeutic targets — the current guideline aims not only to update technical knowledge but also to transform clinical practice, promoting better outcomes and reducing the burden of CVD in the country.

### 1.5. Strength of Recommendation and Certainty of Evidence

The recommendations were developed according to the Grading of Recommendations Assessment, Development and Evaluation (GRADE) methodology, which classifies the strength of recommendation (strong or weak/conditional) and the certainty of evidence (high, moderate, low, or very low) ( Boxes 1.2 and 1.3 ).

- The development of this guideline was structured into thematic chapters, each coordinated by experts with recognized expertise in their respective fields. Each coordinator was responsible for assembling a group of authors, with whom a critical review of the available scientific literature was carried out, aiming to base the recommendations on the best available evidence.
- The working groups held periodic meetings to discuss the topics and draft the recommendations, under the supervision of the guideline's general coordination. The recommendations were subsequently reviewed and refined by the editorial committee, based on predefined technical and scientific criteria.
- The final text of each chapter was submitted for review and approval by all committee members, ensuring methodological alignment and consistency across the content presented.

**Box 1.2:** Strength of Recommendation

Category	Definition
STRONG	Should be done for most patients, in nearly all circumstances.
CONDITIONAL	May be done depending on individual circumstances, preferences, and context.

**Box 1.3:** Certainty of Evidence

Category	Definition
HIGH	There is high confidence that the true effect is close to the estimated effect.
MODERATE	There is moderate confidence in the estimated effect.
LOW	Confidence in the effect is limited.
VERY LOW	Confidence in the estimated effect is very limited. There is a substantial degree of uncertainty in the findings.

### 1.6. Summary of Recommendations

**Table:** Recommendations for the collection and interpretation of the lipid profile in the diagnosis of dyslipidemia

Recommendation	Strength of Recommendation	Certainty of Evidence
Ideally, the lipid profile sample should be collected under stable metabolic conditions.	STRONG	MODERATE
For initial assessment, nonfasting samples are acceptable, particularly in selected populations such as children and older adults.	STRONG	MODERATE
If TG levels are increased (> 440 mg/dL) in a nonfasting sample, a repeat 12-hour fasting sample is recommended, according to the referring physician's discretion.	STRONG	MODERATE

TG: triglycerides.

**Table:** Recommendations for the calculation of low-density lipoprotein cholesterol (LDL-c)

Recommendation	Strength of Recommendation	Certainty of Evidence
The use of the Martin/Hopkins equation for LDL-c calculation is recommended for all individuals.	STRONG	MODERATE
For TG values >800 mg/dL, LDL-c results using the Martin/Hopkins formula may be underestimated. Evaluation of non–HDL-c is recommended.	STRONG	MODERATE

LDL-c: low-density lipoprotein cholesterol; Non-HDL-c: non-high-density lipoprotein cholesterol; TG: triglycerides.

**Table:** Recommendations on the use of non-HDL cholesterol in cardiovascular risk assessment

Recommendation	Strength of Recommendation	Certainty of Evidence
Both LDL-c and non-HDL-c are highly useful for assessing CV risk and as therapeutic targets. Non-HDL-c is particularly valuable for estimating the amount of circulating atherogenic lipoproteins in individuals with increased TG levels (> 150 mg/dL).	STRONG	HIGH

LDL-c: low-density lipoprotein cholesterol; Non-HDL-c: non-HDL cholesterol; TG: triglycerides.

**Table:** Recommendations on the use of Apolipoprotein B (ApoB) in cardiovascular risk assessment

Recommendation	Strength of Recommendation	Certainty of Evidence
Measurement of ApoB may help in assessing CV risk and guiding therapy in individuals with HTG (TG > 150 mg/dL).	STRONG	MODERATE
Non-HDL-c is currently a more practical option because it can be easily calculated and does not impose additional costs for the patient or the health care system.	STRONG	HIGH

ApoB: apolipoprotein B; HTG: hypertriglyceridemia; non-HDL-c: non-HDL cholesterol; TG: triglycerides.

**Table:** Recommendations on the use of lipoprotein(a) (Lp(a)) in cardiovascular risk stratification

Recommendation	Strength of Recommendation	Certainty of Evidence
In the general population, measuring Lp(a) once in a lifetime is recommended when available to assist with risk stratification and/or therapeutic management.	STRONG	MODERATE
In specific conditions, such as premature CAD, aortic stenosis, FH, family history of early ASCVD, or increased Lp(a), measuring Lp(a) once in a lifetime is recommended when available to assist with risk stratification and/or therapeutic management.	STRONG	HIGH
The preferred method for measuring Lp(a) is an assay that is isoform-independent, meaning it measures the number of particles per liter (nmol/L). Measurement in mass units (mg/dL) should be avoided. Conversion formulas do not correct the differences between methods and are not recommended.	STRONG	HIGH
Measurement of Lp(a) by isoform-dependent assay (ie, in mass units [mg/dL]), may be used when it is the only method available.	STRONG	HIGH
In individuals with elevated lipoprotein(a) levels – ≥50 mg/dL (or ≥125 nmol/L) –, whose concentration is predominantly genetically determined, cascade screening of family members is recommended to help identify other potential carriers and enable early cardiovascular risk assessment.	STRONG	HIGH

ASCVD: atherosclerotic cardiovascular disease; CAD: coronary artery disease; FH: familial hypercholesterolemia; LDL-c: low-density lipoprotein cholesterol; Lp(a): lipoprotein(a).

**Table:** Recommendations for genetic testing in familial hypercholesterolemia (FH)

Recommendation	Strength of Recommendation	Certainty of Evidence
**Proband (index case) -** Genetic testing for FH must be offered to individuals of any age with a strong clinical suspicion of FH, based on their personal and/or family medical history. Note: This suspicion includes the following situations: Children with persistent * LDL-c levels ≥ 160 mg/dL or adults with persistent * LDL-c ≥ 190 mg/dL without an apparent secondary cause of hypercholesterolemia † and with at least one first-degree relative who is also affected, or with premature ‡ CAD, or if the family history is unavailable (eg, in cases of adoption). Children with persistent * LDL-c ≥ 190 mg/dL or adults with persistent * LDL-c ≥ 250 mg/dL without an apparent secondary cause of hypercholesterolemia † , even in the absence of a positive family history.	STRONG	MODERATE
**At-risk family members –** Cascade genetic testing for the specific variant(s) identified in the proband with FH (testing of the known familial variant) must be offered to all first-degree relatives. If first-degree relatives are unavailable or decline testing, the known familial variant should be offered to second-degree relatives. Cascade testing should continue throughout the extended family until all at-risk individuals have been tested and all relatives with FH have been identified.	STRONG	MODERATE

* Persistent: Increased LDL-c levels confirmed on at least two separate occasions.

† Secondary causes of hypercholesterolemia include hypothyroidism, nephrotic syndrome, cholestatic liver disease, and certain medications.

‡ Premature CAD: < 55 years in men and < 65 years in women. CAD: coronary artery disease; LDL-c: low-density lipoprotein cholesterol; FH: familial hypercholesterolemia.

**Table:** Recommendations for genetic testing for familial hypercholesterolemia in different clinical scenarios

Recommendation	Strength of Recommendation	Certainty of Evidence
Genetic testing for FH may be considered in adults without available pretreatment LDL-c levels, but with a personal history of premature coronary artery disease ‡ and a family history of hypercholesterolemia and premature coronary artery disease ‡ .	CONDITIONAL	MODERATE
Genetic testing may be considered in adults with persistent LDL-c levels ≥160 mg/dL (in the absence of an apparent secondary cause of hypercholesterolemia † ) in the context of a family history of hypercholesterolemia and a personal or family history of premature coronary artery disease ‡ . *	CONDITIONAL	MODERATE

* Persistent: Increased LDL-cevels confirmed on at least two separate occasions;

† Secondary causes of hypercholesterolemia include hypothyroidism, nephrotic syndrome, cholestatic liver disease, and certain medications;

‡ Premature CAD: < 55 years in men and < 65 years in women. CAD: coronary artery disease; LDL-c low-density lipoprotein cholesterol.

**Table:** Recommendations for complementary diagnostic tests in suspected familial chylomicronemia syndrome

Recommendation	Strength of Recommendation	Certainty of Evidence
Clinical scores are recommended to diagnose FCS.	STRONG	HIGH
Under ideal circumstances, genetic testing is the recommended method for confirming the diagnosis of FCS.	STRONG	HIGH
The genetic panel for FCS should include sequencing of the LPL, GPIHBP1, LMF1, APOA5, and APOC2 genes.	STRONG	HIGH

FCS: familial chylomicronemia syndrome.

**Table:** Recommendations for cardiovascular risk stratification in adults

Recommendation	Strength of Recommendation	Certainty of Evidence
For individuals aged 30-79 years without established CVD, the use of a risk equation to estimate the 10-year risk of an ASCVD event is STRONGLY recommended.	STRONG	HIGH
For individuals aged 30-79 years without established CVD, using PREVENT to estimate the risk of an ASCVD event is STRONGLY recommended.	STRONG	HIGH
For individuals with an intermediate calculated risk, using risk enhancers to reclassify the risk is STRONGLY recommended, regardless of age group.	STRONG	HIGH
For individuals with low calculated risk or aged 18-30 years, using risk enhancers may be applied to reclassify the risk.	STRONG	LOW
For individuals initially classified as intermediate risk, aged > 40 years and LDL-c between 70-159 mg/dL, the CAC score may be useful to determine the need and intensity of lipid-lowering therapy.	STRONG	MODERATE
For individuals initially classified as low risk, aged > 40 years and LDL-c between 70–159 mg/dL, the CAC score is reasonable for those with a family history of premature ASCVD to determine the need and intensity of lipid-lowering therapy.	CONDITIONAL	MODERATE

ASCVD: atherosclerotic cardiovascular disease; AU: Agatston units; CAC: coronary artery calcium; CVD: cardiovascular disease; LDL-c: low-density lipoprotein cholesterol; PREVENT: Predicting Risk of cardiovascular disease EVENTs.

**Table:** Recommendations for lipid therapeutic targets according to cardiovascular risk

Recommendation	Strength of Recommendation	Certainty of Evidence
In individuals at extreme CV risk, LDL-c < 40 mg/dL and non-HDL-c < 70 mg/dL targets are recommended.	STRONG	MODERATE
In individuals at very high CV risk, LDL-c < 50 mg/dL and non-HDL-c < 80 mg/dL targets are recommended.	STRONG	HIGH
In individuals at high CV risk, LDL-c < 70 mg/dL and non-HDL-c < 100 mg/dL targets are recommended.	STRONG	HIGH
In individuals at intermediate CV risk, LDL-c < 100 mg/dL and non-HDL-c < 130 mg/dL targets are recommended.	STRONG	HIGH
In individuals at low CV risk, LDL-c < 115 mg/dL and non-HDL-c < 145 mg/dL targets are recommended.	STRONG	MODERATE
In individuals at high, very high, or extreme CV risk, a ≥ 50% decrease in LDL-c is recommended.	STRONG	HIGH
In individuals at low or intermediate CV risk, a ≥ 30% decrease in LDL-cis recommended.	STRONG	HIGH
In all individuals, especially those with LDL-c or non-HDL-c above the target, lifestyle interventions are recommended.	STRONG	HIGH
In individuals at high, very high, or extreme CV risk, pharmacological therapy combined with lifestyle modifications is recommended.	STRONG	HIGH
In individuals at high, very high, or extreme CV risk with persistently increased LDL-c or non-HDL-c, intensification of pharmacological therapy combined with lifestyle modifications is recommended.	STRONG	HIGH
In individuals at low or intermediate CV risk with LDL-c or non-HDL-c persistently ≥ 30 mg/dL above target, initiation or intensification of pharmacological therapy combined with lifestyle modifications is recommended.	STRONG	HIGH
In individuals with LDL-c and non-HDL-c levels within target, considering an ApoB target to decide on therapeutic intensification is recommended.	STRONG	MODERATE

ApoB: apolipoprotein B; CV: cardiovascular; HDL-c: high-density lipoprotein cholesterol; LDL-c: low-density lipoprotein cholesterol.

**Table:** Dietary Recommendations for the treatment of dyslipidemia

Recommendation	Percentage of Total Caloric Value	Strength of Recommendation	Certainty of Evidence
Total fat	20-35%	STRONG	HIGH
Saturated fat	< 7%	STRONG	HIGH
Trans fat	Do not consume	STRONG	HIGH
Monounsaturated fatty acids	15%	STRONG	HIGH
Polyunsaturated fatty acids	5-10%	STRONG	HIGH
Fiber	25 g/day	CONDITIONAL	MODERATE
Total carbohydrates	50-55%	STRONG	HIGH

**Table:** Recommendations on dietary supplements, functional foods, and lifestyle measures in the management of dyslipidemias

Recommendation	Strength of Recommendation	Certainty of Evidence
In smokers, smoking cessation is recommended to reduce CV risk.	STRONG	HIGH
Spirituality and religiosity should be addressed in medical consultations due to their positive impact on CV health.	CONDITIONAL	MODERATE
In individuals with overweight or obesity, weight loss through nonpharmacological measures is recommended to increase HDL-c, lower TG, and moderately decrease LDL-c.	STRONG	HIGH
Physical activity is recommended to reduce CV risk and improve lipid profile, including increased HDL-c and decreased TG.	STRONG	HIGH
All adults should engage in at least 150 minutes/week of moderate-intensity aerobic activity or 75 minutes/week of vigorous activity, which can be combined for greater benefit.	STRONG	HIGH
Alcohol consumption with the goal of preventing or treating atherosclerosis is not recommended.	STRONG	MODERATE
Dietary supplementation should be considered as part of the management of dyslipidemia.	CONDITIONAL	MODERATE

* Supplements with proven* LDL-c impact include RYR, probiotics, phytosterols. For TG decrease: fish oil (EPA/DHA). CV: cardiovascular; DHA: docosahexaenoic acid; EPA: eicosapentaenoic acid; HDL-c: high-density lipoprotein cholesterol; LDL-c: low-density lipoprotein cholesterol; RYR: red yeast rice; TG: triglycerides.

**Table:** Pharmacological recommendations for the treatment of dyslipidemia

Recommendation	Strength of Recommendation	Certainty of Evidence
In individuals eligible for lipid-lowering therapy, statins are recommended as the first-line treatment option.	STRONG	HIGH
In individuals with an LDL-c reduction target of ≥ 50%, combination therapy with a high-intensity statin and ezetimibe is recommended as an alternative.	STRONG	HIGH
In individuals who do not reach their target despite maximum tolerated statin therapy, treatment intensification with ezetimibe or anti-PCSK9 therapy is recommended.	STRONG	HIGH
Inclisiran is recommended as an alternative to monoclonal anti-PCSK9 antibodies, such as evolocumab or alirocumab.	STRONG	MODERATE
In individuals intolerant to statins and not at goal despite ezetimibe, therapeutic intensification with BPA is recommended.	STRONG	HIGH
In individuals diagnosed with homozygous familial hypercholesterolemia who do not achieve LDL-c targets despite receiving the maximum tolerated doses of lipid-lowering therapies, the use of evinacumab is recommended from the age of 5 years.	STRONG	MODERATE
Pharmacologic treatment aimed at increasing HDL-c is not recommended.	STRONG	HIGH
In individuals with HTG (≥ 150 mg/dL) eligible for lipid-lowering therapy to reduce CV risk, statins are recommended as the preferred first-line treatment.	STRONG	HIGH
In individuals with triglycerides between 150 and 499 mg/dL and established ASCVD or at high cardiovascular risk, the use of IPE (4 g/day) is weakly/conditionally recommended to reduce major CV events, although it is not available in Brazil.	CONDITIONAL	MODERATE
In individuals with TG between 150 and 499 mg/dL who have ASCVD or are at high CV risk, EPA plus DHA formulations are not recommended for preventing CV events.	STRONG	HIGH
In individuals with persistently elevated TG (≥ 500 mg/dL) despite lifestyle interventions, the use of fibrates is recommended to reduce the risk of pancreatitis.	STRONG	MODERATE
In adults with FCS and triglyceride levels ≥ 500 mg/dL, the use of volanesorsen is recommended to reduce the risk of pancreatitis.	STRONG	MODERATE

ASCVD: atherosclerotic cardiovascular disease; BPA: bempedoic acid; CV: cardiovascular; IPE: icosapent ethyl; TG: triglycerides; LDL-c; FCS: familial chylomicronemia syndrome.

**Table:** Recommendations for the combined use of statin, ezetimibe, bempedoic acid, and anti-PCSK9 therapies

Recommendation	Strength of Recommendation	Certainty of Evidence
In individuals at high CV risk, initial therapy with high-intensity statin and ezetimibe is recommended to achieve therapeutic targets.	STRONG	HIGH
In individuals at very high CV risk, initial therapy with high-intensity statin and ezetimibe, and potentially PCSK9 inhibitor therapy, is recommended to achieve therapeutic targets.	STRONG	HIGH
In individuals at extreme CV risk, initial therapy with high-intensity statin, ezetimibe, and PCSK9 inhibitor therapy is recommended to achieve therapeutic targets.	STRONG	HIGH
In individuals who do not reach therapeutic targets with high-intensity statin and ezetimibe, PCSK9 inhibitor therapy and/or BPA are recommended according to treatment goals.	STRONG	HIGH
In individuals with FH and LDL-c ≥ 190 mg/dL, initial therapy with high-intensity statin plus ezetimibe is recommended to achieve therapeutic targets.	STRONG	HIGH
In individuals with statin intolerance, a personalized combination strategy is recommended (eg, BPA combined with a PCSK9 inhibitor or with ezetimibe) to achieve therapeutic targets.	STRONG	HIGH

BPA: bempedoic acid; CV: cardiovascular; FH: familial hypercholesterolemia; PCSK9: proprotein convertase subtilisin/kexin type 9.

**Table:** Recommendations for the management of statin-associated muscle symptoms

Recommendation	Strength of Recommendation	Certainty of Evidence
In patients for whom statin therapy is being considered, baseline measurement of CK and liver enzymes (ALT and AST) is recommended, especially in individuals at high risk for muscle or hepatotoxic events.	CONDITIONAL	LOW
In patients on statins, routine measurement of CK and liver enzymes is recommended in the absence of muscle symptoms, signs of hepatotoxicity, or therapy-related abnormalities.	STRONG	MODERATE
In patients on statins, measurement of CK is recommended in the presence of severe muscle symptoms, and liver enzyme testing is recommended when signs of hepatotoxicity are present.	STRONG	MODERATE
In patients who do not tolerate the suggested statin dose, alternative strategies are recommended to achieve LDL-c reduction goals, including lowering the administration frequency, switching to another statin, or combining with other lipid-lowering agents.	STRONG	HIGH
In patients for whom statin therapy is discontinued, immediate initiation of non-statin lipid-lowering therapy (eg, ezetimibe, BPA, or PCSK9 inhibitors) is recommended, either as a bridge or permanently, with the goal of mitigating CV risk due to elevated LDL-c.	STRONG	HIGH
In patients on statins, vitamin D supplementation is recommended to help mitigate muscle symptoms associated with statin use.	STRONG	HIGH
In patients on statins, routine supplementation with coenzyme Q10 is recommended to help mitigate muscle symptoms associated with statin use.	STRONG	MODERATE

ALT: alanine transaminase; AST: aspartate aminotransferase; BPA: bempedoic acid; CK: creatine kinase; CV: cardiovascular; LDL-c: low-density lipoprotein cholesterol; PCSK9: proprotein convertase subtilisin/kexin type 9.

**Table:** Recommendations for the use of lipid-lowering therapies in patients with heart failure

Recommendation	Strength of Recommendation	Certainty of Evidence
Patients with HF and established ASCVD should continue statin therapy to reduce the risk of ASCVD events.	STRONG	MODERATE
In patients with HF who were already on statin therapy, discontinuation is not recommended if the patient has a clinically acceptable life expectancy.	STRONG	MODERATE
Patients with HFrEF and no ASCVD may use statins when no contraindications are present, provided treatment is individualized.	CONDITIONAL	MODERATE
PCSK9 inhibitors should be maintained in patients with HF at high risk when LDL-c targets are not achieved with statins and ezetimibe.	STRONG	MODERATE

ASCVD: atherosclerotic cardiovascular disease; HF: heart failure; HFrEF: HF with reduced ejection fraction; LDL-c: low-density lipoprotein cholesterol; PCSK9: proprotein convertase subtilisin/kexin type 9.

**Table:** Recommendations for the management of dyslipidemia in people living with HIV

Recommendation	Strength of Recommendation	Certainty of Evidence
In PLWH, statins should be considered as first-line therapy for LDL-c reduction and cardiovascular risk management, according to the relevant target. The choice of statin should take into account the risk of drug–drug interactions.	STRONG	HIGH
In PLWH with statin intolerance or insufficient LDL-c reduction, ezetimibe should be added.	STRONG	MODERATE
In PLWH with high cardiovascular risk and inadequate LDL-c control despite maximally tolerated therapy, or in cases of statin intolerance, PCSK9 inhibitors such as evolocumab should be considered.	STRONG	MODERATE

LDL-c: low-density lipoprotein cholesterol; PCSK9: proprotein convertase subtilisin/kexin type 9; PLWH: people living with HIV.

**Table:** Recommendations for the management of dyslipidemia in people with diabetes

Recommendation	Strength of Recommendation	Certainty of Evidence
For individuals with diabetes, statins are the first-line lipid-lowering therapy for patients with LDL-c levels above the established target.	STRONG	HIGH
For individuals with diabetes, ezetimibe may be used in those who remain above the LDL-c target despite maximally tolerated statin therapy.	STRONG	MODERATE
For individuals with diabetes, PCSK9 inhibitors may be used in those who remain above the LDL-c target despite maximally tolerated statin and ezetimibe therapy.	STRONG	MODERATE
For individuals with diabetes and mild retinopathy, fenofibrate may be used to reduce the progression of diabetic retinopathy.	STRONG	MODERATE

LDL-c: low-density lipoprotein cholesterol; PCSK9: proprotein convertase subtilisin/kexin type 9.

**Table:** Recommendations for the management of dyslipidemia in patients with hypothyroidism

Recommendation	Strength of Recommendation	Certainty of Evidence
In patients with clinical hypothyroidism and dyslipidemia, hormone replacement therapy with levothyroxine is recommended to normalize TSH and improve the lipid profile.	STRONG	HIGH
In patients with clinical hypothyroidism and dyslipidemia, the use of statins is recommended in those whose dyslipidemia persists after normalization of thyroid function, particularly in the presence of high CV risk.	STRONG	MODERATE
In patients with subclinical hypothyroidism and dyslipidemia, levothyroxine therapy is recommended when TSH is between 4.5 and 9.9 mIU/L and the patient presents with hypothyroidism symptoms or high CV risk.	STRONG	MODERATE
In patients with subclinical hypothyroidism and dyslipidemia, levothyroxine therapy is recommended when TSH exceeds 10 mIU/L.	STRONG	HIGH

CV: cardiovascular; TSH: thyroid-stimulating hormone.

**Table:** Recommendations for the management of dyslipidemia in patients with chronic kidney disease

Recommendation	Strength of Recommendation	Certainty of Evidence
In individuals with CKD stages 1-3 and increased CV risk, high-intensity statins are recommended to reduce CV risk.	STRONG	HIGH
In individuals with CKD stages 1-3 and increased CV risk who have not achieved targets, the addition of ezetimibe to high-intensity statins is recommended.	STRONG	MODERATE
In individuals with CKD stages 4-5 not on dialysis, initiation of high-intensity statins, with or without ezetimibe, is recommended.	STRONG	HIGH
In individuals with CKD on dialysis and no established CVD, statin initiation is not recommended.	STRONG	HIGH

CKD: chronic kidney disease; CV: Cardiovascular.

**Table:** Recommendations for the management of dyslipidemia in patients with obesity

Recommendation	Strength of Recommendation	Certainty of Evidence
In individuals with dyslipidemia secondary to obesity, nonpharmacological interventions are recommended as first-line treatment.	STRONG	HIGH
In individuals with dyslipidemia secondary to obesity, statins are recommended as the foundation of pharmacological treatment.	STRONG	HIGH
In individuals with dyslipidemia secondary to obesity, fibrates are recommended when TG are ≥ 500 mg/dL to reduce the risk of pancreatitis.	STRONG	MODERATE
In individuals with dyslipidemia secondary to obesity, fibrates are not recommended for CV risk decrease or for pancreatitis prevention when TG are ≤ 500 mg/dL.	STRONG	HIGH
In individuals with dyslipidemia secondary to obesity, GLP-1 receptor agonists are recommended for their dual effect on weight loss and reduction of cardiovascular events.	STRONG	HIGH
In individuals with dyslipidemia secondary to obesity, bariatric surgery is recommended to improve lipid profile and reduce CV events.	STRONG	MODERATE

CV: cardiovascular; GLP-1: glucagon-like peptide-1; TG: triglycerides.

**Table:** Recommendations for the pharmacological treatment of dyslipidemia in older adults

Recommendation	Strength of Recommendation	Certainty of Evidence
After 75 years of age, it is recommended to individualize the doses of lipid-lowering agents according to frailty, presence of comorbidities, life expectancy, and the use of polypharmacy.	STRONG	MODERATE

**Table:** Recommendations for the management of dyslipidemia in children

Recommendation	Strength of Recommendation	Certainty of Evidence
For the pediatric population, a complete lipid profile screening is recommended universally between 9 and 11 years of age.	STRONG	MODERATE
For the pediatric population with risk factors (mentioned in the text), a complete lipid profile screening is recommended starting at 2 years of age.	STRONG	MODERATE
For the pediatric population, lifestyle modification with nutritional guidance, weight control, and physical activity is strongly recommended as the first therapeutic approach when there is compatible age and clinical judgment.	STRONG	HIGH
For the pediatric population who do not reach LDL-c targets after lifestyle modification, monotherapy with statins is recommended starting at 8 years of age.	STRONG	MODERATE
For the pediatric population who remain above LDL-c targets despite lifestyle modification, the use of ezetimibe is recommended starting at 6 years of age, and combination therapy with statins is recommended starting at 8 years of age.	CONDITIONAL	LOW
For the pediatric population with clinical evaluation indicating high risk, based on LDL-c levels and patient condition, consider using evolocumab from age 10 or alirocumab from age 8.	STRONG	MODERATE
For pediatric patients with a diagnosis of homozygous familial hypercholesterolemia, the use of evinacumab is recommended from 5 years of age in those who do not achieve LDL-c targets despite maximally tolerated lipid-lowering therapy.	STRONG	MODERATE

LDL-c: low-density lipoprotein cholesterol.

**Table:** Recommendations for the management of dyslipidemia in transplant recipients

Recommendation	Strength of Recommendation	Certainty of Evidence
It is recommended to consider all transplant recipients as having an increased CV risk.	STRONG	MODERATE
For all transplant recipients, lipid profiling is recommended about 2 to 3 months after transplantation.	STRONG	MODERATE
In transplant recipients, statins are recommended as the first-line treatment for dyslipidemia to reduce CV events.	STRONG	HIGH
In transplant recipients, it is not recommended against using simvastatin or lovastatin in combination with cyclosporine, tacrolimus, sirolimus, or everolimus.	STRONG	HIGH
In transplant recipients on cyclosporine, it is recommended to use a maximum dose of 5 mg rosuvastatin or 10 mg atorvastatin to avoid drug interactions.	STRONG	MODERATE
In transplant recipients on immunosuppressants, it is recommended to use statins with lower risk of rhabdomyolysis, such as pravastatin, fluvastatin, or rosuvastatin.	STRONG	MODERATE

CV: cardiovascular.

**Table:** Recommendations for the management of dyslipidemia in patients with chronic liver diseases

Recommendation	Strength of Recommendation	Certainty of Evidence
Statin use may be indicated for patients with hepatic steatosis and increased liver enzymes (up to 3× ULN) to reduce CV risk.	STRONG	HIGH
Statin use may be indicated for patients with hepatic steatosis and increased liver enzymes (up to 3× ULN) to improve liver outcomes.	STRONG	MODERATE
Compensated chronic liver disease (Child-Pugh A and B) is not an absolute contraindication for initiating or maintaining statin or anti-PCSK9 therapy.	STRONG	MODERATE
In patients with advanced liver failure (Child-Pugh B and C), the use of ezetimibe is not recommended.	CONDITIONAL	MODERATE

CV: cardiovascular; PCSK9: proprotein convertase subtilisin/kexin type 9; ULN: upper limit of normal.

**Table:** Recommendations for laboratory diagnosis and treatment of dyslipidemia in patients with acute coronary syndrome

Recommendation	Strength of Recommendation	Certainty of Evidence
In patients with ACS, early lipid profile testing (preferably within 24 hours of the acute event) is recommended as a basis for therapeutic decisions.	STRONG	MODERATE
In patients with ACS, lipid profile testing is recommended within 4-6 weeks after hospital discharge.	STRONG	MODERATE
In patients with ACS, initiation of high-intensity statins within the first 24 hours of hospitalization is recommended.	STRONG	HIGH
In patients with ACS, it is recommended to initiate high-intensity statins plus ezetimibe during the acute phase, and to consider early use of PCSK9 inhibitors in patients at very high or extreme risk, as an intensive strategy to rapidly reduce LDL-c, minimize therapeutic inertia, and increase the likelihood of achieving lipid goals.	STRONG	MODERATE

ACS: acute coronary syndrome; LDL-c: low-density lipoprotein cholesterol; PCSK9: proprotein convertase subtilisin/kexin type 9.

**Table:** Recommendations for the management of dyslipidemia in patients with immune-mediated diseases

Recommendation	Strength of Recommendation	Certainty of Evidence
In individuals with RA who meet high-risk criteria for immune-mediated disease, it is recommended to consider this an enhancing factor for CV risk.	STRONG	MODERATE
In individuals with immune-mediated diseases, it is recommended to adequately control inflammatory activity as an essential strategy to decrease CV risk.	STRONG	MODERATE
In individuals with immune-mediated diseases, it is recommended to use CAC score for risk stratification in patients with intermediate risk.	CONDITIONAL	MODERATE
In individuals with immune-mediated diseases, it is recommended to use statins as the first-line treatment for dyslipidemia.	STRONG	HIGH
In individuals with immune-mediated diseases: when statins are not tolerated or the response is inadequate, it is recommended to add ezetimibe or, in very high-risk cases, PCSK9 inhibitors.	STRONG	MODERATE

CAC: coronary artery calcium; CV: cardiovascular; RA: rheumatoid arthritis; PCSK9: proprotein convertase subtilisin/kexin type 9.

**Table:** Recommendations for the management of dyslipidemia in pregnant women

Recommendation	Strength of Recommendation	Certainty of Evidence
For pregnant women with dyslipidemia related to pregnancy or other forms of primary or secondary dyslipidemia, it is recommended to follow a low-fat diet, high in soluble fiber and low glycemic index carbohydrates.	STRONG	MODERATE
For women planning to become pregnant and previously using statins, it is recommended to discontinue statin therapy 60 days before conception.	STRONG	MODERATE
For pregnant women using statins, immediate discontinuation of the drug is recommended; it should only be restarted after the breastfeeding period.	STRONG	MODERATE
For pregnant women at very high risk, therapeutic individualization and shared decision-making are recommended, including the possible reintroduction of statins in the third trimester.	CONDITIONAL	MODERATE
For pregnant women with hypercholesterolemia, the use of bile acid sequestrants is recommended.	CONDITIONAL	LOW
For pregnant and breastfeeding women, it is recommended to avoid the use of ezetimibe, anti-PCSK9 therapies, ANGPTL3 inhibitors (eg, evinacumab), BPA, and lomitapide.	STRONG	MODERATE
For pregnant women with TG > 880 mg/dL despite lifestyle changes, the use of fenofibrate during the second trimester is recommended.	CONDITIONAL	LOW
For pregnant women with TG > 880 mg/dL despite lifestyle changes, the use of omega-3 fatty acids is recommended.	CONDITIONAL	LOW

BPA: bempedoic acid; PCSK9: proprotein convertase subtilisin/kexin type 9; TG: triglycerides.

**Table:** Recommendations for the management of dyslipidemia in women according to cardiovascular risk

Recommendation	Strength of Recommendation	Certainty of Evidence
For women classified as low or intermediate CV risk, the use of clinical risk enhancers is recommended to refine risk stratification and guide more intensive therapeutic decisions	STRONG	MODERATE
For women classified as high, very high, or extreme risk, intensive and combination therapy is recommended	STRONG	HIGH

CV: cardiovascular.

## 2. Epidemiology

### 2.1. Average Plasma Lipid Levels and Prevalence of Dyslipidemia

Epidemiological data on dyslipidemias in Brazil can be drawn from population-based surveys and observational studies. In the 2014-2015 Brazilian National Health Survey (PNS), blood samples were collected from a subpopulation of 8,534 adults to estimate representative lipid profile values for the Brazilian population. The mean total cholesterol and LDL-c levels were 185 mg/dL and 105 mg/dL, respectively, with slightly higher values observed in women compared to men ( Figure 2.1 ). One in three individuals had total cholesterol > 200 mg/dL and one in five had LDL-c > 130 mg/dL, with higher prevalence among women ( Figure 2.2 ). Lower levels of total cholesterol and LDL-c were found in individuals aged 18-29, intermediate levels in those aged 30-44, and the highest levels in those aged 45 and older. Increased total cholesterol and LDL-c were more prevalent among individuals with lower educational attainment.^11 , 15^

**Figure 2.1:** Blood lipid levels in the Brazilian population stratified by sex. Mean levels of TC, LDL-c, and HDL-c, and median TG levels from the 2014-2015 PNS and the baseline of the ELSA-Brasil study. Sources: 2014-2015 PNS^15^and ELSA-Brasil database. ELSA-Brasil: Brazilian Longitudinal Study of Adult Health; HDL-c: high-density lipoprotein cholesterol; LDL-c: low-density lipoprotein cholesterol; PNS: National Health Survey; TC: total cholesterol; TG: triglycerides.

**Figure 2.2:** Lipid abnormalities in the Brazilian population stratified by sex. Prevalence of lipid abnormalities according to the 2014-2015 PNS, the 2019 PNS, the 2016 Vigitel, and the baseline of the ELSA-Brasil study. ELSA-Brasil: Brazilian Longitudinal Study of Adult Health; HDL-c: high-density lipoprotein cholesterol; LDL-c: low-density lipoprotein cholesterol; PNS: National Health Survey; TC: total cholesterol; TG: triglycerides; Vigitel: Brazilian Telephone Survey for Surveillance of Risk and Protection Factors for Chronic Diseases. Sources: 2014-2015 PNS,^15^2019 PNS,^16^2016 Vigitel,^17^and ELSA-Brasil database.

Compared to the 2014-2015 PNS, lower rates of self-reported dyslipidemia were observed in the 2019 PNS^16^(a nationally representative study involving 88,531 adults) and in the 2016 Brazilian Telephone Survey for Surveillance of Risk and Protection Factors for Chronic Diseases^17^(Vigitel) — based on 53,210 adult interviews in the capital cities of Brazil's 26 states and the Federal District ( Figure 2.2 ).

Another source of lipid data in the Brazilian population is the Brazilian Longitudinal Study of Adult Health (ELSA-Brasil), a prospective cohort of 15,105 public servants initially aged 35 to 74 years (mean age 52 ± 9 years) from six capital cities (Salvador, Belo Horizonte, Vitória, Rio de Janeiro, São Paulo, and Porto Alegre).^18^Compared to the 2014-2015 PNS, the ELSA-Brasil baseline (2008-2010) revealed higher levels of total cholesterol, LDL-c, and high-density lipoprotein cholesterol (HDL-c) as well as substantially greater prevalence of increased total cholesterol or LDL-c ( Figure 2.1 and Figure 2.2 ). Differences in population characteristics may account for such discrepancies.

ELSA-Brasil was also used to estimate the prevalence of FH based on the Dutch Lipid Clinic Network criteria, stratified by sex and skin color. The overall prevalence was 3.8 per 1,000 individuals, equivalent to 1 in every 263. This rate was higher in women (1:244) than in men (1:333), and higher among individuals identifying as *Pardo* (mixed-race) (1:204) and Black (1:156), compared to those identifying as White (1:417).^19^

Epidemiological information on dyslipidemias in children and adolescents was provided by ERICA (Study of Cardiovascular Risk in Adolescents), a nationwide, school-based survey involving 38,069 adolescents aged 12 to 17 years living in all 27 Brazilian state capital cities or surrounding areas, conducted between 2013 and 2014. The main results are shown in Figure 2.3 . One in five adolescents had total cholesterol ≥ 170 mg/dL, with higher prevalence among women than men.^20^

**Figure 2.3:** Blood lipids in children and adolescents in Brazil stratified by sex, according to data from ERICA. A. Mean levels of TC, LDL-c, HDL-c, and TG. B. Prevalence of lipid abnormalities. Source: ERICA study.20 ERICA: Study of Cardiovascular Risk in Adolescents; HDL-c: high-density lipoprotein cholesterol; LDL-c: low-density lipoprotein cholesterol; TC: total cholesterol; TG: triglycerides.

### 2.2. Cardiovascular Mortality Attributable to Increased Low-density Lipoprotein Cholesterol

According to data from the GBD study, increased LDL-c is the second leading risk factor for CV deaths in Brazil, following hypertension. The age-standardized CV mortality rate attributable to increased LDL-c in Brazil decreased from 49.6 to 32.1 per 100,000 inhabitants between 2001 and 2021.^1^This reduction reflects advances in risk factor control, improved access to health care services, and better quality of care. However, considering population growth over the same period, the estimated absolute number of CV deaths attributable to increased LDL-c increased from 60,716 to 79,604,^1^underscoring the epidemiological relevance of hypercholesterolemia.

### 2.3. Data on Treatment and Target Achievement

There is a lack of data on lipid-lowering treatment and target attainment in Brazil, which makes it difficult to accurately define the national dyslipidemia treatment landscape. ELSA-Brasil showed that the country was far from the ideal scenario between 2008 and 2010: among participants with increased LDL-c, 42.3% were using lipid-lowering therapy, and only 58.3% achieved the goals recommended by the National Cholesterol Education Program Adult Treatment Panel III (NCEP-ATP III) guidelines.^21^The 2006-2007 L-TAP 2 (Lipid Treatment Assessment Project 2) study, which assessed lipid management in nine countries including Brazil (n = 391), found a similar proportion (62.1%) of Brazilians within LDL-c targets.^22^A particularly concerning scenario emerges when focusing on individuals with severe dyslipidemia (LDL-c > 190 mg/dL): in a Brazilian private institution study with data from 2004 to 2019, only 5.9% of these patients initially received lipid-lowering medication. This increased to 45.4% by the end of follow-up, but only 19.1% achieved an LDL-c reduction greater than 50%.^23^

More recent evidence on dyslipidemia management in Brazil was reported by the NEtwork to Control Atherothrombosis (NEAT) registry, which involved 25 centers (56% public) across all five regions of the country. The study assessed 2,003 patients with coronary and/or PAD between 2020 and 2022. About 5.1% were not on statin therapy, and among those who were, 55.4% were not on high-intensity therapy. Among patients with available LDL-c measurements, only 14.4% had LDL-c levels below 50 mg/dL, while approximately 30% had levels ≥ 100 mg/dL ( Figure 2.4 ). The low adoption of combination therapies is also noteworthy: a total of 6.19% of patients were on concomitant high-intensity statin and ezetimibe therapy, and only 1 patient (0.05%) was receiving triple therapy with high-intensity statin, ezetimibe, and a PCSK9 inhibitor, as illustrated in Figure 2.5 .^24^The main barrier to evidence-based therapy was not the cost, but rather physicians’ decisions not to prescribe these strategies.^25^

**Figure 2.4:** Distribution of LDL-cholesterol (LDL-c) among treated patients according to therapeutic groups in the NEAT Study population with available LDL-c (n = 1035): LDL-cholesterol range n (%). *Patients without treatment (n = 36) and those on anti-PCSK9 therapy (n = 2) were excluded from the analysis.

**Figure 2.5:** Distribution of therapeutic groups among patients with available LDL (n = 2003): therapeutic group n (%). *1 patient in the group without statin use and with ezetimibe was receiving anti-PCSK9 therapy.

Alarming results were reported in a real-world cross-sectional study within Brazil's Family Health Strategy program, using data from 2016 to 2021. Among more than 35,000 adults with a history of MI or stroke, only 6.7% and 0.6% were on statins and high-dose statins, respectively.^26^

Outside Brazil, LDL-c target attainment is also far from optimal. The DA VINCI (EU-Wi *d* e Cross-Section *a* l Obser *v* at *i* o *n* al Study of Lipid-Modifying Therapy Use in Se *c* ondary and Pr *i* mary Care) study assessed goal achievement in primary and secondary prevention patients prescribed lipid-lowering therapy (94% on statins). Data were collected from 2017 to 2018 across 18 European countries. Only 33% achieved the targets set by the 2019 European guideline (54% according to the 2016 guideline), in the context of low use of high-intensity statins (22% in primary prevention and 42% in secondary prevention). Additionally, combination therapy rates were very low: 9% with ezetimibe and 1% with a PCSK9 inhibitor.^27^

In the multicenter Getting to an imprOved Understanding of Low-Density Lipoprotein Cholesterol and Dyslipidemia Management (GOULD) registry in the United States (2016-2020), among secondary prevention patients not using PCSK9 inhibitors, lipid-lowering therapy was intensified in only 22% of those with LDL-c ≥ 100 mg/dL and in 14% of those with LDL-c between 70 and 99 mg/dL over a 2-year period. LDL-c levels < 70 mg/dL were achieved by 21% and 34% of patients in the respective groups. By the end of the study, ezetimibe had been added to only 5.3% of patients.^28^

The PURE (Prospective Urban Rural Epidemiology) study clearly illustrates the impact of socioeconomic conditions on the use of statins. Among participants in secondary prevention, statins were used by only 3.3% of individuals in low-income countries, 4.3% in lower-middle-income countries, 17.6% in upper-middle-income countries, and 66.5% in high-income countries.^29^Another study conducted between 2013 and 2019 with over 116,000 individuals (more than 9,000 with a history of CVD) across 41 low- and middle-income countries showed statin use in only 8% of eligible individuals in primary prevention and 22% in secondary prevention.^30^

Simvastatin is widely available in Brazil's public Unified Health System (SUS), while atorvastatin is only accessible through costly programs, and ezetimibe is offered at select cardiology centers. PCSK9 inhibitors are accessible only through out-of-pocket payments by patients. Barriers such as these hinder the implementation of combination lipid-lowering therapy and the achievement of LDL-c targets in the general population.^31^Furthermore, follow-up testing is often not routinely performed, whether for safety monitoring or lipid profiling.^32^This compromises the early identification of rare adverse events and undermines guideline-based recommendations for goal-directed lipid management, ultimately reducing the quality of care.^33^

Therefore, despite having demonstrated efficacy and safety for over 30 years, including reducing CV mortality in both primary and secondary prevention studies, statins are not being used as much as expected. Furthermore, cholesterol control and achievement of guideline-recommended targets are inadequate across many regions worldwide. Brazil is a country with significant regional income disparities. Increasing the use of statins among eligible individuals and consequently raising the proportion of people who reach lipid targets poses a major public health challenge there. Nevertheless, effective action must be taken to address this issue and lessen the societal impact of CVD.

## 3. Diagnosis

### 3.1. Laboratory Assessment of Lipid Parameters and Apolipoproteins

#### 3.1.1. Pre-Analytical and Analytical Phases

Lipids circulate in the bloodstream bound to specific proteins, forming complexes known as lipoproteins. The accuracy of lipoprotein measurement depends on two stages of the laboratory process: the pre-analytical phase and the analytical phase. The pre-analytical phase involves collection procedures, patient instructions, sample transport, and preparation. This phase also considers intrinsic patient-related factors, such as lifestyle, use of drugs, and comorbidities. The analytical phase refers to the methods and procedures employed by laboratories.^34 , 35^

##### 3.1.1.1. Pre-Analytical Phase

This phase includes all procedures performed before the patient's sample is processed by laboratory equipment.

- **Biological variation:** Lipoprotein levels can fluctuate over time, known as intra-individual biological variation.^36^
- **Tourniquet use during venipuncture** : Hemoconcentration may occur within 1 minute of applying a tourniquet, which can potentially alter the lipid profile. To minimize this effect, release the tourniquet as soon as the needle enters the vein.^37^
- **Patient preparation for sample collection** : When preparing for lipid profile testing, it is recommended that patients maintain a stable metabolic state and their usual diet. Deviation from the usual diet (eg, alcohol intake or high-fat meals), as well as strenuous physical activity on the day before the test, may temporarily alter lipid levels. Several guidelines indicate that fasting is not necessary for initial lipid testing. Triglycerides (TG) levels are the most likely to be affected in nonfasting samples. Increased TG, in turn, may reduce the accuracy of LDL-c calculated using the Friedewald formula, but this can be mitigated by using the Martin/Hopkins equation.^38^

Laboratories should adapt their protocols to allow for flexible fasting durations, while always respecting the referring physician's instructions. Test reports should specify whether the sample was collected in a fasting or 12-hour fasting state, according to the physician's request. In some specific clinical situations (e.g., familial genetic hyperlipidemia), in which the TG concentration is very high (> 440 mg/dL), a repeat lipid profile after a 12-hour fast should be requested. Physicians should interpret lipid profile results in light of the indication for testing, the patient's metabolic state, and overall risk stratification.^39^

**Table:** 

Recommendation	Strength of Recommendation	Certainty of Evidence
Ideally, the lipid profile sample should be collected under stable metabolic conditions.	STRONG	MODERATE
For initial assessment, nonfasting samples are acceptable, particularly in selected populations such as children and older adults.	STRONG	MODERATE
If TG levels are increased (> 440 mg/dL) in a nonfasting sample, a repeat 12-hour fasting sample is recommended, according to the referring physician's discretion.	STRONG	MODERATE

TG: triglycerides.

##### 3.1.1.2. Analytical Phase

Several methods are available and routinely used in clinical laboratories. Others are restricted to research and are rarely applied in clinical practice due to low throughput and/or high cost.

###### 3.1.1.2.1. Research-Restricted Methods

- **Ultracentrifugation:** ^40^this is the reference method for separating different lipoproteins, based on the particles’ buoyancy properties relative to their equilibrium density under high gravitational force. Ultracentrifugation allows for the separation of most lipoproteins: LDL, intermediate-density lipoprotein (IDL), Lp(a), HDL, very LDL (VLDL), and chylomicrons. Despite its strengths, this method is not suitable for routine laboratory use due to its high cost and time-consuming nature and is thus limited to research protocols.
- **Nuclear magnetic resonance (NMR) spectroscopy:** the expression "atherogenic particle count by nuclear magnetic resonance spectroscopy" refers to the quantification of potentially atherogenic lipoprotein particles — primarily LDL, but also VLDL, IDL, and Lp(a) using NMR spectroscopy.^41 - 43^This method is recognized and validated in the medical literature for assessment of CV risk, as the concentration of atherogenic particles (especially small, dense LDL) is more strongly associated with CV outcomes than simple LDL-c levels. NMR enables direct quantification of lipoprotein particle numbers in plasma, based on the physical and chemical properties of lipids and apolipoproteins. The most clinically applied version is proton NMR (^1^H-NMR), which analyzes specific signals from methyl and methylene groups in lipids, allowing for determination of the number and size of lipoprotein subclasses, including LDL, VLDL, and HDL.
- **Mass spectrometry (MS):** MS is playing an increasingly important role in evaluating dyslipidemia by going beyond the traditional quantification of total cholesterol, LDL, HDL, and TG. MS-based approaches, especially lipidomics techniques, enable the detailed characterization of plasma lipid profiles and lipoprotein subclasses. This offers insights into the molecular structure of lipids and apolipoproteins across different lipoprotein fractions.^44 - 46^In a diagnostic context, MS has been used to identify lipid biomarkers associated with hypercholesterolemia. These biomarkers include specific sphingolipids, such as ceramides, and cholesterol sulfate. They have strong discriminatory power for diagnosis and CV risk stratification.^44^Additionally, MS enables the simultaneous analysis of hundreds to thousands of lipid species with high specificity, reproducibility, and robustness, even in large-scale studies. This makes MS particularly relevant for precision medicine and epidemiological research.^45^The ability to quantify regulatory proteins, such as PCSK9, at very low levels enhances MS's potential for studying lipid metabolism and the response to targeted therapies.^46^In summary, MS — through lipidomic and proteomic approaches — provides an in-depth, precise evaluation of dyslipidemia. This method has the potential to identify new biomarkers, clarify pathogenic mechanisms, and enhance CV risk stratification. Thus, it complements and expands upon conventional laboratory methodologies.^44 - 46^However, several limitations hinder its broader adoption in routine laboratory and clinical settings, including extremely high equipment costs, lack of standardized and validated protocols (often developed in-house), limited availability of trained personnel to operate the technique, and low levels of automation and integration in clinical laboratories.^47^

###### 3.1.1.2.2. Conventional Methods – Routine Laboratory Practice

####### 3.1.1.2.2.1. Colorimetric Enzymatic Methods

These are currently the most used methods in clinical laboratories for determining TC, HDL-c, and TG. A number of commercial diagnostic kits show good correlation and low coefficient of variation for TC and TG, allowing for inter-laboratory comparison using the same sample. However, for HDL-c, differences of up to 15% may be observed between available methods. These techniques remain the preferred option due to their high sensitivity and specificity, operational simplicity, low cost, and compatibility with automation in clinical laboratories. To measure LDL-c, direct colorimetric methods are available, but they show considerable variability between different diagnostic kits and are not routinely used in clinical practice. Instead, calculation formulas are most commonly employed in both clinical and laboratory settings.

####### 3.1.1.2.2.2. Point of Care Testing

The term point of care testing (POCT) is widely recognized in the global literature and refers to small devices that perform bedside testing. For lipid profile evaluation, these devices use whole blood samples obtained via capillary puncture. Many portable POCT devices are currently available and approved for use in dyslipidemia assessment. Depending on the device, they may assess TC, HDL-c, TG, and even directly measure LDL-c.^48 , 49^

The main advantage of this technology is that it allows patients to remain close to the testing site, with immediate results — especially useful in intensive care and primary health care settings. One important consideration is that different methodologies and manufacturers are involved in these small devices. Proper validation of each device is essential before clinical implementation. Validation should involve comparing results with those from a certified clinical laboratory with monitored processes (ie, an accredited laboratory).

After validation for routine use, regular quality control procedures must be implemented to ensure the reliability of test execution. Both validation and quality control are mandated by Brazil's National Health Surveillance Agency under Collegiate Board Resolution No. 978 of June 6, 2025.^50^This regulation permits testing using capillary samples in isolated clinics and mobile services (eg, community health campaigns).

Despite technological advances, POCT still shows greater analytical variability when compared to serum-based testing in clinical laboratories. Another point of concern is that the proper execution of POCT requires operator training and a thorough assessment of all procedural steps. Factors that may directly impact result accuracy include ambient temperature, relative humidity, and the volume of whole blood applied to the device.^51^

When evaluating market options for implementing lipid profiling via POCT, it is advantageous to choose equipment capable of performing a full lipid panel, including HDL-c, to enable the calculation of non-HDL-c. This parameter is essential as it allows for the evaluation of atherogenic lipoproteins and CVD risk.

Although POCT tests still carry a considerable cost, their advantages in lipid profiling are numerous — such as in FH screening, workplace health programs, community outreach in remote areas, and testing in children and older adults with difficult venous access, as well as other high-risk situations.^52^

####### 3.1.1.2.2.3. Calculation of Low-Density Lipoprotein Cholesterol

The Friedewald formula was widely used for many years to estimate LDL-c values; however, it has known limitations. Martin et al.^53^proposed an alternative method for estimating LDL-c using ultracentrifugation as a reference. Through statistical modeling, they defined different divisors for TG to more accurately estimate VLDL cholesterol levels. To obtain these divisors, patient non-HDL-c and TG levels are required. Using this new divisor (x), the formula becomes: LDL-c = TC – HDL-c – TG/x, where x ranges from 3.1 to 11.9.

Several other formulas have been described — and continue to be developed — for estimating LDL-c. In a recent publication, Samuel et al. evaluated 23 formulas cited in literature, and the Martin/Hopkins formula showed superior comparative performance.^38^

Notably, all formulas lose accuracy when TG levels are high, particularly when > 800 mg/dL.

**Table:** 

Recommendation	Strength of Recommendation	Certainty of Evidence
The use of the Martin/Hopkins equation for LDL-c calculation is recommended for all individuals.	STRONG	MODERATE
For TG values >800 mg/dL, LDL-c results using the Martin/Hopkins formula may be underestimated. Evaluation of non–HDL-c is recommended.	STRONG	MODERATE

LDL-c: low-density lipoprotein cholesterol; Non-HDL-c: non-high-density lipoprotein cholesterol; TG: triglycerides.

####### 3.1.1.2.2.4. Measurement of Non-High-Density Lipoprotein Cholesterol

Non-HDL-c represents the cholesterol fraction in plasma lipoproteins other than HDL and is calculated by subtracting HDL-c from TC: non–HDL-c = TC – HDL-c. The purpose of using non-HDL-c is to estimate the amount of atherogenic lipoproteins circulating in the plasma, particularly in individuals with increased TG.^54^

**Table:** 

Recomendação	Força da recomendação	Certeza da evidência
Both LDL-c and non-HDL-c are highly useful for assessing CV risk and as therapeutic targets. Non-HDL-c is particularly valuable for estimating the amount of circulating atherogenic lipoproteins in individuals with increased TG levels (> 150 mg/dL).	STRONG	HIGH

CV: cardiovascular LDL-c: low-density lipoprotein cholesterol; Non-HDL-c: non-HDL cholesterol; TG: triglycerides.

####### 3.1.1.2.2.5. Measurement of Apolipoprotein B

ApoB is the main structural protein present in atherogenic lipoproteins and acts as a ligand for the LDL receptor. Its measurement provides a direct estimate of the total concentration of circulating atherogenic lipid particles, since there is a single ApoB molecule in each atherogenic lipoprotein: LDL, VLDL, IDL, and Lp(a). In the general population, LDL-c and ApoB levels are highly correlated and generally provide similar information regarding the risk of ASCVD. However, in individuals with diabetes, obesity, or metabolic syndrome (MetS) — conditions often associated with increased TG — the isolated measurement of LDL-c may underestimate the total concentration of ApoB-containing lipoproteins.^55^This occurs because, in the presence of high TG levels, part of the cholesterol in LDL particles is replaced by TG, which promotes the formation of small, dense LDL particles. These more atherogenic particles make LDL-c a less reliable reflection of the actual number of LDL particles.

Approximately 20% of these patients present a discordance between measured LDL-c and ApoB levels.^56^Therefore, in the presence of increased TG, the estimation of the concentration of atherogenic particles may provide greater accuracy for assessment of CV risk. In such cases, non-HDL-c (indirectly) and ApoB (directly) provide a more precise evaluation. Non-HDL-c therefore represents the TC carried by ApoB-containing lipoproteins, while ApoB directly estimates the number of atherogenic particles in plasma. Comparative studies in population cohorts have shown that both markers are equivalent indicators of CV risk for most individuals. Analyses of CV events in the UK Biobank^57^and a meta-analysis of prospective cohort studies in individuals at risk of or with CVD have demonstrated similar risk assessment capabilities with both markers.^58^On the other hand, more recent publications suggest that a subgroup of individuals — estimated at 8%-23% — present discordant ApoB and non-HDL-c levels, with ApoB emerging as a better predictor of coronary calcification and CV events.^59^

ApoB also offers more accurate assessment of atherogenic lipoproteins in the setting of very low LDL-c. Importantly, ApoB levels are not significantly altered in the postprandial state in individuals with TG < 400 mg/dL.^60^

One limitation of the widespread use of ApoB is the lack of well-established thresholds for initiating or intensifying pharmacological therapy, compared to LDL-c or non-HDL-c levels.

**Table:** 

Recomendação	Força da recomendação	Certeza da evidência
Measurement of ApoB may help in assessing CV risk and guiding therapy in individuals with HTG (TG > 150 mg/dL).	STRONG	MODERATE
Non-HDL-c is currently a more practical option because it can be easily calculated and does not impose additional costs for the patient or the health care system.	STRONG	HIGH

ApoB: apolipoprotein B; HTG: hypertriglyceridemia; non-HDL-c: non-HDL cholesterol; TG: triglycerides.

####### 3.1.1.2.2.6. Lipoprotein(a)

Lp(a) is a particle similar to LDL in that ApoB is covalently bound to a molecule called apolipoprotein(a) [apo(a)]. In addition to its pro-atherogenic effects, Lp(a) has pro-inflammatory effects, likely related to its oxidized phospholipid content. Moreover, the structural resemblance of apolipoprotein(a) to plasminogen raises the possibility of pro-thrombotic effects.

Plasma concentrations of Lp(a) are not influenced by diet, age, sex, fasting state, or lifestyle, and are largely (> 90%) genetically determined. Individual values are generally stable throughout life; therefore, repeated measurements are not necessary for assessment of risk. Measuring Lp(a) is challenging due to variations among analytical methods, partly because of the apolipoprotein(a) structure, which can vary widely in size.

Lp(a) concentrations should preferably be measured using a method that minimizes the impact of isoform size. The recommendation is to measure the concentration of circulating particles (in nmol/L). If this is not available, measuring Lp(a) mass concentration (in mg/dL) is acceptable.^61^The current guideline does not recommend converting mass units to molar units due to poor accuracy.

An Lp(a) concentration > 50 mg/dL (or > 125 nmol/L) is found in approximately 20% of individuals of European and South Asian descent, 40% of African Americans, and fewer than 10% of East Asians.^62^However, larger studies involving different ethnic groups are needed. Lp(a) concentrations are generally 5%-10% higher in women than in men. In men, Lp(a) levels remain relatively constant, while in women, they tend to increase slightly after menopause.^63^

Population studies have shown a linear relationship between higher Lp(a) levels and increased risk of MI and aortic valve calcification. Increased Lp(a) levels also increase the risk of recurrent ASCVD in a dose-dependent manner. Importantly, high Lp(a) is a risk factor even in individuals with low LDL-c levels.^64^

Individuals with extremely high Lp(a) levels (≥ 180 mg/dL or 390 nmol/L) are at significantly increased CV risk, with event rates comparable to other genetic dyslipidemias for which family screening is recommended.^65^

Mendelian randomization studies have clearly shown that genetic variants in the *LPA* locus, which exclusively regulate Lp(a) levels, are strongly associated with coronary artery disease (CAD) risk, suggesting a causal relationship between Lp(a) and ASCVD. Genetic studies also suggest that major reductions in Lp(a) levels (> 60%) may be necessary to reduce CV events.^66^Newer investigational agents — such as antisense oligonucleotides (ASOs) and small interfering RNAs (siRNAs), which can reduce serum levels by up to 99% — are currently being evaluated in clinical trials.^67^

Notably, commonly used lipid-lowering medications, such as statins and ezetimibe, do not reduce Lp(a) levels. Currently available therapies that moderately reduce Lp(a) (by 20%-30%) include PCSK9 inhibitors. Preliminary evidence suggests that treatment with PCSK9 inhibitors after acute coronary syndrome (ACS) in patients with very high Lp(a) may reduce CV events, independent of LDL-c decrease; however, these findings are not yet sufficient to support routine use in individuals with increased Lp(a).

Apheresis is not commonly used in our setting, as it is expensive, invasive, and time-consuming for both patients and medical teams.

In summary, Lp(a) is a prevalent risk marker for ASCVD that is still not routinely assessed. Its association with both incident and recurrent CV events, along with its potential to improve CV risk stratification, supports universal screening to identify individuals with very high levels. Although current data support the potential role of Lp(a) as a therapeutic target in the future, we still lack randomized clinical trial (RCT) results for therapies that specifically lower Lp(a).

In this context, for primary prevention, we recommend that patients with Lp(a) ≥ 50 mg/dL (or ≥ 125 nmol/L) receive early, intensive counseling on lifestyle modification and management of other risk factors. Vascular imaging studies may also be considered to detect early SAD in selected individuals, as well as to support earlier initiation of statins or other lipid-lowering therapies — especially in individuals at intermediate risk and/or those at low risk with moderately increased LDL-c levels.

In secondary prevention, the presence of increased Lp(a) is strongly predictive of recurrent events and suggests the need for more intensive LDL-c-lowering therapy and stricter control of other risk factors. Given the elevated Lp(a) levels in the index patient — whose concentration is largely genetically determined — we recommend conducting cascade screening of family members to identify other potential carriers and to assess the associated cardiovascular risk.

**Table:** 

Recommendation	Strength of Recommendation	Certainty of Evidence
In the general population, measuring Lp(a) once in a lifetime is recommended when available to assist with risk stratification and/or therapeutic management.	STRONG	MODERATE
In specific conditions, such as premature CAD, aortic stenosis, FH, family history of early ASCVD, or increased Lp(a), measuring Lp(a) once in a lifetime is recommended when available to assist with risk stratification and/or therapeutic management.	STRONG	HIGH
The preferred method for measuring Lp(a) is an assay that is isoform-independent, meaning it measures the number of particles per liter (nmol/L). Measurement in mass units (mg/dL) should be avoided. Conversion formulas do not correct the differences between methods and are not recommended.	STRONG	HIGH
Measurement of Lp(a) using a non–isoform-independent assay, i.e., one that reports mass units (mg/dL), may be used when it is the only option available.	STRONG	HIGH
Measurement of Lp(a) by an isoform-dependent assay (i.e., expressed in mass units [mg/dL]) may be used when it is the only method available. In individuals with elevated lipoprotein(a) levels – ≥50 mg/dL (or ≥125 nmol/L) – whose concentration is predominantly genetically determined, cascade screening of family members is recommended to help identify other potential carriers and to enable early cardiovascular risk assessment.	STRONG	MODERATE

ASCVD: atherosclerotic cardiovascular disease; CAD: coronary artery disease; FH: familial hypercholesterolemia; LDL-c: low-density lipoprotein cholesterol; Lp(a): lipoprotein(a).

####### 3.1.1.2.2.7. Reference Values for the Lipid Profile

This update suggests that the reference and therapeutic target values for the lipid profile (in adults over 20 years old) should be presented according to the metabolic state preceding sample collection — either fasting or 12-hour fasting. Thus, the reference and target values, determined based on the assessment of CV risk estimated by the requesting physician, are presented in Table 3.1 and should be included in laboratory reports nationwide to ensure consistency in the treatment of dyslipidemias. As with LDL-c and non-HDL-c, the reference values vary according to the estimated CV risk. In this update, therapeutic target values for these variables are suggested according to risk category. The parameters TC, HDL-c, LDL-c, and non-HDL-c are not influenced by food intake. For TG in the nonfasting state, the desirable value is < 175 mg/dL. The laboratory should state the fasting time in the report as either "nonfasting" or "12-hour fasting," according to the physician's criteria. The report should also include reference values for TG in both fasting and nonfasting states.

**Table 3.1:** Reference values or therapeutic targets for the lipid profile, apolipoprotein B, and lipoprotein(a)

Parameter	12-Hour Fasting	Nonfasting	CV Risk Category
Total Cholesterol	< 190 mg/dL	< 190 mg/dL	
TG	< 150 mg/dL	< 175 mg/dL	
HDL-c	> 40 mg/dL	> 40 mg/dL	
LDL-c	< 115 mg/dL	< 115 mg/dL	Low
	< 100 mg/dL	< 100 mg/dL	Intermediate
	< 70 mg/dL	< 70 mg/dL	High
	< 50 mg/dL	< 50 mg/dL	Very high
	< 40 mg/dL	< 40 mg/dL	Extreme
Non-HDL-c	< 145 mg/dL	< 145 mg/dL	Low
	< 130 mg/dL	< 130 mg/dL	Intermediate
	< 100 mg/dL	< 100 mg/dL	High
	< 80 mg/dL	< 80 mg/dL	Very high
	< 70 mg/dL	< 70 mg/dL	Extreme
ApoB	< 100 mg/dL	< 100 mg/dL	Low
	< 90 mg/dL	< 90 mg/dL	Intermediate
	< 70 mg/dL	< 70 mg/dL	High
	< 55 mg/dL	< 55 mg/dL	Very high
	< 45 mg/dL	< 45 mg/dL	Extreme
Lipoproteín(a)	< 75 nmol/L (< 30 mg/dL)	< 75 nmol/L (< 30 mg/dL)	Value used for **cardiovascular risk stratification** . No therapeutic target defined.

The laboratory report must indicate the fasting status (nonfasting or 12-hour fasting), based on the physician's instructions. Reference and therapeutic target values must be included in laboratory reports to standardize the treatment of dyslipidemias. For lipoprotein(a), the use of an isoform-independent assay is recommended, with results expressed in nmol/L. The mg/dL unit may be used only when an alternative is unavailable; however, it is not the preferred method.

### 3.2. Genetic Diagnosis of Dyslipidemias

#### 3.2.1. Genetically-Based Hypercholesterolemias

##### 3.2.1.1. Considerations for Requesting Genetic Testing

Genetic testing allows identification of the type and severity of the variant (defective *LDLR* gene vs. null *LDLR* gene), which correlates with the degree of hypercholesterolemia and the risk of developing CAD, including premature CAD; carriers of null variants in the *LDLR* gene have a more severe phenotype,^68 , 69^while non-null variants in *LDLR* gene as well as pathogenic variants in the APOB and PCSK9 genes, generally present with a milder phenotype.^69^The definitions genotype-positive and phenotype-positive for FH should be used to identify and treat the entire spectrum of patients with FH who have a detected pathogenic variant [genotype-positive], those without [phenotype-positive, genotype-negative], and those who have not undergone genetic testing.

Genetic confirmation positively influences the initiation of lipid-lowering therapy, adherence, and LDL-c decrease;^70 - 72^it also facilitates cascade screening of family members for FH. Since it is an autosomal semi-dominant genetic condition, screening relatives of an affected individual (index case or proband) "at risk" can be highly effective in identifying additional individuals with FH who will require treatment.^71 , 73^It helps identify new patients with FH^74 - 77^and contributes to the prevention of CAD, MI, and death.^78 - 80^Genetic cascade screening can lower the age at which affected relatives are diagnosed compared to index cases^81^and decrease TC and LDL-c levels in those relatives.^71^Guidelines on FH recommend that when a pathogenic variant is found in an index case, that specific variant should be used to screen affected family members, reducing screening costs.^82 , 83^

Genetic testing plays an important role in pre- and post-test genetic counseling. Testing the proband provides accurate risk information during counseling and guides the proper approach for family genetic cascade screening. It also enables molecular-level discrimination between individuals with monoallelic semi-dominant hypercholesterolemia, biallelic monogenic semi-dominant (formerly simple homozygous), biallelic monogenic semi-dominant with two distinct variants (formerly compound heterozygous), biallelic digenic semi-dominant (formerly double heterozygous), and biallelic recessive with two identical copies or one copy each of two distinct variants (formerly autosomal recessive).^84^

Specifically for those with biallelic digenic variants, the proband's parents should be tested for the identified variants to determine which variant was inherited from the mother and which from the father, and/or to determine if one of the variants is a de novo mutation — which, although rare, can occur. All maternal and paternal relatives with FH should be tested to detect the variant on each side of the family. Without this type of screening, probands with FH carrying two distinct variants may be misclassified as severe heterozygotes, which could negatively impact family members at risk who remain unaware that both sides of the family are affected due to the presence of two pathogenic variants in the proband.^85^

The American Society of Human Genetics recommends genetic testing in children and adolescents when clinical intervention is warranted.^86^In heterozygous FH (HeFH), statin therapy should be started between ages 8 and 10, and lifestyle interventions should begin even earlier. In children with biallelic FH, high-intensity treatment should be initiated at diagnosis.^87^If untreated, children with FH are at increased risk of coronary events in adulthood due to cumulative exposure to increased LDL-c levels, with many experiencing CV events at a young age. Children with FH who begin statin therapy early have lower event rates than their parents.^88^

We recommend that the genetic panel for FH include the following genes: *LDLR* , *APOB* , *PCSK9* , and *LDLRAP1* . The latter gene is responsible for the autosomal recessive form of FH and includes biallelic variants. Additionally, the panel should include the *ABCG5* / *ABCG8* genes, which cause sitosterolemia, and the *LIPA* gene, which causes lysosomal acid lipase deficiency (LAL-D) — both of which are phenocopies of FH^84^and require different therapeutic approaches. Genetic testing also allows inference of polygenic forms when no pathogenic variant is found (genotype-negative) in an individual with a phenotype-positive presentation.^84^In such cases, cascade genetic screening is not considered cost-effective.

**Table:** 

Recommendations for genetic testing in familial hypercholesterolemia	Strength of Recommendation	Certainty of Evidence
**Proband (index case) –** Genetic testing for FH should be offered to individuals of any age with a strong clinical suspicion of familial hypercholesterolemia (FH), based on their personal and/or family medical history. Note: This suspicion includes the following situations: Children with persistent * LDL-c levels ≥ 160 mg/dL or adults with persistent * LDL-c ≥ 190 mg/dL without an apparent secondary cause of hypercholesterolemia † and with at least one first-degree relative who is also affected, or with premature ‡ CAD, or if the family history is unavailable (eg, in cases of adoption). Children with persistent * LDL-c ≥ 190 mg/dL or adults with persistent * LDL-c ≥ 250 mg/dL without an apparent secondary cause of hypercholesterolemia † , even in the absence of a positive family history.	STRONG	MODERATE
**At-risk family members –** Cascade genetic testing for the specific variant(s) identified in the proband with FH (testing of the known familial variant) must be offered to all first-degree relatives. If first-degree relatives are unavailable or decline testing, the known familial variant should be offered to second-degree relatives. Cascade testing should continue throughout the extended family until all at-risk individuals have been tested and all relatives with FH have been identified.	STRONG	MODERATE

* Persistent: Increased LDL-c levels confirmed on at least two separate occasions;

† Secondary causes of hypercholesterolemia include hypothyroidism, nephrotic syndrome, cholestatic liver disease, and certain medications;

‡ Premature CAD: < 55 years in men and < 65 years in women. CAD: coronary artery disease; LDL-c: low-density lipoprotein cholesterol; FH: familial hypercholesterolemia. Adapted from Sturm et al.^89^

**Table:** 

Recommendations for genetic testing for familial hypercholesterolemia in different clinical scenarios	Strength of Recommendation	Certainty of Evidence
Genetic testing for FH may be considered in adults without available pretreatment LDL-c levels, but with a personal history of premature coronary artery disease ‡ and a family history of hypercholesterolemia and premature coronary artery disease ‡ .	CONDICIONAL	MODERATE
Genetic testing may be considered in adults with persistent LDL-c levels ≥160 mg/dL (in the absence of an apparent secondary cause of hypercholesterolemia † ) in the context of a family history of hypercholesterolemia and a personal or family history of premature coronary artery disease ‡ . *	CONDICIONAL	MODERATE

* Persistent: Increased LDL-c levels confirmed on at least two separate occasions;

† Secondary causes of hypercholesterolemia include hypothyroidism, nephrotic syndrome, cholestatic liver disease, and certain medications;

‡ Premature CAD: < 55 years in men and < 65 years in women; CAD: coronary artery disease; LDL-c: low-density lipoprotein cholesterol

### 3.3. Diagnosis of Hypertriglyceridemia

#### 3.3.1. Familial Chylomicronemia Syndrome

##### 3.3.1.1. Definition

Fasting plasma TG levels are normally < 150 mg/dL, and in the postprandial state, values < 175 mg/dL are considered normal.^37^TG levels may be increased due to multiple factors, including but not limited to diet, obesity, and insulin resistance conditions such as diabetes or use of certain drugs which are known to interact with polygenic determinants.^90 , 91^Extremely increased TG levels can occur in inherited conditions such as familial chylomicronemia syndrome (FCS, Fredrickson Type I), familial combined hyperlipidemia (Type IIa), dysbetalipoproteinemia or remnant lipoprotein hyperlipidemia (Type III), familial HTG (Type IV), and multifactorial chylomicronemia (MCS, Type V),^91 - 94^as well as in generalized lipodystrophies and familial partial lipodystrophy (LD).^95^

FCS is an autosomal recessive disorder that affects approximately 1 to 10 individuals per million and is most often caused by pathogenic or likely pathogenic biallelic variants in the *LPL* gene, which encodes the enzyme lipoprotein lipase (LPL). However, variants in the *GPIHBP1* , *LMF1* , *APOA5* , and *APOC2* genes have also been identified as the cause of FCS.^96 - 98^It may also present with two distinct biallelic or digenic variants.

FCS is characterized by an increased risk of recurrent acute pancreatitis.^99 , 100^

##### 3.3.1.2. Clinical and Laboratory Diagnosis of Familial Chylomicronemia Syndrome

Clinical manifestations of monogenic forms of chylomicronemia typically occur during childhood or early adulthood. Diagnosis is often delayed and made later in adulthood when complications have already developed.^90^Common findings include recurrent abdominal pain (50%),^96^recurrent pancreatitis episodes (50%),^101^hepatosplenomegaly,^102^eruptive xanthomas (17%-33%),^96^lipemia retinalis (30%), correlating with higher TG levels.^102^Neurological manifestations — such as fatigue, mental confusion, irritability, and cognitive deficits (eg, "mental fog") — are among the most commonly reported symptoms in individuals with FCS, thereby impairing their quality of life.^102 , 103^

##### 3.3.1.3. Diagnostic Scores

The score developed by Moulin et al.^104^uses the presence of TG levels (> 885 mg/dL fasting and outside the acute phase) as a selection criterion and scores based on increased TG levels and exclusion of secondary causes ( Box 3.1 ). This score has been tested in cohorts of patients with genetically confirmed FCS and in multifactorial chylomicronemia and has been validated in other cohorts. It is recommended for use as a screening tool for genetic testing, or when genetic testing is not available.

**Box 3.1:** Moulin Score for Suspected FCS^104^

Criteria		Score
Fasting TG levels > 885 mg/dL in at least three consecutive tests *		+5
Fasting TG levels > 1,770 mg/dL on at least one occasion		+1
Previous TG levels < 177 mg/dL on at least one occasion		-5
Absence of secondary factors † (except pregnancy ‡ and use of ethinylestradiol)		+2
History of pancreatitis		+1
Unexplained recurrent abdominal pain		+1
No history of familial combined hyperlipidemia		+1
No response to lipid-lowering therapy (TG decrease < 20%)		+1
Age at symptom onset:		
	< 40 years	+1
	< 20 years	+2
	< 10 years	+3
**FCS Score Interpretation**		
	**Very likely**	≥ **10**
	**Unlikely**	≤ **9**
	**Very unlikely**	≤ **8**

* Measurements taken at least 1 month apart.

† Includes alcohol consumption, diabetes, MetS, hypothyroidism, corticosteroid use, and other drugs.

‡ If diagnosis occurs during pregnancy, a second test should be performed postpartum for confirmation. FCS: familial chylomicronemia syndrome; MetS: metabolic syndrome; TG: triglycerides.

##### 3.3.1.4. Differential Diagnosis

In adults, the main differential diagnoses for FCS are MCS^92^(Fredrickson type V hyperlipoproteinemia), which includes heterozygous variants in the five canonical genes for FCS or a high polygenic score, aggravated by comorbidities or secondary causes of hypertriglyceridemia (HTG).^92^Another differential diagnosis for FCS is LD, characterized by selective loss of adipose tissue, which may present with severe HTG and pancreatitis. Inherited LDs are rare disorders that may manifest at birth (congenital generalized form) or present fat loss later in life. In partial forms, diagnostic suspicion should be considered in the presence of moderate to severe HTG associated with thigh skinfold thickness < 22 mm in women or < 10 mm in men, and/or cases of diabetes requiring subcutaneous insulin at daily doses >2 IU/kg.^95^

##### 3.3.1.5. Genetic Diagnosis

FCS may be caused by pathogenic or likely pathogenic biallelic variants in the *LPL* , *GPIHBP1* , *LMF1* , *APOA5* , or *APOC2* genes. It may also present with two distinct biallelic variants or in digenic form.^105^The genetic panels used include the five canonical genes for FCS as well as genes related to lipodystrophies, cystic fibrosis, pancreatitis, and the LIPA gene, which causes LAL-D.

##### 3.3.1.6. Lipoprotein Lipase Activity

LPL activity is markedly reduced in patients with FCS who carry a biallelic variant in the *LPL* gene, in those with biallelic loss-of-function variants in other canonical FCS-related genes, or in individuals with digenic variants^106^. Typically, the activity of this enzyme is reduced to less than 25% in patients with FCS.

##### 3.3.1.7. Other Diagnostic Tests

Autoimmune chylomicronemia may be caused by the presence of anti- *GPIHBP1* antibodies, characterized by intermittent HTG and associated with previous autoimmune conditions, including the presence of antinuclear antibodies.^107^

**Table:** 

Recommendation	Strength of Recommendation	Certainty of Evidence
Clinical scores are recommended to diagnose FCS.	STRONG	HIGH
Under ideal circumstances, genetic testing is the recommended method for confirming the diagnosis of FCS.	STRONG	HIGH
The genetic panel for FCS should include sequencing of the LPL, GPIHBP1, LMF1, APOA5, and APOC2 genes.	STRONG	HIGH

CAD: coronary artery disease; CV: cardiovascular; CVD: CV disease; FCS: familial chylomicronemia syndrome; FH: familial hypercholesterolemia; LDL-c: low-density lipoprotein cholesterol; Lp(a): lipoprotein(a); Non-HDL-c: non-high-density lipoprotein cholesterol; TG: triglycerides.

## 4. Risk Stratification

### 4.1. Cardiovascular Risk Stratification

CV risk stratification is the foundation of clinical decision-making in the prevention of CVD, guiding different therapeutic approaches, including the management of LDL-c. Matching the intensity of treatment to the absolute risk of events is essential to ensure benefits for individuals at higher risk and to avoid excessive or unnecessary treatment in those at lower risk.

Although CV risk is a continuous variable, establishing risk categories facilitates the formulation of recommendations and the implementation of the most appropriate treatment ( Table 4.1 ). Accordingly, the current guideline recommends that ASCVD risk be categorized as low, intermediate, high, very high, or extreme ( Figure 4.1 ). Individuals who have already experienced a CV event are considered at the highest risk (very high or extreme risk) ( Table 4.1 and 4.2 ). In the absence of established CVD, the risk category will be determined by the presence or absence of SAD on imaging and by numerous risk factors (both traditional and enhancers). Individuals with type 2 diabetes mellitus (T2DM) require distinct risk stratification due to the high atherogenic potential of T2DM. Similarly, recognizing the role of lifelong exposure to atherogenic lipoproteins in predisposing to events, the current guideline recommends that individuals with LDL-c ≥ 190 mg/dL be classified as high risk, while those with LDL-c between 160 and 189 mg/dL be considered at intermediate risk. For adults with no prior CV events or SAD, without diabetes and with LDL-c < 160 mg/dL, ASCVD risk categorization can be defined using risk equations or scores.

**Table 4.1:** Risk categories

Risk Category	Definition
**Low**	Calculated 10-year risk score < 5 % No risk enhancers No T2DM
**Intermediate**	Calculated 10-year risk score 5 to < 20% with no risk enhancers Calculated low risk (< 5%) with a risk enhancer present T2DM: men < 50 years, women < 56 years, without Hr stratifiers or VHr stratifiers LDL-c > 160-189 mg/dL
**High**	Calculated 10-year risk score ≥ 20% Intermediate risk with a risk enhancer SAD: carotid plaque < 50%; CAC > 100 AU or > 75th percentile; atherosclerotic plaques < 50% on CCTA; AAA LDL-c ≥ 190 mg/dL T2DM: men ≥ 50 years, women ≥ 56 years, with one or two Hr stratifier, no VHr stratifier Lp(a) > 180 mg/dL (> 390 nmol/L)
**Very high**	Significant ASCVD (coronary, cerebrovascular, or peripheral vascular with ≥ 50% stenosis) or previous major ASCVD event CAC > 300 AU T2DM with 1 VHr stratifier or ≥ 3 Hr stratifier
**Extreme** *	History of multiple major ASCVD events, or one major event AND ≥ 2 Hr conditions

* Extreme risk defined as a history of multiple major atherosclerotic cardiovascular events or 1 major atherosclerotic cardiovascular event plus at least 2 high-risk conditions AAA: abdominal aortic aneurysm; AU: Agatston units; CAC: coronary artery calcium; CCTA: coronary computed tomography angiography; CKD: chronic kidney disease; CV: cardiovascular; Lp(a): lipoprotein (a); Hr: high risk; VHr: very high risk; LDL-c: Low-density lipoprotein cholesterol; T2DM: type 2 diabetes mellitus.

**Figure 4.1:** ASCVD risk stratification. AAA: abdominal aortic aneurysm; ASCVD: atherosclerotic cardiovascular disease; AU: Agatston units; CAC: coronary artery calcium score; CKD: chronic kidney disease; CV: cardiovascular; eGFR: estimated glomerular filtration rate; FH: familial hypercholesterolemia; LDL-c: low-density lipoprotein cholesterol; PAD: peripheral arterial disease; MI: myocardial infarction.

**Table 4.2:** Criteria for Extreme CV Risk

**Major ASCVD events**	Recent ACS (within the past 12 months) History of MI History of ischemic stroke Symptomatic PAD (history of claudication with ankle-brachial index < 0.85, or prior revascularization or amputation)
**High-risk conditions**	Age ≥ 65 years FH History of CABG or PCI not related to a major ASCVD event Diabetes Hypertension CKD (eGFR 15-59 mL/min/1.73 m2) Current smoking Persistently increased LDL-c (≥ 100 mg/dL) despite treatment with maximally tolerated statin and ezetimibe Acute ASCVD less than 2 years ago

ACS: acute coronary syndrome; ASCVD: atherosclerotic cardiovascular disease; CABG: coronary artery bypass grafting; CKD: chronic kidney disease; CV: cardiovascular; FH: familial hypercholesterolemia; LDL-c: low-density lipoprotein cholesterol; eGFR: estimated glomerular filtration rate; MI: myocardial infarction; PAD: peripheral arterial disease; PCI: percutaneous coronary intervention.

### 4.2. Cardiovascular Risk Scores

CV risk scores are used to estimate the risk of a CV event over a specific time frame. Although many CV risk scores have been developed in different regions of the world, we still lack a score derived from Brazilian population data. Some scores, such as the World Health Organization^108^and Globorisk-LAC2 scores,^109^have the advantage of being calibrated for the Brazilian population and can be used for assessing CV risk.

In the United States, a new risk equation — the Predicting Risk of Cardiovascular Disease EVENTs (PREVENT) score — was published in 2024 ( https://professional.heart.org/en/guidelines-and-statements/prevent-calculator ), derived from over 3 million individuals from various cohorts and validated in another 3 million individuals. This score is intended for individuals aged 30 to 79 years without prior cardiovascular disease. In addition to traditional risk factors (sex, age, total cholesterol, HDL-c, systolic blood pressure, use of antihypertensive medication, diabetes, and smoking), it incorporates statin use, body mass index (BMI), and estimated glomerular filtration rate (eGFR), with optional variables including glycated hemoglobin (HbA1c) and urinary albumin-to-creatinine ratio. The Predicting Risk of cardiovascular disease EVENTs (PREVENT) score estimates the 10-year and 30-year risk of ASCVD events (eg, coronary death, nonfatal MI, or stroke), heart failure (HF), and total CV risk. The base model of PREVENT equations for atherosclerotic risk demonstrated satisfactory discrimination in the validation sample (C-statistic 0.774 for women and 0.736 for men).^110 , 111^Because of its robustness and contemporaneity, and in the absence of a risk equation derived from the Brazilian population, the current guideline recommends the use of PREVENT to assess 10-year ASCVD risk to define the need for lipid-lowering therapy.

### 4.3. Cardiovascular Risk Enhancers

Risk enhancers are markers not included in traditional scores that provide additional information to refine the assessment of CV risk, which may aid clinical decision-making. They are particularly useful in cases where there is uncertainty about the best medical course of action. These factors can be identified from medical history or complementary exams ( Table 4.3 ), and their performance in risk assessment can be determined by metrics such as the area under the receiver operating characteristic (ROC) curve (discrimination) or risk reclassification indices. The use of risk enhancers in clinical practice requires critical judgment: the greater the number and intensity of risk enhancers, the more appropriate it is to reclassify the individual into a higher risk category ( Figure 4.2 ). Individuals with an intermediate calculated risk benefit the most from using risk enhancers, as this is the group in which risk reclassification occurs most frequently. Therefore, active screening for additional risk enhancers is recommended in this population. Nevertheless, risk enhancers can also be used in individuals with a low estimated 10-year risk, especially from a lifetime risk perspective.

**Table 4.3:** CV Risk Enhancers

Aggravating factor	Definition
**Family history of premature CVD**	First-degree relative with an event before age < 55 (men) or < 65 (women)
**Adiposity and its manifestations**	Adiposity Hepatic steatosis, especially more severe forms (eg, with fibrosis) or associated with cardiometabolic factors
**Chronic inflammatory conditions**	Rheumatoid arthritis Psoriasis Systemic lupus erythematosus IBD (eg, ulcerative colitis and Crohn's disease) Chronic HIV infection
**Organ transplantation**	Examples include heart, liver, kidney
**Women-specific risk enhancers**	Early (≤ 12 years) or late (≥ 17 years) menarche Pregnancy-related disorders (eg, preeclampsia, eclampsia, gestational hypertension, gestational diabetes) Preterm birth Intrauterine growth restriction Recurrent miscarriages (≥ 3 spontaneous pregnancy losses) Premature menopause (< 40 years)
**Lp(a)**	≥ 50 mg/dL or ≥ 125 nmol/L
**hs-CRP**	≥ 2.0 mg/L

CV: cardiovascular; CVD: CV disease; IBD: inflammatory bowel disease; hs-CRP: high-sensitivity C-reactive protein; Lp(a): lipoprotein(a); MetS: metabolic syndrome.

**Figure 4.2:** Schematic representation of the use of risk enhancers in the assessment of CV risk. Risk enhancers should be incorporated into the continuum of CV risk. The greater the number and the severity of the alterations, the greater the upward adjustment of risk compared to that estimated by traditional scores.

#### 4.3.1. Family History of Premature Cardiovascular Disease

Compared with participants without a parental history of CVD, those with at least one parent with premature CVD (onset age < 55 years in the father or < 65 years in the mother) had a higher risk of events, with an adjusted odds ratio (OR) of 2.0 (95% CI, 1.2-3.1) for men and 1.7 (95% CI, 0.9-3.1) for women.^112^

#### 4.3.2. Adiposity and its Manifestations

Obesity, defined by a BMI ≥ 30 kg/m^2^, is consistently associated with an increased risk of major CV events, including CAD, HF, and stroke. Meta-analyses of large populations have shown that individuals with overweight or obesity have a significantly higher risk of developing CAD and CV mortality.^113^This risk is even higher in individuals with diabetes or MetS.

Visceral obesity, assessed by waist circumference (WC), is significantly associated with an increased risk of major CV events. WC is a robust measure of central adiposity and represents a relevant CV risk factor independent of BMI.^114^Meta-analyses of cohort studies have shown that a 1 standard deviation increase in WC (approximately 12.6 cm) is associated with a hazard ratio (HR) of 1.27 (95% CI, 1.20-1.33) for fatal and nonfatal CV events, adjusted for age, sex, and smoking.^115^This risk is observed across different populations and in both sexes, being slightly higher in women. These data reinforce the importance of WC as a key parameter in the assessment and management of CV risk. The current concept of adiposity is defined by an elevated BMI associated with one elevated anthropometric parameter (waist circumference, waist-to-hip ratio, or waist-to-height ratio) or with two elevated anthropometric parameters.^116 , 117^

Visceral obesity is strongly associated with the manifestation of MetS and steatosis, including hepatic steatosis. When considered independently, MetS is a well-established risk enhancer for major CV events in individuals without prior CVD. MetS is characterized by a cluster of abnormalities — increased WC, increased TG, decreased HDL-c, increased blood pressure, and increased fasting glucose — that together increase the risk of ASCVD. Several meta-analyses have shown that MetS is associated with a twofold increased risk of CVD and a 1.5-fold increased risk of all-cause mortality, regardless of the definition used.^118^

Hepatic steatosis, especially in the form of metabolic dysfunction–associated steatotic liver disease (MASLD), is consistently associated with an increased risk of major CV events. Several cohort studies have demonstrated that individuals with MASLD or nonalcoholic fatty liver disease (NAFLD) have higher HRs for MI, stroke, congestive HF, and overall CVD compared to those with no steatosis. Adjusted HRs range from 1.12 to 1.75, with stronger associations observed in patients with histological confirmation of the disease or hepatic steatosis independent of CAD characteristics. Some meta-analyses support this association.^119^

In summary, the association between obesity and CV risk is well established and becomes more significant with the development of visceral obesity, steatosis, and MetS. The link with atherogenesis occurs through multiple and overlapping mechanisms among these manifestations. Therefore, we consider MetS a risk enhancer for reclassification, while the other findings help define the clinical condition related to obesity and the effectiveness of interventions targeting obesity or steatosis.

#### 4.3.3. Chronic Inflammatory Conditions

Several chronic inflammatory and autoimmune conditions — such as rheumatoid arthritis,^120^psoriasis,^121^systemic lupus erythematosus,^122^inflammatory bowel diseases (IBDs) (eg, ulcerative colitis and Crohn's disease),^123^and chronic HIV infection^124^— are independently associated with an increased risk of various manifestations of CVD, including atherosclerosis. The proposed mechanisms are diverse and involve not only the frequent coexistence of classical risk factors and the adverse effects of treatments, but also a systemic pro-inflammatory state that promotes endothelial dysfunction and accelerates atherogenesis.

For example, a meta-analysis including over 500,000 cases and nearly 30 million controls found that psoriasis was associated with overall CVD (OR, 1.4; 95% CI, 1.2-1.7), ischemic heart disease (OR, 1.5; 95% CI, 1.2-1.9), and peripheral vascular disease (OR, 1.5; 95%, CI 1.2-1.8).^125^In the case of people living with HIV, a large systematic review showed a HR of 2.16 (95% CI, 1.68-2.77) for developing CVD compared to individuals without HIV.^126^Thus, traditional CV risk equations may underestimate the actual risk in people with inflammatory and autoimmune diseases, supporting their inclusion among risk enhancers.

#### 4.3.4. Organ Transplantation

Individuals who undergo organ transplantation — such as heart, liver, or kidney — are at increased risk of various forms of CVD, including CAD, compared with the general population. The mechanisms appear to be multifactorial: frequent presence of traditional risk factors (hypertension, diabetes, dyslipidemia), metabolic effects of immunosuppressive agents like corticosteroids, chronic inflammation, and immune activation leading to endothelial dysfunction, accelerated atherosclerosis, and a prothrombotic state.^127^Such observations support considering post-transplantation status as a CV risk enhancer.

#### 4.3.5. Women-Specific Cardiovascular Risk-Enhancing Factors

The current guideline emphasizes the importance of considering several women-specific factors in the assessment of CV risk.

##### 4.3.5.1. Age at Menarche

Age at menarche is a nontraditional but relevant CV risk factor in women. Both early menarche (commonly defined as ≤ 12 years) and late menarche (≥ 17 years) are associated with an increased risk of CV events and all-cause mortality, with stronger evidence supporting the risk associated with early menarche.^128^

##### 4.3.5.2. Pregnancy-Related Disorders and Preterm Birth

Hypertensive disorders of pregnancy (preeclampsia, eclampsia, and gestational hypertension), gestational diabetes, preterm birth, and intrauterine growth restriction are associated with increased future CV risk. A meta-analysis of data from more than 6.4 million women (258,000 with preeclampsia) demonstrated that preeclampsia was independently associated with a 4-fold higher risk of incident HF and a 2-fold higher risk of CAD. Similarly, women with gestational diabetes have a 2-fold increased risk of CV events (HR, 1.98; 95% CI, 1.57-2.50).^129^

Preterm birth has an immediate physical and emotional impact and is associated with increased maternal risk of hospitalization for CVDs, including MI, stroke, and other CV admissions.^130^

##### 4.3.5.3. Recurrent Miscarriages

Recurrent miscarriages (three or more spontaneous pregnancy losses) are associated with a significant long-term increase in maternal CV risk, including CAD, stroke, and hypertension. The risk is more pronounced in younger women (< 35 years) and increases progressively with the number of losses.^131^Therefore, a history of recurrent miscarriages should be considered an independent marker of CV risk in women, warranting clinical vigilance and early preventive interventions to address traditional risk factors and provide ongoing cardiometabolic follow-up.

##### 4.3.5.4. Premature Menopause

Premature menopause (defined as menopause before age 45, and especially before age 40) is associated with an increased risk of CVDs such as CAD, HF, stroke, PAD, and major CV events.^132^It should be incorporated into risk stratification and decision-making regarding preventive interventions.

### 4.4. Additional Tests

#### 4.4.1. Lipoprotein(a)

The association between Lp(a) and CV risk is well documented in several prospective cohort studies. The ERFC (Emerging Risk Factors Collaboration) study analyzed the impact of increased Lp(a) on CV event risk in individuals without previous CVD. The coronary event rates in the highest and lowest thirds of Lp(a) distribution were 5.6 (95% CI, 5.4-5.9) and 4.4 (95% CI, 4.2-4.6) per 1,000 person-years, respectively. The HR for coronary events, adjusted only for age and sex, was 1.16 (95% CI, 1.11-1.22) for a 3.5-fold higher usual Lp(a) level (equivalent to 1 standard deviation increase), and 1.13 (95% CI, 1.09-1.18) after further adjustment for lipids and other conventional risk factors. Adjusted HRs were 1.10 (95% CI, 1.02-1.18) for ischemic stroke and 1.01 (95% CI, 0.98-1.05) for nonvascular mortality.^133^Data from another UK cohort (UK Biobank) showed a linear association between Lp(a) and ASCVD across the entire distribution, with an HR of 1.11 (95% CI, 1.10-1.12) per 50 nmol/L increase.^64^

An Lp(a) level ≥ 50 mg/dL or 125 nmol/L indicates a risk enhancer, raising risk classification from low to intermediate or from intermediate to high. Very high levels, > 180 mg/dL or 390 nmol/L, indicate a high risk of CV events.^65^

#### 4.4.2. High-Sensitivity C-Reactive Protein

Inflammation plays a central role in the pathophysiology of ASCVD. Serum C-reactive protein (CRP), especially when measured by high-sensitivity assays, is a biomarker of systemic inflammation associated with CV outcomes, including MI and stroke, in numerous epidemiological studies. In the Framingham Offspring study, high-sensitivity CRP (hs-CRP) improved net reclassification beyond traditional risk factors by 5.6% for CV events (p = 0.014) and 11.8% for coronary events (p = 0.009).^134^A systematic review reported that analyses from four large cohorts consistently found evidence that including hs-CRP improves risk stratification among individuals initially classified as intermediate risk.^135^Therefore, the guideline recommends considering hs-CRP ≥ 2.0 mg/L as a CV risk enhancer.

A recent analysis from the Women's Health Study, which evaluated approximately 27,000 initially healthy women, showed that a single combined measurement of high-sensitivity CRP, LDL-c, and Lp(a) was predictive of incident cardiovascular events during a 30-year period. This finding suggests that the use of these biomarkers can aid in lifelong risk stratification, potentially adding value to traditional risk assessment models.^136^

Two subsequent large studies confirmed the predictive power of this combination of biomarkers in both men and women, regardless of lipid-lowering therapy use.^137 , 138^

Beyond the risk prediction potential of these three combined parameters, the demonstrated benefit from reducing their concentrations [with the cardiovascular benefit of lowering Lp(a) concentrations still yet to be demonstrated] suggests the importance of initiating early preventive strategies when these variables are elevated.

#### 4.4.3. High-Sensitivity Cardiac Troponins

Cardiac troponins I and T are specific circulating biomarkers with a well-established role in the diagnosis and prognosis of ACS. In individuals with chronic coronary syndrome and in the general population, high-sensitivity cardiac troponin (hs-cTn) assays can detect low-grade MI with prognostic value. Several prospective observational studies in healthy populations have demonstrated that increased hs-cTn levels, even within the normal range, are independently associated with CV outcomes, including CV death, CAD, and stroke,^139^providing incremental value to risk stratification beyond traditional risk factors.^140^Hs-cTn can also be used in conjunction with CAC. In MESA (Multi-Ethnic Study of Atherosclerosis), the incidence of atherosclerotic events was similar in individuals with undetectable hs-cTn and CAC = 0 at baseline, while those with detectable hs-cTn and CAC > 0 had the highest event rate (HR 3.50 compared to the reference group with undetectable hs-cTn and CAC = 0).^141^

Although promising, the use of hs-cTn for CV risk stratification in healthy individuals has important limitations. Since serum concentrations of hs-cTn are very low (in the order of pg/mL), significant variations may occur within the normal range due to assay imprecision. Physiological variations related to sex and age are also observed, with generally higher values in men and in the elderly. In addition, a clear cutoff value to accurately distinguish individuals at higher or lower CV risk has not been established, although some proposals exist. For a specific hs-cTn assay brand, Farmakis et al. suggested defining high risk when hs-cTnI is > 12 ng/L in men or > 10 ng/L in women, low risk when < 6 ng/L in men or < 4 ng/L in women, and intermediate risk for values between these limits.^139^

#### 4.4.4. B-Type Natriuretic Peptide and N-Terminal Pro-B-Type Natriuretic Peptide

Prospective population-based studies have shown that increased levels of B-type natriuretic peptide (BNP) or its N-terminal fragment (NT-proBNP) predict increased risk of a wide range of clinical outcomes, including all-cause mortality, CV mortality, HF, atrial fibrillation, stroke, transient ischemic attack (TIA), and the composite outcome of CAD and stroke, independently of other risk factors.^142 , 143^NT-proBNP has also demonstrated incremental value in CV risk discrimination and reclassification when added to models based on traditional risk factors.^143^However, well-defined thresholds for re-stratifying risk based on NT-proBNP levels are still lacking. Likewise, no RCTs guide therapeutic interventions based on this biomarker, nor are there cost-effectiveness studies supporting its routine use in clinical practice. Since the association between BNP and CV outcomes is stronger for HF and that its relationship with coronary events is not always evident,^142^the current guideline does not recommend measuring BNP or NT-proBNP for ASCVD risk stratification in asymptomatic individuals or those without established CVD.

### 4.5. Markers of Subclinical Atherosclerotic Disease

Imaging tests that demonstrate the presence of SAD can be used as evidence of ongoing ASCVD. Naturally, varying degrees of this presentation exist along a continuum, and the greater the presence and burden of subclinical disease, the higher the risk of CV events. The two main tools for detecting SAD are CAC scoring and carotid artery ultrasound with assessment of carotid plaque presence. CAC can be objectively quantified via coronary artery computed tomography. Carotid plaque presence, for which various definitions exist in the literature, may depend on operator evaluation.

#### 4.5.1. Coronary Artery Calcium Score

CAC reflects the calcified component of atherosclerotic plaque in the coronary arteries. The higher the CAC score, the greater the total plaque burden and the higher the risk of ASCVD events. Absence of CAC (CAC = 0) is a strong negative predictor of CV risk and is associated with low rates of CV events and mortality over 10 years. The favorable prognosis of CAC = 0 is observed even in older individuals or those with multiple risk factors, although it is less evident in individuals with diabetes, smokers, or those with a family history of premature ASCVD.^144^On the other hand, individuals with CAC > 300 Agatston units (AU) have a CV event risk similar to those with a prior clinical atherosclerotic event^145^and should therefore be classified as very high risk. Individuals with CAC > 100 AU or above the 75th percentile for age and sex should be considered at high risk. Among patients with FH, CAC > 100 AU indicates very high risk ( Table 4.4 ).

**Table 4.4:** CAC Score in CV Risk Stratification

Individuals with intermediate calculated risk, age > 40 years, and LDL-c between 70-159 mg/dL benefit from CAC quantification, when available
Individuals with CAC > 100 AU or above the 75th percentile for sex and age should be classified as high CV risk
Individuals with CAC > 300 AU should be classified as very high CV risk
Individuals with diabetes and CAC between 10 and 300 AU should be classified as high CV risk
Individuals with FA and CAC > 100 AU should be classified as very high CV risk

AU: Agatston units; CAC: coronary artery calcium; CV: cardiovascular; FA: familial hypercholesterolemia; LDL-c: low-density lipoprotein cholesterol.

A systematic review and meta-analysis was conducted, including studies that assessed the performance of CAC compared to traditional risk scores. Regardless of the modeling approach, the meta-analysis showed a consistent association between higher CAC scores and increased risk of CV events. The addition of CAC to traditional risk scores improved risk discrimination by 0.04 units compared to using traditional scores alone (mean difference [MD], 0.04; 95% CI, 0.01-0.06; I^2^= 0%; p = 0.0033; moderate certainty of evidence; Figure 4.3 ).^146^The outcome of risk reclassification with the inclusion of CAC in traditional cardiovascular risk scores was evaluated in six studies using the net reclassification improvement (NRI). Adding CAC to traditional scores increased risk reclassification by 0.33 units (scale from 0 to 1), compared with traditional scores alone (MD, 0.33; 95% CI, 0.17–0.49; I² = 87.5%; p < 0.001; moderate certainty of evidence; Figure 4.4 ). Such findings support the recommendation to use CAC when available in individuals over 40 years of age with LDL-c levels between 70-159 mg/dL and an intermediate calculated risk (or even low risk with a family history of premature ASCVD), to guide the need for and intensity of lipid-lowering therapy.^146^

**Figure 4.3:** Meta-analysis evaluating the change in C-statistic with the addition of the CAC score to the traditional CV risk score for MACE.^146^CAC: coronary artery calcium; CV: cardiovascular; MACE: major adverse CV events.

**Figure 4.4:** Meta-analysis evaluating risk reclassification with the addition of the CAC score to the traditional CV risk score.^146^CAC: coronary artery calcium; CV: cardiovascular.

Although cost-effectiveness studies conducted in the United Kingdom, Canada,^147^and the United States^148^recommend the use of CAC in intermediate-risk patients (10-year risk by pooled cohort equations of 5%-7.5% or by QRISK3 of 10%-20%) and advocate high-intensity statin use in patients with CAC ≥ 1 and no statin use in those with CAC = 0, a Brazilian study suggests the use of moderate-intensity statins in patients with CAC ≥ 1, using the MESA study population and microsimulation based on Brazilian costs.^149^

#### 4.5.2. Carotid Artery Ultrasound

As a tool with very low risk and relative availability, ultrasound of different arterial beds, such as the carotid and lower limb arteries, can be used to detect SAD.^150 - 152^For CV risk prediction, the most studied modality is carotid artery ultrasound, which allows the analysis of two main variables: carotid intima-media thickness (CIMT) and the presence of atherosclerotic plaque. Carotid plaque may be defined by the following criteria: (1) any focal thickening considered of atherosclerotic origin that invades the lumen of any segment of the carotid artery (protruding plaque type); or (2) in the case of diffuse atherosclerosis of the vessel wall, when CIMT is ≥ 1.5 mm in any carotid artery segment (diffuse plaque type).^150^

A meta-analysis of 14 studies showed that a 1-standard-deviation increase in common carotid CIMT was associated with increased risk of stroke (HR, 1.32; 95% CI, 1.27-1.38), MI (HR, 1.27; 95% CI, 1.22-1.33), and the composite outcome of stroke and MI (HR, 1.28; 95% CI, 1.19-1.37). However, the ability of CIMT to discriminate risk beyond Framingham risk score factors was not relevant (C-statistic increased from 0.757 to 0.759), and the NRI was low: 0.8% for the general population and 3.6% for the intermediate-risk population.^151^Since the lack of standardization in the measurement of CIMT and its low risk reclassification power, there is currently no evidence to support the clinical use of CIMT as a risk enhancer.

On the other hand, the presence of carotid plaque in asymptomatic individuals is a better predictor of CV events than CIMT. A meta-analysis of 11 population-based studies showed that carotid plaque outperformed CIMT in predicting MI (ROC AUC 0.64 vs. 0.61).^152^A Swedish cohort analysis showed that the improvement in C-statistic and NRI with the addition of plaque to SCORE2 for 10-year events was 2.20% and 46.1%, respectively (both p < 0.0001).^153^Therefore, the presence of carotid plaque provides incremental information beyond clinical risk scores alone.

### 4.6. Cardiovascular Risk Stratification in Diabetes

T2DM is associated with a 2fold higher CV risk compared to the population without T2DM. It is an independent risk factor not only for ASCVDs such as MI and ischemic stroke, but also for HF and atrial fibrillation.^154 , 155^In assessing CV risk in individuals with T2DM, in addition to traditional risk factors, T2DM-specific variables should be considered, such as disease duration (risk substantially increases when ≥ 10 years), HbA1c levels, and the presence of target organ damage. The current guideline proposes a classification of CV risk in individuals with T2DM into four categories (intermediate, high, very high, and extreme risk), based on the patient's age and the presence or absence of risk stratifiers or extreme risk criteria ( Tables 4.5 , 4.6 , 4.7 and 4.8 ).

**Table 4.5:** CV Risk Stratification in Diabetes

Risk Category	T2DM	T1DM	Required Condition
**Intermediate**	Men < 50 years Women < 56 years	Use the ST1RE calculator if < 20 years of disease	No Hr stratifier No VHr stratifier No extreme risk criteria
**High**	Men ≥ 50 years Women ≥ 56 years		One or two Hr stratifiers No VHr stratifier No extreme risk criteria
	T1DM and T2DM: any age if an Hr stratifier is present		
**Very high**	Any age if a VHr stratifier is present		One VHr stratifier or ≥ three Hr stratifiers
**Extreme**	Any age if extreme risk criteria are present		Extreme risk criteria*

* Extreme risk criteria: history of multiple major ASCVD events or one major ASCVD event plus at least two high-risk conditions (Box 8). Hr: high risk; VHr: very high risk; SBD: Brazilian Society of Diabetes; ST1RE: Steno T1 Risk Engine; T1DM: type 1 diabetes mellitus; T2DM: type 2 diabetes mellitus. Adapted from Izar et al.^157^

**Table 4.6:** Hr stratifiers in diabetes

Traditional	
	T2DM for more than 10 years
	Family history of premature CAD
	MetS as defined by the IDF
	Treated or untreated hypertension
	Active smoking
	Early-stage CV autonomic neuropathy
	Mild nonproliferative diabetic retinopathy
**Renal**	
	Kidney disease stratified as high risk (Box 4)
**SAD**	
	CAC score between 10-300 AU
	Carotid plaque < 50%
	CCTA with < 50% atherosclerotic plaque
	AAA

AAA: abdominal aortic aneurysm; AU: Agatston units; CAC: coronary artery calcium; CCTA: coronary computed tomography angiography; CV: cardiovascular; IDF: International Diabetes Federation; Hr: high risk; MetS: metabolic syndrome; SAD: subclinical atherosclerotic disease; SBD: Brazilian Society of Diabetes; T2DM: type 2 diabetes mellitus. Adaptado de Izar et al.^157^

**Table 4.7:** CV risk according to Hr and VHr renal stratifiers in diabetes

Stage DKD GFR (mL/min/1.73m^2^)			Normal	Moderately increased (microalbuminuria)	Severely increased (macroalbuminuria)
			< 30 mg/g	30-299 mg/g	≥300 mg/g
**G1**	Normal or high	≥ 90	Check age, EAR and EMAR High risk	High risk	Very high risk
**G2**	Mildly decreased	89-60		High risk	Very high risk
**G3a**	Mild to moderately decreased	59-45	High risk	High risk	Very high risk
**G3b**	Moderately decreased	44-30	High risk	Very high risk	Very high risk
**G4-G5**	Severely decreased or Kidney failure	<30	Risco muito alto	Very high risk	Very high risk

CV: cardiovascular; DKD: diabetic kidney disease; eGFR: estimated glomerular filtration rate; Hr: high risk; VHr: very high risk. Adapted from Izar et al.^157^

**Table 4.8:** VHr Stratifiers in Diabetes

No prior manifest ASCVD event – VHr 1	
	Three or more Hr stratifiers
	T1DM with duration > 20 years, diagnosed after age 18
	Stenosis > 50% in any vascular territory
	Renal VHr stratifier (Box 5)
	Severe HTG: TC > 310 mg/dL or LDL-c > 190 mg/dL
	Established CV autonomic neuropathy: two abnormal CATs for CAN
	Moderate-to-severe or severe nonproliferative diabetic retinopathy, proliferative retinopathy, or evidence of progression
**Prior manifest ASCVD event – VHr 2**	
	ACS: acute MI or unstable angina
	Previous MI or stable angina
	Atherothrombotic stroke or TIA
	Coronary, carotid, renal, or peripheral revascularization
	Peripheral vascular insufficiency or limb amputation

ACS: acute coronary syndrome; ASCVD: atherosclerotic cardiovascular disease; CAT: cardiovascular autonomic tests; TC: total cholesterol; Hr: high risk; HTG: hypertriglyceridemia; LDL-c: low-density lipoprotein cholesterol; TIA: transient ischemic attack; T1DM: type 1 diabetes mellitus; T2DM: type 2 diabetes mellitus; VHr: very high risk; MI: myocardial infarction. Adapted from Izar et al.^157^

For individuals with type 1 diabetes mellitus (T1DM) with less than 20 years of disease duration and without risk stratifiers or extreme risk criteria, the current guideline recommends the use of the Steno Type 1 Risk Engine to predict a first CV event ( https://steno.shinyapps.io/T1RiskEngine/ )^156^( Table 4.5 ).

### 4.7. Categories of Atherosclerotic Cardiovascular Risk

The current guideline recommends moving away from the binary classification of "primary prevention" and "secondary prevention" and instead recognizing CV risk along a so-called risk continuum. In this approach, CV risk is viewed as a continuous variable, ranging from one end — representing low risk in a young individual without a history of CV events and without risk factors — to the other extreme, which represents individuals with multiple recurrent CV events (see Central Illustration ).

The definitions of the risk categories recommended in the current guideline are presented in Table 4.3 .

### 4.8. Particularities of Cardiovascular Risk Stratification in Older Adults

CV risk assessment in older adults has some specific considerations. First, it is well recognized that the strength of the association between risk factors and CV events weakens with aging. On the other hand, since age is the factor with the greatest weight in determining risk, the absolute risk of CV events increases with age. Second, older risk scores did not account for the competing risk of death from nonCV causes, which often led to overestimation of risk and consequently of the predicted benefit of therapeutic interventions in older individuals. Third, most risk equations were developed for populations under 80 years old. PREVENT, for example, is intended for individuals up to 79 years of age,^110 , 111^although it has shown good performance in older populations in a study.^158^

In Europe, the SCORE2-OP (Systematic COronary Risk Evaluation 2 for Older Persons) was developed to estimate CV risk in individuals aged ≥ 70 years.^159^The score was calibrated for four regions based on the CV mortality rates of European countries, with the rates from moderate-risk areas being the most similar to those observed in Brazil. According to the SCORE2-OP for moderate-risk European regions, the 10-year risk of CV death, nonfatal MI, or nonfatal stroke in individuals aged ≥ 80 years is invariably ≥ 20%, making this population a high-risk subgroup.

### 4.9. Particularities of Cardiovascular Risk Stratification in Young Adults

Because age is the main determinant of CV risk, young individuals often have a low 10-year risk estimate even when risk factors are poorly controlled. The PREVENT score adopted in the current guideline was not developed for individuals under 30 years of age,^110 , 111^so this age group still lacks a dedicated model for risk assessment. Even lifetime risk estimation models do not include very young individuals: the Lifetime Risk model recommended by the American Heart Association includes individuals starting at age 50,^59^and the LIFE-CVD2 model starts at age 35.^161^

In addition to conventional risk factors, the current guideline recommends evaluating risk enhancers in adults under 30 years of age. Depending on the type and intensity of the risk enhancer, the individual may be reclassified into a higher risk category, especially considering the long-term consequences.

For young adults aged ≥ 30 years, calculating 30-year CV risk by using PREVENT may be appropriate. Although no thresholds are defined for risk categorization, the 30-year estimate can be used to raise awareness of risk factors and motivate patients regarding treatment.

### 4.10. Cardiovascular Risk Stratification in Childhood and Adolescence

The risk of CVD in children and adolescents can be stratified based on exposure to traditional risk factors (eg, homozygous FH [HoFH] or HeFH, hypertension, severe obesity, and T2DM). In addition, the presence of underlying conditions (eg, T1DM, CKD, childhood cancer treatment, chronic inflammatory conditions such as juvenile idiopathic arthritis [JIA]) may increase vulnerability to the adverse effects of traditional risk factors, justifying aggressive therapies for their control to reduce CV risk.^162^The current guideline adopts the risk stratification model proposed by the American Heart Association in 2019, which categorizes children based on underlying diseases^162^( Table 4.9 ).

**Table 4.9:** CV Risk Stratification in Childhood and Adolescence According to Underlying Conditions

Risk Category	Underlying Conditions
**High**	HoFH, T1DM, T2DM, end-stage renal disease, Kawasaki disease with persistent aneurysms, solid organ transplant vasculopathy, childhood cancer survivor (stem cell transplant recipient)
**Moderate**	Severe obesity, HeFH, confirmed hypertension, aortic coarctation, increased Lp(a), pre-dialysis CKD, aortic stenosis, childhood cancer survivor (thoracic radiotherapy)
**At risk**	Obesity, insulin resistance with comorbidities (eg, dyslipidemia, NAFLD, and PCOS), white-coat hypertension, HCM and other cardiomyopathies, pulmonary hypertension, chronic inflammatory conditions (eg, JIA, SLE, IBD, HIV), coronary artery translocation or anomalous coronary arteries, or transposition of the great arteries, childhood cancer (chemotherapy with cardiotoxic agents only), Kawasaki disease with regressed aneurysms

AS: aortic stenosis; CKD: chronic kidney disease; FH: familial hypercholesterolemia; HCM: hypertrophic cardiomyopathy; IBD: inflammatory bowel disease; JIA: juvenile idiopathic arthritis; Lp(a): lipoprotein(a); NAFLD: nonalcoholic fatty liver disease; PCOS: polycystic ovary syndrome; SLE: systemic lupus erythematosus; T1DM: type 1 diabetes mellitus; T2DM: type 2 diabetes mellitus.

**Table:** 

Recommendation	Strength of Recommendation	Certainty of Evidence
For individuals aged 30-79 years without established CVD, the use of a risk equation to estimate the 10-year risk of an ASCVD event is strongly recommended.	STRONG	HIGH
For individuals aged 30-79 years without established CVD, using PREVENT to estimate the risk of an ASCVD event is strongly recommended.	STRONG	HIGH
For individuals with an intermediate calculated risk, using risk enhancers to reclassify the risk is strongly recommended, regardless of age group.	STRONG	HIGH
For individuals with low calculated risk or aged 18-30 years, using risk enhancers may be applied to reclassify the risk.	STRONG	LOW
For individuals initially classified as intermediate risk, aged > 40 years and LDL-c between 70-159 mg/dL, the CAC score may be useful to determine the need and intensity of lipid-lowering therapy.	STRONG	MODERATE
For individuals initially classified as low risk, aged > 40 years and LDL-c between 70–159 mg/dL, the CAC score is reasonable for those with a family history of premature ASCVD to determine the need and intensity of lipid-lowering therapy.	CONDICIONAL	MODERATE

ASCVD: atherosclerotic cardiovascular disease; AU: Agatston units; CAC: coronary artery calcium; CVD: cardiovascular disease; LDL-c: low-density lipoprotein cholesterol; PREVENT: Predicting Risk of cardiovascular disease EVENTs.

## 5. Treatment Targets

### 5.1. Primary and Co-Primary Target: Low-Density Lipoprotein Cholesterol and Non-High-Density Lipoprotein Cholesterol

Although the defined cholesterol targets have not been systematically tested but derived from randomized controlled trials (RCTs) sub-analyses based on achieved LDL-c levels, we believe that defining clear targets allows for personalized treatment aligned with individual CV risk. Moreover, using targets can facilitate communication between physician and patient, improving adherence and helping to reduce therapeutic inertia ( Table 5.1 ).

**Table 5.1:** Recommended Targets According to CV Risk

CV Risk Category	LDL-c Target (mg/dL)	% LDL-c decrease	Non-HDL-c Target (mg/dL)	ApoB Target (mg/dL)
	Primary Target	Primary target	Co-Primary Target	Secondary Target
**Low risk**	< 115	≥ 30%	< 145	< 100
**Intermediate risk**	< 100	≥ 30%	< 130	< 90
**High risk**	< 70	≥ 50%	< 100	<70
**Very high risk**	< 50	≥ 50%	< 80	< 55
**Extreme risk**	< 40	≥ 50%	< 70	< 45

ApoB: apolipoprotein B; CV: cardiovascular; LDL-c: low-density lipoprotein cholesterol; Non-HDL-c: non-high-density lipoprotein cholesterol.

Evidence from interventional studies and meta-regressions clearly and unequivocally demonstrates that CV risk is reduced by lowering LDL-c.^163 - 169^This reduction is proportional to the magnitude of LDL-c decrease: for every 39 mg/dL (1 mmol/L) reduction in LDL-c, the relative risk (RR) of major CV events is reduced by approximately 20%-25%, with no evidence of a lower limit below which no additional benefit is observed.^166^Thus, the current guideline recommends not only specific targets but also the adoption of a minimum percentage reduction in LDL-c.

Non-HDL-c, obtained by subtracting HDL-c levels from total plasma cholesterol concentration, shows a strong correlation with circulating serum levels of apoB.^57 , 170 - 172^Therefore, it is considered a more accurate marker of the atherogenic burden associated with lipid-rich lipoproteins than isolated LDL-c concentration, especially in individuals with HTG.^171 - 173^In such cases — where LDL particles are enriched with TG — the cholesterol concentration within LDL particles may be relatively reduced, leading to an underestimation of the total atherogenic burden contributed by both LDL and other atherogenic lipoproteins.^174^Therefore, non-HDL-c has been recognized as a more accurate predictor of CV risk in such clinical contexts.^175 - 177^The recommended target for non-HDL-c, aimed at reducing the risk of ASCVD events, is defined as a value 30 mg/dL higher than the LDL-c goal for each CV risk category.^55^This parameter is considered an additional therapeutic target to be pursued after achieving the recommended LDL-c target for the respective risk stratum.^55^

Accordingly, the current guideline sets goals for LDL-c as the primary therapeutic target and for non-HDL-c as a co-primary target, both defined according to the CV risk category.

In line with evidence from RCTs, genetic studies, and epidemiological data, it is recommended to introduce pharmacological therapy in individuals classified as low risk with LDL-c levels persistently above 145 mg/dL, despite lifestyle measures.^178 - 188^

Therapeutic intervention recommendations:

- **For individuals at low to intermediate risk:** a reduction in LDL-c of 30% or more is recommended.
- **For individuals at high, very high, or extreme risk:** the therapeutic goal is a reduction in LDL-c of 50% or more.

High-potency statins or combination therapy are preferably recommended. Because of the interindividual variability in response to statins,^189^it is often necessary to combine them with other lipid-lowering agents to achieve the proposed targets.

### 5.2. Recommendations for Targets According to Cardiovascular Risk Stratification ( Figure 5.1 )

**Figure 5.1:** Target Recommendations According to Cardiovascular Risk Stratification.

#### 5.2.1 Individuals at Intermediate Risk

A meta-analysis based on individual patient data from RCTs with statins showed that in individuals categorized as low risk, there was an absolute reduction of 11 major CV events per 1,000 people for every 38.7 mg/dL reduction in LDL-c over 5 years. This benefit far outweighs any inherent risks associated with statin therapy.^190^

Even if stratified as low risk, individuals who maintain LDL-c ≥ 145 mg/dL despite lifestyle modifications, excluding secondary causes, qualify for initiation of lipid-lowering therapy.^178 - 188^

In this context, it is recommended:

- LDL-cdecrease ≥ 30%
- Primary LDL-c target: < 115 mg/dL
- Co-primary non-HDL-c target: < 145 mg/dL

#### 5.2.2. Individuals at Intermediate Risk

In this context, it is recommended:

- LDL-c decrease ≥ 30%
- Primary LDL-c target: < 100 mg/dL
- Co-primary non-HDL-c target: < 130 mg/dL

#### 5.2.3. Individuals at high risk

For individuals classified as high CV risk, the current guideline recommends high-intensity lipid-lowering therapy. Therefore, the preferential use of high-potency statins or combination therapy (statin plus ezetimibe) is recommended.

In this context, it is recommended:

- LDL-c reduction ≥ 50%
- Primary LDL-c target: < 70 mg/dL
- Co-primary non-HDL-c target: < 100 mg/dL

#### 5.2.4. Individuals at Very High Risk

The LDL-c target is < 50 mg/dL and the non-HDL-c target is < 80 mg/dL. This recommendation is primarily based on the IMPROVE-IT (IMProved Reduction of Outcomes: Vytorin Efficacy International Trial) study, in which combination therapy with simvastatin and ezetimibe, reaching a mean LDL-c of 53 mg/dL, resulted in a further decrease in CV risk compared to monotherapy (mean LDL-c of 69 mg/dL).^165^

In patients with established ASCVD — and therefore at very high CV risk — the combination of moderate-potency statin and ezetimibe has been shown to be noninferior to high-potency statin monotherapy in reducing CV events. In the same study, combination therapy was associated with a higher rate of achieving recommended LDL-c targets and a lower rate of lipid-lowering therapy discontinuation due to intolerance.^191^Subsequent evidence showed additional CV risk reduction with the addition of PCSK9 inhibitors in individuals with ASCVD and residual dyslipidemia despite statin therapy, with or without ezetimibe. In the FOURIER (Further Cardiovascular Outcomes Research With PCSK9 Inhibition in Subjects With Elevated Risk) trial, LDL-c decrease mediated by evolocumab (median level of 30 mg/dL) reduced the RR of major CV events by 15% after a median follow-up of 2.2 years.^168^In the ODYSSEY OUTCOMES (Evaluation of Cardiovascular Outcomes After an Acute Coronary Syndrome During Treatment With Alirocumab) trial, alirocumab use in patients post-ACS (1 to 12 months prior to randomization) reduced LDL-c to a median of 53 mg/dL, resulting in a 15% RR decrease in clinically relevant CV outcomes after a median follow-up of 2.8 years.^169^

In this context, it is recommended:

- LDL-c decrease ≥ 50%
- Primary LDL-c target: < 50 mg/dL
- Co-primary non-HDL-c target: < 80 mg/dL

#### 5.2.5. Individuals at Extreme Risk

Patients with ASCVD, even after an ACS, have a variable residual risk for new CV outcomes. A sub-analysis of the ODYSSEY OUTCOMES trial, which stratified participants according to the risk categorization proposed by the ACC/AHA Dyslipidemia Guideline, showed a CV event rate of 20.4% in the subgroup with a history of multiple prior events, compared to 5.6% among those without high-risk criteria.^192^Similarly, several sub-analyses of the FOURIER trial revealed a higher incidence of CV events in subgroups with high-risk features such as extensive coronary disease, multiple prior CV events, increased Lp(a), presence of diabetes, PAD, and multivessel disease. As expected, considering that the absolute benefit of therapeutic intervention is proportional to baseline risk, these patients experienced greater clinical benefit when achieving more stringent lipid targets and using higher-cost therapies^193 - 197^— reflected in lower number needed to treat and, consequently, improved cost-effectiveness. A secondary analysis of the FOURIER trial demonstrated a monotonic linear relationship between achieved LDL-c levels and incidence of adverse CV events, with progressive risk reduction observed down to serum concentrations below 20 mg/dL. Such findings reinforce the principle that in CV prevention, "the lower the LDL-c, the better."^198^Moreover, to date, there is no consistent evidence of adverse events associated with very low LDL-c levels.^199 - 203^Thus, the current guideline recommends more stringent therapeutic targets for individuals classified as being at extreme CV risk: LDL-c < 40 mg/dL and non-HDL-c < 70 mg/dL.

In this context, it is recommended:

- LDL-c reduction ≥ 50%
- Primary LDL-c target: > 40 mg/dL
- Co-primary non-HDL-c target: < 70 mg/dL

### 5.3. Apolipoprotein B

Measurement of serum ApoB levels is widely recognized as the most accurate indicator of CV risk associated with atherogenic lipoproteins. This is because each potentially atherogenic lipoprotein particle circulating in the bloodstream — including LDL, VLDL, Lp(a), VLDL remnants, and chylomicrons — contains exactly one ApoB molecule. Thus, direct quantification of ApoB accurately reflects the total number of circulating atherogenic particles. Furthermore, its measurement does not require fasting, which facilitates its clinical use. Notably, chylomicrons contain a truncated isoform of ApoB, ApoB-48, while the other atherogenic particles contain ApoB-100.^7 , 59 , 204 , 205^

Currently, ApoB testing is not covered by most health insurance plans and is also not available through the SUS. As a viable alternative, the measurement of non-HDL-c shows good correlation with serum ApoB levels and can be easily obtained from any standard lipid panel, since it only requires subtracting HDL-c from total cholesterol. This approach represents a practical, accessible tool for estimating atherogenic burden in clinical settings where the direct measurement of ApoB is unavailable.^57 , 170 , 171^However, ApoB targets may be used for additional stratification in patients who have already achieved their LDL-c and non-HDL-c targets. The equivalence between LDL-c and ApoB targets^206^is shown below:

- LDL-c 40 mg/dL ≈ ApoB 45 mg/dL
- LDL-c 50 mg/dL ≈ ApoB 55 mg/dL
- LDL-c 70 mg/dL ≈ ApoB 70 mg/dL
- LDL-c 100 mg/dL ≈ ApoB 90 mg/dL
- LDL-c 115 mg/dL ≈ ApoB 100 mg/dL

### 5.4. High-Density Lipoprotein Cholesterol

No specific therapeutic targets are proposed for HDL-c. Although epidemiological studies have shown a strong association between low HDL-c levels and a higher incidence of CV events,^207^there is no robust evidence indicating that this relationship is causal. In Mendelian randomization studies, individuals genetically predisposed to higher HDL-c levels did not present a reduced risk of atherosclerosis.^208^Moreover, therapies aimed at increasing HDL-c levels have not demonstrated consistent benefits in reducing CV events.^209 - 214^Thus, HDL-c should be used as a CV risk marker but not as a therapeutic target.

### 5.5. Triglycerides

TG-rich lipoproteins and particularly their metabolic remnants contribute to the residual risk of ASCVD. Serum TG concentration is a marker of atherosclerotic residual risk,^215 , 216^and tends to increase alongside the prevalence of conditions such as obesity and T2DM, in which TG levels are often mildly to moderately increased.^217 , 218^

Fasting serum TG levels ≥ 150 mg/dL or postprandial TG levels ≥ 175 mg/dL are considered abnormal,^173^e and the recommended target is to maintain levels below these thresholds. However, bear in mind that LDL-c and non-HDL-c targets are more important for patients with mild to moderate HTG.^91^

Besides lifestyle modifications, which are effective in lowering TG, the current guideline recommends, when pharmacological therapy is necessary, the preferential use of agents targeting non-HDL-c decrease, such as high-intensity statins and ezetimibe. Proper glycemic control contributes to lowering serum levels of TG. In this regard, glucose-lowering agents with proven CV benefits, such as SGLT2 inhibitors and GLP-1 receptor agonists (GLP-1 RAs), may be used to simultaneously manage glycemia and reduce CV risk. Even in nondiabetic individuals, high doses of a GLP-1 RA (semaglutide 2.4 mg) have been shown to reduce TG and CV risk in patients with established CVD and overweight or obesity.^219^

Severe HTG, defined as serum levels of TG ≥ 500 mg/dL (or ≥ 885 mg/dL according to many authors), is associated with an increased risk of acute pancreatitis. In such cases, more potent TG-lowering agents, such as fibrates, are recommended, with the therapeutic goal of keeping levels < 500 mg/dL.

### 5.6. Lipoprotein(a)

Currently, no treatment is specifically approved to lower serum levels of Lp(a). Ongoing phase 3 prospective studies may provide consistent data on the relationship between the degree of Lp(a) decrease and its effect on CV outcomes in patients at high or very high risk.^220^

Nevertheless, the current guideline reinforces the importance of assessing the levels of Lp(a) at least once in a lifetime for all individuals. Even though the impact of Lp(a) decrease on CV risk remains uncertain and therapeutic targets have not yet been defined, the documentation of increased levels of Lp(a) may assist in CV risk stratification,^221 , 222^thus supporting the adoption or intensification of strategies aimed at controlling other modifiable risk factors.

**Table:** 

Recommendation	Strength of Recommendation	Certainty of Evidence
In individuals at extreme CV risk, LDL-c < 40 mg/dL and non-HDL-c < 70 mg/dL targets are recommended.	STRONG	MODERATE
In individuals at very high CV risk, LDL-c < 50 mg/dL and non-HDL-c < 80 mg/dL targets are recommended.	STRONG	HIGH
In individuals at high CV risk, LDL-c < 70 mg/dL and non-HDL-c < 100 mg/dL targets are recommended.	STRONG	HIGH
In individuals at intermediate CV risk, LDL-c < 100 mg/dL and non-HDL-c < 130 mg/dL targets are recommended.	STRONG	HIGH
In individuals at low CV risk, LDL-c < 115 mg/dL and non-HDL-c < 145 mg/dL targets are recommended.	STRONG	MODERATE
In individuals at high, very high, or extreme CV risk, a ≥ 50% decrease in LDL-c is recommended.	STRONG	HIGH
In individuals at low or intermediate CV risk, a ≥ 30% decrease in LDL-c is recommended.	STRONG	HIGH
In all individuals, especially those with LDL-c or non-HDL-c above the target, lifestyle interventions are recommended.	STRONG	HIGH
In individuals at high, very high, or extreme CV risk, pharmacological therapy combined with lifestyle modifications is recommended.	STRONG	HIGH
In individuals at high, very high, or extreme CV risk with persistently increased LDL-c or non-HDL-c, intensification of pharmacological therapy combined with lifestyle modifications is recommended.	STRONG	HIGH
In individuals at low or intermediate CV risk with LDL-c or non-HDL-c persistently ≥ 30 mg/dL above target, initiation or intensification of pharmacological therapy combined with lifestyle modifications is recommended.	STRONG	HIGH
In individuals with LDL-c and non-HDL-c levels within target, considering an ApoB target to decide on therapeutic intensification is recommended.	STRONG	MODERATE

ApoB: apolipoprotein B; CV: cardiovascular; HDL-c: high-density lipoprotein cholesterol; LDL-c: low-density lipoprotein cholesterol.

## 6. Nonpharmacological Treatment

### 6.1. Lifestyle Recommendations to Improve Lipid Profile

#### 6.1.1. Nutritional Aspects

The recent trend is to emphasize a qualitative rather than a quantitative approach to macronutrients, prioritizing minimally processed, fiber-rich dietary patterns, and reducing the intake of added sugars and refined carbohydrates.^223^

See below for evidence regarding the main dietary macronutrients:

#### 6.1.2. Carbohydrates

In the PURE study, a high carbohydrate intake (> 60% of total energy) was associated with increased all-cause mortality.^224^Similarly, the ARIC (Atherosclerosis Risk in Communities) study showed that diets providing 50%-55% of total energy from carbohydrates were associated with lower total mortality risk, whereas diets high in refined carbohydrates (eg, sugars and processed flours) were linked to increased risk of CVD and metabolic diseases.^224 , 225^As demonstrated in a recent meta-analysis of prospective cohorts, extreme diets (< 40% or > 65% of energy from carbohydrates) were associated with higher overall mortality.^226^

**Table:** 

Recomendações dietéticas para o tratamento das dislipidemias	% do valor calórico total (VCT)	Strength of Recommendation	Certainty of Evidence
Gorduras totais	20-35%^228^	STRONG	HIGH
Gorduras saturadas	< 7%^55^	STRONG	HIGH
Gorduras trans	Não ingerir^228^	STRONG	HIGH
Ácidos graxos monoinsaturados	15%^229^	STRONG	HIGH
Ácidos graxos poli-insaturados	5-10%^229^	STRONG	HIGH
Fibras	25 g/dia	CONDICIONAL	MODERATE
Carboidratos totais	50-55%^223 , 224^	STRONG	HIGH

It is recommended to reduce or eliminate added sugars and refined flours^224 , 226^and to prioritize foods with a low glycemic index (eg, legumes, whole grains, fruits with peel) and fiber-rich carbohydrate sources.^227^

#### 6.1.3. Fats

Replacing saturated fats with unsaturated fats, such as monounsaturated (eg, olive oil, avocado) and polyunsaturated fats (eg, fatty fish, flax seeds), has been shown to reduce LDL-c and prevent CVD. Conversely, replacing saturated fats with refined carbohydrates tends to increase TG levels and lower HDL-c, both of which are also associated with increased CV risk.^55 , 228 , 229^

#### 6.1.4. Soluble Fiber

Soluble fibers are characterized by their ability to absorb water and form a viscous gel in the gastrointestinal tract, which inhibits micelle formation and delays gastric emptying. This leads to reduced cholesterol absorption, increased bile acid excretion, and modulation of the gut microbiota, resulting in significant cholesterol-lowering effects.^230 - 232^

The recommendation is a minimum intake of 25 g/day of total fiber,^233^including sources such as β-glucan (oats),^231^psyllium, legumes, and fruits with peel.

### 6.2. Smoking Cessation

Smoking accounts for 50% of all preventable deaths among smokers, with half of these being related to ASCVD.^234^The risk of ASCVD in smokers under 50 years of age is five times higher than in nonsmokers and is associated with a higher incidence of stroke, PAD, and sudden cardiac death.^235^Smoking contributes to the development of atherosclerosis through multiple pathophysiological mechanisms: endothelial dysfunction due to reduced bioavailability of endothelial nitric oxide (NO); increased production of reactive oxygen species (ROS), which promotes LDL-c oxidation; increased circulating levels of inflammatory markers and stimulation of a prothrombotic state; and a decrease in HDL-c levels.^236 , 237^Smoking cessation provides immediate and progressive CV benefits, regardless of age or smoking duration. After 1 year of smoking abstinence, the risk of MI decreases by 50%.^238^

The approach should be multifactorial and include both behavioral and pharmacological strategies. Individual or group counseling programs are effective in reducing smoking rates. The indication of pharmacological treatment depends on the patient's level of nicotine dependence, which can be assessed using the Fagerström test.^239^This approach includes nicotine replacement therapy (NRT) using patches, gums, and lozenges. Bupropion, a dopamine and norepinephrine reuptake inhibitor, helps reduce withdrawal symptoms, contributing to more effective management.

**Table:** 

Recommendation	Strength of Recommendation	Certainty of Evidence
Smoking cessation reduces the development of atherosclerosis and, consequently, CV risk.	STRONG	HIGH

CV: cardiovascular.

### 6.3. Management of Weight

In individuals with obesity, the lipid profile is typically characterized by increased TG, low HDL-c, and small, dense, pro-atherogenic LDL-c particles. These factors, combined with increased visceral fat and insulin resistance, place many patients within the criteria for MetS.^240^

Nonpharmacological treatment of obesity requires a continuous, patient-centered approach. Multidisciplinary behavioral interventions must last at least 6 months to promote lifestyle changes. Weight loss of 8%-10% is directly associated with a reduction of 5-10 mg/dL in LDL-c, 20-30 mg/dL in TG, and an increase of 3-5 mg/dL in HDL-c.^240 , 241^These findings were also supported by the Look AHEAD trial,^242^which included patients with diabetes, overweight, or obesity. A weight loss of 5%-10% within 1 year was associated with a 40 mg/dL decrease in TG and a 5 mg/dL increase in HDL-c. However, LDL-c decrease was modest and not statistically significant. Long-term weight maintenance remains challenging, with recurrent weight regain observed after 2 years of follow-up.^243^

The adoption of healthy eating habits from childhood helps prevent obesity and related chronic diseases.^244^Recent studies have focused on various diets and behaviors, particularly caloric restriction strategies, which have proven effective for weight loss within 6 months.^245 , 246^However, the benefits of weight loss on CV risk factors are mostly observed over the long term.^247^Multidisciplinary approaches involving psychologists, nutritionists, physical educators, and mental health professionals have been shown to be effective.^248^

**Table:** 

Recommendation	Strength of Recommendation	Certainty of Evidence
Weight loss through nonpharmacological measures is recommended to increase HDL-c levels, decrease TG, and modestly decrease LDL-c.	STRONG	HIGH

TG: triglycerides; HDL-c: high-density lipoprotein cholesterol; LDL-c: low-density lipoprotein cholesterol.

### 6.4. Spirituality

A growing body of evidence highlights a strong relationship between spirituality, religiosity, and the processes of health, illness, and healing, forming part of a holistic view of the human being alongside physical, psychological, and social aspects.^249^Spirituality and religiosity are often used by patients to cope with illness and suffering. Understanding their relevance, identifying needs, and providing appropriate spiritual and religious support benefit not only patients, but also the multidisciplinary team and the health care system as a whole. Spirituality is expressed through beliefs, values, traditions, and practices.^250^

Scientific evidence shows that religiosity/spirituality is associated with lower prevalence of smoking, alcohol consumption, and physical inactivity, and with better nutritional and pharmacological adherence in individuals with dyslipidemia, hypertension, obesity, and diabetes.^251 , 252^

**Table:** 

Recommendation	Strength of Recommendation	Certainty of Evidence
Spiritual and religious guidance should be part of medical consultations due to its impact on CV health.	CONDICIONAL	MODERATE

CV: cardiovascular.

### 6.5. Physical Activity

Sedentary behavior is associated with an increased risk of several major chronic diseases and mortality, while physical activity is an essential component in the prevention and treatment of CVD.^253^Its impact on lipids is primarily seen through increases in HDL-c and a moderate benefit in the levels of LDL-c, although changes in the absolute levels of LDL-c are less pronounced.^254^Additionally, there is a consistent reduction in plasma levels of TG.^255^

Adults should perform at least 150 minutes/week of moderate-intensity aerobic activity or 75 minutes of vigorous activity and can benefit from combining both.^256^

Resistance training is associated with lower risks of CV events and all-cause mortality. It is recommended to perform 1 to 3 sets of 8-12 repetitions at 60%-80% of 1-repetition maximum (1-RM), at least twice per week, across 8-10 exercises targeting all major muscle groups.^256^

For older adults or deconditioned individuals, it is recommended to start with 1 set of 10–15 repetitions at 40%–50% of one-repetition maximum (1-RM). In addition, older adults should engage in multicomponent physical activity that includes aerobic, muscle-strengthening, and balance exercises to prevent falls.^257 , 258^

For physically inactive adults, even low-intensity physical activity — such as 15 minutes/day — can yield health benefits.^257 - 259^

Before starting an exercise program, a clinical evaluation is recommended. If available, a treadmill stress test while on CV medication can help determine the appropriate exercise intensity based on heart rate. According to the American College of Sports Medicine, light to moderate activity should correspond to 50%-70% of peak heart rate, while moderate to vigorous activity should be in the 70%-85% range for those already adapted.^260^

**Table:** 

Recommendation	Strength of Recommendation	Certainty of Evidence
Adults should perform at least 150 minutes of moderate-intensity aerobic activity/week or 75 minutes/week of vigorous activity.	STRONG	HIGH

HDL-c: high-density lipoprotein cholesterol; LDL-c: low-density lipoprotein cholesterol; TG: triglycerides.

### 6.6. Alcohol Intake

Epidemiological studies suggest that high alcohol consumption is associated with increased risk of stroke, CAD, and HF, while an inverse (log-linear) relationship is observed with MI risk.^261^Mendelian randomization studies indicate that the lowest CV risk occurs in abstinent individuals, and that any amount of alcohol may raise blood pressure and BMI.^262 , 263^

The upper safety limit for alcohol consumption is approximately 100 g/week, for both men and women.^261^Biomarker studies suggest that moderate alcohol intake (especially red wine) may increase HDL-c and adiponectin, decrease fibrinogen,^264 , 265^and improve markers of inflammation and hemostasis.^266^On the other hand, high consumption (> 30 g/day) is associated with increased TG and TC. Regarding glucose metabolism, light alcohol intake may reduce the risk of T2DM, showing a U-shaped association in both sexes; however, this association is lost with doses > 50-60 g/day and is independent of the beverage type.^267 , 268^

The harmful effects of high alcohol consumption are well documented. Therefore, recommendations should be made with caution since definitive conclusions are still lacking.

**Table:** 

Recommendation	Strength of Recommendation	Certainty of Evidence
Alcohol consumption for the prevention and treatment of atherosclerosis should not be encouraged.	STRONG	HIGH

### 6.7. Dietary Supplements and Functional Foods in Dyslipidemia

These options may be considered as an additional strategy for patients who do not achieve lipid targets through lifestyle changes alone. Clinical evidence is variable, and well-designed studies are needed to confirm the efficacy and safety of these supplements.

Main studied functional foods include:

- **Soy protein** : Believed to reduce cholesterol absorption and increase fecal steroid excretion.^269^
- **Phytosterols** : Compete with cholesterol for intestinal absorption. Efficacy depends on dosage, baseline LDL-c levels, and the food carrier used.^270^The European Society of Cardiology (ESC)/European Atherosclerosis Society guidelines^55^now recommend plant sterols as part of lifestyle changes to reduce cholesterol. However, recent genetic evidence suggests a potential atherogenic effect, reinforcing the need for RCTs with robust CV outcomes before broad recommendations, as noted by the German Cardiac Society.^271^
- **Green tea** : May provide vascular benefits by improving endothelial function.^272^Proposed mechanisms include reduced intestinal cholesterol absorption^228^and increased fecal fatty acid excretion.^273^
- **Sesame seeds** : Contain unsaturated fatty acids, vitamin E, and lignans, which may have favorable effects on lipids and CV health by reducing TG levels.^274^
- **Probiotics** : May lower TC and LDL-c, improve BMI, and decrease inflammatory markers. The main proposed mechanism is decreased enterohepatic circulation of bile salts.^275^Studies report reductions of TC (–13.6%), LDL-c (–8.4%), LDL-c/HDL-c ratio (–12.8%), TG (–9.0%), and oxidized LDL (–11.3%), along with HDL-c increase (+5.5%) with *Lactobacillus plantarum* .^276^
- **Red yeast rice (RYR)** : A fermented product synthesized by *Monascus purpureus* yeast, contains monacolins — bioactive compounds with lipid-lowering effects.^277^Monacolin K, similar to lovastatin, is widely used to manage cholesterol. Studies indicate that RYR has varying phytochemical composition and potential synergistic effects, inhibiting 3-hydroxy-3-methylglutaryl-coenzyme A (HMG-CoA) reductase more effectively than lovastatin alone. It also shows lower cellular toxicity, especially in muscle cells. However, further research is needed to validate its clinical efficacy.^278^
- **Fish oil:** Rich in marine omega-3 fatty acids such as eicosapentaenoic acid (EPA) and docosahexaenoic acid (DHA), fish oil is recommended in clinical guidelines for patients with HTG.^279^It lowers the levels of TG by inhibiting TG synthesis and turnover.^280^The results of specific studies on omega-3 treatment will be addressed in the dedicated treatment chapter.

**Table:** 

Recommendation	Strength of Recommendation	Certainty of Evidence
Dietary supplementation * should be considered as part of the management of dyslipidemia.	CONDITIONAL	MODERATE

* Supplements with proven LDL-c impact include RYR, probiotics, phytosterols. For TG decrease: fish oil (EPA/DHA).

## 7. Pharmacological Treatment

The treatment of dyslipidemias has undergone remarkable advancements in recent decades. Although lipid-related risk factors for CVD are partly influenced by lifestyle, optimal lipid control often requires additional pharmacological interventions. Statins have traditionally been the cornerstone of treatment, with proven efficacy in lowering LDL-c levels and preventing CV events. When combined with other oral medications, such as ezetimibe and more recently adenosine triphosphate (ATP) citrate synthase inhibitor, these therapies have provided additional improvements in lipid profiles.

However, biotechnological advances have enabled the development of long-acting injectable therapies, such as PCSK9 inhibitors and RNA interference agents, which allow for even greater reductions in LDL-c, with convenient dosing schedules and good tolerability. In parallel, new therapies have emerged targeting specific pathways, including apolipoprotein C-III (ApoC-III), angiopoietin-like proteins 3 and 4, and Lp(a), expanding therapeutic options beyond the control of LDL-c.

More recently, gene therapy and genetic editing approaches have begun to emerge as a new frontier in the management of dyslipidemias, with the potential to permanently modify metabolic pathways involved in lipid regulation. These advancements reflect a paradigm shift in the treatment of dyslipidemia, enabling increasingly personalized and effective interventions for the prevention of ASCVD.

Figure 7.1 illustrates the key molecular targets for therapeutic interventions in dyslipidemia.

**Figure 7.1:** Molecular targets of the main treatments for dyslipidemia. BPA: bempedoic acid; CE: cholesteryl esters; LPL: lipoprotein lipase; TG: triglycerides.

### 7.1 Statins

The decrease in the levels of LDL-c through statin therapy remains the most validated approach for decreasing CV events and overall mortality, both in primary and secondary prevention.^55^Statins are competitive inhibitors of HMG-CoA reductase, the rate-limiting enzyme in cholesterol synthesis. Inhibiting HMG-CoA reductase increases the expression of hepatic LDL receptors, enhancing LDL clearance from circulation. Similarly, the action of statins can potentially affect the broader range of circulating lipoproteins that interact with LDL receptors, such as LDL, VLDL, and chylomicron remnants.

The degree of LDL-c decrease depends on the dose and varies among different statins. High-intensity therapy involves potent statins used at higher doses and typically lowers LDL-c by 50% or more. Moderate-intensity therapy is defined by an expected LDL-c reduction of 30%-50%.^37^Importantly, there is considerable interindividual variability in LDL-c decrease with the same dose. Poor responses to statin therapy may be explained by genetic variability but can also result from poor adherence.

Meta-analyses of RCTs have shown that for every 39 mg/dL decrease in LDL-c, statins reduce all-cause mortality by 10% and confer a RR reduction of approximately 22% in CV events (eg, ACS, coronary death, need for myocardial revascularization, or stroke).^281^

Moreover, more potent statins lead to approximately 15% greater reductions in CV events compared to less intensive regimens.

Statins are therefore recommended as the first-line therapy for reducing CV risk in patients with dyslipidemia.

A lipid profile should be repeated approximately 4 weeks after starting statin therapy or after adjusting the dose. Once lipid targets are achieved, annual testing is generally sufficient unless there are suspected nonadherence or adverse effects.

Side effects are uncommon. Muscle-related symptoms are the most frequent. A recent meta-analysis of over 180 observational studies and RCTs found that the prevalence of statin-induced myopathy is 9%.^282^These symptoms usually occur within the first few weeks of treatment but may appear even years later. In most cases, they resolve within a few days of statin discontinuation. Symptoms range from myalgia, with or without creatine kinase (CK) elevation, to rhabdomyolysis. However, the risk of severe muscle injury is < 0.1%. CK should be assessed prior to initiating statin therapy, but routine monitoring is not recommended unless muscle symptoms arise (eg, pain, tenderness, stiffness, cramps, weakness, or localized/generalized fatigue) or there is concomitant use of interacting drugs.

Statins can generally be used in individuals with mild to moderate liver disease, as the risk of severe hepatotoxicity is very low. Baseline assessment of liver enzymes — particularly alanine aminotransferase (ALT) — is recommended before initiating therapy. Routine monitoring is unnecessary unless there are signs or symptoms suggestive of liver injury (eg, fatigue or weakness, appetite loss, abdominal pain, dark urine, or jaundice). Mild ALT elevations (up to 3× ULN [upper limit of normal]) occur in 0.5%-2.0% of statin users, more commonly with potent agents or higher doses. In such cases, therapy should not be discontinued, but liver enzymes should be reassessed in 4-6 weeks. On the other hand, if the levels of ALT rise to more than 3× ULN on two consecutive occasions, statin discontinuation or dose reduction is advised.

An increase in blood glucose and a higher risk of developing T2DM have been observed with statin use. A minor, clinically insignificant rise in HbA1c may also occur. This risk is greater with potent statins, higher doses, older age, and predisposing factors such as overweight or insulin resistance. It is estimated that 255 individuals would need to be treated with statins over 4 years for one additional case of diabetes to occur. Therefore, the absolute reduction in CV risk among high-risk patients clearly outweighs the potential adverse effect of a slight increase in diabetes incidence.^283^

### 7.2 Ezetimibe

Ezetimibe is a selective inhibitor of the NPC1L1 protein, which is responsible for intestinal cholesterol absorption. When combined with statins, it provides an additional LDL-c decrease by counteracting the compensatory increase in intestinal cholesterol absorption that occurs following inhibition of hepatic synthesis.

A RCT demonstrated that adding ezetimibe to simvastatin in patients with ACS decreased CV events.^165^A total of 18,144 patients with ACS were randomized to receive either simvastatin alone or in combination with ezetimibe and were followed for a median of 6 years. The combination led to a significant reduction in major CV events (HR, 0.936; 95% CI, 0.89-0.99; p = 0.016), proportional to the additional LDL-c decrease (about 17 mg/dL). The benefit was more pronounced in high-risk subgroups, such as patients with diabetes and older individuals.^284^In addition, ezetimibe is an effective, safe, low-cost alternative for patients with partial or complete statin intolerance. It is preferred over PCSK9 inhibitors in many clinical scenarios, particularly due to cost and adherence considerations.

The combination of a low dose of a potent statin (rosuvastatin 10 mg) with ezetimibe was shown to be noninferior to high-dose monotherapy with rosuvastatin 20 mg in reducing CV events in the RACING (RAndomized Comparison of Efficacy and Safety of Lipid-lowerING With Statin Monotherapy Versus Statin/Ezetimibe Combination for High-risk Cardiovascular Diseases) trial. Furthermore, the combination was associated with a lower discontinuation rate.^191^Studies support the good tolerability of combining ezetimibe with rosuvastatin.^285^

### 7.3 Novel Messenger RNA-Targeting Therapies

An innovative therapeutic modality targeting messenger RNA (mRNA) to reduce protein synthesis is already being used to suppress hepatic PCSK9 production and is currently being tested in clinical trials for other types of dyslipidemia, such as TG-rich lipoproteins and Lp(a) decrease. Compared to traditional therapies, mRNA-based treatments offer advantages such as specific inhibition of the target protein and long dosing intervals — up to 6 months. The two main drug classes that reduce protein production by targeting mRNA are:

1. Single-stranded ASOs;
2. Double-stranded siRNAs.

Both drug classes are administered parenterally, with cytoplasmic release into the target tissue and specific binding to a sequence within the target mRNA. This binding leads to degradation of the target mRNA, resulting in decreased translation of the encoded protein.

### 7.4 Anti-Proprotein Convertase Subtilisin/Kexin Type 9 Therapy

PCSK9 inhibitors were the first drug class capable of drastically reducing LDL-c levels in patients already using statins. The primary function of the PCSK9 protein is to degrade LDL receptors in hepatocytes. When PCSK9 is inhibited, intact LDL receptors return to the cell surface, allowing for greater LDL particle uptake from the circulation and consequently lowering the levels of LDL-c.

Among PCSK9 inhibitors, the monoclonal antibodies evolocumab and alirocumab — administered subcutaneously every 2-4 weeks — reduce LDL-c levels by approximately 55%-60% in statin users.^168 , 169^In two clinical trials involving patients with ASCVD already on statins, evolocumab and alirocumab reduced the rate of major CV events by 15%, with more pronounced benefits observed after the first year of treatment.^168 , 169^Patients who achieved extremely low LDL-c levels experienced even greater CV benefits.^198 , 286^No significant side effects or cognitive changes were identified over up to 8.4 years of follow-up, even in individuals with levels of LDL-c < 25 mg/dL.^203 , 286 , 287^

Despite these results, lipid-lowering therapies remain underused, highlighting the need for alternative strategies to improve adherence. In this context, inclisiran — a siRNA that inhibits the production of PCSK9 — offers improved dosing convenience, as it is administered subcutaneously every 6 months in a health care setting. Inclisiran provides a substantial and sustained LDL-c reduction of approximately 50%-55% in statin users, without significant side effects.^288^Ongoing studies aim to determine whether inclisiran use leads to CV risk reduction in patients with established ASCVD.

Although CV outcome trials for inclisiran are not yet published, its efficacy in sustainably reducing LDL-c supports its inclusion among anti-PCSK9 therapies. Because of the well-established causal relationship between LDL-c levels and CV risk, this drug class is considered a valid option for managing high-risk patients. Nevertheless, differences in the level of supporting evidence should be acknowledged.

Despite the proven efficacy and safety of anti-PCSK9 therapies, their high cost may limit widespread use, especially in Brazil. Therefore, it is suggested to prioritize PCSK9 inhibitor therapy in patients at high risk of ASCVD events or in those who are statin-intolerant.

### 7.5 Bempedoic Acid

BPA is a lipid-lowering agent used in the treatment of hypercholesterolemia, especially in patients who are statin-intolerant or require adjunctive therapies to achieve desired lipid goals.^289 , 290^It is the first oral drug in the class of hepatic *ACLY* inhibitors.^291^This agent stands out for its ability to inhibit cholesterol biosynthesis, acting similarly to statins but at an earlier metabolic step. By interfering earlier in the cholesterol synthesis pathway, BPA effectively decreases the levels of LDL-c. This reduction is achieved through upregulation of LDL receptor expression, a consequence of decreased intracellular cholesterol concentration.

One of the main advantages of BPA is its suitability for patients who are intolerant or inadequately responsive to statins, offering a viable and effective alternative for managing hypercholesterolemia.^292^Furthermore, its action at a distinct metabolic step may enhance the efficacy of combination therapies, providing broader control of lipid levels. These features make BPA a valuable component in dyslipidemia management strategies, particularly in cases requiring additional interventions to reach target levels.

The CLEAR Outcomes (Cholesterol Lowering via Bempedoic Acid, an ACL-Inhibiting Regimen) trial evaluated the efficacy of BPA in reducing CV risk in statin-intolerant patients.^293^That double-blind, placebo-controlled RCT included 13,970 patients who were unable or unwilling to take statins due to unacceptable adverse effects. Patients were assigned to receive BPA 180 mg daily or placebo. After a median follow-up of 40.6 months, BPA significantly decreased the levels of LDL-c compared to placebo, with a mean difference of 29.2 mg/dL. Moreover, the incidence of major adverse CV events was significantly lower in the BPA group compared to placebo (11.7% vs. 13.3%; HR, 0.87; 95% CI, 0.79-0.96; p = 0.004). The clinical benefit was proportional to the LDL-c decrease, mirroring findings from statin trials.

### 7.6 Cholesteryl Ester Transfer Protein Inhibitors and High-Density Lipoprotein Cholesterol–Raising Therapies

RCTs aimed at raising HDL-c levels have yielded predominantly neutral results. Cholesteryl ester transfer protein (CETP) inhibitors significantly increased HDL-c but failed to reduce CV events beyond what would be expected from the accompanying LDL-c decrease.^209 - 212^Similarly, niacin raised HDL-c levels but did not lower CV event rates.^213 , 214^More recently, obicetrapib, a next-generation, oral, low-dose, once-daily CETP inhibitor in development for the treatment of dyslipidemia, cardiovascular risk reduction, and Alzheimer's disease, has been reversing the tide of largely negative results for CETP inhibition and is on track to become the first CETP inhibitor available for clinical use. Unlike earlier compounds in this class, it has demonstrated significant reductions in LDL-c, non-HDL-c, ApoB, Lp(a), and small LDL particles, in addition to increases in functional HDL (pre-β HDL, ApoA-1, ApoE). In the BROADWAY phase 3 trial, in patients with ASCVD or HeFH on maximally tolerated lipid-lowering therapy, it reduced LDL-c by approximately 30%.^294^The BROOKLYN phase 3 trial showed LDL-C reductions of 36–41% in HeFH, with more than 50% of patients achieving LDL-c <70 mg/Dl.^295^The ongoing PREVAIL phase 3 trial is evaluating the impact on major cardiovascular outcomes in over 9,500 patients with ASCVD and uncontrolled LDL-c.

More recently, an intervention designed to improve HDL function — rather than simply increase HDL-c concentrations — also failed to demonstrate CV benefit.^296^There is currently no evidence that raising HDL-c reduces CV event rates.

### 7.7 Fibrates

Fibrates activate peroxisome proliferator–activated receptor alpha (PPAR-α), which increases the production and activity of LPL — the enzyme responsible for intravascular hydrolysis of TG — and reduces ApoC-III, which normally inhibits LPL. Fibrate therapy also increases the synthesis of ApoA-I, leading to higher HDL concentrations. The levels of TG are typically reduced by 30%-60% with the use of fibrates.

Although two early trials conducted prior to widespread statin use — the Helsinki Heart Study^297^and the VA-HIT (Veterans Affairs High-Density Lipoprotein Cholesterol Intervention Trial) trial^298^— have demonstrated significant reductions in CV risk with gemfibrozil, similar benefits were not observed with fenofibrate in the FIELD (Fenofibrate Intervention and Event Lowering in Diabetes)^299^and ACCORD (Action to Control Cardiovascular Risk in Diabetes)^300^trials or with bezafibrate in the BIP (Bezafibrate Infarction Prevention) trial.^301^The ACCORD trial evaluated fenofibrate versus placebo in a population of patients with dyslipidemia and diabetes already on simvastatin therapy and found no significant reduction in fatal CV events, MI, or stroke.^300^However, a subgroup analysis of 941 patients with HTG (TG ≥ 204 mg/dL) and low HDL-c (< 35 mg/dL) showed a reduction in CV events among those receiving fibrates. Other studies have also shown benefits in this specific subgroup with high TG and low HDL-c. Nevertheless, an RCT targeting this population was needed.

The PROMINENT (Pemafibrate to Reduce Cardiovascular Outcomes by Reducing Triglycerides in Patients With Diabetes) trial assessed the use of pemafibrate — a more potent fibrate for reducing TG and increasing HDL-c — for the prevention of CV outcomes in over 10,000 patients with diabetes, TG levels between 200-499 mg/dL, and HDL-c ≤ 40 mg/dL. More than 95% of patients were on statin therapy, most receiving high-intensity treatment. Despite improvements in TG and other lipoproteins, pemafibrate did not significantly reduce CV events compared to placebo.^302^Therefore, despite findings from post hoc or secondary analyses of previous fibrate trials, the PROMINENT trial clearly demonstrated that fibrates are not formally indicated for reducing CV risk in patients at high risk already receiving statins.

On the other hand, fibrates remain first-line treatment for patients with TG ≥ 500 mg/dL, with the primary goal of reducing the risk of acute pancreatitis. In this population, TG decreases with fibrates may be substantial, and treatment should be initiated even in the absence of robust CV benefit. Fenofibrate is preferred when used in combination with statins due to its lower risk of myopathy. The combination with gemfibrozil is contraindicated because of the high incidence of severe muscle-related adverse effects such as rhabdomyolysis.^303^

### 7.8 Omega-3 Fatty Acids

Omega-3 fatty acids, particularly EPA and DHA, have been extensively studied for their ability to lower blood levels of TG. However, the clinical benefits of these fatty acids vary depending on the population studied, the dosage used, and the specific formulation administered.

The REDUCE-IT (Reduction of Cardiovascular Events with Icosapent Ethyl–Intervention Trial) trial demonstrated that icosapent ethyl (IPE), a purified form of EPA, significantly reduced major CV events in patients with established CVD or T2DM who were on statin therapy and had fasting TG levels between 135 and 499 mg/dL.^304^In that study, the decrease in CV risk was disproportionate to the TG lowering, suggesting an alternative mechanism of benefit. The 4 g/day IPE formulation used in the trial is not available in Brazil.

In contrast, the STRENGTH (STatin Residual Risk Reduction with EpaNova in HiGh Cardiovascular Risk PatienTs with Hypertriglyceridemia) trial, which evaluated a combination of EPA and DHA in patients at high CV risk, did not show a significant reduction in CV events.^305^This discrepancy has sparked debate over differences between purified versus mixed formulations. Other trials using generic EPA/DHA supplements have shown inconsistent results regarding CV outcomes, despite effectively lowering TG levels.^306 - 310^

For patients with TG ≥ 500 mg/dL — who were not included in the REDUCE-IT trial — omega-3 therapy is primarily supported for reducing the risk of pancreatitis. Previous studies have shown that daily doses of 2 to 4 g of EPA plus DHA can reduce TG levels by up to 45% in this population. Although the direct CV benefits remain unclear in this group, guidelines from the American Heart Association and the Endocrine Society recommend omega-3s as part of a comprehensive strategy for lipid-lowering and metabolic complication prevention.^311 , 312^

Overall, omega-3s have a favorable safety profile, with adverse effects generally mild, including gastrointestinal discomfort and a fishy aftertaste. However, they may exert antiplatelet effects, requiring caution when combined with other antithrombotic therapies, and have been associated with an increased risk of atrial fibrillation.

### 7.9 Apolipoprotein C-III Inhibitors

ApoC-III is a glycoprotein that plays a key role in the regulation of TG levels. ApoC-III inhibits the activity of LPL, the enzyme responsible for TG hydrolysis, and reduces hepatic clearance of TG-rich lipoproteins, resulting in increased serum TD levels. Loss-of-function variants in the gene encoding ApoC-III have been associated with decreased TG levels and lower CV risk.^313 , 314^

Volanesorsen, an ASO, acts on mRNA to prevent ApoC-III protein synthesis, thereby lowering TG levels. In patients with TG ≥ 500 mg/dL of various etiologies, volanesorsen reduced the risk of pancreatitis by 82% and yielded a relative reduction in hepatic steatosis of 24%-53%.^315 , 316^Despite these promising results, thrombocytopenia emerged as a frequent adverse event.^317^Currently, the drug is approved in Brazil for adults with FCS, a condition characterized by mutations in genes related to LPL.

More recently, olezarsen, a next-generation ASO, also demonstrated efficacy in patients with FCS, reducing TG levels by up to 44% compared to placebo and lowering the risk of pancreatitis by 88%^318^In patients with predominantly moderate HTG, olezarsen decreased TG levels by 49%-53% compared to placebo.^319^

Plozasiran, a siRNA that blocks the synthesis of ApoC-III, lowered TG levels by up to 62% versus placebo in patients with moderate to severe HTG and reduced the risk of pancreatitis by 83% in patients with chylomicronemia.^320 - 322^In early trials, adverse events associated with olezarsen or plozasiran were uncommon and included mild injection site reactions, slight transaminase elevation, and — in the case of plozasiran — hyperglycemia. Thrombocytopenia was not reported as a treatment-related adverse event. Both agents are still under investigation and not yet approved for use in Brazil.

### 7.10 Angiopoietin-Like Protein 3 Inhibitors

Angiopoietin-like protein 3 (ANGPTL3) is an endogenous inhibitor of LPL. Members of the ANGPTL protein family are recognized as important regulators of lipoprotein metabolism and have been investigated as pharmacological targets for the reduction of ASCVD risk. Loss-of-function mutations in ANGPTL3 are associated with reduced risk of CAD, while gain-of-function mutations are linked to increased CAD risk.^323^ANGPTL3 deficiency leads to reductions in TG, LDL-c, and HDL-c. Importantly, LDL-c reduction occurs independently of the LDL receptor.

Evinacumab is a monoclonal antibody that inhibits ANGPTL3 to reduce the concentration of TG-rich remnant lipoproteins with atherogenic potential. Because it does not rely on LDL receptor function, evinacumab can be used as adjunctive therapy for decreasing LDL-c in patients with HoFH^324 , 325^— a rare genetic condition marked by extremely high levels of LDL-c from childhood and a markedly increased risk of premature CVD. Accordingly, evinacumab is approved for use in adults and pediatric patients aged 5 years and older with HoFH who do not achieve LDL-c targets despite maximally tolerated lipid-lowering therapy. The recommended dose is 15 mg per kilogram of body weight, administered via intravenous infusion over 60 minutes every four weeks. The most common side effects include flu-like symptoms (eg, fever and headache), infusion-site reactions, and gastrointestinal complaints.

A recent trial of an ANGPTL3-targeting ASO was halted due to increased liver enzymes and hepatic fat accumulation.^326^Three additional studies investigating siRNA therapies are currently ongoing.

### 7.11 Lipoprotein(a) Inhibitors

Apo(a) is a component of Lp(a), which has been associated with MI, stroke, and aortic valve stenosis. Currently, no specific treatment is available to decrease the levels of Lp(a). New therapies offer hope for patients with significantly elevated Lp(a) — a subgroup for whom no targeted treatments currently exist. The main strategies under development are RNA-silencing therapies, especially ASOs and siRNAs, designed to decrease hepatic production of Apo(a).

### 7.12 Clustered Regularly Interspaced Short Palindromic Repeats and Gene Therapies

Gene-editing technologies such as clustered regularly interspaced short palindromic repeats (CRISPR)-Cas9 are being explored as potential "genetic cures" for hereditary dyslipidemias. In animal models, a single CRISPR-based edit targeting the PCSK9 gene resulted in sustained LDL-c decreases (> 60%) for over 1 year. Although still experimental, this approach could revolutionize the treatment of lipid disorders.

**Table:** 

Recommendation	Strength of Recommendation	Certainty of Evidence
In individuals eligible for lipid-lowering therapy, statins are recommended as the first-line treatment option.	STRONG	HIGH
In individuals with an LDL-c reduction target of ≥ 50%, combination therapy with a high-intensity statin and ezetimibe is recommended as an alternative.	STRONG	HIGH
In individuals who do not reach their target despite maximum tolerated statin therapy, treatment intensification with ezetimibe or anti-PCSK9 therapy is recommended.	STRONG	HIGH
Inclisiran is recommended as an alternative to monoclonal anti-PCSK9 antibodies, such as evolocumab or alirocumab.	STRONG	MODERATE
In individuals intolerant to statins and not at goal despite ezetimibe, therapeutic intensification with BPA is recommended.	STRONG	HIGH
In individuals diagnosed with homozygous familial hypercholesterolemia who do not achieve LDL-c targets despite receiving the maximum tolerated doses of lipid-lowering therapies, the use of evinacumab is recommended from the age of 5 years.	STRONG	MODERATE
Pharmacologic treatment aimed at increasing HDL-c is not recommended.	STRONG	HIGH
In individuals with HTG (≥ 150 mg/dL) eligible for lipid-lowering therapy to reduce CV risk, statins are recommended as the preferred first-line treatment.	STRONG	HIGH
In individuals with triglycerides between 150 and 499 mg/dL and established ASCVD or at high cardiovascular risk, the use of IPE (4 g/day) is weakly/conditionally recommended to reduce major CV events, although it is not available in Brazil	CONDITIONAL	MODERATE
In individuals with TG between 150 and 499 mg/dL who have ASCVD or are at high CV risk, EPA plus DHA formulations are not recommended for preventing CV events.	STRONG	HIGH
In individuals with persistently elevated TG (≥ 500 mg/dL) despite lifestyle interventions, the use of fibrates is recommended to reduce the risk of pancreatitis.	STRONG	MODERATE
In adults with Familial Chylomicronemia Syndrome (FCS) and triglyceride levels ≥ 500 mg/dL, the use of volanesorsen is recommended to reduce the risk of pancreatitis.	STRONG	MODERATE

ASCVD: atherosclerotic cardiovascular disease; BPA: bempedoic acid; CV: cardiovascular; IPE: icosapent ethyl; TG: triglycerides; LDL-c

### 7.13 Combination Therapy

The standard approach to achieving LDL-c targets typically involves a stepwise strategy with lipid-lowering therapies: treatment begins with a statin — usually a high-intensity statin at the highest recommended or tolerated dose — followed by sequential addition of ezetimibe and if necessary PCSK9 inhibitors. However, there remains a significant gap between guideline recommendations and clinical practice, with registry data showing low rates of LDL-c control, therapeutic inertia, and poor adherence to treatment.^25 , 327^In response to this challenge, the treatment paradigm has shifted from the concept of high-intensity statins to that of high-intensity lipid-lowering therapy, emphasizing combination therapy from the outset.^328 , 329^This approach aims to reduce CV risk more rapidly and effectively, particularly in individuals at very high CV risk.

Studies have shown that the efficacy in lowering LDL-c can be significantly enhanced through the use of combination lipid-lowering therapies:

- **Hig** **h** **-intensity statin + ezetimibe:** LDL-c reduction of up to 65%, representing a widely recommended strategy for high-risk patients.
- **High-intensity statin + ezetimibe + PCSK9 inhibitor:** LDL-c reductions exceeding 85%, currently the most potent regimen available.
- **Moderate-intensity statin + ezetimibe:** When high-intensity statins are not tolerated, the combination of a moderate-intensity statin with ezetimibe may achieve an additional 20–25% reduction.
- **Moderate-intensity statin + bempedoic acid + ezetimibe:** An effective alternative for patients intolerant to high-intensity statins, with LDL-c reductions of up to 60%.
- **Bempedoic acid + ezetimibe + PCSK9 inhibitor:** A non-statin strategy that may achieve LDL-c reductions greater than 75%, particularly useful in cases of statin intolerance or contraindication.

Other therapeutic combinations are also possible, as illustrated in Figure 7.2 , which presents various lipid-lowering regimens tailored to clinical needs and patient tolerance profiles.

**Figure 7.2:** Efficacy of lipid-lowering therapy in LDL-c reduction.^78^MIS: moderate-intensity statin; HIS: high-intensity statin; EZE: ezetimibe; BA: bempedoic acid; anti-PCSK9: anti-proprotein convertase subtilisin/kexin type 9 therapy. *Approximate values.

These combinations reflect the concept of high-intensity lipid-lowering therapy, which emphasizes synergistic pharmacologic interventions from the outset, aiming to overcome clinical barriers and optimize lipid control in high-risk populations.

#### 7.13.1 Benefits of Combination Therapy

Registry data show that most patients still fail to achieve recommended LDL-c targets despite the availability of a broad range of therapeutic options.^25 , 27 , 28 , 327 , 330^In the REACT study, which included 2,364 patients at high CV risk in Brazil, 77% were on statins, yet 20%-30% had LDL-c levels ≥ 100 mg/dL.^331^

#### 7.13.2. Statin and Ezetimibe Combination

The IMPROVE-IT trial showed that adding ezetimibe 10 mg to simvastatin 40 mg resulted in an additional 24% reduction in LDL-c levels and a proportional 7.2% reduction in major CV events compared to statin monotherapy over 7 years. On average, high-intensity statin monotherapy reduces LDL-c by approximately 50%; when combined with ezetimibe, this reduction increases to about 65%.^332^

A simulation analysis of the DA VINCI study, which included 2,482 patients who had not reached LDL-c goals, showed that statin monotherapy optimization would not be sufficient for most patients to achieve targets. However, the addition of ezetimibe would double the proportion of individuals reaching their LDL-c targets, regardless of their CV risk category.^333^

Supporting this evidence, the RACING trial demonstrated that combining rosuvastatin 10 mg with ezetimibe was noninferior to rosuvastatin 20 mg monotherapy in reducing major CV outcomes. Among these two high-intensity lipid-lowering strategies — one using a moderate-intensity statin plus ezetimibe and the other using high-intensity statin monotherapy — the combination approach led to greater LDL-c goal achievement over time and lower treatment discontinuation rates.^191^

#### 7.13.3 Statin and Proprotein Convertase Subtilisin/Kexin Type 9-Targeted Therapy Combination

In the FOURIER trial, evolocumab, a PCSK9-targeted agent, reduced LDL-c by 59% and the risk of major CV events by 15% over a 26-month follow-up in patients already receiving optimized statin doses.^168^In the ODYSSEY OUTCOMES trial, alirocumab decreased LDL-c by up to 62.7% and major CV events by 15% in patients receiving high-intensity statin therapy.^169^

More recently, an implementation strategy using inclisiran — administered immediately after failure to achieve LDL-c < 70 mg/dL despite maximally tolerated statin therapy — showed superior effectiveness compared to usual care: 81.8% of patients reached LDL-c < 70 mg/dL and 71.6% achieved LDL-c < 55 mg/dL, compared to 22.2% and 8.9%, respectively, in the usual care group.^3334^

#### 7.13.4. Ezetimibe and Bempedoic Acid Combination

In a phase 3 randomized controlled trial (RCT) involving patients at high CV risk (about 65% previously on statins), the combination of ezetimibe and bempedoic acid reduced LDL-c levels by 38%, compared to reductions of 17% with bempedoic acid alone and 23% with ezetimibe alone.^335^

#### 7.13.5. Statin, Ezetimibe, and Proprotein Convertase Subtilisin/Kexin Type 9-Targeted Therapy Combination

The combined use of high-intensity statins, ezetimibe, and PCSK9-targeted therapy can lead to an approximate 85% reduction in LDL-c levels. This strategy may be particularly suitable for patients at very high or extreme risk, or for those with severe hypercholesterolemia who fail to reach LDL-c goals with dual therapy.

#### 7.13.6. Statin, Ezetimibe, and Bempedoic Acid Combination

In a simulation analysis involving 105,577 patients at high (n = 28,677) or very high (n = 76,900) CV risk, 88% were on statin monotherapy. The sequential addition of ezetimibe and BPA significantly increased LDL-c target attainment rates: from 11.2% with statin alone to 33.1% with statin plus ezetimibe, and to 61.9% with the further addition of BPA.^336^

**Table:** 

Recommendation	Strength of Recommendation	Certainty of Evidence
In individuals at high CV risk, initial therapy with high-intensity statin and ezetimibe is recommended to achieve therapeutic targets.	STRONG	HIGH
In individuals at very high CV risk, initial therapy with high-intensity statin and ezetimibe, and potentially PCSK9 inhibitor therapy, is recommended to achieve therapeutic targets.	STRONG	HIGH
In individuals at extreme CV risk, initial therapy with high-intensity statin, ezetimibe, and PCSK9 inhibitor therapy is recommended to achieve therapeutic targets.	STRONG	HIGH
In individuals who do not reach therapeutic targets with high-intensity statin and ezetimibe, PCSK9 inhibitor therapy and/or BPA are recommended according to treatment goals.	STRONG	HIGH
In individuals with FH and LDL-c ≥ 190 mg/dL, initial therapy with high-intensity statin plus ezetimibe is recommended to achieve therapeutic targets.	STRONG	HIGH
In individuals with statin intolerance, a personalized combination strategy is recommended (eg, BPA combined with a PCSK9 inhibitor or with ezetimibe) to achieve therapeutic targets.	STRONG	HIGH

BPA: bempedoic acid; CV: cardiovascular; FH: familial hypercholesterolemia; PCSK9: proprotein convertase subtilisin/kexin type 9.

## 8. Management of Statin Intolerance

Statin intolerance typically presents as muscle-related complaints, which may include pain, weakness, or cramps. This issue is clinically relevant due to the association between nonadherence or discontinuation of statin therapy and an increased risk of CVD.

### 8.1 Definition

Statin intolerance is defined as the inability to tolerate various statins, at any dose, due to the onset of symptoms and/or laboratory abnormalities, requiring treatment discontinuation. Symptoms and/or laboratory abnormalities must resolve upon statin withdrawal and recur upon rechallenge with the same or another statin. These effects should not be attributable to drug interactions or to conditions known to increase the likelihood of statin intolerance.

### 8.2 Prevalence

According to a meta-analysis,^282^the prevalence of statin intolerance is significantly lower in RCTs than in cohort studies (4%-21%). Higher prevalence rates have been observed in populations under primary prevention compared to secondary prevention, and among older individuals, women, Asians, and Black individuals as well as in those with obesity, diabetes, hypothyroidism, liver disease, renal dysfunction, concomitant use of antiarrhythmic drugs or calcium channel blockers, and with increasing statin doses.^282^The conclusion of this analysis, which included over 4 million patients, suggests that the prevalence of statin intolerance is frequently overestimated and highlights the need for careful evaluation of patients with suspected statin-related symptoms.^282^

### 8.3 Diagnosis

Several diagnostic criteria for statin intolerance have been proposed; however, they all rely on clinical judgment and laboratory tests.^337^To date, no sensitive or specific biomarkers have been identified. Therefore, diagnosis requires the exclusion of other possible causes and a clear causal relationship between statin use and adverse effects.^337^In cases of suspected intolerance, four key elements must be identified:^337^

1. **Clinical presentation:** includes subjective symptoms such as myalgia, muscle weakness, or cramps, and/or abnormal lab findings (CK, AST, or ALT);
2. **Type and dose of statin:** patients must be unable to tolerate at least two different statins, one of which at the lowest daily dose (eg, atorvastatin 10 mg, rosuvastatin 5 mg, simvastatin 5 mg, pitavastatin 1 mg, pravastatin 10 mg, fluvastatin 20 mg, lovastatin 20 mg);
3. **Timing and causality:** adverse reactions must occur after statin initiation or dose escalation, improve upon discontinuation, and reappear upon rechallenge;
4. **Exclusion of other causes:** the likelihood that adverse reactions are due to other diseases or drug interactions must be low.

### 8.4 Nocebo Effect

The nocebo effect refers to the manifestation of adverse symptoms that are not pharmacologically attributable to treatment but rather arise from negative patient expectations. In the context of statins, this effect can lead to premature treatment discontinuation. The etiology of the nocebo effect is linked to the anticipation of harm triggered by various factors, such as the prescriber's communication about potential side effects, detailed informed consent procedures in clinical trials, exposure to nonclinical information sources (eg, the internet or social media), and observing symptoms in other patients.^338^

### 8.5 Muscle Symptoms

The term statin-associated muscle symptoms (SAMS) is commonly used to refer to muscle-related symptoms linked to statin use, such as pain, cramps, or weakness. However, this terminology does not necessarily imply a causal relationship with statin therapy. Muscle symptoms are typically bilateral, symmetrical, and limited to skeletal muscles.^339^

#### 8.5.1. Clinical Characteristics, Classification, and Management of Statin-Associated Muscle Symptoms

The clinical management of SAMS should be guided by both the presence of muscle symptoms and CK elevation, following the seven patterns of SAMS outlined in the current guideline. These patterns range from asymptomatic CK elevation up to 3× ULN ( **SAMS 0** ), to tolerable ( **SAMS 1** ) and intolerable myalgia ( **SAMS 2** ), moderate ( **SAMS 3** ) and severe myopathy ( **SAMS 4** ), rhabdomyolysis ( **SAMS 5** ), and immune-mediated necrotizing myositis ( **SAMS 6** ). Recognizing the distinct phenotypes and severity levels helps facilitate practical clinical management ( Figures 8.1 and 8.2 ).

**Figure 8.1:** Flowchart for the investigation and probable diagnosis of SAMS. ANA: antinuclear antibodies; BPA: bempedoic acid; CK: creatine kinase; ESR: erythrocyte sedimentation rate; HDL-c: LDL-c: low-density lipoprotein cholesterol; PCSK9: proprotein convertase subtilisin/kexin type 9; ULN: upper limit of normal; T4: thyroxine; TSH: thyroid-stimulating hormone.

**Figure 8.2:** Flowchart for the specific diagnosis and management of SAMS. ANA: antinuclear antibodies; CK: creatine kinase; ESR: erythrocyte sedimentation rate; HMG-CoA: 3-hydroxy-3-methylglutaryl-coenzyme A; HMGCR: 3-hydroxy-3-methylglutaryl-coenzyme A reductase; LDL-c: low-density lipoprotein cholesterol; PCSK9: proprotein convertase subtilisin/kexin type 9; SAMS: statin-associated muscle symptoms; T4: thyroxine; TSH: thyroid-stimulating hormone; ULN: upper limit of normal..

When assessing a potential SAMS case, it is important to (i) acknowledge all muscle complaints (pain, weakness, or cramps), not just pain, and to consider previous muscle symptoms, comorbidities, and concomitant medications; (ii) recognize the usual time frame between statin initiation and symptom onset, typically 4 to 12 weeks, though symptoms may rarely occur after more than a year or emerge abruptly after dose escalation or the introduction of a drug or food that affects pharmacokinetics;^340^(iii) understand that the pattern of muscle pain and weakness is usually symmetrical and proximal, affecting large muscle groups such as the gluteal muscles, thighs, calves, and dorsal musculature. Muscle complaints are more frequent in physically active individuals.

In the presence of intolerable muscle pain, serum CK levels should be measured immediately. If CK levels exceed 7× ULN or remain persistently above 3× ULN, thyroid function (TSH, free T4), erythrocyte sedimentation rate (ESR), and antinuclear factor (ANF) should also be assessed. In cases of intolerable symptoms, serum urea, creatinine, and myoglobinuria should also be tested. If a secondary cause that could explain SAMS is identified, it should be addressed, and the statin should be reintroduced at a low dose with gradual titration.

#### 8.5.2. Tolerable and Intolerable Muscle Symptoms

Most often, muscle complaints occur without substantial CK elevation.^341^The most important step in such cases is to determine symptom tolerability, as the impact can vary widely among individuals, especially in those with comorbidities such as hypothyroidism, collagen diseases, and fibromyalgia.

In cases of tolerable muscle pain with or without CK elevation ( **SAMS 0** ) or with elevation up to 3× AND 7^x^ULN ( **SAMS 1** ), temporary dose reduction or statin switching may be considered, but no additional concern is warranted ( Figure 8.1 ). If CK levels are elevated between 3× and 7× ULN with tolerable symptoms, dose reduction and closer CK monitoring are required ( Figure 8.2 ). In the presence of intolerable symptoms and CK elevation ( **SAMS 2** ), statin discontinuation becomes necessary and should prompt a broader diagnostic investigation ( Figures 8.1 and 8.2 ). Newly occurring muscle complaints meeting the clinical suspicion for SAMS should therefore trigger a CK test request. Overall, the less severe forms of SAMS ( **SAMS 0 to 4** ) are self-limiting and do not result in permanent damage.

#### 8.5.3. Creatine Kinase Elevation

During statin therapy, transient elevations in CK may occur even in asymptomatic patients, but they are generally not clinically significant. Thus, routine CK monitoring during statin therapy is not recommended, except when a new medication is introduced or the statin dose is increased. Among patients requiring new CK testing, those who are asymptomatic with mild CK elevations (< 3× ULN) ( **SAMS 0** ) do not need to discontinue or adjust their statin regimen (see Flowcharts 1 and 2 ). In asymptomatic patients with CK elevations between 3× and 7× ULN ( **SAMS 1** ), treatment interruption is not required; the same or a different statin may be reinitiated at a low dose, with dose titration every 4-6 weeks.

Similarly, the current Brazilian Society of Cardiology (SBC) guideline recommends temporary discontinuation of statin therapy and periodic CK monitoring every 4-6 weeks if CK levels are between 3× and 7× ULN in the presence of intolerable muscle symptoms ( **SAMS 2** ). For those with CK levels between 3 and 7× ULN and tolerable or no symptoms **(SAMS 1)** , switching to a low-intensity statin regimen and closer CK monitoring every 4–6 weeks is recommended.

However, regardless of the presence of symptoms, if CK levels exceed 7× ULN, statin therapy should be discontinued for 4-6 weeks, followed by repeat CK measurement and reassessment. If CK does not decrease < 7× ULN within 6 weeks off statin therapy ( **SAMS 4 or SAMS 6** ), the patient should be evaluated for secondary causes through detailed clinical and laboratory investigations, including assessment of renal function, thyroid hormones (TSH, free T4), erythrocyte sedimentation rate (ESR), and antinuclear factor (ANF). If no pain improvement occurs after statin discontinuation and no associated causes are found, differential diagnosis between immune-mediated myopathy ( **SAMS 6** ) and non-statin-related myopathy should be considered.

In cases where serum CK levels decrease after discontinuation but rise again to > 7x ULN during statin reintroduction or dose titration (after excluding secondary causes such as exercise, hypothyroidism, or metabolic myopathies), a lower dose of the same statin or an alternative statin should be used. Subsequently, non-statin lipid-lowering therapy should be added to achieve the lowest possible level of LDL-c relative to the target goal.

#### 8.5.4. Rhabdomyolysis

Rhabdomyolysis ( **SAMS 5** ) is the most severe muscle-related adverse event associated with statin therapy. It may lead to muscle necrosis, serious electrolyte disturbances, acute kidney injury, coagulopathy, shock, and death. The most observed diagnostic criterion is muscle pain accompanied by a sudden increase in CK levels > 10× ULN. However, in rarer cases, rhabdomyolysis may present as muscle weakness or mild symptoms with CK elevations exceeding 40-50× ULN. Therefore, for diagnostic purposes, rhabdomyolysis is defined as either an asymptomatic CK elevation > 50× ULN or muscle pain with CK > 10× ULN combined with renal dysfunction (defined as a rise in serum creatinine ≥ 0.5 mg/dL) and myoglobinuria.

#### 8.5.5. Statin-Induced Immune-Mediated Necrotizing Myopathy

Statin-associated immune-mediated necrotizing myopathy ( **SAMS 6** ) is typically related to the presence of serum autoantibodies directed against 3-hydroxy-3-methylglutaryl-coenzyme A reductase (HMGCR), the pharmacological target of statins. In most cases of statin-associated myopathy, symptoms resolve after drug discontinuation. However, in exceptionally rare instances, an autoimmune myopathy may develop. On physical examination, patients typically report muscle weakness, such as difficulty rising from a chair, climbing stairs, or lifting heavy objects. Laboratory findings reveal markedly elevated and persistent CK levels (usually > 2,000 U/L) despite statin withdrawal.

Diagnosis is established by detecting anti-HMGCR autoantibodies in the context of prior statin exposure, muscle symptoms, and persistent CK elevation.^342 , 343^Muscle biopsy typically shows myocyte necrosis and inflammatory infiltration.^343^Treatment includes corticosteroids and immunosuppressive therapy.^343^Unlike other forms of SAMS, symptoms in **SAMS 6** do not improve after statin discontinuation. However, since statin exposure is the underlying trigger, immediate discontinuation remains essential.

### 8.6. Factors Associated with Statin Intolerance

Numerous factors, both pharmacological and patient-related,^343^may contribute to the development of statin-associated adverse effects. Some are listed in Table 8.1 . Table 8.2 presents key drug interactions that may exacerbate or trigger muscle symptoms in statin users.

**Table 8.1:** Factors Associated with Statin Intolerance

Risk Factor	Description and Pathophysiological Rationale	Clinical Implications and Management
**A. Advanced age**	Aging leads to a physiological decline in vital organ function. Reduced renal eGFR and decreased efficiency of hepatic metabolism (cytochrome P450 enzymes) alter statin pharmacokinetics, increasing plasma concentration and drug exposure time.	Start with lower doses ("start low, go slow"). Monitor renal (creatinine, eGFR) and liver function. Carefully assess polypharmacy, which is common in this population.
**B. Women**	Women generally have lower body mass and distribution volume than men, which can result in higher plasma statin concentrations at the same dose. Hormonal factors may also influence metabolism and muscle sensitivity.	Consider dose adjustment based on body weight and mass. Be alert to even mild muscle complaints.
**C. Asian ethnicity**	East Asian populations (eg, Chinese and Japanese) show a higher prevalence of genetic polymorphisms in hepatic transporters (eg, *OATP1B1* , which is encoded by the *SLCO1B1* gene) and metabolic enzymes. This reduces hepatic uptake and/or statin clearance, increasing systemic exposure.	Use conservative starting doses, especially for rosuvastatin and atorvastatin. The recommended starting dose of rosuvastatin is 5 mg. Titrate cautiously.
**D. Hypothyroidism**	Uncontrolled hypothyroidism itself causes myopathy, with symptoms such as muscle pain, cramps, and elevated CK. It may also reduce statin metabolism, enhancing toxicity. Symptoms may overlap and cause confusion.	Always assess TSH before starting statin therapy and in patients who develop muscle symptoms. Treating thyroid dysfunction may resolve symptoms.
**E. Alcohol consumption**	Chronic excessive alcohol intake may cause alcoholic myopathy and burdens hepatic metabolism. This increases muscle vulnerability to statins and impairs drug metabolism.	Assess alcohol intake history. Advise moderation or cessation. Monitor liver enzymes (AST/ALT) and CK in patients with risky alcohol use.
**F. Neuromuscular diseases**	Patients with pre-existing neuromuscular diseases (eg, ALS, dystrophies) have reduced muscle reserve and may experience worsening or unmasking of symptoms with statin use.	Statin use must be extremely cautious. Decisions should be shared with a neurologist, carefully weighing CV benefits and risks.
**G. CKD**	Renal insufficiency (especially eGFR < 30-60 mL/min) significantly reduces clearance of hydrophilic statins (pravastatin, rosuvastatin) and their metabolites, increasing the risk of accumulation and toxicity.	Adjust statin dose based on eGFR. Atorvastatin and fluvastatin are safer options due to lower renal elimination.
**H. Liver disease**	The liver is the main site of statin metabolism. Active liver disease or hepatic failure impairs this process, increasing serum drug levels.	Statins are contraindicated in active or decompensated liver disease. In stable chronic liver disease, use cautiously at low doses with transaminase monitoring.
**I. Excessive physical exercise**	Strenuous or unaccustomed exercise causes muscle microtrauma and CK elevation. This may be confused with SAMS or, rarely,	Advise the patient to start exercise programs gradually. In case of symptoms after intense exercise, consider interrupting the statin and reassessing CK after recovery.
**J. Personal/family history**	A previous history of statin intolerance increases the likelihood of intolerance to another. Family history suggests a genetic predisposition to SAMS, such as polymorphisms in metabolizing genes.	Consider the use of lower doses and statins with a lower risk of myopathy (e.g., pravastatin and fluvastatin) or non-statin therapies. Reintroduction should be performed with caution.
**K. Low body mass**	Similar to the "female sex" factor, individuals with low body weight or frailty (sarcopenia) have a lower volume of distribution for the drug, which leads to higher plasma concentrations and an increased risk of toxicity.	Dosing should be individualized and conservative. High doses of high-potency statins should be avoided.
**L. SLCO1B1 polymorphism**	The SLCO1B1 gene encodes the organic anion transporter OATP1B1, which is responsible for the hepatic uptake of statins. Reduced-function polymorphisms (genetic variants) decrease this uptake, increasing statin concentrations in the blood and muscle.	This is the main genetic predictor of simvastatin-induced myopathy, but it also affects other statins. In practice, genetic testing is not routine, but suspicion increases with a positive family history or intolerance to multiple statins.
**M. High statin dose**	The risk of SAMS is directly proportional to the dose and potency of the statin. High doses (e.g., simvastatin 80 mg – contraindicated; atorvastatin 80 mg; rosuvastatin 40 mg) significantly increase systemic exposure to the drug.	Use the lowest effective dose to achieve the LDL-c target. Consider combination with a non-statin agent (e.g., ezetimibe) to avoid the need for high statin doses.
**N. Nocebo effect**	The widespread dissemination of negative information about statins in the media and on the internet may create an expectation of adverse effects in the patient. This nocebo effect may cause the patient to attribute any muscle pain to the medication or even induce real symptoms.	Validate the patient's complaint. Provide clear, evidence-based information on the actual risks and the substantial benefits of therapy. In studies, many patients who report SAMS in open-label arms do not present symptoms in double-blind trials.
**O. Drug interactions**	Many drugs inhibit cytochrome P450 enzymes (mainly CYP3A4), which are responsible for the metabolism of atorvastatin, lovastatin, and simvastatin. Concomitant use drastically increases statin levels. Examples include azole antifungals, macrolide antibiotics, fibrates (especially gemfibrozil), protease inhibitors, and amiodarone.	Perform a complete review of the patient's prescription before initiating or adjusting statin therapy. Opt for statins with different metabolic pathways (pravastatin, rosuvastatin, and fluvastatin) if the interaction is unavoidable. Consult drug interaction tables.

ALS: amyotrophic lateral sclerosis; ALT: alanine aminotransferase; AST: aspartate aminotransferase; CK: creatine kinase; CKD: chronic kidney disease; eGFR: estimated glomerular filtration rate; LDL-c: low-density lipoprotein cholesterol; SAMS: statin-associated muscle symptoms; TSH: thyroid-stimulating hormone.

**Table 8.2:** FDrug Interaction Table for Statins

Interacting Drug Class	Mechanism of Interaction and Affected Statins	Clinical Risk and Management Recommendations
**1. Anticoagulants**	Warfarin: Inhibition of CYP2C9 (fluvastatin, rosuvastatin) or displacement from protein binding sites (lovastatin).^354^DOACs: No clinically significant interactions.^355^	Warfarin: Increases bleeding risk. Intensive INR monitoring is mandatory when starting or adjusting most statins (exception: pravastatin). DOACs (eg, dabigatran, apixaban, rivaroxaban, and edoxaban): can be safely coadministered.
**2. Azole antifungals**	Potent inhibition of CYP3A4, blocking metabolism of simvastatin, lovastatin, and atorvastatin.^356^	High risk of myopathy/rhabdomyolysis. Contraindicated: itraconazole + simvastatin/lovastatin (up to 20-fold increase in exposure).^357^ Safe alternatives: pravastatin (not metabolized by CYP3A4),^358^fluvastatin, or rosuvastatin (use with monitoring).
**3. ARVs**	Modulation of CYP3A4:^359^ PIs: inhibit statin metabolism. NNRTI (efavirenz): induces statin metabolism.	Preferred statins (safer): pravastatin and pitavastatin. With PIs: – Avoid: simvastatin, lovastatin. – Not recommended: atorvastatin with strong PIs (eg, ritonavir). – Caution: rosuvastatin (greatly increased exposure with lopinavir/ritonavir). With efavirenz (NNRTI): Decreases atorvastatin, simvastatin, and pravastatin concentrations. Monitor effectiveness and adjust dose if needed.^360^
**4. CCBs**	Inhibition of simvastatin and lovastatin metabolism (CYP3A4) by verapamil and diltiazem. Amlodipine also interacts.^361 , 362^	Increased risk of myopathy. Simvastatin: maximum dose 20 mg/day with verapamil or amlodipine. Lovastatin: maximum dose 40 mg/day with verapamil.
**5. Antiarrhythmic agents (amiodarone)**	Inhibitor of *CYP3A4* and P-gp, primarily affecting simvastatin and lovastatin.^363^	Increased risk of myopathy. Simvastatin: maximum dose 20 mg/day. Lovastatin: maximum dose 40 mg/day. No adjustment needed: atorvastatin, rosuvastatin, pravastatin, fluvastatin, pitavastatin.
**6. Immunosuppressants**	Inhibition of *CYP3A4* and OATP1B1 transporter by cyclosporine, tacrolimus, everolimus, sirolimus.^364 , 365^	Significantly increased risk of myopathy/rhabdomyolysis. Avoid: lovastatin, simvastatin, pitavastatin. Use with limited dose (only if essential): – Fluvastatin ≤ 40 mg/day – Pravastatin ≤ 20 mg/day – Rosuvastatin ≤ 5 mg/day – Atorvastatin > 10 mg/day requires strict monitoring (CK, symptoms).
**7 Macrolides**	Potent inhibition of *CYP3A4* by clarithromycin and erythromycin, affecting simvastatin, lovastatin, and atorvastatin.^366^	Increased risk of myotoxicity. Avoid: simvastatin, lovastatin, atorvastatin with erythromycin and clarithromycin. Safe alternative: azithromycin has no significant interaction and can be used with any statin. Unaffected statins: rosuvastatin, fluvastatin, pravastatin.
**8. Fibrates**	Gemfibrozil: inhibits statin glucuronidation, increasing serum levels.^303^ Fenofibrate: safer interaction profile.	Increased risk of myopathy. Genfibrozil: – Avoid combination with lovastatin, pravastatin, simvastatin. – Use cautiously with atorvastatin, pitavastatin, rosuvastatin. – Safe: fluvastatin. Fenofibrate: combination with any statin is considered reasonable when clinically indicated.

CK: creatine kinase; DOACs: direct oral anticoagulants; INR: international normalized ratio; NNRTI: non-nucleoside reverse transcriptase inhibitor; PIs: protease inhibitors; P-gp: P-glycoprotein.

### 8.7. Management of Statin-Intolerant Patients

It is advisable to monitor patients for the development of SAMS, particularly during the first few months of statin therapy and after dose increases. CK levels should be measured if symptoms occur. Patients with known risk factors for SAMS require closer monitoring due to their increased risk of developing rhabdomyolysis.

Statin therapy should be discontinued immediately if CK levels exceed 10x ULN or if myopathy is suspected or diagnosed. In cases of moderate CK elevation (3× to 10× ULN), weekly CK monitoring is recommended. A baseline CK measurement should be obtained before initiating statin therapy, especially in individuals at high risk for muscle-related adverse events, such as those with a history of statin intolerance, a family history of myopathy, or concurrent use of drugs that increase the risk of myopathy. Routine CK testing is not recommended in asymptomatic individuals without medication changes or comorbidities.

Baseline liver enzyme testing (ALT and AST) should be performed before starting statin therapy. During treatment, liver function should be assessed if symptoms or signs suggest hepatotoxicity (eg, fatigue, weakness, loss of appetite, abdominal pain, dark urine, or jaundice). If liver enzyme abnormalities are present, potential contributing factors should be excluded and a possible link to statin use investigated. If ALT/AST levels exceed 3× ULN along with an increase in total bilirubin, statin dose reduction or discontinuation should be considered. If ALT/AST elevations are < 3× ULN, observation or switching to a statin with a different metabolic pathway (eg, pravastatin) is recommended.

A small proportion of patients may experience increases in liver enzymes — particularly ALT and AST — with little or no impact on gamma-glutamyl transferase, alkaline phosphatase, or bilirubin. Statin-induced transaminase elevations typically occur within the first 6 months of treatment, are asymptomatic, and usually resolve with dose reduction or discontinuation. Isolated transaminase elevations, in the absence of elevated bilirubin, have not been clinically or histologically linked to acute or chronic liver injury.^344^

#### 8.7.1. Discontinuation and Reintroduction of Statin Therapy

Discontinuation of statin therapy in patients at CV risk is associated with an increased incidence of major adverse CV events and mortality.^345^Therefore, every effort should be made to encourage patients to remain on treatment. The first step in maintaining therapy is to discontinue the statin (if still in use) and implement a 2-week washout period to assess whether symptoms resolve.

Alongside statin withdrawal, it is advisable to initiate non-statin lipid-lowering therapy — such as ezetimibe, BPA, and/or PCSK9 inhibitors — to mitigate the risks associated with increased levels of LDL-c if statins are discontinued without therapeutic replacement.^346 , 347^Patients should be clearly informed that ezetimibe, BPA, and PCSK9 inhibitors are not part of the statin class, in order to reduce misconceptions that may compromise treatment adherence.^348^

After this period, reintroduction of the same statin at a lower dose or substitution with a different statin may be attempted.^345^Switching to a statin metabolized through an alternative pathway may benefit some patients (eg, replacing the lipophilic, hepatically metabolized atorvastatin with a hydrophilic statin such as rosuvastatin or pravastatin, which are partially renally excreted, or pitavastatin, which is also lipophilic but metabolized via alternative hepatic pathways).

Although not supported by clinical trials, for patients who are highly reluctant to resume daily statin use or who have experienced symptoms with more than two daily statin regimens, intermittent dosing with a long half-life statin (eg, rosuvastatin, atorvastatin, or pitavastatin) may be attempted.

Following statin reinitiation, adjunctive lipid-modifying agents may be used to help achieve target levels of LDL-c. In cases of absolute intolerance to any statin dose, combination or standalone non-statin therapies may be employed ( Figures 8.1 , 8.2 e 8.3 ).

**Figure 8.3:** Simplified flowchart. ANA: antinuclear antibodies; CK: creatine kinase; ESR: erythrocyte sedimentation rate; PCSK9: proprotein convertase subtilisin/kexin type 9; T4: thyroxine; TSH: thyroid-stimulating hormone; ULN: upper limit of normal.

#### 8.7.2. Use of Products Without Proven Benefit

Vitamin D deficiency has been associated with the occurrence of statin-associated muscle symptoms; however, supplementation has not been shown to improve muscle-related outcomes.^349^

Coenzyme Q10 (CoQ10) is an essential compound for mitochondrial energy production and functions as an antioxidant. Statin therapy may reduce CoQ10 levels in the body, which has been proposed as a contributing factor to fatigue and muscle pain. This is due to statins inhibiting the HMG-CoA reductase enzyme, which is not only involved in cholesterol synthesis but also in the biosynthesis of CoQ10.^350 , 351^While this biological rationale supports a possible benefit in mitigating SAMS, meta-analyses and larger trials^350 - 353^have not demonstrated any clear efficacy. Before, there is insufficient evidence to recommend CoQ10 supplementation in patients undergoing statin therapy for SAMS.

#### 8.7.3. Drug interactions of statins

##### 8.7.3.1. Anticoagulants

Drug interactions between statins and warfarin have been reported, primarily mediated by CYP2C9 pathway inhibition, especially with fluvastatin and rosuvastatin, or by displacement from protein-binding sites, as seen with lovastatin. Most statins, except for pravastatin, have potential for this interaction.^354^In contrast, studies have shown no clinically significant interactions between warfarin and atorvastatin or pitavastatin. Similarly, no relevant interactions have been demonstrated with direct oral anticoagulants (DOACs) such as dabigatran, apixaban, rivaroxaban, and edoxaban.^355^For patients on warfarin, intensified monitoring of the international normalized ratio (INR) is mandatory when initiating or adjusting a statin.

##### 8.7.3.2. Azole Antifungals

Azole antifungals act as CYP3A4 isoenzyme inhibitors, which blocks the metabolism of statins processed through this pathway. This interaction increases statin plasma concentrations, raising the risk of myopathy and rhabdomyolysis.^356^Coadministration of itraconazole with simvastatin or lovastatin is contraindicated due to a marked increase in statin exposure (up to 20-fold increase in AUC), critically elevating musculoskeletal toxicity risk.^357^

In contrast, pravastatin, which is not metabolized via CYP3A4, does not have significant interaction with itraconazole.^358^When rosuvastatin is combined with fluconazole, an increase in AUC and Cmax is observed, but with no demonstrated clinical relevance. Therefore, for patients on azole antifungals, pravastatin — and to a lesser extent, fluvastatin or rosuvastatin (with monitoring) — represent safer therapeutic alternatives.

##### 8.7.3.3. Antiretroviral Agents

Managing dyslipidemia in HIV-positive patients is increasingly common due to improved survival and increased CV risk. Antiretroviral therapy (ART) poses clinically significant interactions with statins, mainly via CYP3A4 modulation by protease inhibitors (PIs) and non-nucleoside reverse transcriptase inhibitors (NNRTIs).^359^

Pravastatin, due to its sulfation metabolism, and pitavastatin are the statins of choice and safest options in this context. However, pravastatin may require dose adjustment (increase) when associated with PIs such as nelfinavir or ritonavir. Conversely, simvastatin and lovastatin should be avoided with PIs. Atorvastatin is also discouraged with potent PIs (eg, ritonavir, atazanavir). Rosuvastatin requires caution, as its combination with lopinavir/ritonavir or atazanavir/ritonavir has been shown to substantially increase systemic exposure (AUC and Cmax). In contrast, efavirenz (an NNRTI) acts as an enzymatic inducer, lowering the concentrations of atorvastatin, simvastatin, and pravastatin, which requires clinical monitoring and potential dose adjustments to maintain lipid-lowering efficacy.^360^

##### 8.7.2.4. Calcium Channel Blockers

Diltiazem may increase simvastatin's Cmax by 3.6-fold and AUC by 5-fold, and lovastatin's by 3.5-fold, raising the risk of myopathy.^361^Dose adjustments are recommended for patients treated with verapamil and simvastatin (maximum 20 mg/day) or lovastatin (maximum 40 mg/day).

The dihydropyridine calcium channel blocker amlodipine also increases simvastatin's Cmax and AUC.^362^The U.S. Food and Drug Administration (FDA) recommends that simvastatin doses should not exceed 20 mg/day when coadministered with any dose of amlodipine, due to the elevated risk of myopathy and/or rhabdomyolysis at higher doses.

##### 8.7.3.5. Antiarrhythmic Agents

Amiodarone is an irreversible CYP3A4 inhibitor and may interact with statins metabolized by CYP450 or that are P-gp substrates. Reports have shown increased toxicity when amiodarone is combined with CYP3A4-substrate statins, particularly simvastatin. A 75% increase in both AUC and Cmax of simvastatin has been observed when coadministered with amiodarone.^363^

No dose adjustments are required for atorvastatin, rosuvastatin, pravastatin, fluvastatin, or pitavastatin when used with amiodarone. However, lovastatin should not exceed 40 mg/day, and simvastatin should be limited to no more than 20 mg/day when prescribed with amiodarone.

##### 8.7.3.6. Immunosuppressants

Coadministration of statins with calcineurin inhibitors (eg, cyclosporine, tacrolimus) and mTOR inhibitors (eg, everolimus, sirolimus) significantly increases the risk of myopathy and rhabdomyolysis. This interaction occurs through inhibition of CYP3A4 and the OATP1B1 transporter, raising serum statin levels. Clinically, it is recommended to avoid combinations with lovastatin, simvastatin, and pitavastatin. When therapy is essential, fluvastatin (up to 40 mg/day), pravastatin (≤ 20 mg/day), or rosuvastatin (≤ 5 mg/day) may be used. Atorvastatin doses > 10 mg/day require strict monitoring of CK levels and symptoms of muscle toxicity.^364 , 365^

##### 8.7.3.7. Macrolides

Macrolides — especially clarithromycin and erythromycin — are potent CYP3A4 inhibitors, followed by the weaker inhibitor roxithromycin and, lastly, azithromycin. CYP3A4 is the isoenzyme that metabolizes simvastatin, lovastatin, and atorvastatin, and its inhibition increases plasma statin concentrations and the risk of myotoxicity. Rosuvastatin, fluvastatin, and pravastatin are not significantly affected by this interaction. Among macrolides, azithromycin is considered safe for use with all statins. Erythromycin significantly increases simvastatin and atorvastatin levels.^366^Therefore, simvastatin, lovastatin, and atorvastatin should be avoided in combination with erythromycin and clarithromycin due to the increased risk of myopathy.

##### 8.7.3.8 Interactions Between Lipid-Lowering Agents

Although combining statins with fibrates can be effective for lipid lowering, it also increases the risk of myopathy. This interaction is particularly critical with gemfibrozil, which inhibits statin glucuronidation, increasing their serum concentrations. Gemfibrozil should not be combined with lovastatin, pravastatin, or simvastatin.^304^Its combination with atorvastatin, pitavastatin, and rosuvastatin may be considered if clinically indicated, despite a modest increase in statin levels. Fluvastatin does not exhibit pharmacokinetic interaction with gemfibrozil and can be used without dose restrictions. In contrast, fenofibrate has a safer profile and its use with any statin is considered acceptable when clinically indicated.

**Table:** 

Recommendation	Strength of Recommendation	Certainty of Evidence
In patients for whom statin therapy is being considered, baseline measurement of CK and liver enzymes (ALT and AST) is recommended, especially in individuals at high risk for muscle or hepatotoxic events.	CONDICIONAL	LOW
In patients on statins, routine measurement of CK and liver enzymes is recommended in the absence of muscle symptoms, signs of hepatotoxicity, or therapy-related abnormalities.	STRONG	MODERATE
In patients on statins, measurement of CK is recommended in the presence of severe muscle symptoms, and liver enzyme testing is recommended when signs of hepatotoxicity are present.	STRONG	MODERATE
In patients who do not tolerate the suggested statin dose, alternative strategies are recommended to achieve LDL-c reduction goals, including lowering the administration frequency, switching to another statin, or combining with other lipid-lowering agents.	STRONG	HIGH
In patients for whom statin therapy is discontinued, immediate initiation of non-statin lipid-lowering therapy (eg, ezetimibe, BPA, or PCSK9 inhibitors) is recommended, either as a bridge or permanently, with the goal of mitigating CV risk due to elevated LDL-c.	STRONG	HIGH
In patients on statins, vitamin D supplementation is recommended to help mitigate muscle symptoms associated with statin use.	STRONG	HIGH
In patients on statins, routine supplementation with coenzyme Q10 is recommended to help mitigate muscle symptoms associated with statin use.	STRONG	MODERATE

ALT: alanine transaminase; AST: aspartate aminotransferase; BPA: bempedoic acid; CK: creatine kinase; CV: cardiovascular; LDL-c: low-density lipoprotein cholesterol; PCSK9: proprotein convertase subtilisin/kexin type 9.

## 9. Dyslipidemia in Specific Populations: Clinical Management Considerations

### 9.1 Heart Failure

Dyslipidemia is a common comorbidity in patients with HF, particularly when ASCVD is present. Adequate lipid control is essential, as it can significantly influence clinical outcomes in this population. Statins are recommended for individuals with ischemic HF due to their proven effectiveness in reducing CV events. However, in patients with HF with reduced ejection fraction (HFrEF) without established ASCVD, the mortality benefit of statin therapy remains uncertain.

Importantly, tatin discontinuation in patients with HF — even in those with advanced or decompensated disease — is not recommended. There is no robust evidence to support such a practice, and observational studies suggest increased mortality and hospitalization rates following statin withdrawal, particularly in older patients.^367 - 370^

The prevalence of HF, dyslipidemia, and CAD is especially high among older adults. In this population, polypharmacy is a frequent concern and may lead to unintentional discontinuation of statins or other lipid-lowering agents. However, such interruptions may increase the risk of CV events and death, as demonstrated in observational studies.^369^

For patients who do not reach LDL-c targets with statins — particularly those at high CV risk — additional therapies such as ezetimibe or PCSK9 inhibitors should be considered.

In conclusion, the management of dyslipidemia in HF should be individualized, with an emphasis on maintaining lipid-lowering therapy, especially in patients with ASCVD. Statin discontinuation is not recommended due to the increased risk of adverse events. In older patients, careful assessment of polypharmacy is essential to avoid inappropriate treatment interruption. Further studies are needed to clarify the role of emerging lipid-lowering therapies in this clinical setting.^371^

**Table:** 

Recommendation	Strength of Recommendation	Certainty of Evidence
Patients with HF and established ASCVD should continue statin therapy to reduce the risk of ASCVD events.	STRONG	MODERATE
In patients with HF who were already on statin therapy, discontinuation is not recommended if the patient has a clinically acceptable life expectancy.	STRONG	MODERATE
Patients with HFrEF and no ASCVD may use statins when no contraindications are present, provided treatment is individualized.	CONDICIONAL	MODERATE
PCSK9 inhibitors should be maintained in patients with HF at high risk when LDL-c targets are not achieved with statins and ezetimibe.	STRONG	MODERATE

ASCVD: atherosclerotic cardiovascular disease; HF: heart failure; HFrEF: HF with reduced ejection fraction; LDL-c: low-density lipoprotein cholesterol; PCSK9: proprotein convertase subtilisin/kexin type 9.

### 9.2 People Living with HIV

People living with HIV (PLWH) have nearly twice the risk of CVD compared to the general population, due to factors such as chronic inflammation, adverse effects of ART, and a high prevalence of traditional risk factors.^124^Despite the availability of effective LDL-c-lowering therapies, achieving lipid targets in this population remains a significant challenge. Statins continue to be the cornerstone of LDL-c reduction in PLWH; however, their use is often hindered by drug interactions with ART, leading to increased adverse effects and poor adherence.^372^( Table 9.1 ). The REPRIEVE trial demonstrated a 35% reduction in CV events with pitavastatin use, underscoring its role in primary prevention.^372^Nonetheless, challenges such as statin intolerance and underutilization of therapy persist, with studies showing that only 37% of PLWH at high risk receive appropriate lipid-lowering treatment.^124^

**Table 9.1:** Safety Profile and Statin Interactions in PLWH

Statin	Recommended Dose	Interaction with ART	Key Comments
**Pitavastatin**	4 mg/day	LOW	Safest statin for use with ART; highlighted in the REPRIEVE trial showing a 35% reduction in CV events.^372^
**Rosuvastatin**	10-20 mg/day	MODERATE	Potent; use with caution alongside protease inhibitors.
**Atorvastatin**	10-20 mg/day	MODERATE	Dose adjustment needed with ART; avoid high doses with ritonavir.
**Pravastatin**	20-40 mg/day	LOW	Lower potency but safe with most antiretrovirals.
**Simvastatin**	Avoid	HIGH	Contraindicated with protease inhibitors (eg, ritonavir and cobicistat)

ART: antiretroviral therapy; CV: cardiovascular; LDL-c: low-density lipoprotein cholesterol; PCSK9: proprotein convertase subtilisin/kexin type 9; PLWH: people living with HIV.

Ezetimibe has also been underutilized, with only 3.3% of patients receiving this therapy despite its favorable safety profile.^373^Moreover, traditional CV risk calculators often underestimate risk in PLWH, highlighting the need for individualized approaches that incorporate HIV-specific factors such as immune activation and ART exposure.^374^Evolocumab, a PCSK9 inhibitor, has emerged as a promising alternative for PLWH with statin intolerance or residual CV risk. The BEIJERINCK trial demonstrated a 56.9% decrease in the levels of LDL-c, with 72.5% of patients achieving a ≥ 50% decrease.^373^Additionally, evolocumab was shown to reduce the levels of Lp(a), which may be particularly relevant in PLWH because of the inflammatory milieu associated with chronic viral infection.^373^

Achieving LDL-c goals in PLWH remains a relevant clinical challenge, marked by barriers such as drug interactions, poor treatment adherence, and underuse of available therapeutic options. Despite the well-established role of statins as the foundation of lipid-lowering therapy, incorporating new strategies — especially PCSK9 inhibitors — represents a promising approach to improving CV outcomes in this population at high risk. Therapeutic advancement, coupled with personalized care, may contribute substantially to overcoming these limitations and reducing residual risk.^375 , 376^

**Table:** 

Recommendation	Strength of Recommendation	Certainty of Evidence
In PLWH, statins should be considered as first-line therapy for LDL-c reduction and cardiovascular risk management, according to the relevant target. The choice of statin should take into account the risk of drug–drug interactions.	STRONG	HIGH
In PLWH with statin intolerance or insufficient LDL-c reduction, ezetimibe should be added	STRONG	MODERATE
In PLWH with high cardiovascular risk and inadequate LDL-c control despite maximally tolerated therapy, PCSK9 inhibitors such as evolocumab should be considered.	STRONG	MODERATE

LDL-c: low-density lipoprotein cholesterol; PCSK9: proprotein convertase subtilisin/kexin type 9; PLWH: people living with HIV.

### 9.3. Diabetes

Diabetes is a metabolic disorder that frequently predisposes individuals to CVD, making it one of the leading causes of morbidity and mortality in patients with T1DM and T2DM. Recent data indicate that diabetes per se increases the risk of CVD by approximately twofold on average; however, this risk varies considerably depending on the population and the preventive strategies adopted.^377 , 378^Individuals with both diabetes and CAD have a significantly higher risk of future CV events. In the case of T2DM, the risk of ASCVD is largely influenced by the presence of target organ damage, such as nephropathy (microalbuminuria), neuropathy, or retinopathy — with the risk increasing as more conditions are present.^379^

Good glycemic control in T1DM leads to fewer CV events. However, in T2DM, the presence of CVD is independent of intensive glycemic control.^380^In addition to insulin resistance and deficiency, dyslipidemia is a common comorbidity in patients with diabetes and can significantly affect clinical outcomes. Increased TG or low fasting and postprandial HDL-c levels are observed in approximately half of individuals with T2DM^381^and are also frequently found in those with abdominal adiposity, insulin resistance, or impaired glucose tolerance.^382^

#### 9.3.1. Specific Characteristics of Dyslipidemia in Insulin Resistance and Type 2 Diabetes

Diabetic dyslipidemia refers to a cluster of lipid and lipoprotein abnormalities that are metabolically interrelated. In T2DM, increased large VLDL particles trigger a cascade of events resulting in atherogenic remnants, small dense LDL particles, and TG-rich HDL particles.^383^Alterations in the composition of LDL and HDL particles affect their function. ApoC-III levels are increased, which impairs the clearance of TG-rich lipoproteins and their remnants, prolonging their circulation time^382^and making them more atherogenic. This defective catabolism of TG-rich lipoproteins appears to be a more significant factor in increased plasma TG than increased production rates, resulting in an excess of remnant particles. This overall profile is also characterized by an increase in ApoB-containing particles. TG-rich lipoproteins — including chylomicrons, VLDL, and their remnants — carry a single ApoB molecule, just like LDL particles. As a result, the harmful nature of diabetic dyslipidemia is not always captured by standard lipid measures, since LDL-c levels may remain within normal limits. Thus, non-HDL-c or ApoB are better markers of TG-rich lipoproteins and remnants.^43^

#### 9.3.2. Treatment of Dyslipidemia in Patients with Diabetes

Lifestyle Modifications

- **Diet:** A balanced diet rich in unsaturated fatty acids (eg, those found in olive oil, nuts, and avocados) and low in saturated and trans fats can help improve lipid levels. Adequate fiber intake is also important to reduce the levels of LDL-c and TG.
- **Physical activity:** Regular exercise — particularly aerobic and resistance training — can increase HDL and reduce TG. Exercise also improves insulin sensitivity.
- Weight loss: Reducing weight, especially visceral fat, can improve the lipid profile and insulin resistance.

#### 9.3.3. Pharmacological Treatment

- **Statins:** LDL-c is the primary target of lipid-lowering therapy in patients with diabetes. Studies conducted specifically in individuals with T2DM, as well as subgroups of people with diabetes included in large statin trials, have consistently demonstrated significant CV benefits of statin therapy.^384^According to the CTT meta-analysis, statin treatment reduces the incidence of major CV events by approximately 23% over 5 years for every 38.4 mg/dL reduction in LDL-c, regardless of baseline LDL-c or other characteristics.
- **Ezetimibe:** Ezetimibe may be used in combination with statins to further lower LDL-c levels, especially in patients at high CV risk. It reduces LDL-c by 24%, and when added to statin therapy, decreases the risk of major vascular events.^385^The RR reduction is proportional to the absolute LDL-c reduction and consistent with the effect seen with statins. In the IMPROVE-IT trial, the diabetic subgroup had a higher rate of major CV events than those without diabetes (46% vs. 31%). Ezetimibe appeared particularly effective in patients with diabetes, with a 15% RR reduction (95% CI, 6%-22%) and a 5.5% absolute risk reduction.^284^
- **PCSK9 inhibitors:** In patients with diabetes and severe dyslipidemia and high CV risk, PCSK9 inhibitors (eg, alirocumab and evolocumab) are effective treatment options for lowering LDL-c. These drugs reduce LDL-c levels by approximately 60% and decrease the risk of major CV events.^168^In the FOURIER trial, the RR reduction for CV events was similar in patients with and without diabetes; however, due to a higher baseline risk in those with diabetes, the absolute risk reduction was greater (2.7% reduction in major CV events over 3 years).^196^Similar benefits were observed in patients with diabetes following ACS in the ODYSSEY trial.^197^More recently, inclisiran has shown a substantial and sustained reduction in LDL-c across all glycemic and BMI strata.^386^
- **Fibrates:** Fibrates — agonists of PPAR-α — are potent lipid-modifying agents. Their main effects include lowering TG and increasing HDL-c levels. Several controlled trials have failed to show CV benefit, particularly when used as an "add-on" to statin therapy. However, subsequent analyses of large clinical trials, meta-analyses, and real-world data have proposed a potential role for fibrates in specific subgroups of patients with atherogenic dyslipidemia and MetS.^387^Recently, in patients with T2DM, mild to moderate HTG, and low HDL and LDL-c levels, pemafibrate did not reduce CV event incidence compared to placebo, although it significantly lowered TG, VLDL cholesterol, remnant cholesterol, and ApoC-III.^303^Despite this neutral outcome, there are indicators of potential benefit in microvascular ischemic complications such as PAD. Subsequent analyses showed a reduction in ischemic ulceration and gangrene of the lower limbs.^388^Emerging data from the PROMINENT trial and experimental studies also suggest that pemafibrate may offer benefits in NAFLD and diabetes-related microangiopathy — topics that warrant further investigation.^389^The LENS study evaluated the role of fenofibrate in patients with mild diabetic retinopathy. A total of 1,151 participants randomized to fenofibrate versus placebo were included. During a median of 4 years, retinopathy progression occurred in 131 (22.7%) of 576 participants in the fenofibrate group versus 168 (29.2%) of 575 in the placebo group (HR: 0.73, 95% CI 0.58-0.091, p=0.006).^390^
- **Omega-3 fatty acids:** Omega-3 supplements may be used to lower elevated TG and improve the lipid profile in diabetic patients. Omega-3 fatty acids — particularly EPA — have been studied for their ability to reduce TG levels. The REDUCE-IT trial showed that IPE, a purified form of EPA, reduced CV events in patients with T2DM and established CV disease with TG levels between 135 and 499 mg/dL.^305^While other EPA studies have shown inconsistent results, REDUCE-IT is the most relevant for this population. A 4 g/day dose of IPE significantly reduced CV events in patients with diabetes and is considered effective, though platelet function monitoring is advised due to its antiplatelet effects.

The current guideline includes a specific risk and treatment flowchart for patients with diabetes, recognizing this group's high CV risk and distinct clinical characteristics. Lipid-lowering therapy in this population has a greater impact, given the elevated absolute risk of CV events. Thus, we recommend referring to the chapters on risk stratification, therapeutic targets, and treatment strategies. Primary targets should focus on LDL-c and, as a co-primary, non-HDL-c, along with the importance of measuring ApoB, especially in patients with diabetes due to the increased presence of atherogenic ApoB-containing particles.

**Table:** 

Recommendation	Strength of Recommendation	Certainty of Evidence
For individuals with diabetes, statins are the first-line lipid-lowering therapy for patients with LDL-c levels above the established target	STRONG	HIGH
For individuals with diabetes, ezetimibe may be used in those who remain above the LDL-c target despite maximally tolerated statin therapy	STRONG	MODERATE
For individuals with diabetes, PCSK9 inhibitors may be used in those who remain above the LDL-c target despite maximally tolerated statin and ezetimibe therapy	STRONG	MODERATE
For individuals with diabetes and mild retinopathy, fenofibrate may be used to reduce the progression of diabetic retinopathy	STRONG	MODERATE

LDL-c: low-density lipoprotein cholesterol; PCSK9: proprotein convertase subtilisin/kexin type 9.

### 9.4. Hypothyroidism

Hypothyroidism is a common endocrine disorder affecting approximately 4% to 10% of the population in its subclinical form and about 0.3%-0.4% in its clinical form. It is more prevalent in women, with a ratio of up to 4:1 compared to men.^391 , 392^It is classified as clinical (increased TSH with low free T4) or subclinical (increased TSH with normal free T4). Both forms significantly affect lipid metabolism and CV risk.

Thyroid hormones — especially T3 — regulate hepatic expression of LDL receptors (LDL-R) and LPL activity. In hypothyroidism, reduced LDL-R expression leads to impaired LDL-c clearance and increased plasma levels of LDL-c.^393^Decreased LPL activity contributes to impaired TG clearance and resulting HTG. Additionally, increased levels of Lp(a) and accumulation of small dense LDL particles — more atherogenic in nature — are observed.^394 , 395^

These changes result in a pro-atherogenic lipid profile, even in patients with subclinical hypothyroidism. The pro-inflammatory state associated with hypothyroidism, along with endothelial dysfunction, also fosters an environment conducive to CVD development.

Studies have shown that persistently elevated TSH is associated with increased CIMT, arterial stiffness, and a higher incidence of CV events, such as MI and stroke.^396^Dyslipidemia caused by hypothyroidism is also often less responsive to statins until thyroid function is restored.^397^

Levothyroxine therapy is indicated in all cases of clinical hypothyroidism. In subclinical hypothyroidism, hormone replacement should be considered when TSH ≥10 mIU/L or in patients with typical symptoms, established CVD, diabetes, or multiple risk factors.^398^Treatment is also recommended for patients with subclinical hypothyroidism and TSH between 4.5 and 9.9 mIU/L when dyslipidemia is present.^399^Laboratory parameters should be monitored periodically — typically after 6-8 weeks — to adjust the levothyroxine dosage and assess treatment response. Normalization of thyroid function generally improves the lipid profile, particularly by reducing LDL and TC levels.^399^

Importantly, until thyroid hormone levels are normalized, patients may be more vulnerable to statin-related side effects such as myalgia or, in rare cases, rhabdomyolysis. Hormonal replacement and normalization of TSH and free T4 levels generally reduce these risks, making statin use safer.

The decision to initiate statin therapy should be individualized, considering overall CV risk, thyroid treatment response, and close monitoring of adverse effects.

On the other hand, hyperthyroidism is generally associated with a hypolipidemic profile, characterized by reduced total cholesterol, LDL-c, and TG due to increased LDL-R expression and accelerated hepatic lipoprotein metabolism.^393^However, HDL-c levels may also decrease, attributed to increased CETP activity.

**Table:** 

Recommendation	Strength of Recommendation	Certainty of Evidence
In patients with clinical hypothyroidism and dyslipidemia, hormone replacement therapy with levothyroxine is recommended to normalize TSH and improve the lipid profile.	STRONG	HIGH
In patients with clinical hypothyroidism and dyslipidemia, the use of statins is recommended in those whose dyslipidemia persists after normalization of thyroid function, particularly in the presence of high CV risk.	STRONG	MODERATE
In patients with subclinical hypothyroidism and dyslipidemia, levothyroxine therapy is recommended when TSH is between 4.5 and 9.9 mIU/L and the patient presents with hypothyroidism symptoms or high CV risk.	STRONG	MODERATE
In patients with subclinical hypothyroidism and dyslipidemia, levothyroxine therapy is recommended when TSH exceeds 10 mIU/L.	STRONG	HIGH

CV: cardiovascular; TSH: thyroid-stimulating hormone.

### 9.5. Chronic Kidney Disease

CKD is recognized as an important independent CV risk factor. The association between CKD and CVD is one of the leading causes of morbidity and mortality among dialysis and kidney transplant patients.^400^Dyslipidemias in patients with CKD present distinct characteristics compared to those in the general population. These lipid metabolism alterations are primarily characterized by reduced LPL activity, HTG, accumulation of remnant lipoproteins (VLDL and IDL), elevated LDL, and changes in HDL composition and function. These abnormalities, combined with chronic inflammation and oxidative stress, significantly increase atherogenic risk — even in individuals with normal total cholesterol or LDL-c levels — which complicates risk stratification using traditional parameters.^401 , 402^

Therapeutic management is therefore critical to reduce CV risk, slow CKD progression, and address chronic inflammation. It also indirectly contributes to the control of associated conditions such as diabetes and MetS.

The primary therapeutic strategy involves the use of statins, with or without ezetimibe, aiming to reduce absolute CV risk regardless of specific LDL-c targets, especially in patients with reduced GFR.^55 , 403 - 405^The combination of statin and ezetimibe has shown additional LDL-c reduction in pre-dialysis patients.^406 , 407^In dialysis patients, particularly those undergoing peritoneal dialysis, statin therapy may be initiated in individuals at very high CV risk, as lipid metabolism is relatively preserved, potentially increasing the benefit of pharmacologic treatment.^55 , 400 , 401^For those on hemodialysis, the benefits of initiating lipid-lowering therapy remain controversial, and there is concern regarding potential drug interactions and adverse effects. However, for patients already receiving lipid-lowering therapy prior to starting dialysis, continuation is recommended.^400 , 406 - 409^In kidney transplant recipients, CV risk is elevated due to both traditional risk factors and adverse effects of immunosuppressive therapy. Statin use is associated with reduced CV events and mortality.^401^Simvastatin and atorvastatin are metabolized via hepatic CYP3A4, which can lead to relevant interactions with immunosuppressants such as cyclosporine. Therefore, statins with lower interaction risk — such as pravastatin, fluvastatin, or rosuvastatin — are preferred.^401^Lipid-lowering therapies other than statins lack robust data in the CKD population. Other interventions — including a balanced diet low in saturated fat, smoking cessation, regular physical activity, and weight control — have proven benefits for lipid profile, endothelial function, and comorbidity management, and should be part of comprehensive patient counseling.^55 , 401 , 403 , 404^

**Table:** 

Recommendation	Strength of Recommendation	Certainty of Evidence
In individuals with CKD stages 1-3 and increased CV risk, high-intensity statins are recommended to reduce CV risk.	STRONG	HIGH
In individuals with CKD stages 1-3 and increased CV risk who have not achieved targets, the addition of ezetimibe to high-intensity statins is recommended.	STRONG	MODERATE
In individuals with CKD stages 4-5 not on dialysis, initiation of high-intensity statins, with or without ezetimibe, is recommended.	STRONG	HIGH
In individuals with CKD on dialysis and no established CVD, statin initiation is not recommended.	STRONG	HIGH

CV: cardiovascular; CKD: chronic kidney disease.

### 9.6. Obesity

Dyslipidemia and obesity are highly prevalent clinical conditions worldwide and are associated with significant morbidity and an increased risk of major CV events. The coexistence of these conditions amplifies metabolic disturbances, fostering a pro-atherogenic, inflammatory environment conducive to macrovascular ASCVD.

Combining obesity and dyslipidemia substantially increases the risk of CAD, stroke, HF, and PAD.^410 , 411^The typical lipid pattern in individuals with obesity — HTG, low HDL-c, and small dense LDL particles — represents the lipid phenotype most strongly associated with MetS and accelerated atherogenesis.^412^Excess adipose tissue, particularly visceral fat, promotes insulin resistance via inflammatory adipokines such as TNF- and IL-6, and reduced levels of adiponectin.^413^This resistance leads to increased lipolysis and circulating free fatty acids, which are transported to the liver, resulting in increased VLDL production and HTG.^413^Studies show that a body weight loss of 5%-10% already leads to significant improvements in lipid and glycemic profiles.^414^

Nonpharmacological interventions are essential and constitute first-line therapy. Recommendations include a calorie-restricted, balanced diet low in saturated fat (< 7% of total calories), increased intake of soluble fiber and phytosterols, and at least 150 minutes of moderate-intensity aerobic exercise per week.^415^

The need for pharmacological lipid-lowering therapy depends on the lipid profile and calculated CV risk. Statins have well-established benefits in reducing CV morbidity and mortality and should form the foundation of treatment when LDL-c is elevated, regardless of obesity status.^416^Ezetimibe may be added to statins when stricter LDL-c targets are required.^165^PCSK9 inhibitors are indicated for patients at very high CV risk or with FH and refractory LDL-c despite maximally tolerated treatment. Fibrates are primarily used in cases of HTG ≥ 500 mg/dL to reduce the risk of pancreatitis.^303^

GLP-1 receptor agonists — such as liraglutide and semaglutide — not only promote significant weight loss but also improve the lipid profile, contributing to modest yet consistent reductions in TG and TC.^417^Recent evidence from the SELECT trial showed that in patients with established CVD and very high CV risk, semaglutide 2.4 mg significantly reduced major CV events, independently of weight loss, which reinforces its role as a cardioprotective agent beyond its weight-related effects.^219 , 418^

Bariatric surgery remains the most effective intervention for sustained weight loss and significantly improves metabolic parameters — including marked reductions in LDL-c and TG, increased HDL-c, and improved insulin resistance.^419^

**Table:** 

Recommendation	Strength of Recommendation	Certainty of Evidence
In individuals with dyslipidemia secondary to obesity, nonpharmacological interventions are recommended as first-line treatment.	STRONG	HIGH
In individuals with dyslipidemia secondary to obesity, statins are recommended as the foundation of pharmacological treatment.	STRONG	HIGH
In individuals with dyslipidemia secondary to obesity, fibrates are recommended when TG are ≥ 500 mg/dL to reduce the risk of pancreatitis.	STRONG	MODERATE
In individuals with dyslipidemia secondary to obesity, fibrates are NOT recommended for CV risk decrease or for pancreatitis prevention when TG are ≤ 500 mg/dL.	STRONG	HIGH
In individuals with dyslipidemia secondary to obesity, GLP-1 receptor agonists are recommended for their dual effect on weight loss and CV event decrease.	STRONG	HIGH
In individuals with dyslipidemia secondary to obesity, bariatric surgery is recommended to improve lipid profile and reduce CV events.	STRONG	MODERATE

CV: cardiovascular; GLP-1: glucagon-like peptide-1; TG: triglycerides.

### 9.7. Older Individuals

The risk of CVD increases with age,^420^as does the risk of nonCV mortality, inevitably reducing life expectancy. For older adults, the treatment of risk factors for CVD must be carefully evaluated to balance benefits and risks, as these factors may differ among individuals with limited life expectancies.^421 , 422^Importantly, older adults generally face a higher risk of adverse events and drug side effects.^423 , 424^Therefore, identifying those who may benefit from preventive treatment is crucial.

The decision to initiate lipid-lowering therapy in older adults must consider specific factors such as drug pharmacokinetics, lower levels of clinical evidence for certain age groups, and the high prevalence of SAD in individuals over 65 years of age.^425^

Levels of TC are generally higher until the sixth decade of life, decreasing slightly with age. Even in genetic dyslipidemias, significant elevations in TC, TG, and LDL-c are uncommon. Secondary dyslipidemias are more frequent and may be due to hypothyroidism, diabetes, glucose intolerance, obesity, nephrotic syndrome, or medications such as thiazide diuretics and nonselective beta-blockers. The association between high cholesterol levels and increased risk of CAD in middle-aged and early older adults weakens with advancing age^426^— likely a consequence of frailty orcompetitive events. In the Prospective Studies Collaboration, which included 61 prospective observational studies, the association between TC and vascular mortality was demonstrated in 900,000 individuals with no history of vascular disease, aged 40 to 89 years. The relative risk reduction of ischemic heart disease for each 39 mg/dL reduction in total cholesterol was smaller in older age groups, but as the absolute rate of this event increased with increasing age, there was a greater absolute difference in the oldest age group.

Although stronger evidence supports the role of dyslipidemias in the pathogenesis of atherosclerosis and CAD in middle-aged individuals based on observational and experimental studies,^427^later studies have provided relevant findings that may support the treatment of dyslipidemia in the older adults as well.^428^In older adults, treatment decisions must consider the patient's overall health and mental status, socioeconomic conditions, family support, comorbidities, and concurrent medications, which may interact with lipid-lowering therapies and influence adherence and treatment continuity.^429^

### 9.8. Nonpharmacological Treatment

Recommendations for nonpharmacological therapy should follow the same principles applied to younger adults considering the caloric, protein, and vitamin needs of older individuals. Additionally, the recommendation for regular physical activity should be included.

### 9.9. Pharmacological Treatment

Statins are the first-line pharmacological agents in this population. Evidence from studies involving older adults — such as the PROSPER (PROspective Study of Pravastatin in the Elderly at Risk) trial using pravastatin,^430^the Heart Protection Study with a large sample of individuals over 65 years old,^431^an exploratory analysis of the JUPITER (Justification for the Use of Statins in Prevention: an Intervention Trial Evaluating Rosuvastatin) trial in individuals aged 70 and older,^432^and the HOPE-3 trial, in which half the patients were over 65^433^— as well as a meta-analysis by the Cholesterol Treatment Trialists (CTT) collaboration,^166^all demonstrated a RR reduction in CV events in this subgroup receiving statin therapy.

The EWTOPIA 75 (Ezetimibe Lipid-Lowering Trial on Prevention of Atherosclerotic Cardiovascular Disease in 75 or Older) trial evaluated the efficacy of ezetimibe in preventing CV events in individuals over 75 years of age and showed a reduction in the primary and secondary outcomes and coronary revascularization, with no difference in all-cause mortality or stroke.^434^

The STAREE (Statins in Reducing Events in the Elderly) trial^435^is currently investigating the effects of statin therapy for primary prevention of CV events and mortality reduction in healthy individuals over 70 years of age. Results are expected to be available in 2025.

The US Preventive Services Task Force states that there is insufficient evidence to support initiating the treatment of dyslipidemia in adults over 75 years old without a prior history of CVD.^436^

The SCORE2-OP, recommended by the ESC to estimate CV risk in individuals over 70, may support treatment decision-making in this population.^159^

Therefore, the treatment of dyslipidemia in older adults should be tailored to the individual, accounting for factors such as comorbidities, life expectancy, and potential drug interactions. Treatment should be recommended for individuals at high or very high CV risk, with similar targets to those for younger individuals.

**Table:** 

Recommendation	Strength of Recommendation	Certainty of Evidence
After 75 years of age, it is recommended to individualize the doses of lipid-lowering agents according to frailty, presence of comorbidities, life expectancy, and the use of polypharmacy.	STRONG	MODERATE

### 9.10. Children

Atherosclerosis is a progressive process that begins in childhood and is enhanced by factors such as increased cholesterol, obesity, and MetS — conditions that increase CV risk in adulthood. Knowledge about dyslipidemias in children has advanced significantly, enabling earlier diagnosis and more effective therapeutic approaches. Notably, LDL-c targets in this age group differ from those used in the adult population, being more lenient and adjusted to the developmental stage. The primary goal remains early prevention, ensuring safety and effectiveness while avoiding both the progression of atherosclerosis and adverse effects on growth and metabolic maturation.

#### 9.10.1 Lipid Profile in Childhood

In children and adolescents, the assessment of lipids should be conducted after 8-9 hours of fasting and should include TC, HDL-c, TG, and LDL-c, which can be calculated or directly measured.^437^

#### 9.10.2. Screening

Universal screening: between ages 9-11 and 17-21, preferably while fasting, regardless of family history.^438^

Selective screening: between ages 2-8 and 12-16 in children with risk factors such as:^438^

- Family history of hypercholesterolemia or premature CVD
- Overweight/obesity
- Diabetes, hypertension, or tobacco use

#### 9.10.3. Primary Dyslipidemias

These are genetic, present from an early age, and can be monogenic or polygenic. The main forms are:

- HeFH;^87^
- A dominant genetic disorder characterized by increased LDL-c from birth;
- Affects 1 in every 250-300 individuals and is among the most common inherited causes of CVD;^439^
- Caused by mutations in genes such as the LDL receptor, ApoB, PCSK9.^439 , 440^

Diagnosis: LDL-c > 190 mg/dL or > 160 mg/dL with a positive family history. Genetic testing is useful but can be replaced by phenotypic evaluation. LDL-c should be measured at least twice over a 3-month period.^87^

Treatment: Includes diet, physical activity, and statin therapy, which should begin between ages 8-10 (or even earlier in severe cases). Statins reduce LDL-c by an average of 32% and are safe. Studies show that early initiation significantly reduces future CV events. The therapeutic goal is LDL-c < 130 mg/dL.^441 , 442^

Additional therapies:

- Ezetimibe can reduce LDL-c by up to 27% and may be combined with statins;^443^
- PCSK9 inhibitors (eg, evolocumab and alirocumab) are indicated in resistant cases or in statin intolerance;
- Evolocumab reduces LDL-c by approximately 44% in children and adolescents. Both are approved for pediatric use.^444^

#### 9.10.4. Homozygous Familial Hypercholesterolemia

- A rare, severe form (1:300,000) caused by mutations inherited from both parents;^84^
- LDL-c levels are typically > 400 mg/dL, with early xanthomas and a high risk of CVD during childhood;^84^
- Diagnosis: Based on extremely elevated LDL-c, clinical signs, and family history. Genetic testing confirms the condition and guides treatment and family screening;^84^
- Treatment:
  – Early initiation of statins and ezetimibe (starting at age 2);^84^
  – LDL apheresis is recommended before age 5, especially in severe cases;^84^
  – PCSK9 inhibitors (if effective), lomitapide (not yet approved for children), and evinacumab are emerging options;^84^
- LDL-c goal: < 115 mg/dL, with a lower target in those with established ASCVD, although difficult to achieve.^84^

#### 9.10.5. Hypertriglyceridemias

These result from increased VLDL production or reduced lipolysis. TG levels between 175-885 mg/dL are considered mild to moderate; levels > 885 mg/dL are classified as severe. Secondary causes include poor diet, endocrine disorders, medications, and alcohol consumption.^313 , 445^

Treatment includes: lifestyle interventions – diet and physical activity; and pharmacotherapy – statins (reduce TG by up to 30%) can be initiated from age 10; fibrates and omega-3 fatty acids may be used when TG > 400 mg/dL.^445^

#### 9.10.6. Monogenic Hypertriglyceridemia (Severe Hypertriglyceridemias)

FCS: a rare autosomal recessive disorder caused by mutations in the LPL gene and related genes. FCS typically manifests in childhood with TG levels > 1,000 mg/dL, recurrent pancreatitis, lipemia retinalis, and xanthomas. Treatment includes a diet with severe fat restriction (8%-10% of total calories) and use of medium-chain fatty acids.^97^

MCS: caused by multiple genes and worsened by factors such as diabetes, obesity, and certain medications. It is more common than FCS and responds well to lifestyle changes and treatment of comorbid conditions.^97^

#### 9.10.7. Secondary Dyslipidemias

These are caused by underlying diseases or medications. The most common include:^437^

- T1DM and T2DM
- Hypothyroidism
- CKD
- Lupus
- Isotretinoin, corticosteroids, oral contraceptives
- Liver disease

Treatment of the underlying condition usually leads to normalization of the lipid profile.

#### 9.10.8. Statin Therapy is Indicated Based on risk in Secondary Dyslipidemias, Particularly in High-Risk Conditions or in the Presence of Risk Factors (Threshold Values for Initiating Treatment)

Children > 10 years:

- LDL-c ≥ 130 mg/dL with multiple risk factors (eg, diabetes and hypertension)
- LDL-c ≥ 160 mg/dL with one risk factor, such as family history of premature heart disease or multiple mild risk factors (eg, untreated hypertension or obesity)
- LDL-c ≥ 190 mg/dL with no risk factors

**Table:** 

Recommendation	Strength of Recommendation	Certainty of Evidence
For the pediatric population, a complete lipid profile screening is recommended universally between 9 and 11 years of age.	STRONG	MODERATE
For the pediatric population with risk factors (mentioned in the text), a complete lipid profile screening is recommended starting at 2 years of age.	STRONG	MODERATE
For the pediatric population, lifestyle modification with nutritional guidance, weight control, and physical activity is strongly recommended as the first therapeutic approach when there is compatible age and clinical judgment.	STRONG	HIGH
For the pediatric population who do not reach LDL-c targets after lifestyle modification, monotherapy with statins is recommended starting at 8 years of age.	STRONG	MODERATE
For the pediatric population who remain above LDL-c targets despite lifestyle modification, the use of ezetimibe is recommended starting at 6 years of age, and combination therapy with statins is recommended starting at 8 years of age.	CONDITIONAL	LOW
For the pediatric population with clinical evaluation indicating high risk, based on LDL-c levels and patient condition, consider using evolocumab from age 10 or alirocumab from age 8.	STRONG	MODERATE

LDL-c: low-density lipoprotein cholesterol; PCSK9: proprotein convertase subtilisin/kexin type 9.

### 9.11. Transplant Recipients

Advances in rejection therapy for transplant recipients have significantly improved survival rates, making CVD the leading cause of mortality.^446^In this context, dyslipidemia plays a central role, being highly prevalent among solid organ transplant recipients. It is estimated to occur in up to 80% of kidney transplant recipients, 70% of liver recipients, and 50% of heart recipients, while prevalence in the general population is around 35%.^447 , 448^

Although factors such as genetic predisposition and pre-existing comorbidities contribute to lipid abnormalities, the primary causal factor is the use of immunosuppressants, especially calcineurin inhibitors (eg, cyclosporine and tacrolimus) and corticosteroids. These agents elevate levels of LDL-c and TG and are also associated with hypertension, insulin resistance, hyperglycemia, and diabetes. While drugs like azathioprine appear to have a neutral impact on lipid metabolism, the effects of newer therapies such as monoclonal and polyclonal antibodies still lack robust data.^448 - 450^

Cyclosporine, approved in 1983, revolutionized immunosuppression but adversely affects cholesterol metabolism: it inhibits CYP27A1, reducing bile acid synthesis and hepatic cholesterol excretion; interferes with LDL-c receptor binding; reduces LPL activity; and directly impairs pancreatic beta-cell function, inducing apoptosis and reduced insulin secretion. The dyslipidemia it causes is similar to that of corticosteroids, though with greater LDL-c increase.^451^

CVDs are the leading cause of death after heart and kidney transplants, and the second most common in liver transplants.^448 - 450^In heart transplantation, graft vasculopathy stands out — an aggressive form of atherosclerosis characterized by concentric intimal hyperplasia and progressive luminal narrowing, responsible for up to 10% of deaths.^450^

Although transplant recipients are not automatically classified as a distinct risk category, patients receiving solid organ transplants (eg, kidney, heart, and liver) are at significantly increased CV risk.

**Table 9.2:** Statins Used in Children and Adolescents

Medication	Potency	FDA Approved	Age	Dose	Mean LDL-c Decrease
**Atorvastatin**	HIGH	Yes	≥ 10 years	10-20 mg/day	40%
**Fluvastatin**	LOW	Yes	≥ 10 years	20-80 mg/day	34%
**Lovastatin**	LOW	Yes	≥ 10 years	10-40 mg/day	17-37%
**Pitavastatin**	HIGH	Yes	≥ 8 years	1-4 mg/day	23-39%
**Pravastatin**	LOW	Yes	≥ 8 years	20 mg/day (8-13 years)	23-33%
				40 mg/day (14-18 years)	
**Rosuvastatin**	HIGH	Yes	≥ 7 years	5-20 mg/day	38-50%
**Simvastatin**	MODERATE	–	≥ 10 years	10-40 mg/day	31-41%

FDA: Food and Drug Administration; LDL-c: low-density lipoprotein cholesterol.

**Tabela 9.3:** Diversos hipolipemiantes para tratamento das dislipidemias em crianças e adolescentes

Medicamento	Potência	Aprovado pelo Food and Drug Administration	Idade	Dose	Redução média do LDL-c e TG %
**Ezetimiba**	LOW	Sim*	> 6 anos	10 mg/dia	LDL-c ↓25-30%
**Resinas de troca**	LOW	Sim	< ou > 10 anos	2-8 g/dia	LDL-c ↓15% a 30%
**Terapia anti- PCSK9 (evolocumabe)**	HIGH	Sim	≥ 8 anos	420 mg/uma vez ao mês 140 mg/15 dias	LDL-c ↓45%
**Fibratos**	MODERATE	Não	≥ 10 anos	NA	TG ↓25%
**Ômega 3**	MODERATE	NA	A partir de 7 anos	1-4 g/dia	TG ↓20%
**Fitosteróis**	LOW	NA	A partir dos 2 anos	Até 2 g/dia	LDL-c ↓10 a 15%

NA: não aplicável. *FDA aprovou uso de ezetimiba ≥10 anos, porém a presente diretriz indica ≥ 6 anos de idade por conta de estudo clinico randomizado mostrando segurança e eficácia a partir dessa faixa etária.^443^

Therefore, statin therapy is strongly recommended, except during the first one to two months following liver transplantation. Because of the risk of drug interactions, it is recommended to start with low doses. Notably, simvastatin, lovastatin, and pitavastatin should be avoided in patients on cyclosporine. In such cases, the FDA also advises caution with atorvastatin, although studies suggest that 10 mg/day may be safe. Typical starting doses include 5 mg rosuvastatin, 40 mg pravastatin, or 40 mg fluvastatin, with gradual titration.^446^The combination of lovastatin, simvastatin, or pitavastatin with everolimus, tacrolimus, or sirolimus should also be avoided due to the risk of rhabdomyolysis and liver toxicity.^452 , 453^

Ezetimibe is considered a safe alternative.^55^To date, PCSK9 inhibitors have not been extensively tested in this population, although they theoretically do not pose significant drug interaction risks, as they do not rely on the cytochrome P450 system. However, clinical safety trials are needed.^451^

Managing dyslipidemia in transplant recipients requires individualized care, as the very drugs that prolong life are those that cause lipid disturbances. Careful statin use, with attention to drug interactions, is crucial. Managing dyslipidemia should be a priority, as neglecting this aspect may compromise long-term survival in these patients.

**Table:** 

Recommendation	Strength of Recommendation	Certainty of Evidence
It is recommended to consider all transplant recipients as having an increased CV risk.	STRONG	MODERATE
For all transplant recipients, lipid profiling is recommended about 2 to 3 months after transplantation.	STRONG	MODERATE
In transplant recipients, statins are recommended as the first-line treatment for dyslipidemia to reduce CV events.	STRONG	HIGH
In transplant recipients, it is not recommended against using simvastatin or lovastatin in combination with cyclosporine, tacrolimus, sirolimus, or everolimus.	STRONG	HIGH
In transplant recipients on cyclosporine, it is recommended to use a maximum dose of 5 mg rosuvastatin or 10 mg atorvastatin to avoid drug interactions.	STRONG	MODERATE
In transplant recipients on immunosuppressants, it is recommended to use statins with lower risk of rhabdomyolysis, such as pravastatin, fluvastatin, or rosuvastatin.	STRONG	MODERATE

CV: cardiovascular.

### 9.12. Chronic Liver Diseases

Statins are contraindicated in patients with decompensated liver disease or acute hepatic failure but are considered safe for lipid-lowering therapy in patients with compensated liver disease.^454 - 456^

#### 9.12.1. Metabolic Dysfunction-Associated Steatotic Liver Disease

##### 9.12.1.1. Definition

MASLD is defined as hepatic steatosis in adults who have at least one cardiometabolic risk factor (eg, overweight/obesity, T2DM, prediabetes, hypertension, or atherogenic dyslipidemia) in the absence of secondary causes.^457^

##### 9.12.1.2. Prevalence and Cardiovascular Risk

MASLD is highly prevalent and associated with an increased risk of major CV events.

##### 9.12.1.3. Reducing Cardiovascular Risk

Comorbidities associated with MASLD — such as hypertension, diabetes, obesity, and dyslipidemia — should be addressed.^457^Statins are the first-line treatment, as they target the atherogenic dyslipidemia found in MASLD and reduce ASCVD morbidity and mortality in these patients.

##### 9.12.1.4. Liver Outcomes

Cohort studies suggest that statin use in MASLD is associated with reduced all-cause mortality, reduced risk of hepatic decompensation, and lower incidence of hepatocellular carcinoma (HCC).^458^Statins also appear to reduce the progression of liver stiffness (an indicator of fibrosis), both in patients with and without advanced liver disease.^459^Meta-analyses and systematic reviews indicate that statins may improve liver function tests and reduce the degree of steatosis and fibrosis, likely due to anti-inflammatory and anti-fibrotic mechanisms.^458^

##### 9.12.1.5. Safety

Statins should not be discontinued in patients with stable chronic liver disease and normal or mildly elevated liver enzymes (up to 3× ULN), as they do not promote disease progression. Liver function tests should be monitored periodically.^339^

Ezetimibe: The use of ezetimibe in MASLD remains controversial; however, combination therapy with a statin may be used according to standard indications to achieve therapeutic goal.^460^Due to limited data, ezetimibe should be avoided in patients with more advanced hepatic dysfunction (eg, Child-Pugh B and C).

##### 9.12.1.6. Intrahepatic Cholestasis

Primary biliary cholangitis (PBC) is an autoimmune cholestatic liver disease characterized by chronic inflammation and dyslipidemia.^461^Elevated total cholesterol levels are mainly due to increased lipoprotein X, which is nonatherogenic and does not raise CV risk. Lipid-lowering agents are indicated in the presence of additional CV risk factors in this population.

Statins are not contraindicated in patients with compensated PBC but should not be used in decompensated liver disease.^462^There is no evidence that statins reduce intrahepatic cholestasis markers in PBC.^462^Studies have shown that ezetimibe is safe in patients with PBC and, when combined with statins, may be considered for patients with PBC and CV risk factors.^46^^1^

##### 9.12.1.7. Hepatic Cirrhosis

While cirrhosis was once thought to protect against atherosclerosis, it is now known that the prevalence of CAD in cirrhotic patients may be higher than in the general population. CV risk varies by the etiology of the liver disease and is higher in cirrhosis caused by alcohol, HCV (hepatitis C virus), and NASH.^463^Statins are safe and may be used in patients with Child-Pugh Class A and B cirrhosis.^463 - 465^

However, in Child-Pugh Class C cirrhosis, statins are not advised due to moderate to severe liver dysfunction, which impairs drug metabolism and increases serum levels, raising the risk of adverse effects.^463 , 464^Current evidence on the benefits of statins in reducing portal hypertension and hepatic decompensation in cirrhotic patients is scarce; thus, statins are not indicated for these outcomes.^463^

Ongoing RCTs are evaluating the impact of statins on liver outcomes in cirrhosis.

##### 9.12.1.8. Hepatocellular Carcinoma

Case-control studies, primarily among patients with viral hepatitis, have shown a 25% reduction in HCC incidence among users of lipophilic statins compared to nonusers.^463^Meta-analyses suggest statin therapy is associated with a reduced incidence of HCC; however, randomized prospective data are still needed.

**Table:** 

Recommendation	Strength of Recommendation	Certainty of Evidence
Statin use may be indicated for patients with hepatic steatosis and increased liver enzymes (up to 3× ULN) to reduce CV risk.	STRONG	MODERATE
Statin use may be indicated for patients with hepatic steatosis and increased liver enzymes (up to 3× ULN) to improve liver outcomes.	STRONG	MODERATE
Compensated chronic liver disease (Child-Pugh A and B) is not an absolute contraindication for initiating or maintaining statin or anti-PCSK9 therapy.	STRONG	MODERATE
In patients with advanced liver failure (Child-Pugh B and C), the use of ezetimibe is not recommended.	CONDITIONAL	MODERATE

CV: cardiovascular; PCSK9: proprotein convertase subtilisin/kexin type 9; ULN: upper limit of normal.

### 9.13. Acute Coronary Syndrome

Intensive cholesterol control after an ACS is a key pillar of prevention in individuals classified as very high-risk within the CV risk continuum. In recent decades, robust evidence has reinforced the importance of early initiation of potent lipid-lowering therapy, which has been proven to reduce recurrent CV events and mortality.

In 2005, Fonarow demonstrated that initiating statin therapy within the first 24 hours of hospitalization for ACS reduced early complications and in-hospital mortality.^466^In 2008, Pitt et al. showed that LDL-c levels change minimally during the first 4 days after the event, validating early cholesterol measurement as a reliable therapeutic guide.^467^

These observations have led to the standard practice of providing lipid-lowering therapy during hospitalization. Studies with PCSK9 inhibitors (eg, alirocumab and evolocumab) have shown that LDL-c levels < 40 mg/dL are associated with sustained reduction in atherothrombotic events and have a good safety profile.^203^Thus, the more intensive the LDL-c reduction, the greater the clinical benefit.

The SWEDEHEART registry showed that patients who reached their non-HDL-c goal within 2 months and maintained it long-term had lower risk, unlike those with slow treatment escalation.^468^

The AHA/ACC guidelines recommend a sequential and rational use of three drug classes: high-intensity statins, ezetimibe, and PCSK9 inhibitors. The timing of escalation depends on baseline LDL-c levels and prior statin use.^403 , 469^The ESC proposes a similar approach, starting immediately with a high-intensity statin regardless of baseline LDL-c, followed by re-evaluation within 4-6 weeks. If the LDL-c goal is not achieved, ezetimibe should be added and if needed a PCSK9 inhibitor.^55^

The strategy proposed by the ESC Acute Cardiovascular Care committee — "Strike Early and Strike Strong" — supports starting a statin + ezetimibe during the acute ACS phase, with early addition of a PCSK9 inhibitor for patients at very high CV risk. This approach is based on three pillars: (1) high risk of CV events in the first 90 days; (2) traditional stepwise escalation may delay goal attainment by up to 12 weeks; and (3) imaging studies show plaque regression and stabilization with early use of alirocumab and evolocumab.^332^

Despite strong evidence, implementation in clinical practice remains suboptimal. Barriers such as clinical inertia, unfounded concerns about statins, geographic disparities, high costs of anti-PCSK9 therapies, and health system variability contribute to low adherence. These gaps are observed even in countries with strong public health care systems.

In the future, lipid management in ACS may include emerging therapies such as inclisiran, which acts via RNA interference and offers potential for early use and long-lasting effect. Furthermore, strategies to address residual risk — such as control of HTG and increased Lp(a) after LDL-c goal achievement — may enhance secondary prevention interventions.

The current guideline recommends early initiation of combination lipid-lowering therapy in ACS, with a high-intensity statin plus ezetimibe started during hospitalization. This strategy aims to reduce therapeutic inertia, increase goal attainment for LDL-c, and provide more effective CV risk reduction. Lipid profile reassessment should occur between 4 to 6 weeks, at which point anti-PCSK9 therapy is indicated if LDL-c goals have not been achieved. In individuals at extreme CV risk, this combined approach from the beginning of hospitalization is especially recommended.^470^

**Table:** 

Recommendation	Strength of Recommendation	Certainty of Evidence
In patients with ACS, early lipid profile testing (preferably within 24 hours of the acute event) is recommended as a basis for therapeutic decisions.	STRONG	MODERATE
In patients with ACS, lipid profile testing is recommended within 4-6 weeks after hospital discharge.	STRONG	MODERATE
In patients with ACS, initiation of high-intensity statins within the first 24 hours of hospitalization is recommended.	STRONG	HIGH
In patients with ACS, it is recommended to initiate high-intensity statins plus ezetimibe during the acute phase, and to consider early use of PCSK9 inhibitors in patients at very high or extreme risk, as an intensive strategy to rapidly reduce LDL-c, minimize therapeutic inertia, and increase the likelihood of achieving lipid goals.	STRONG	MODERATE

ACS: acute coronary syndrome; LDL-c: low-density lipoprotein cholesterol; PCSK9: proprotein convertase subtilisin/kexin type 9.

### 9.14. Immune-Mediated Diseases

Patients with immune-mediated diseases such as rheumatoid arthritis (RA), SLE, spondylarthritis, and psoriasis have a significantly increased CV risk,^471^comparable to that of individuals with T2DM. The systemic inflammation typical of these conditions not only causes endothelial dysfunction and accelerates atherosclerosis but also leads to qualitative and quantitative changes in lipoproteins. Oxidation of LDL-c and dysfunction of HDL-c are observed, making them pro-atherogenic even when serum levels are within the normal range.^472^This "lipid paradox" may mask atherosclerotic risk and delay the initiation of preventive measures.

In addition, medications may contribute to secondary dyslipidemia. Glucocorticoids decrease HDL-c and increase TG and LDL-c.^473^Immunosuppressants such as cyclosporine may also raise TC and TG.

Some diseases have particularities that increase dyslipidemia and CV risk. Psoriasis, for example, is associated with a higher prevalence of obesity, hypertension, and diabetes.

Another factor to consider is disease severity and long-term control. Since traditional risk scores such as Framingham do not account for inflammatory status, CV risk is often underestimated in patients with immune-mediated diseases. Therefore, risk-adjusted approaches have been proposed for these populations.^474^

Overall, SLE, active RA, and moderate-to-severe psoriasis should be considered high-risk conditions for CVD, even in the absence of other risk factors. In cases of uncertain CV risk, CAC scoring via CT may be useful to guide therapeutic decisions.

Proper management of dyslipidemia requires a multidimensional approach, combining control of the underlying disease, lifestyle changes, and specific pharmacologic treatment. The first pillar of treatment is controlling inflammatory activity, which improves the lipid profile and reduces overall CV risk.^475^Agents such as methotrexate and hydroxychloroquine tend to improve lipid levels indirectly by controlling inflammation.

Pharmacologic treatment of dyslipidemia in patients with immune-mediated diseases follows specific guidelines adapted to the increased risk in these patients. Therapeutic targets are usually more aggressive than in the overall population and also depend on risk enhancers such as nephropathy, prolonged use of corticosteroids, or a history of CV events.^55^

Statins are the first-line treatment due to their proven effectiveness in reducing CV events and their pleiotropic anti-inflammatory effects.^476^In patients with statin intolerance or inadequate response, ezetimibe may be added, or in very high-risk cases, PCSK9 inhibitors.^477^

In addition to pharmacologic treatment, lifestyle changes are essential. These interventions are especially important because patients with immune-mediated diseases have a higher prevalence of associated risk factors such as physical inactivity and insulin resistance.

**Table:** 

Recommendation	Strength of Recommendation	Certainty of Evidence
In individuals with RA who meet high-risk criteria for immune-mediated disease, it is recommended to consider this an enhancing factor for CV risk.	STRONG	MODERATE
In individuals with immune-mediated diseases, it is recommended to adequately control inflammatory activity as an essential strategy to decrease CV risk.	STRONG	MODERATE
In individuals with immune-mediated diseases, it is recommended to use CAC score for risk stratification in patients with intermediate risk.	CONDICIONAL	HIGH
In individuals with immune-mediated diseases, it is recommended to use statins as the first-line treatment for dyslipidemia.	STRONG	MODERATE
In individuals with immune-mediated diseases: when statins are not tolerated or the response is inadequate, it is recommended to add ezetimibe or, in very high-risk cases, PCSK9 inhibitors.	STRONG	MODERATE

CAC: coronary artery calcium; CV: cardiovascular; RA: rheumatoid arthritis; PCSK9: proprotein convertase subtilisin/kexin type 9.

### 9.15. Pregnancy

During pregnancy, transient lipid profile changes (2nd and 3rd trimesters) include increases in TC and LDL-c (30%-50%), TG (50%-100%), and HDL-c (20%-40%).^478^

#### 9.15.1. Gestational Dyslipidemia in Normolipidemic Women

Transient gestational dyslipidemias are the most prevalent (20%-30% of pregnant women). When occurring early (1st trimester), they increase TG-rich lipoproteins and lower HDL-c, raising the risk of preeclampsia, gestational diabetes, persistent postpartum hypertension, and long-term ASCVD.^479^In fetuses, these changes are associated with prematurity, low birth weight, and macrosomia.^480^

#### 9.15.2. Gestational Dyslipidemia in Women with Pre-Existing Dyslipidemia

HeFH: the most common genetic dyslipidemia, characterized by high LDL-c and early CAD risk.^481^During pregnancy, levels of LDL-c increase significantly, especially since lipid-lowering therapies are stopped during pregnancy and breastfeeding. Despite high LDL-c levels, maternal-fetal outcomes do not differ between women with and without HeFH.^4^No evidence suggests that treatment discontinuation during pregnancy increases long-term CV risk.^482^

HoFH: it is far rarer and is caused by two pathogenic variants that lead to markedly increased LDL-c. This condition predisposes individuals to early atherosclerosis and aortic valve/supravalvular disease. Hormonal changes and discontinuing treatment during pregnancy can further increase levels of LDL-c, complicating management. Nevertheless, maternal cardiac events are uncommon, and there are no prospective studies that assess the risk of CV morbidity or mortality due to therapy withdrawal during gestation.^482^

HTG: severe gestational HTG is defined as plasma TG > 1,000 mg/dL. It can be due to monogenic or polygenic causes, or secondary factors (eg, uncontrolled diabetes). Maternal risks include acute pancreatitis, hyperviscosity syndrome, and preeclampsia.^483^

#### 9.15.3. Lipoprotein(a)

Lp(a) is a risk factor for both arterial and venous thrombosis. During pregnancy, it rises between the 10th and 35th weeks in 20%-30% of women.^484^Structurally similar to plasminogen, elevated Lp(a) is associated with maternal complications (eg, preeclampsia) and neonatal risks (eg, preterm birth).^484^

#### 9.15.4. Pharmacological Treatment

##### 9.15.4.1. Statins

Safety: case reports described a high incidence of structural malformations — especially in the nervous and skeletal systems — in babies exposed to lipophilic statins during the first trimester.^485^

The FDA previously classified statins as category X drugs, banning their use during pregnancy and recommending discontinuation during conception attempts, pregnancy, and breastfeeding.^486^

##### 9.15.4.2. New Evidence

Observational studies, systematic reviews, and meta-analyses have not shown an increase in the rates of congenital malformations or other harm in women exposed to statins during pregnancy. Systematic reviews and meta-analyses in women with hyperlipidemia or comorbidities and risk of preeclampsia have not demonstrated increased rates of congenital malformations or other adverse outcomes in pregnant women exposed to statins.^487 - 490^

In HoFH, a retrospective study compared pregnant women who continued statin therapy during pregnancy with those who discontinued it. No differences were found in overall pregnancy outcomes, CV complications, or congenital malformations between the two groups.^491^

New FDA Position: in pregnant women with significantly elevated cholesterol and CV risk, FDA argues that "the benefits of statins may include prevention of serious or potentially fatal events in a small group of very high-risk pregnant patients."^486^

Recommendation: statins should only be maintained during pregnancy for very high-risk patients and/or those with FH. This decision must be shared between physician and patient, considering the difficulty in assessing maternal risks of discontinuing the medication and potential fetal risks of maintaining it. While safety data on statin use during pregnancy has evolved, it remains extremely limited.

If statin therapy is maintained, it should be discontinued during the first trimester and reintroduced in the third trimester. Pravastatin is the statin with the strongest safety evidence.

##### 9.15.4.3. Bile Acid Sequestrants

Bile acid sequestrants may be used during pregnancy and are not associated with an increased risk of congenital abnormalities. However, they are generally poorly tolerated and can impair the absorption of fat-soluble vitamins.^492^There are reports of subdural hematomas in fetuses due to vitamin K deficiency in mothers under long-term cholestyramine therapy for intrahepatic cholestasis.^492^

##### 9.15.4.4. Lipoprotein Apheresis

Extracorporeal removal of ApoB-containing lipoproteins by apheresis, when available, is the preferred treatment for HoFH.^493^

##### 9.15.4.5. Ezetimibe

Not recommended during pregnancy or breastfeeding.^494^

New drugs: anti-PCSK9 therapies (evolocumab, alirocumab, inclisiran), ANGPTL3 inhibitors (evinacumab), BPA, and lomitapide are not recommended during pregnancy or breastfeeding.

Fibrates are not recommended during pregnancy.^494^However, there are a few case reports, especially from the second trimester onward. It is recommended to consider fenofibrate during the second trimester if TG > 880 mg/dL.

##### 9.15.4.6. Omega-3 Fatty Acids

Recommendation: omega-3 fatty acids may be an effective option for patients with severe HTG, especially those at risk for pancreatitis (TG > 880 mg/dL).^494^

**Table:** 

Recommendation	Strength of Recommendation	Certainty of Evidence
For pregnant women with dyslipidemia related to pregnancy or other forms of primary or secondary dyslipidemia, it is recommended to follow a low-fat diet, high in soluble fiber and low glycemic index carbohydrates.	STRONG	MODERATE
For women planning to become pregnant and previously using statins, it is recommended to discontinue statin therapy 60 days before conception.	STRONG	MODERATE
For pregnant women using statins, immediate discontinuation of the drug is recommended; it should only be restarted after the breastfeeding period.	STRONG	MODERATE
For pregnant women at very high risk, therapeutic individualization and shared decision-making are recommended, including the possible reintroduction of statins in the third trimester.	CONDITIONAL	MODERATE
For pregnant women with hypercholesterolemia, the use of bile acid sequestrants is recommended.	CONDITIONAL	LOW
For pregnant and breastfeeding women, it is recommended to avoid the use of ezetimibe, anti-PCSK9 therapies, ANGPTL3 inhibitors (eg, evinacumab), BPA, and lomitapide.	STRONG	MODERATE
For pregnant women with TG > 880 mg/dL despite lifestyle changes, the use of fenofibrate during the second trimester is recommended.	CONDITIONAL	LOW
For pregnant women with TG > 880 mg/dL despite lifestyle changes, the use of omega-3 fatty acids is recommended.	CONDITIONAL	LOW

BPA: bempedoic acid; PCSK9: proprotein convertase subtilisin/kexin type 9; TG: triglycerides.

### 9.16. Women

Women share many traditional risk factors with men; however, these factors may have a different impact on women due to biological and sociocultural differences. Additionally, there are women-specific risk factors — often under-recognized in clinical practice^14 , 495 , 496^— that should be considered risk enhancers,^497^as detailed in the risk stratification chapter.

Throughout life, women's lipid profiles display distinct patterns influenced by hormonal and physiological factors. During the reproductive phase, lipid profile fluctuations occur across the menstrual cycle, with LDL-c peaking in the follicular phase and decreasing during the luteal phase. Oral contraceptives can raise levels of TC and TG. During pregnancy, TG can physiologically double and TC may increase by approximately 1.5 times. After menopause, the drop in estrogen leads to increases in LDL-c and TG, raising atherosclerotic risk.

Oral hormone therapy during menopause reduces LDL-c and increases HDL-c but may also raise TG levels — particularly with conjugated equine estrogens or 17β-estradiol combined with progestins such as medroxyprogesterone acetate. Transdermal therapy has a neutral effect on TG, a lower hepatic impact, and preserves the positive effect on HDL-c.

Since women have specific risk factors related to hormonal changes and life stages such as pregnancy and menopause, identifying and accounting for these risk enhancers is strongly recommended by the current guideline and is fundamental to personalized risk stratification. Factors such as persistent dyslipidemia, early family history, and SAD should guide the adoption of effective therapeutic targets, without distinction between men and women. Regardless of sex, intensive treatment should be indicated according to the risk category, aiming to reduce LDL-c to guideline-recommended levels, using high-potency statins, combination therapy with ezetimibe, and, when necessary, anti-PCSK9 therapies. This therapeutic equity is essential to overcome clinical inertia and historical undertreatment, thereby expanding effective CV protection in women.

**Table:** 

Recomendação	Força da recomendação	Certeza da evidência
For women classified as low or intermediate CV risk, the use of clinical risk enhancers is recommended to refine risk stratification and guide more intensive therapeutic decisions.	STRONG	MODERATE
For women classified as high, very high, or extreme risk, intensive and combination therapy is recommended.	STRONG	HIGH

CV: cardiovascular.

## 10. Conclusion

The 2025 Brazilian Guideline on Dyslipidemias and Prevention of Atherosclerosis represents a decisive step toward effectively reducing CV risk across all life stages. On the basis of solid scientific evidence, the guideline moves beyond the traditional approach focused solely on dyslipidemia and embraces a broader vision of ASCVD prevention, integrating implementation strategies, imaging tools, and innovative biomarkers.

The 10 key messages ( Figure 10.1 ) in the current guideline consolidate the pillars of modern prevention: from promoting healthy lifestyles and assessing risk with new markers such as Lp(a) and ApoB to recognizing specific risk profiles, including risk enhancers and the category of extreme CV risk, with the adoption of more aggressive and individualized treatment targets. The lowering of LDL-c targets in individuals at low short-term risk acknowledges the importance of early and sustained LDL-c control. The inclusion of tools such as PREVENT and CAC scoring enhances the ability to reclassify and personalize care.

**Figure 10.1:** Key Messages of the Guideline.

In this new landscape, lipid-lowering therapy has evolved into a more robust and strategic arsenal, with an emphasis on reducing CV risk rather than merely correcting laboratory lipid levels. The recommendation of early combination therapy with statins, ezetimibe, PCSK9 inhibitors, and, in selected cases, BPA reflects the urgency of implementing intensive and safe interventions that improve therapeutic efficacy — especially in settings of poor adherence, statin intolerance, or difficulty achieving lipid targets.

By recognizing atherosclerosis as a progressive and silent condition that begins early and is influenced by multiple social, genetic, and clinical factors, the current guideline reinforces a commitment to a continuous and integrated approach throughout the life course. More than a technical document, it aims to serve as a tool to transform clinical practice in Brazil, promoting equity, scientific advancement, and a direct impact on population health.

Ultimately, the Brazilian Guideline on Dyslipidemias and Prevention of Atherosclerosis reconnects treatment with its true purpose: to prevent CV events, save lives, and offer future generations a longer and better life.
